# The Formosalides: Structure Determination by Total Synthesis

**DOI:** 10.1002/anie.202011472

**Published:** 2020-11-03

**Authors:** Saskia Schulthoff, James Y. Hamilton, Marc Heinrich, Yonghoon Kwon, Conny Wirtz, Alois Fürstner

**Affiliations:** ^1^ Max-Planck-Institut für Kohlenforschung 45470 Mülheim/Ruhr Germany

**Keywords:** alkyne metathesis, natural products, platinum, structure elucidation, total synthesis

## Abstract

Total synthesis allowed the constitution of the cytotoxic marine macrolides of the formosalide family to be confirmed and their previously unknown stereostructure to be assigned with confidence. The underlying blueprint was inherently modular to ensure that each conceivable isomer could be reached. This flexibility derived from the use of strictly catalyst controlled transformations to set the stereocenters, except for the anomeric position, which is under thermodynamic control; as an extra safety measure, all stereogenic centers were set prior to ring closure to preclude any interference of the conformation adopted by the macrolactone rings of the different diastereomers. Late‐stage macrocyclization by ring‐closing alkyne metathesis was followed by a platinum‐catalyzed transannular 6‐exo‐dig hydroalkoxylation/ketalization to craft the polycyclic frame. The side chain featuring a very labile unsaturation pattern was finally attached to the core by Stille coupling.

## Graphical abstract

Total synthesis allowed the identification of the most likely stereostructure of the cytotoxic marine macrolides of the formosalide family (see structure) from eight possibilities. The successful route was based upon an inherently flexible blueprint that builds all stereogenic centers by strictly catalyst controlled transformations prior to formation of the polycyclic framework by late‐stage alkyne metathesis followed by a π‐acid‐catalyzed transannular cyclization

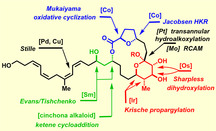

Dinoflagellates are one of the largest groups of marine eukaryotes and, as such, form a major fraction of plankton.^[1]^ In spite of their rather simple morphology, some of these unicellular organisms comprise disproportionally large genomes and, as a result, are capable of producing secondary metabolites of exceptional structural complexity and diversity. Examples include the protein phosphatase inhibitor okadaic acid,^[2]^ the family of the ladder polyether toxins (maitotoxin, brevetoxin, ciguatoxin etc.),^[3]^ the complex alkaloid saxitoxin,^[4]^ the unusual neurotoxin belizentrin,[^5^, ^6^] and the amphidinolide family of macrolides;[^7^, ^8^] this selection represents only a tiny fraction of the product spectrum but showcases the molecular intricacy as well as the potent and diverse biological activities that many dinoflagellate‐derived compounds do possess.

In 2009, Lu and co‐workers reported the isolation of two unusual cytotoxic macrolides from a marine dinoflagellate *Prorocentrum* sp.;^[9]^ this genus is known as a rich source of bioactive secondary metabolites.[^2^, ^10^] The producing organism (strain PL040104002) was found in the wash‐off epiphytes of seaweeds collected off the southern Taiwanese coastline and was mass‐cultured in the laboratory in a seawater medium. Formosalide A (**1**) and B (**2**) differ only in the anomeric substituent at C8 (Scheme 1); in view of the fact that the cell harvest was extracted with MeOH and the subsequent chromatographic purification steps used MeOH‐containing eluents,^[9]^ it is not inconceivable that the tertiary methyl ketal in **2** is an artefact of isolation. The 17‐membered lactone is reminiscent of the amphidinolide family, many members of which feature odd‐numbered rings as their principle constituents, which are puzzling from the biosynthetic viewpoint.[^7^, ^8^] Likewise, the 2,5‐*trans*‐disubstituted tetrahydrofuran and the “glycosidic” tetrahydropyran unit inscribed into the macrocyclic frame of **1** and **2** find close correspondence in the amphidinolide series and other marine natural products.^[11]^ A particularly striking substructure of the formosalides is the skipped, all‐*cis* configured diene/diene motif within the lipophilic side chain, which is expected to be highly isomerization‐prone for its thermodynamically unfavorable character.

**Scheme 1:**
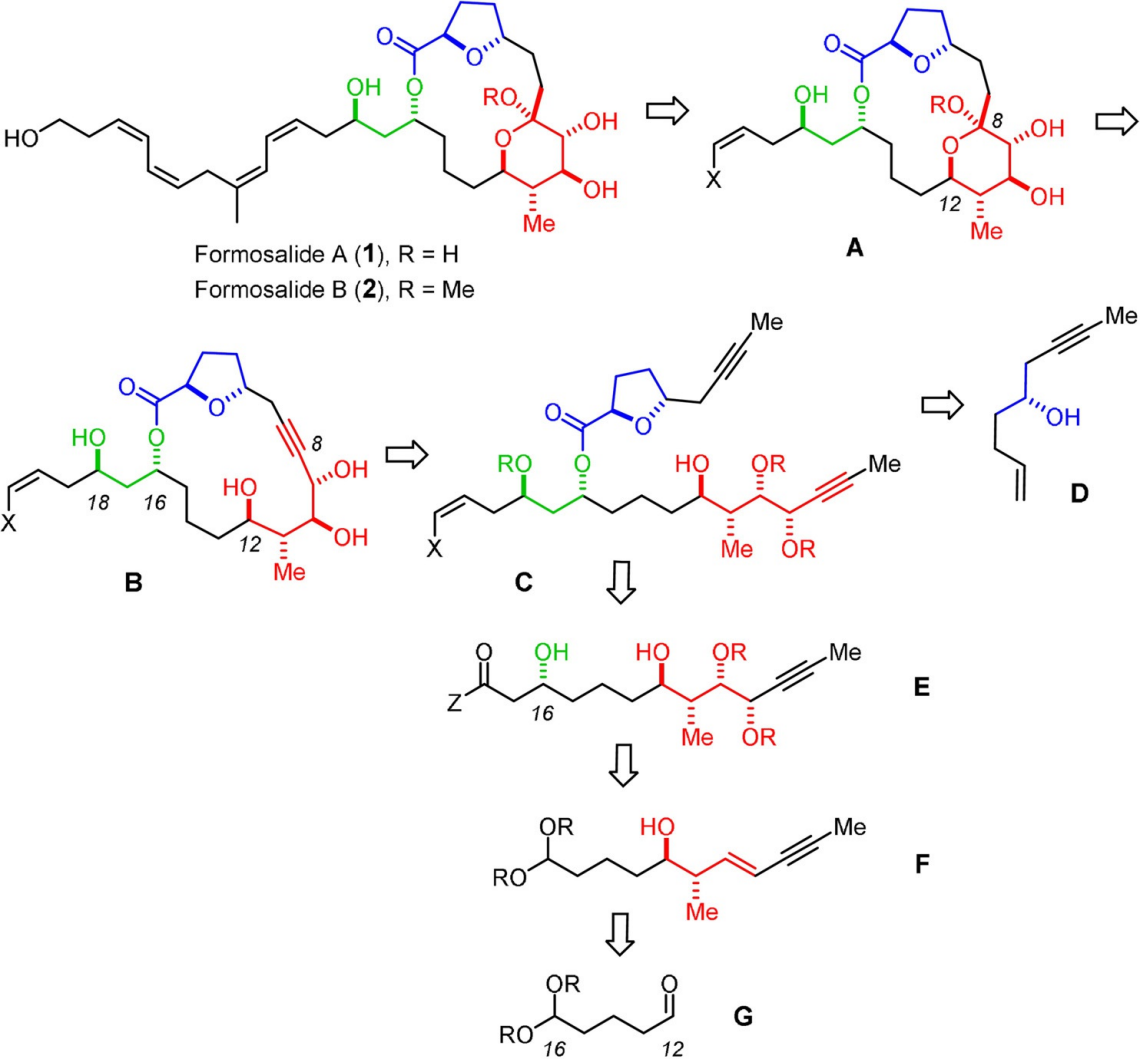
One of eight possible stereomers that might represent the formosalides: since only the relative stereochemistry of the color‐coded stereoclusters was determined by the isolation team but their inter‐relationships and the absolute configuration could not be established, eight isomers need to be considered (see the Supporting Information); blueprint of an “integral synthesis”: provided that the projected transformations proceed under catalyst (reagent) control, the entire panel of possible isomers comes into reach.

Thorough NMR studies allowed only the relative configuration within each of the three color‐coded stereoclusters to be determined but could not establish their mutual relationships nor their absolute configuration;^[9]^ eight possible combinations (four diastereomers and their respective enantiomers) have hence to be considered. Because of the flexibility of the macrocycle and the lack of crystallographic data, synthesis is arguably the most definitive way to the answer.^[12]^ A priori, such a project must be capable of producing any conceivable isomer.^[13]^ We reasoned that the blueprint sketched in Scheme 1 qualifies for such an “integral approach”:^[14]^ specifically, the late‐stage attachment of the polyene side chain seemed advisable for the sake of convergence and for stability reasons alike. Convergence is also imperative for the assembly of the eight diastereomeric macrocyclic cores that are potentially needed. To reach this goal, we opted for a strategy that puts all pertinent stereocenters in place in a strictly catalyst‐controlled fashion,^[15]^ capable of overturning, if necessary, the inherent bias of the individual substrates. Since macrocyclic stereocontrol can be significant,^[16]^ it was planned to do so *prior* to ring closure in order to prevent any interference of the conformations adopted by the macrolactones, which will vary across the different diastereomers. As a consequence, the two critical cyclization reactions necessarily become late‐stage events: it was planned to convene them at the “anomeric” position by taking advantage of the fact that a *tert*‐hemiketal has the same formal oxidation state as the C atom of an alkyne.^[17]^ When encoded in the form of a triple bond, a sequence of ring closing alkyne metathesis (RCAM)[^18^, ^19^] (**C** → **B**) followed by transannular functionalization of the resulting cycloalkyne (**B** → **A**) should allow the macrocyclic frame and the yet missing ketal subunit to be formed. The latter maneuver, however, bore considerable risk since 6‐*exo*‐dig hydroalkoxylations of alkynes in general are challenging;[^20^, ^21^, ^22^] in the projected transannular format, the conformation of the macrocycle might intervene.[^23^, ^24^] If successful, however, such a merger of RCAM and π‐acid catalysis^[25]^ streamlines the assembly process and hence minimizes the synthetic exertion.

The preparation of the required building blocks in all necessary stereochemical renditions started from aldehyde **3** derived from cyclopentene in one step (Scheme 2).^[26]^ When subjected to an iridium catalyzed Krische propargylation with **14**,[^27^, ^28^] multigram quantities of **4** were secured, basically as a single isomer (dr >20:1, *ee* ≥95 %).[^29^, ^30^] Since the C−C bond formation is catalyst‐controlled, it suffices to use the enantiomeric SEGPHOS ligand to obtain *ent*‐**4** analogously (see the SI). PMB protection of the newly formed alcohol, unmasking of the alkyne, followed by a Cy_2_BH‐entrained hydroboration of **5** in neat HB(pin)[^31^, ^32^] set the stage for chain extension by Suzuki coupling of the resulting alkenyl boronate **6** with freshly prepared 1‐iodo‐1‐propyne.^[33]^ As expected for an *E*‐configured enyne, the Sharpless asymmetric dihydroxylation of **7** worked well using (DHQ)_2_PYR as the ligand in the presence of methanesulfonamide as additive^[34]^ to give diol **8** in excellent yield with a dr=9:1.^[35]^ The analogous reaction of *ent*‐**7** under the aegis of the pseudoenantiomeric (DHQD)_2_PYR furnished *ent*‐**8** with similar efficiency and selectivity.

**Scheme 2:**
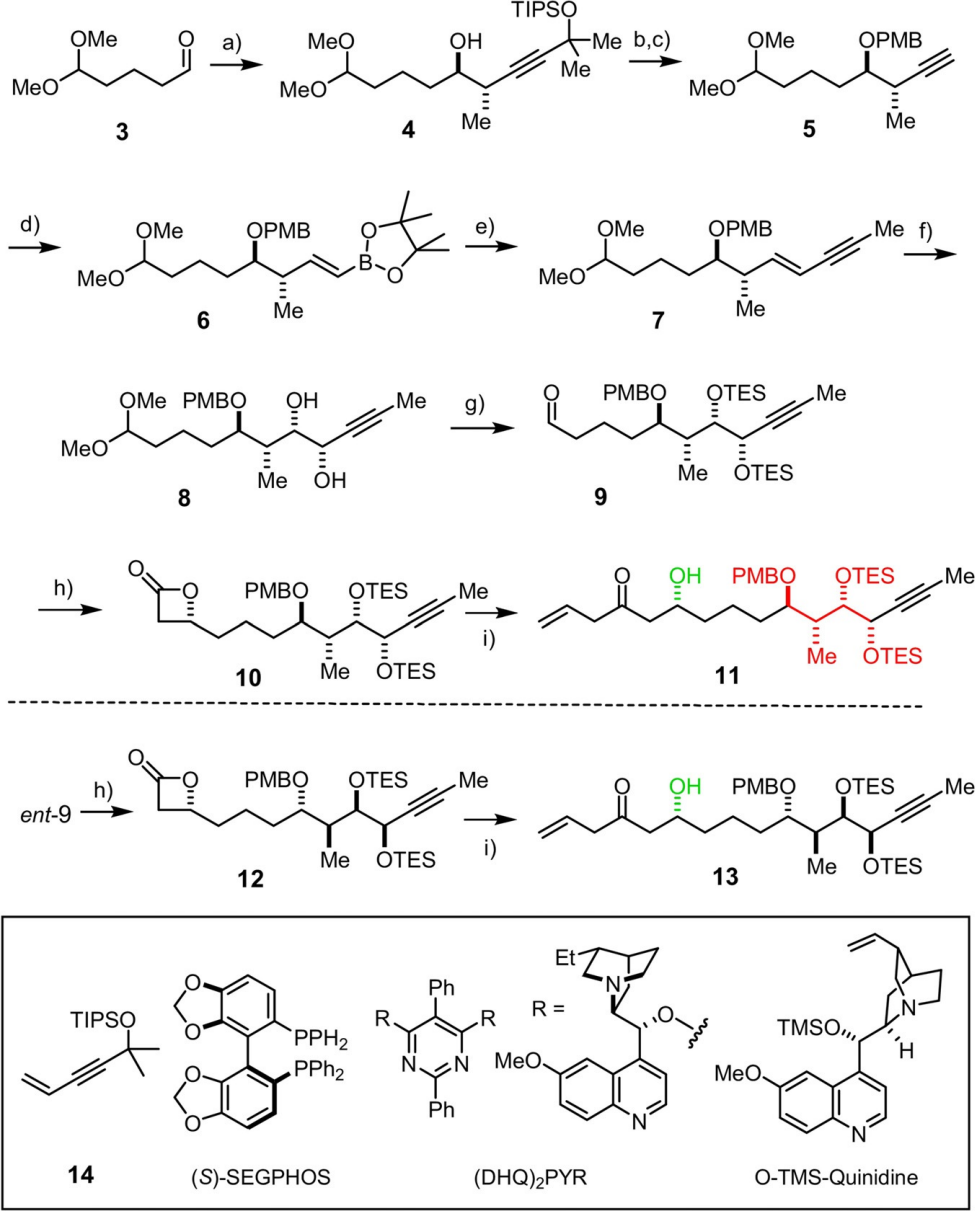
a) [{Ir(cod)Cl}_2_] (2.5 mol %), (*S*)‐SEGPHOS (5.0 mol %), **14**, formic acid, Na_2_SO_4_, THF, 60 °C, 56 % (7.8 g scale, dr >20:1, >95 % *ee*); b) NaH, PMBCl, Bu_4_NI (10 mol %), DMF, 0 °C→RT, 91 % (9.1 g scale, 98 % *ee*); c) TBAF, THF, then NaOH, toluene, reflux, 86 % (4.6 g scale); d) (pin)BH, Cy_2_BH (10 mol %), neat, 82 % (4.6 g scale); e) [Pd(PPh_3_)_4_] (5 mol %, aq. NaOH (2 M), 1‐iodo‐1‐propyne, THF, 80 °C, 69 % (2.8 g scale); f) (DHQ)_2_PYR (5 mol %), K_2_OsO_4_⋅2 H_2_O (2.1 mol %), K_3_Fe(CN)_6_, K_2_CO_3_, *t*BuOH/H_2_O, MeSO_2_NH_2_, 0 °C, 94 % (dr=9:1, 2.9 g scale); g) TESOTf, 2,6‐lutidine, CH_2_Cl_2_, 0 °C, 63 % (1.3 g scale); h) acetyl chloride, *O*‐TMS‐quinidine (10 mol %), LiClO_4_, *i*Pr_2_NEt, CH_2_Cl_2_/Et_2_O, −78 °C, 78 % (**10**, dr=19:1, 1.1 g scale), 84 % (**12**, dr=10:1, 1.0 g scale); i) (i) Me_3_Al, *N*,*O*‐dimethylhydroxylamine hydrochloride, CH_2_Cl_2_, 0 °C→RT, then **10** (or **12**), 0 °C; (ii) allylmagnesium chloride, THF, −78 °C→0 °C, 88 % (**11**, 1 g scale), 95 % (**13**, 0.9 g scale); cod=1,5‐cyclooctadiene, Cy=cyclohexyl, pin=pinacolato, PMB=*p*‐methoxybenzyl, TBAF=tetra‐*n*‐butylammonium fluoride, TES=triethylsilyl, TIPS=tri(isopropyl)silyl, TMS=trimethylsilyl.

With the C9–C12 stereotetrad representing the future tetrahydropyran unit of formosalide being established in both enantiomeric formats, we next sought to set the second stereocluster at C16/C18. Treatment of **8** with TESOTf/2,6‐lutidine^[36]^ afforded aldehyde **9** in readiness for chain extension via an acetate aldol equivalent. The asymmetric ketene cycloaddition catalyzed by *O*‐TMS quinidine served this purpose exceedingly well in that it converted **9** into β‐lactone **10** (78 %, dr=19:1), but *ent*‐**9** into the *diastereomeric* β‐lactone **12** (84 %, dr >20:1 after flash chromatography);^[37]^ not only is the reaction practical and scalable, but doubly diastereoselective to the extent needed in the current endeavor.^[38]^

With this important aspect rigorously confirmed, it was clear that all stereoisomers of **C** were within reach. Therefore, the preparation of the yet missing isomers was postponed until after the other critical steps of the projected synthesis had been validated. For proof‐of‐concept, two truncated formosalide‐like macrolides of type **A** (X=H, R=Me) with simple alkene termini were initially targeted. To this end, **10** was first converted into the corresponding Weinreb amide,^[39]^ which was then transformed into the β,γ‐unsaturated ketone **11** on treatment with allylmagnesium chloride.[^40^, ^41^] The diastereomeric compound **13** was obtained analogously, thus setting the stage for fragment coupling with concomitant 1,3‐*anti* reduction via a samarium‐catalyzed Evans–Tishchenko redox esterification.[^42^, ^43^, ^44^] The required aldehyde **18** was attained in either enantiomeric form starting from **16** by a cobalt‐catalyzed oxidative cyclization which forms the *trans*‐disubstituted tetrahydrofuran ring with excellent selectivity (Scheme 3).^[45]^ This exact transformation had already served our previous total synthesis of amphidinolide C and F;^[46]^ it illustrates the chemoselectivity of such oxidative Mukaiyama cyclization reactions in that only the olefinic site of **16** is engaged by the cobalt catalyst,[^45^, ^47^] whereas the triple bond remains untouched.[^13a^, ^13b^, ^46^]

**Scheme 3:**
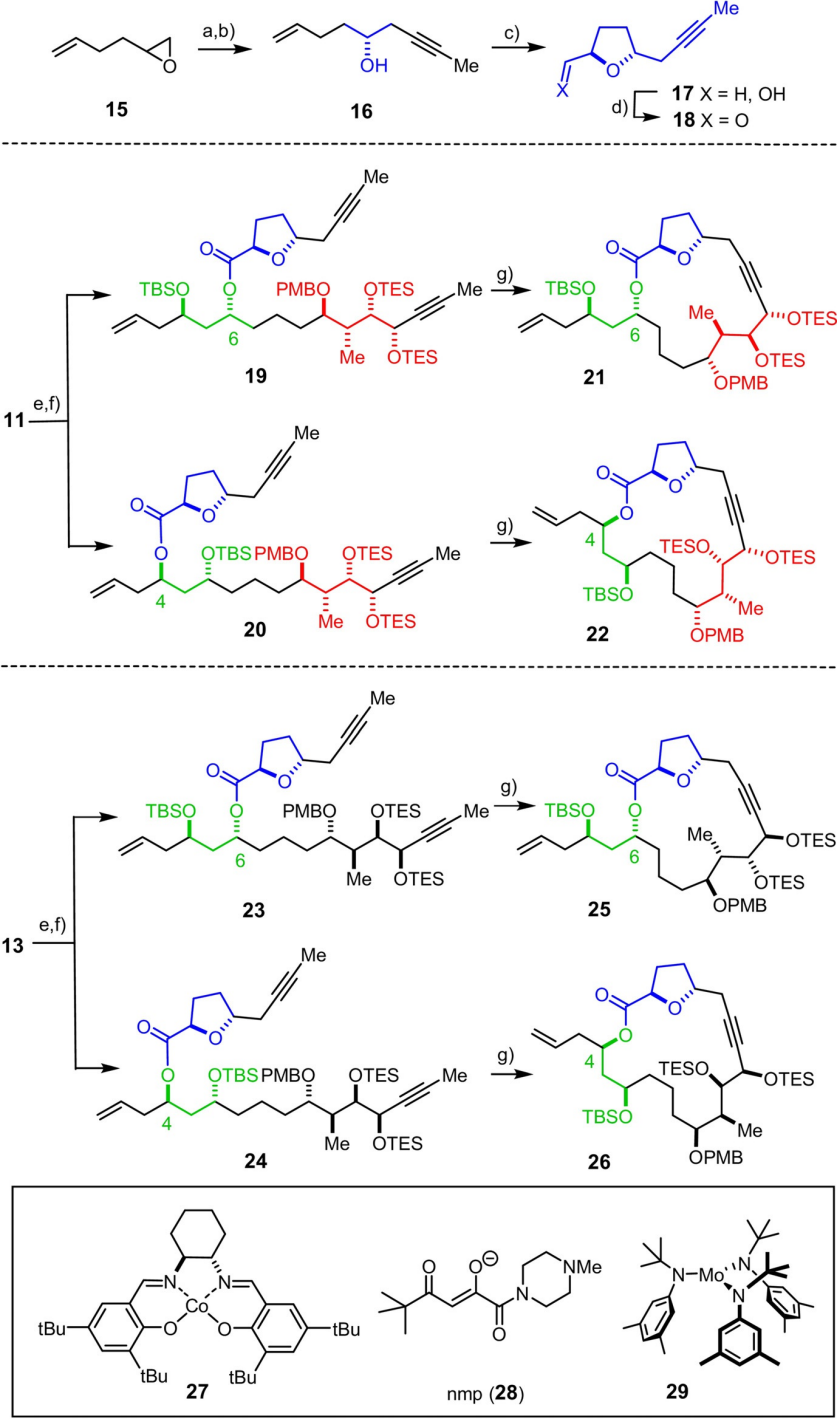
a) (*R*,*R*)‐**27**, HOAc (15 mol %), H_2_O, toluene, 0 °C→RT, 36 % (>99 % *ee*, 7.5 g scale); b) propyne, *n*BuLi, BF_3_⋅Et_2_O, THF, −78 °C, 87 % (6.1 g scale); c) Co(nmp)_2_ (10 mol %), *t*BuOOH, O_2_ (1 atm), *i*PrOH, 55 °C, 84 % (dr >20:1, 3.8 g scale); d) [SO_3_⋅pyridine], DMSO, *i*Pr_2_NEt, CH_2_Cl_2_, 89 % (1 g scale); e) (i) SmI_2_, PhCHO, THF; (ii) **18**, **11** (or **13**) f) TBSOTf, 2,6‐lutidine, CH_2_Cl_2_, 0 °C, 68 % (over two steps, **19**+**20** (3:1)), 64 % (over two steps, **23**+**24** (4:1)); g) **29** (30 mol %), CH_2_Cl_2_, toluene, 110 °C, 82 % (**21**+**22**), 77 % (**25**+**26**); TBS=*tert*‐butyldimethylsilyl.

With both partners in hand, fragment coupling via an Evans–Tishchenko reaction could be tackled. Despite the excellent track record of this transformation,^[43]^ the specific application to fragments **11** and **18** turned out to be exceptionally taxing (Scheme 3): the catalytic (substoichiometric) variant proved erratic and was not pursued further; even the reaction using over‐stoichiometric amounts of [(PhCHO)_2_SmI⋅SmI_3_] as the promoter gave product mixtures, despite considerable experimentation. Although the 1,3‐*anti* reduction per se proceeded nicely, a mixture of the *regioisomeric* esters **19** and **20** was formed. This outcome is perplexing in view of the generally accepted mechanism, which involves a highly ordered chairlike transition state that does not put the site of ester formation at risk.[^42^, ^43^] The same scrambling also plagued the reaction of the diastereomeric reagent combination **13** + **18** that furnished the constitutional isomers **23** and **24**. We are unaware of any precedent and are inclined to believe that the observed transesterification reflects an acyl migration occurring post factum but in situ. The additional O‐donor in the THF ring of the aldehyde partner **18**, which might transiently ligate the oxophilic Sm^III^ cation, could play a role.^[48]^ In any case, Evans–Tishchenko reactions using (aliphatic) aldehydes with heteroatom sites in vicinity are rare, and problems have occasionally been reported.[^44a^, ^49^, ^50^, ^51^]

As the separation of the regioisomeric esters was tedious, the crude material was O‐silylated and the resulting mixture subjected to ring closing alkyne metathesis (RCAM) using a catalyst generated in situ by activation of **29** with CH_2_Cl_2_, as previously described by our group.[^52^, ^53^] The resulting 17‐ and 19‐membered macrolides **21** and **22** can be separated by ordinary flash chromatography. The macrocyclization worked equally well in the diastereomeric series (**25**/**26**), thus showing the robustness of RCAM.[^18^, ^19^]

Subsequent oxidative cleavage of the PMB ether gave alcohols **30** and **33** in readiness for acetal formation via π‐acid‐catalyzed transannular addition of the free ‐OH group across the triple bond,^[20]^ to be followed by addition of MeOH (or H_2_O) to the resulting enol ether to form the desired (hemi)ketal (Scheme 4).^[20]^ [(C_2_H_4_)PtCl_2_]_2_ (Zeise's salt) in Et_2_O proved to be the promotor of choice for the 6‐*exo*‐dig hydroalkoxylation step,[^20^, ^54^] which has apparently not yet found any application in transannular format.[^20^, ^55^, ^56^] For the exceptional sensitivity of the resulting enol ether **31**, it proved mandatory to supplement the Pt^II^ catalyst with 2,6‐di‐*tert*‐butylpyridine to avoid instant decomposition;^[57]^ even with this precaution, loss of material could not be fully suppressed. Gratifyingly, the diastereomeric product **34** proved somewhat more stable. In any case, (crude) **31** and **34** were transformed without delay into the corresponding methyl ketals on treatment with TBAF followed by addition of MeOH promoted by admixed TMSCl. It is notable that both transannular addition reactions proceeded with the same regiochemical outcome, but the macrocyclic conformational diversity impact strongly on the stability of the products and hence the efficiency of this step.

**Scheme 4:**
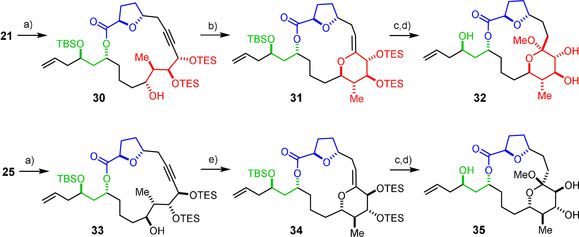
a) DDQ, CH_2_Cl_2_, phosphate buffer (pH 7), 0 °C, 47 % (**30**), 96 % (**33**); b) [(C_2_H_4_)PtCl_2_]_2_ (50 mol %), 2,6‐di‐*tert*‐butylpyridine, Et_2_O; c) TBAF, THF; d) TMSCl, MeOH, 0 °C, 19 % (**32**, unoptimized, over three steps (b–d)); 44 % (**35**, unoptimized, over two steps (c,d)); e) [(C_2_H_4_)PtCl_2_]_2_ (3 mol %), Et_2_O, 77 %; DDQ=2,3‐dichloro‐5,6‐dicyano‐*p*‐benzoquinone.

We were delighted by the serendipitous finding that the NMR data of **32** showed an astounding match with those of formosalide B, whereas those of isomer **35** were clearly different (see the SI).^[58]^ Since the spectra of the different diastereomers are obviously readily distinguishable^[59]^ and based on the assumption that the presence/absence of the extended side chain is unlikely to alter the spectral fingerprints of the core, we saw ourselves in the favorable position that the preparation of a single product isomer rather than the entire ensemble might allow the stereostructure of formosalide to be ascertained with confidence. To this end, the approach to the model compounds was simply rerouted by attaching a handle for late‐stage introduction of the side chain (Scheme 5). Specifically, **30** was subjected to a modified Lemieux‐Johnson oxidation to cleave the terminal alkene without interference of the triple bond.^[60]^ The resulting crude aldehyde **36** underwent a Stork–Zhao olefination to form the required *Z*‐alkenyl iodide **38** as the only double bond isomer, provided that reaction was carried out in DMPU/THF (4:1).^[61]^ Interestingly, however, small amounts of epoxide **37** were also isolated, which seems to originate from a side reaction reminiscent of the Corey–Chaykovsky epoxidation using sulfur (rather than phosphorus) ylides.^[62]^ After cleavage of the PMB ether, the resulting compound **39** was subjected to the platinum‐mediated transannular cyclization in buffered medium followed by addition of MeOH and concomitant cleavage of the TES groups under slightly acidic conditions to give the expected methyl ketal **40** in readiness for chain extension by Stille–Migita coupling to complete the formosalide carbon skeleton. As the configuration of **40** matches that of the much more sensitive model **32**, the transannular addition/ketalization step faced the same issue of passing through an exceptionally fragile intermediate; each of numerous experiments furnished the desired product, yet the scatter in the yield was extreme (15–85 %), despite the greatest care.

**Scheme 5:**
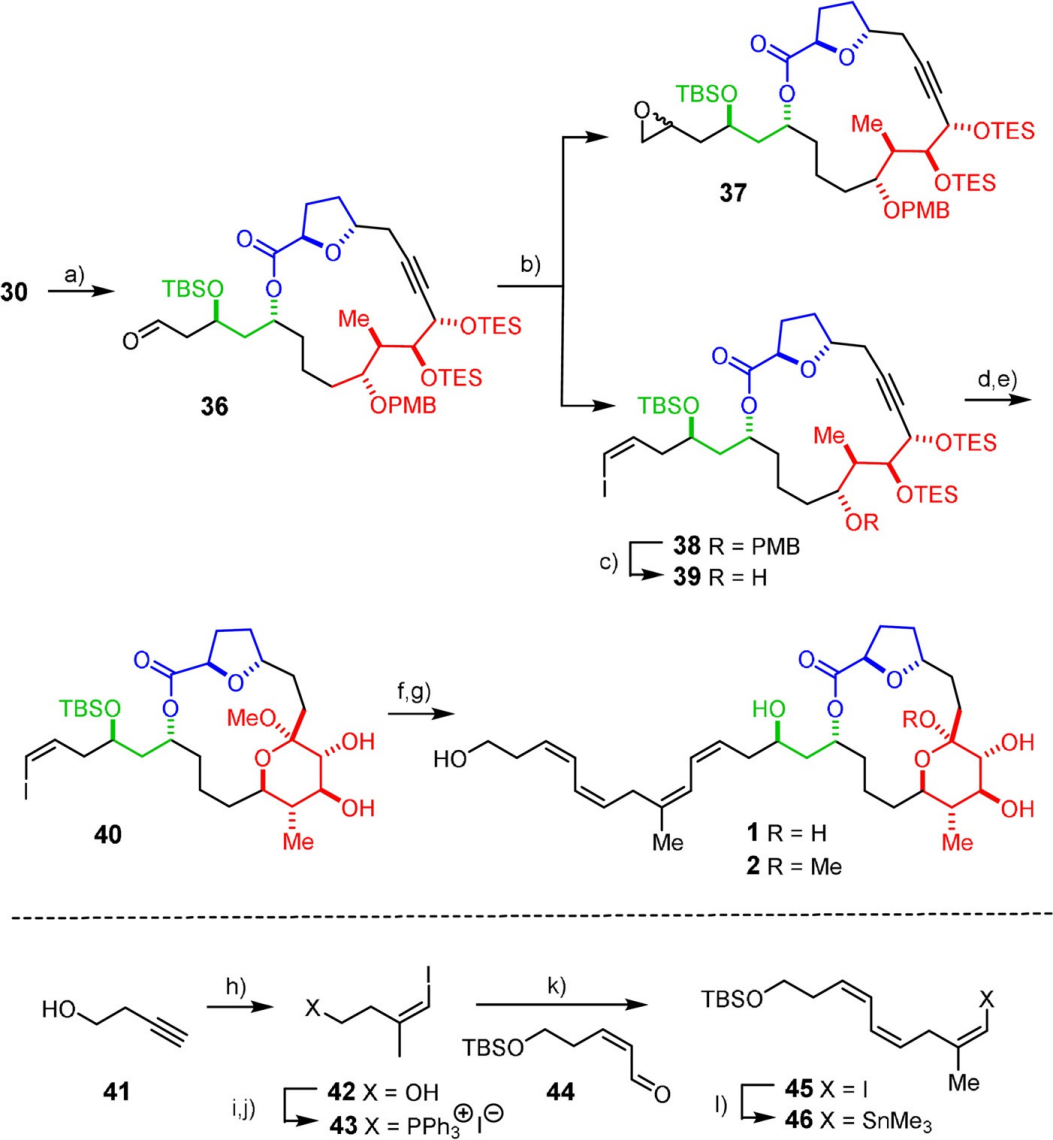
a) OsO_4_ (3 mol %), NaIO_4_, 2,6‐lutidine, 1,4‐dioxane/H_2_O, 88 %; b) (i) (iodomethyl)triphenylphosphonium iodide, NaHMDS, THF; (ii) **36**, DMPU/THF, −78 °C, **38** (53 %, *Z*:*E*>20:1) + **37** (9 %, dr=1.8:1); c) DDQ, CH_2_Cl_2_, phosphate buffer (pH 7), 0 °C, 62 %; d) [(C_2_H_4_)PtCl_2_]_2_ (50 mol %), 2,6‐di‐*tert*‐butylpyridine, Et_2_O; e) TMSCl, MeOH, 0 °C, 15–85 % (over two steps, see text); f) **46**, Pd(PPh_3_)_4_ (20 mol %), CuTC, [Ph_2_PO_2_][NBu_4_], DMF, 0 °C→RT, 62 %; g) TASF, aq. DMF, 0 °C→RT, 41 % (**1**) + 49 % (**2**); h) (i) Me_3_Al, Cp_2_ZrCl_2_, 1,2‐dichloroethane, H_2_O cat., RT→reflux; (ii) I_2_, Et_2_O, −30 °C→RT, 61 %; i) TsCl, Et_3_N, CH_2_Cl_2_, 0 °C→RT, 78 %; j) (i) NaI, acetone, reflux; (ii) PPh_3_, MeCN, 82 %; k) LiHMDS, THF, −78 °C, then **44**, DMPU, −78 °C, 59 %; l) Me_3_SnSnMe_3_, [(PPh_3_)_2_PdCl_2_] (5 mol %), THF, 35 °C, 79 %; CuTC=copper thiophene‐2‐carboxylate, DMPU=1,3‐dimethyl‐3,4,5,6‐tetrahydro‐2(1*H*)‐pyrimidinone, HMDS=hexamethyldisilazide, TASF=tris(dimethylamino)sulfur trimethylsilyl difluoride, Ts=*p*‐toluenesulfonyl.

As expected, the required stannane partner **46** is a very delicate compound, the preparation of which mandated careful optimization in order to prevent isomerization from occurring. The successful route (Scheme 5) started from commercial 3‐butyn‐1‐ol (**41**), which was converted via the known alkenyl iodide **42** ^[63]^ into the phosphonium salt **43** which underwent a *Z*‐selective Wittig reaction with enal **44**. The resulting product **45** had to be handled with greatest care in the dark to avoid instant scrambling. The same threat overshadowed its subsequent conversion into stannane **46** on treatment with hexamethylditin and catalytic amounts of [(Ph_3_P)_2_PdCl_2_]:^[64]^ the reaction mandates heating but produces a mixture of isomers as soon as the temperature raises to ≥40 °C. When strictly monitored, however, the desired alkenyl stannane **46** was secured in good yield and purity.

With **40** and **46** in hand, the critical Stille–Migita coupling was performed under conditions previously developed in this laboratory for fragile polyunsaturated compounds and other demanding cases.^[65]^ It is the combination of Pd(PPh_3_)_4_ as catalyst with CuTC as promotor and [Ph_2_PO_2_][NBu_4_] as an effective yet essentially neutral tin scavenger that allowed the reaction to proceed even at 0 °C such that the configurational integrity of all stereochemical elements was preserved.[^13c^, ^66^] The remaining TBS ether in the resulting product was instantly deprotected with TASF in aqueous DMF; although these conditions are known as particularly mild,^[67]^ we observed partial hydrolysis of the ketal. In the present context, this unexpected outcome was deemed a fortuitous coincidence as it furnished synthetic formosalide A (**1**) and (**2**) in a single operation for independent comparison with the spectra of the natural products. As forecasted by the model compound **32**, the recorded NMR data of synthetic **2** were in excellent agreement with those of the authentic formosalide **B** reported in the literature. The comparison of synthetic **1** with formosalide A proved more difficult because this compound shows an interesting behavior in that the NMR spectrum is not static: a gentle drift of the ^13^C NMR signals with time and concentration is observed (see the SI), which is tentatively ascribed to slow conformational changes that come along with reorganization of the peripheral hydrogen bonding network. This phenomenon is well documented for polyhydroxylated macrocycles;^[68]^ despite the slight fuzziness, the match between recorded and reported data can rightfully be regarded as excellent too. Moreover, the sign and magnitude of the [*α*]_D_ value of both compounds also correspond well to those of the natural products (see the SI).^[9]^

Therefore, we claim with confidence that the relative and absolute configuration of formosalide A and B are correctly described by structures **1** and **2**, respectively. The project had originally been designed to cover the entire ensemble of eight conceivable isomers, as required for an ultimate proof. For favorable circumstances, however, the preparation of three model compounds^[58]^ and of a single product diastereomer sufficed to make a convincing case, allowing us to establish the most likely stereostructure of these marine natural products. The project bears witness of the excellent level of contemporary catalyst‐controlled asymmetric synthesis as well as of the great strides that macrocycle formation owes the advent of metathesis in general and RCAM in particular. These methods form a sound basis for future forays of our laboratory into transannular chemistry, a highly promising field in which a similar level of confidence has yet to be attained.

## Conflict of interest

The authors declare no conflict of interest.

## Supporting information

As a service to our authors and readers, this journal provides supporting information supplied by the authors. Such materials are peer reviewed and may be re‐organized for online delivery, but are not copy‐edited or typeset. Technical support issues arising from supporting information (other than missing files) should be addressed to the authors.

Supplementary

Supplementary
