# Supplementary material for: The Formosalides: Structure Determination by Total Synthesis
Source: Angew Chem Int Ed Engl. 2020 Nov 3;60(1):446–54. doi: 10.1002/anie.202011472 (PMC7821135; doi:10.1002/anie.202011472)

(5*S*,6*R*)-1,1-Dimethoxy-6,9-dimethyl-9-((triisopropylsilyl)oxy)dec-7-yn-5-ol (*ent*-4)

^{1}H NMR (400 MHz, $CDCl_3$)

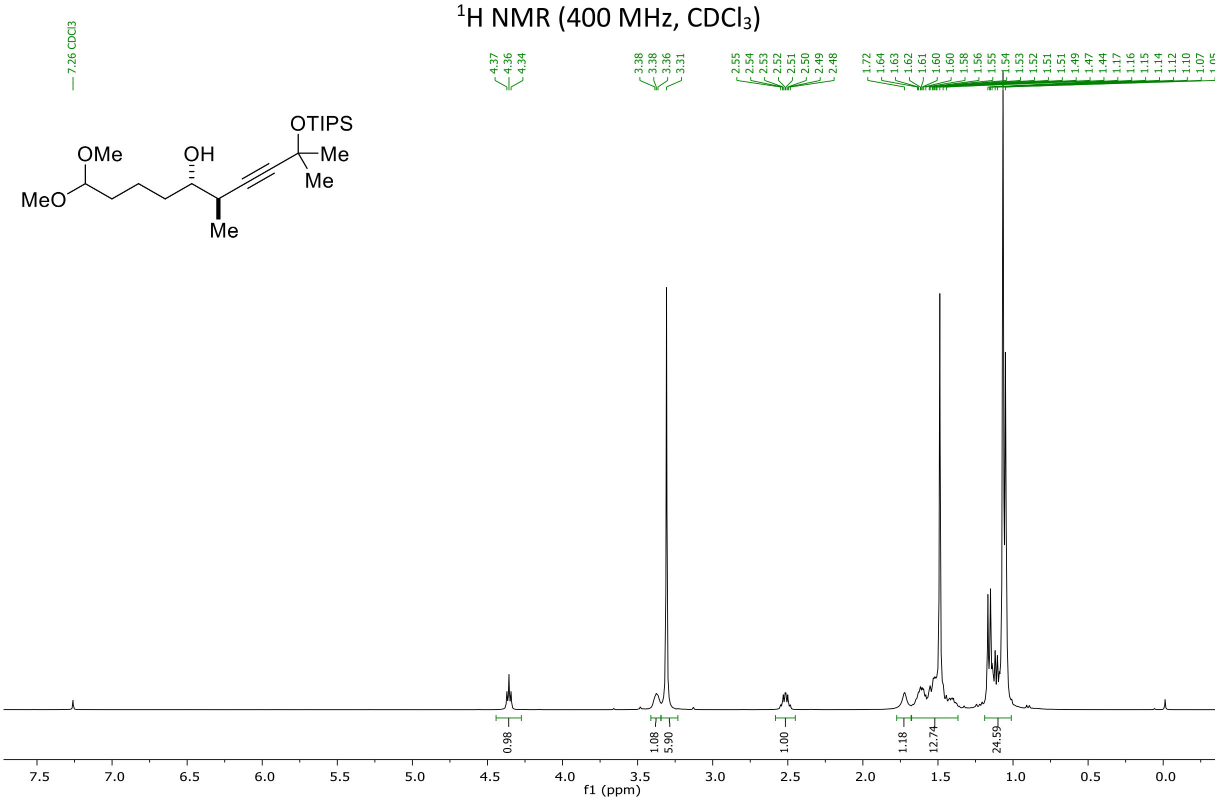

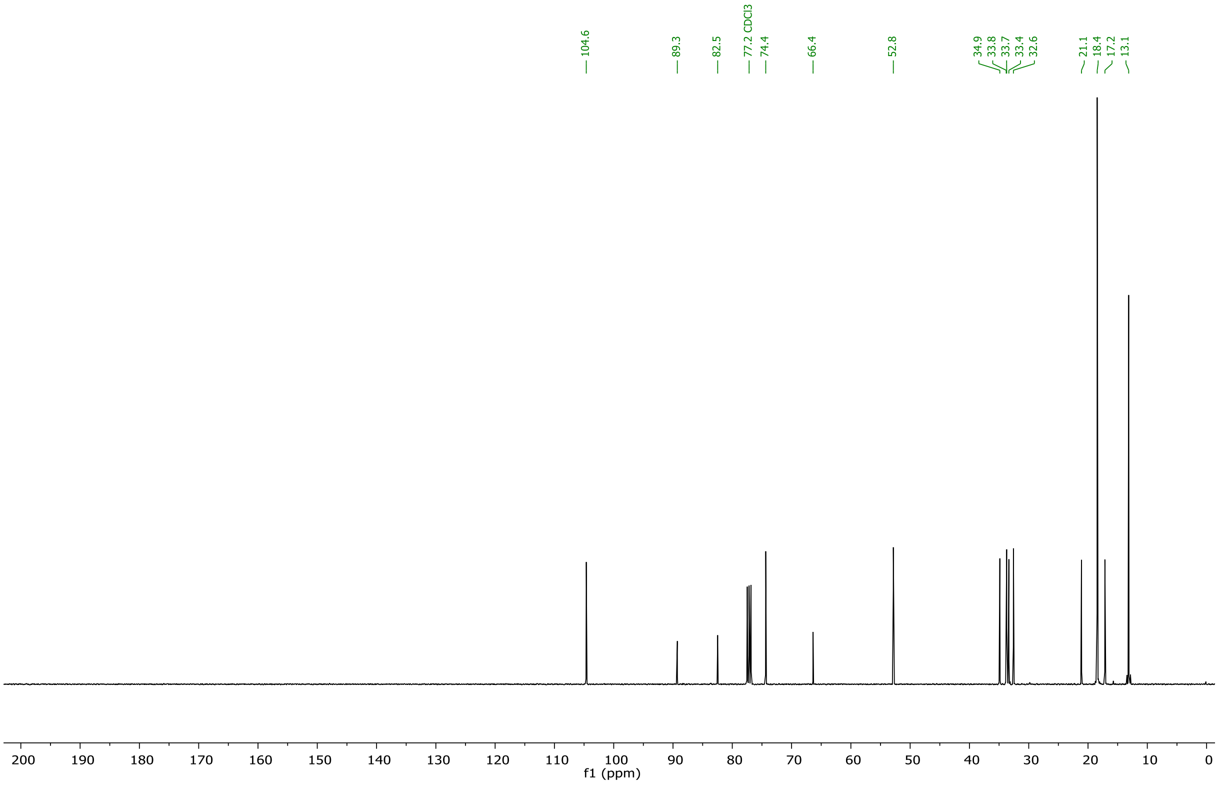

^{13}C NMR (101 MHz, $CDCl_3$)

PMB-ether ***ent*-S2**

^{1}H NMR (400 MHz, $CDCl_3$)

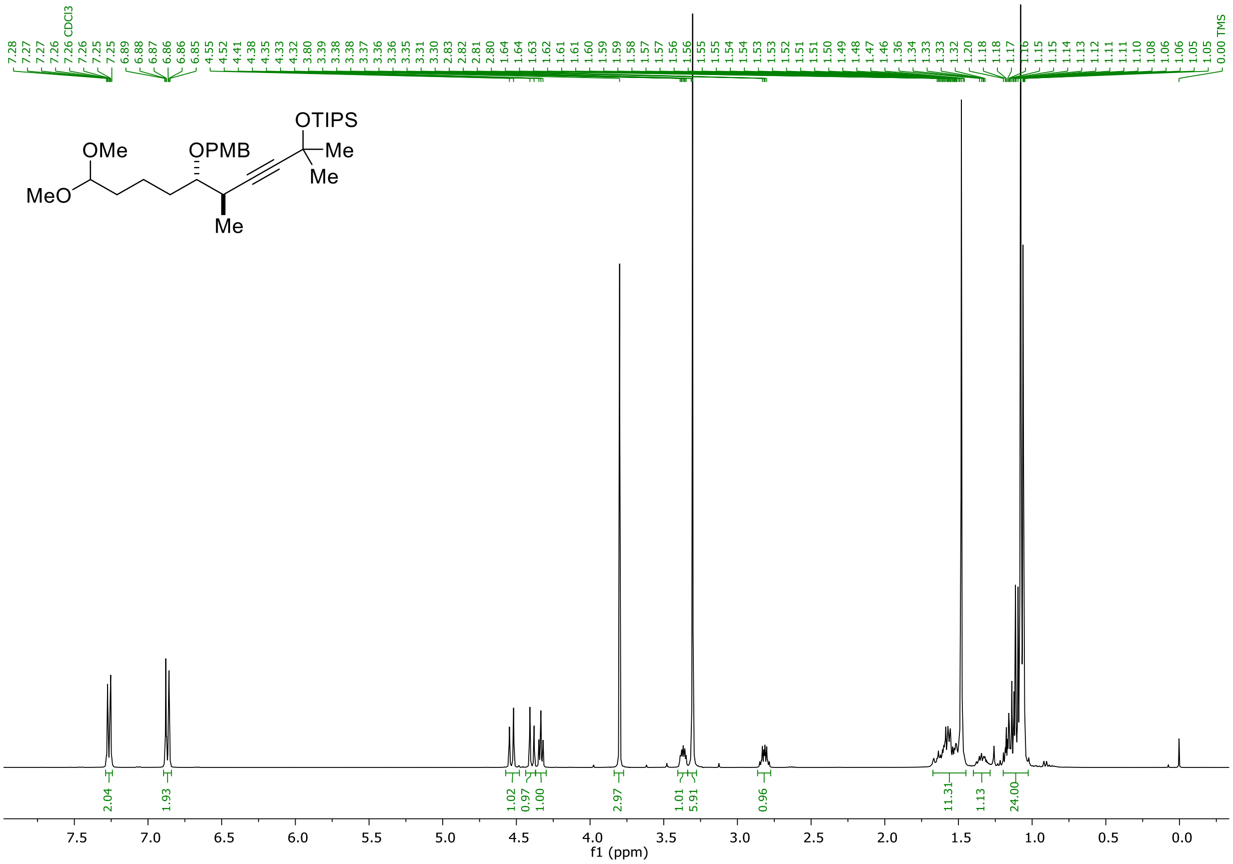

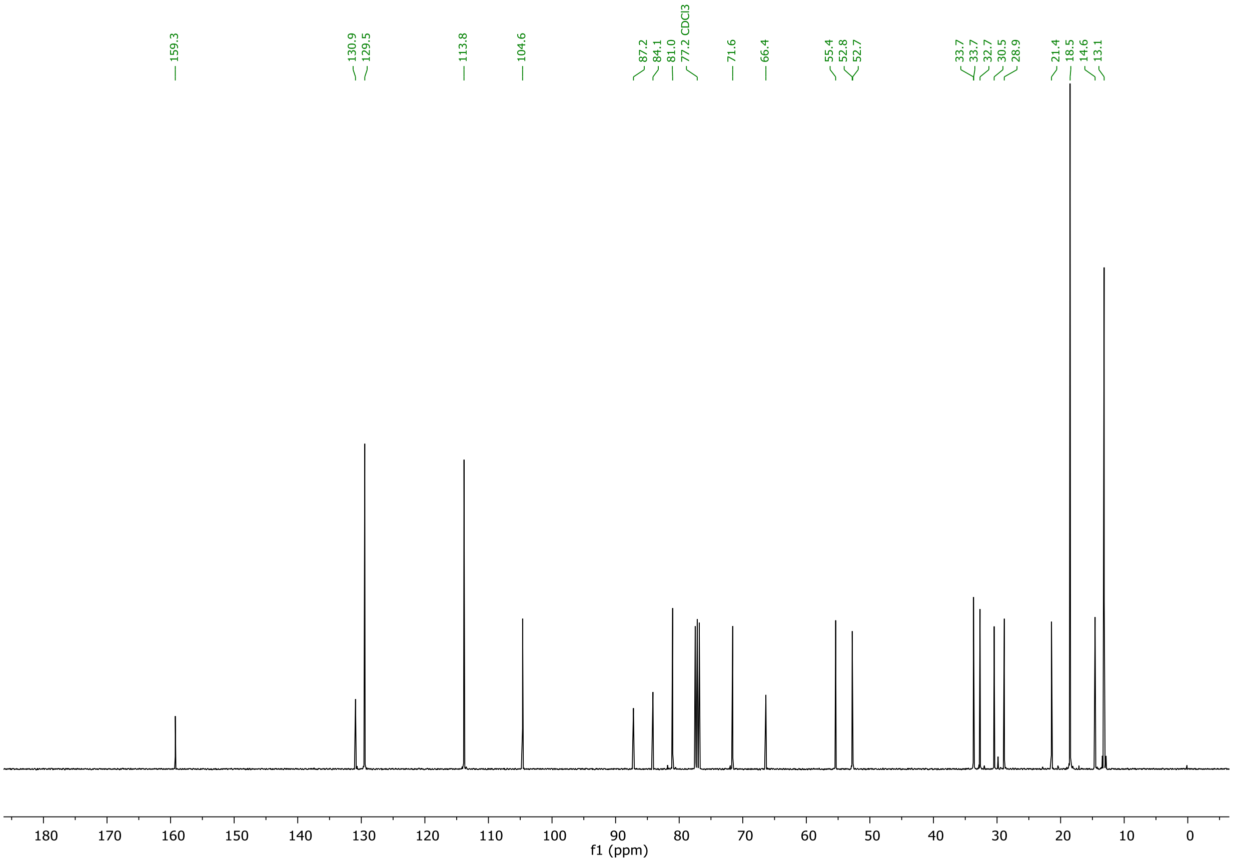

^{13}C NMR (101 MHz, $CDCl_3$)

1-((((3*R*,4*S*)-8,8-Dimethoxy-3-methyloct-1-yn-4-yl)oxy)methyl)-4-methoxybenzene (*ent*-5)

^{1}H NMR (400 MHz, $CDCl_3$)

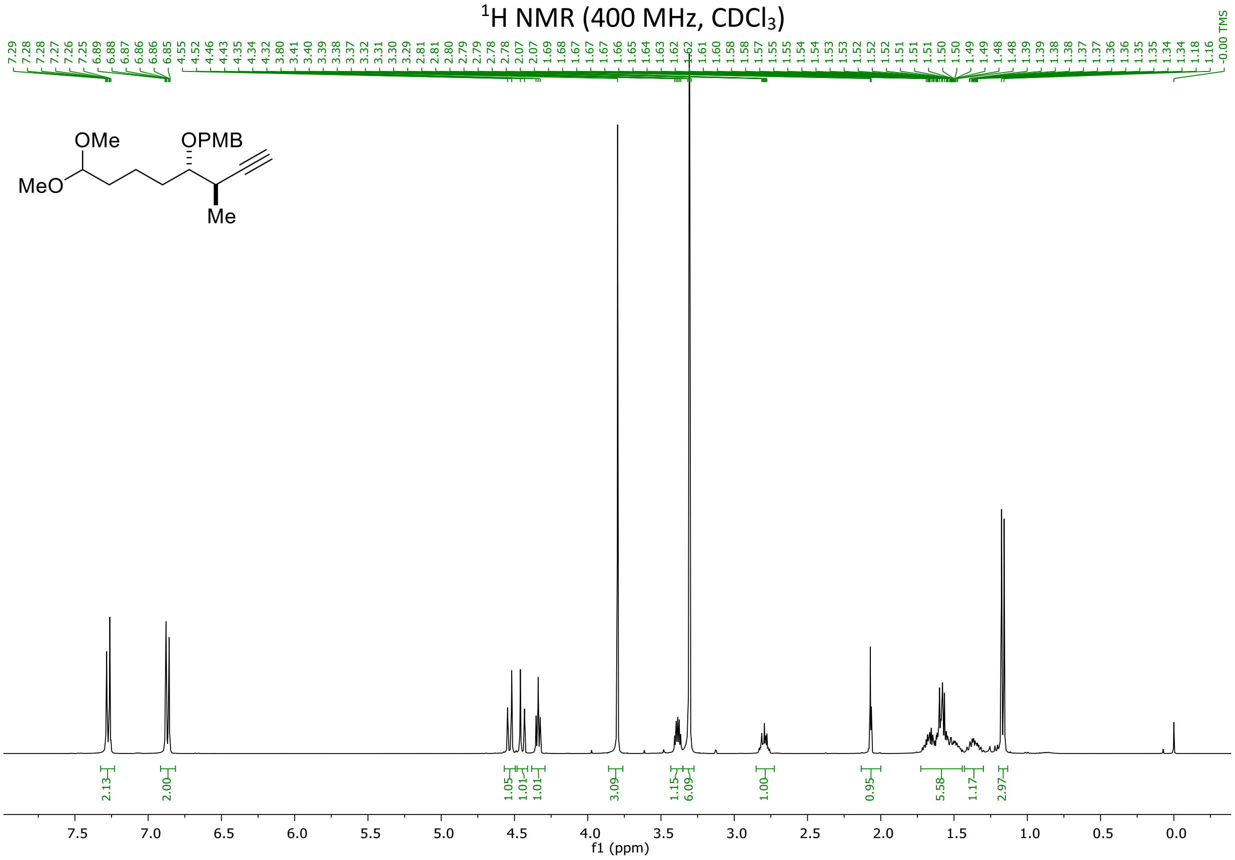

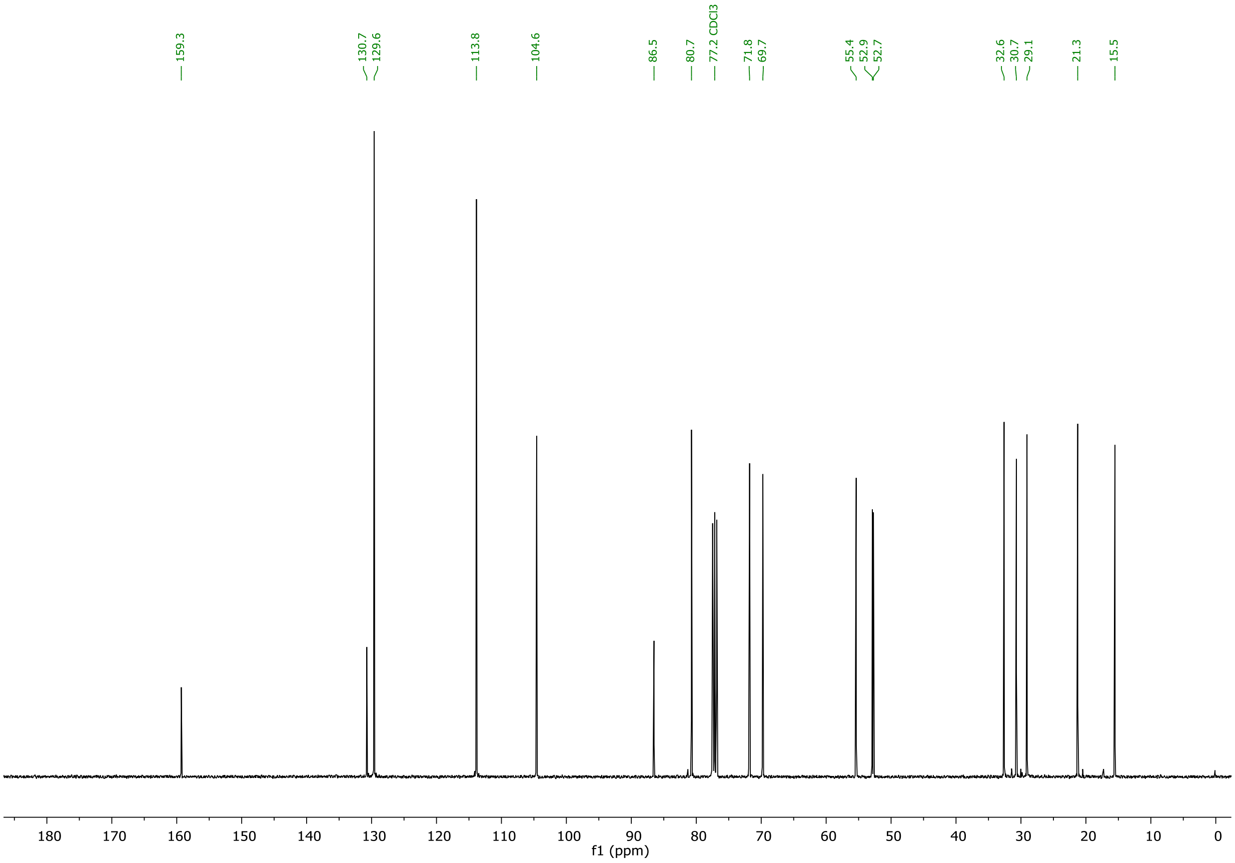

^{13}C NMR (101 MHz, $CDCl_3$)

2-((3*R*,4*S*,*E*)-8,8-Dimethoxy-4-((4-methoxybenzyl)oxy)-3-methyloct-1-en-1-yl)-4,4,5,5-tetramethyl-1,3,2-dioxaborolane (*ent*-6)

^{1}H NMR (400 MHz, $CDCl_3$)

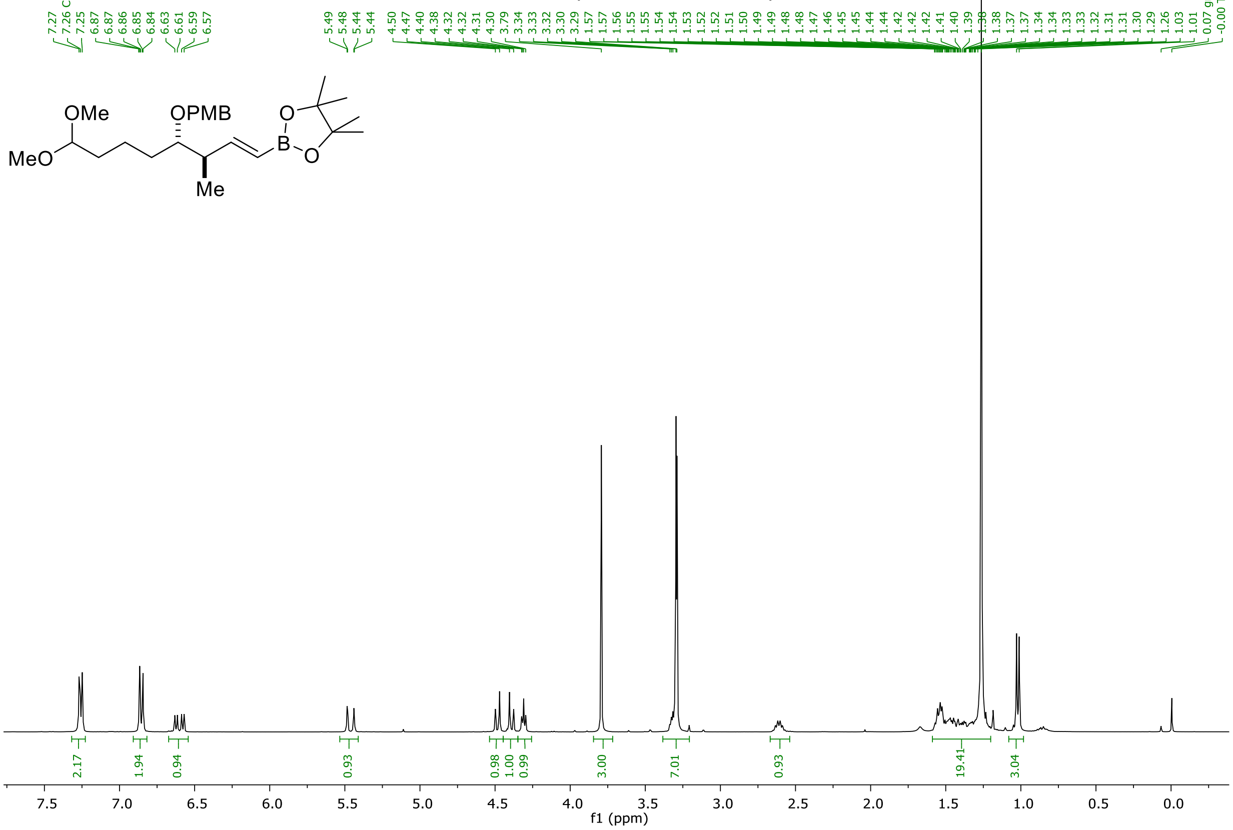

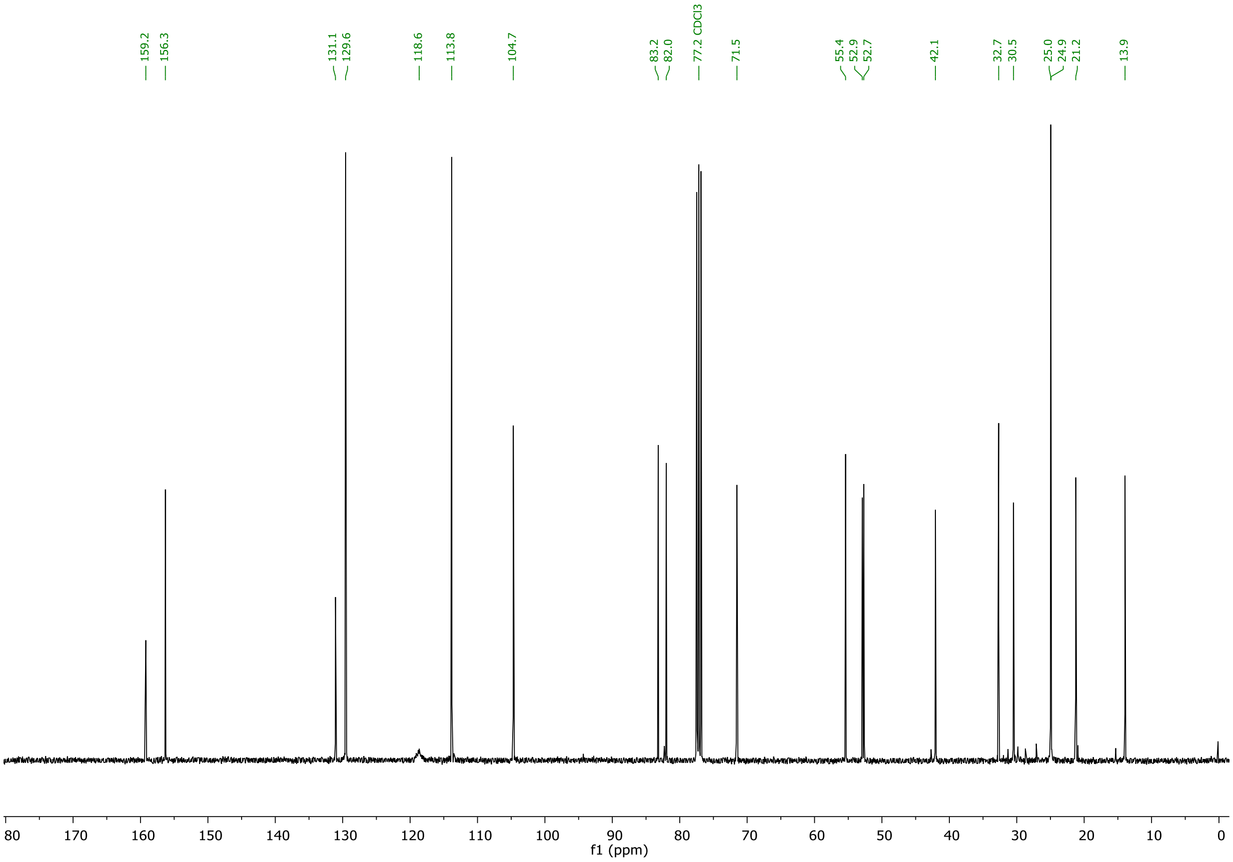

^{13}C NMR (101 MHz, $CDCl_3$)

1-((((5*S*,6*R*,*E*)-1,1-Dimethoxy-6-methylundec-7-en-9-yn-5-yl)oxy)methyl)-4-methoxybenzene (*ent*-7)

^{1}H NMR (400 MHz, $CDCl_3$)

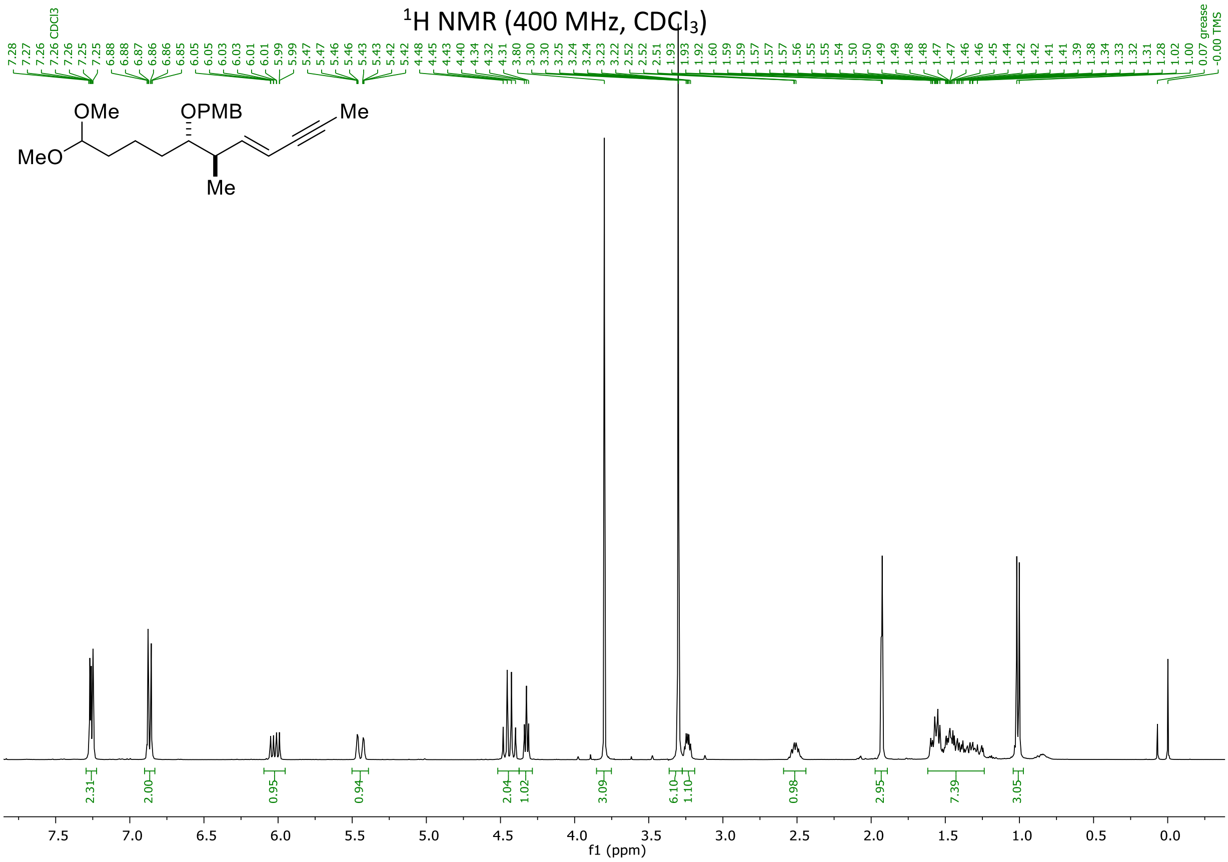

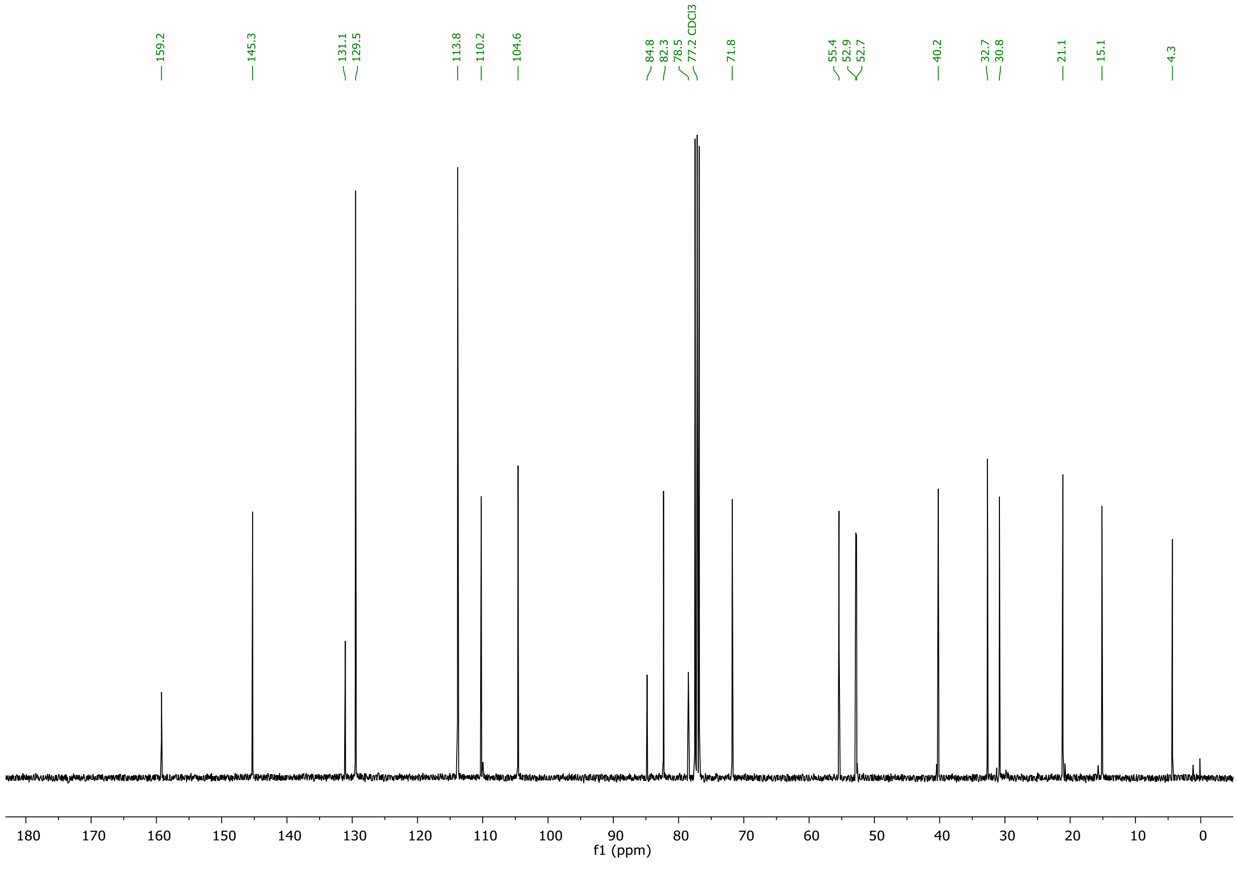

^{13}C NMR (101 MHz, $CDCl_3$)

(4*R*,5*R*,6*R*,7*S*)-11,11-Dimethoxy-7-((4-methoxybenzyl)oxy)-6-methylundec-2-yne-4,5-diol (*ent*-8)

^{1}H NMR (400 MHz, $CDCl_3$)

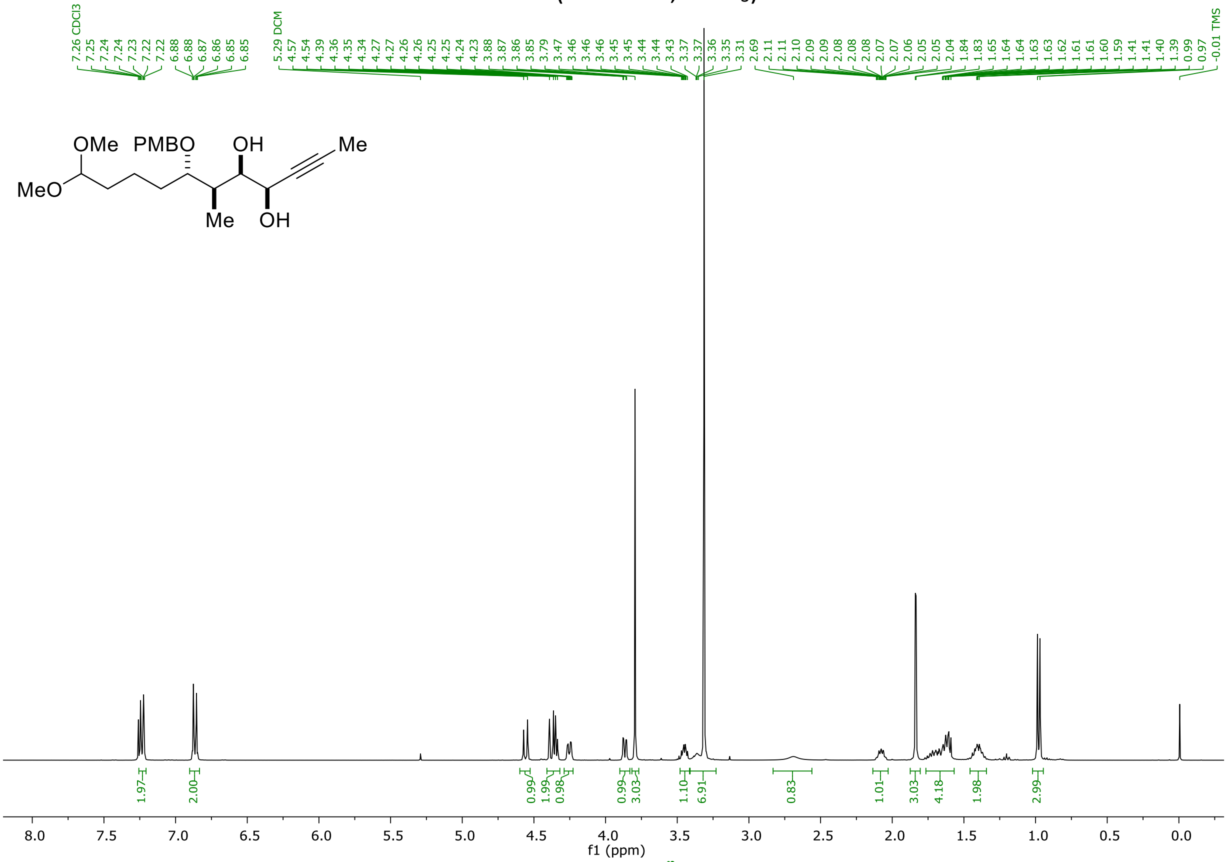

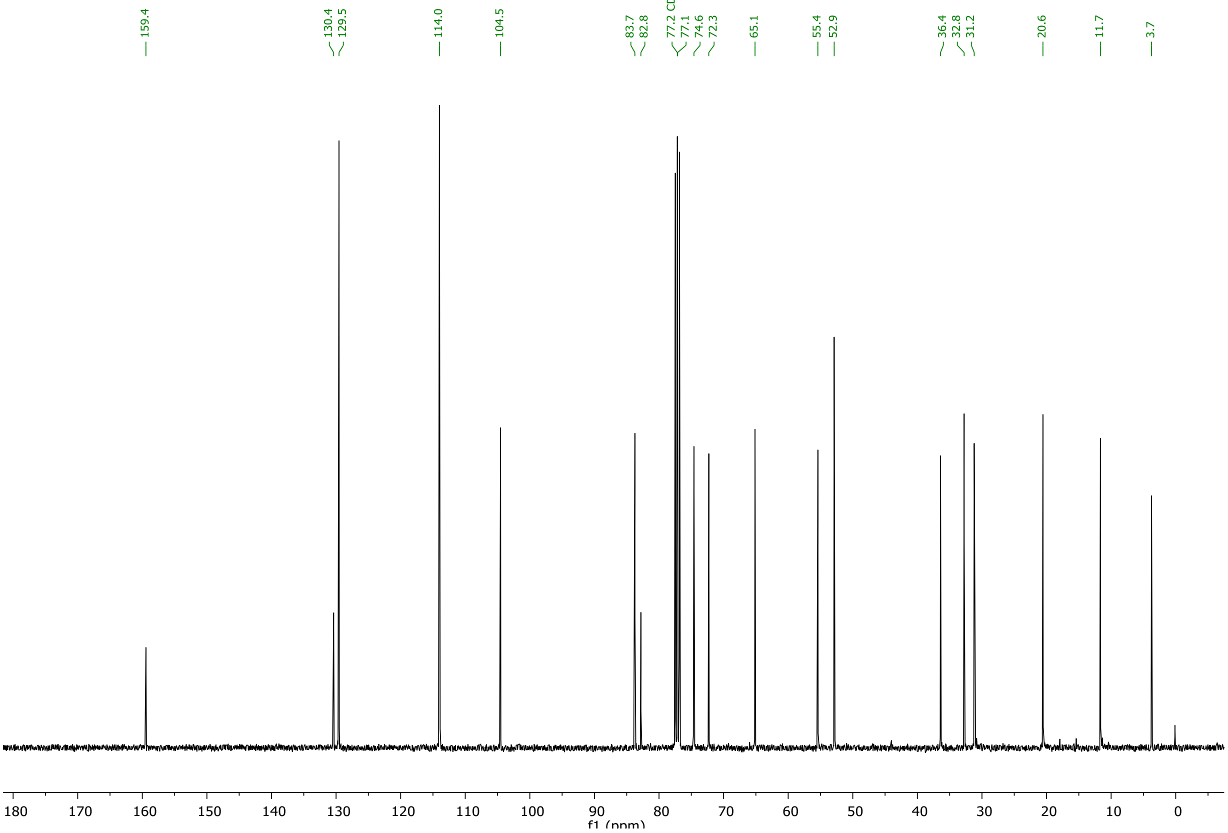

^{13}C NMR (101 MHz, $CDCl_3$)

(5*S*,6*S*,7*R*,8*R*)-5-((4-Methoxybenzyl)oxy)-6-methyl-7,8-bis((triethylsilyl)oxy)undec-9-ynal (*ent*-9)

^{1}H NMR (400 MHz, $CDCl_3$)

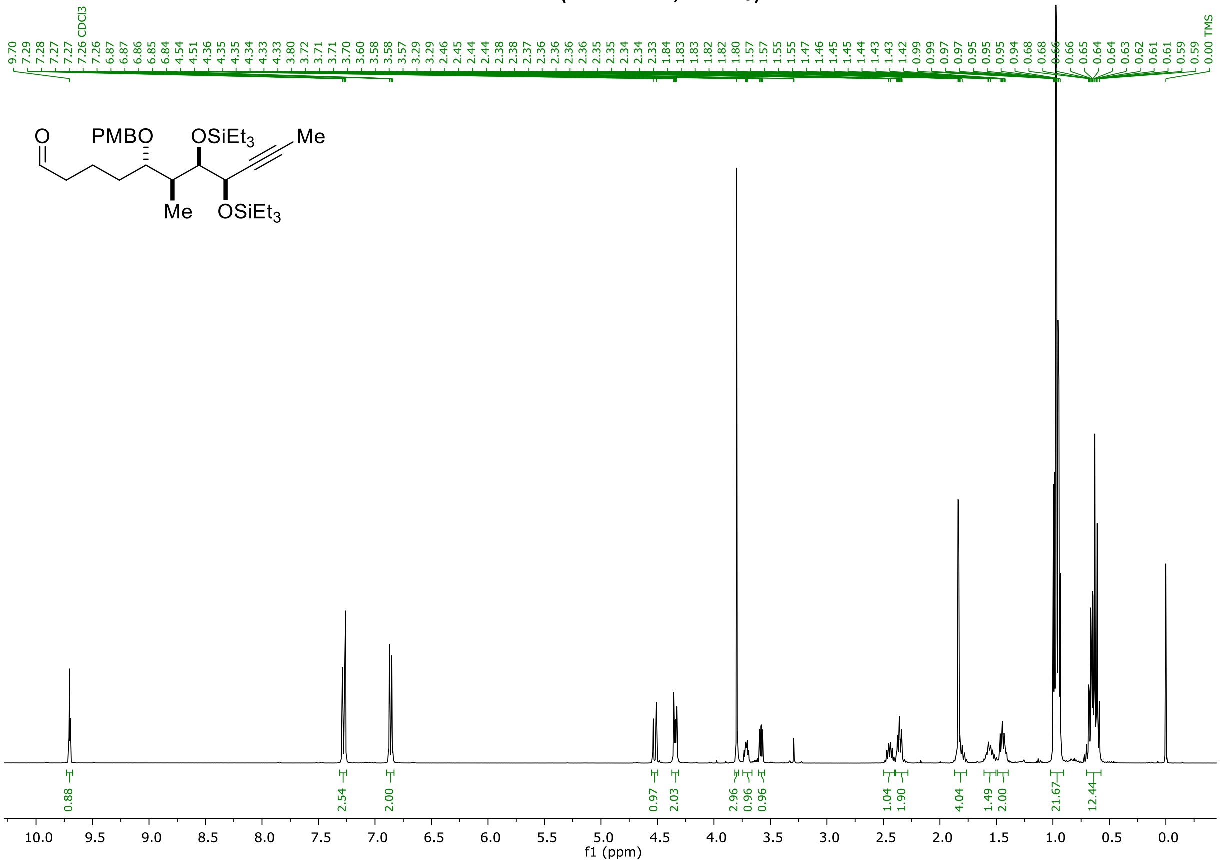

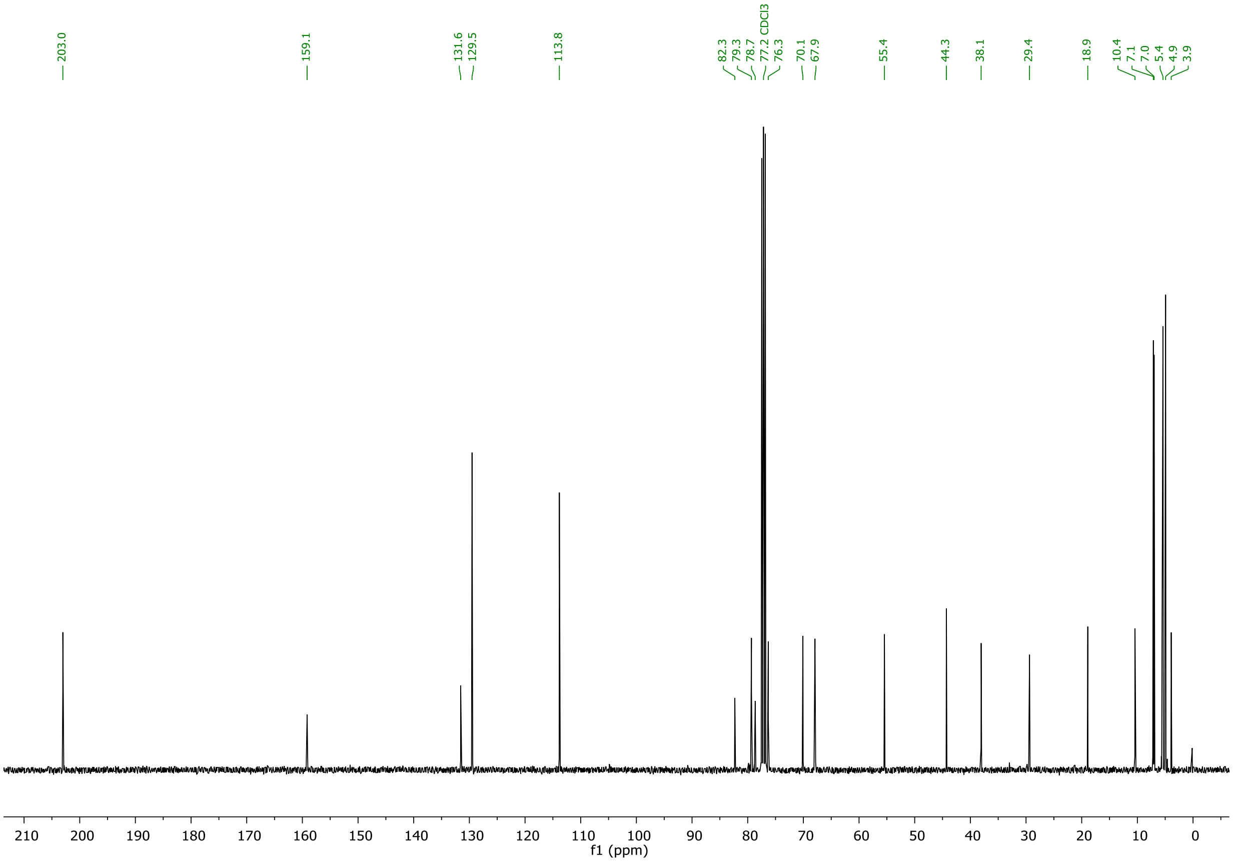

^{13}C NMR (101 MHz, $CDCl_3$)

β-Lactone 12

^{1}H NMR (400 MHz, $CDCl_3$)

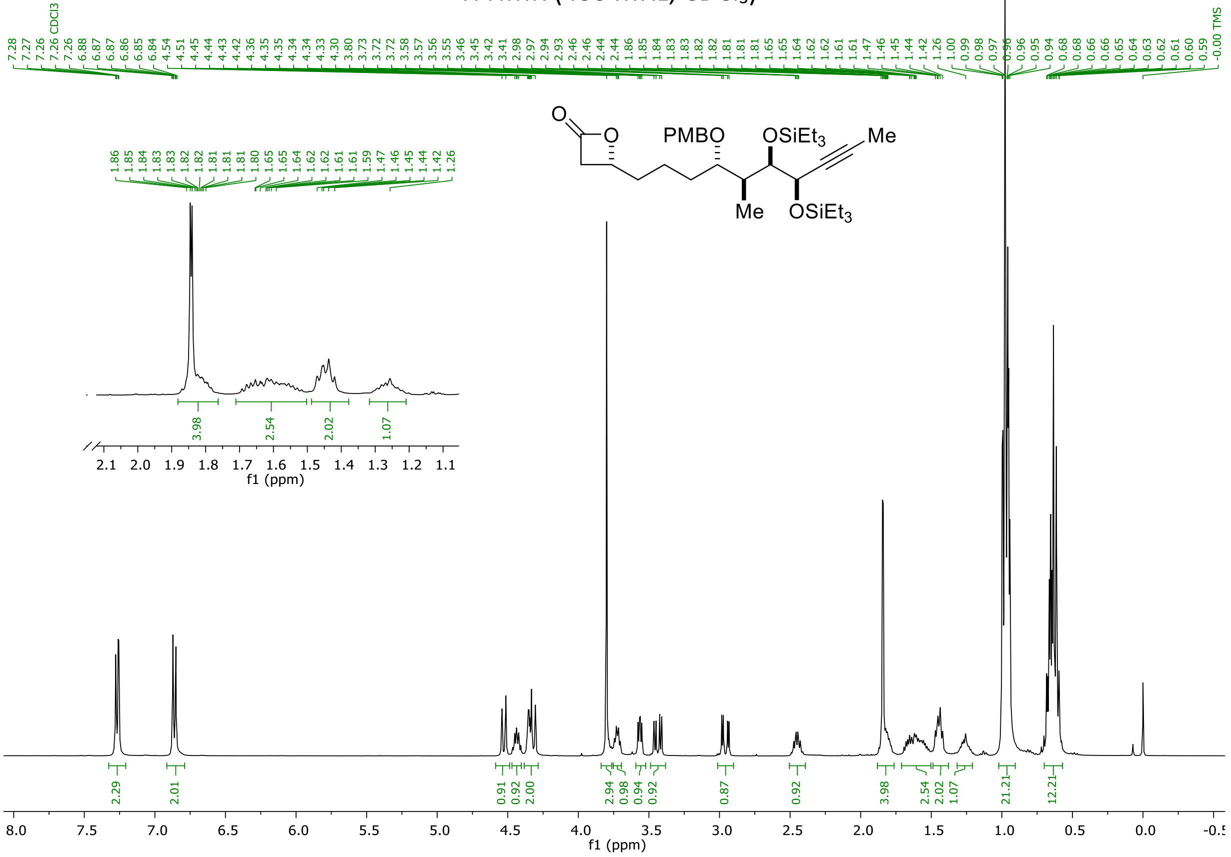

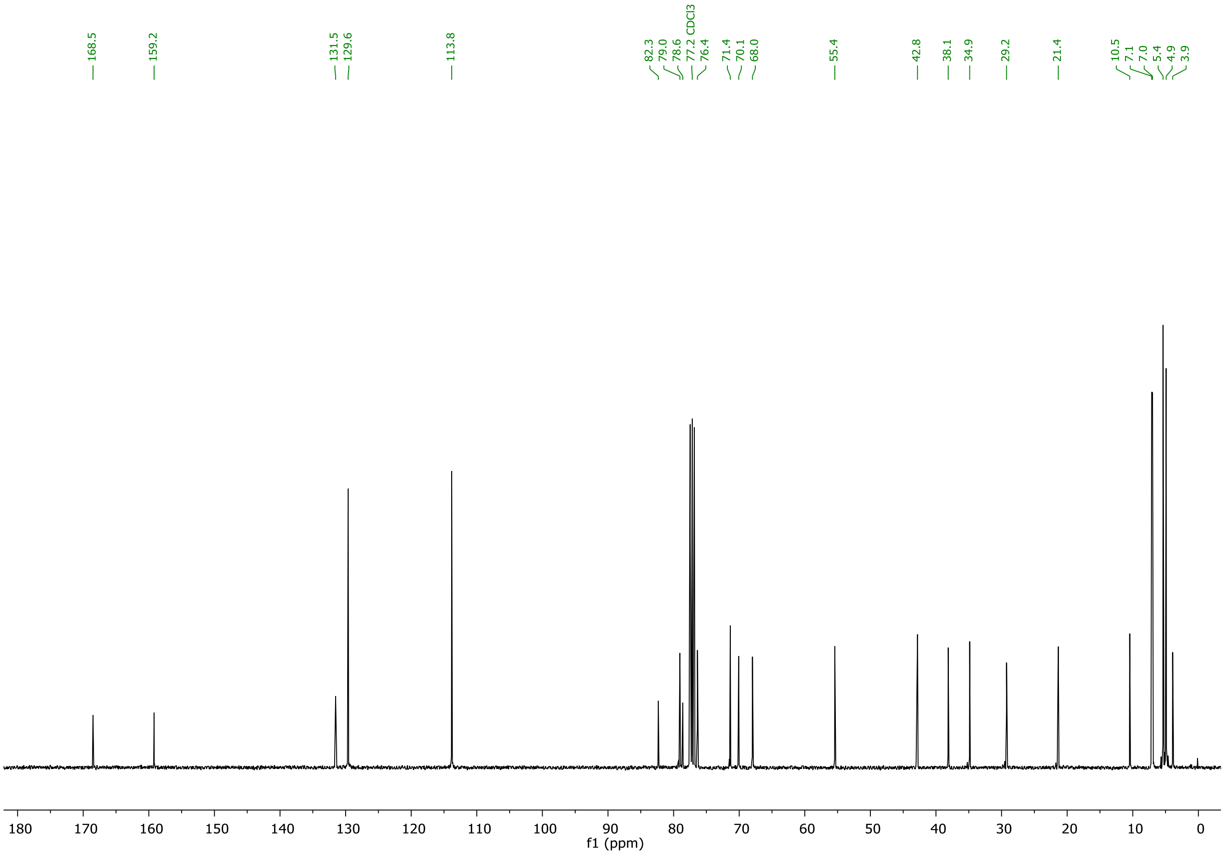

^{13}C NMR (101 MHz, $CDCl_3$)

β-Lactone 10

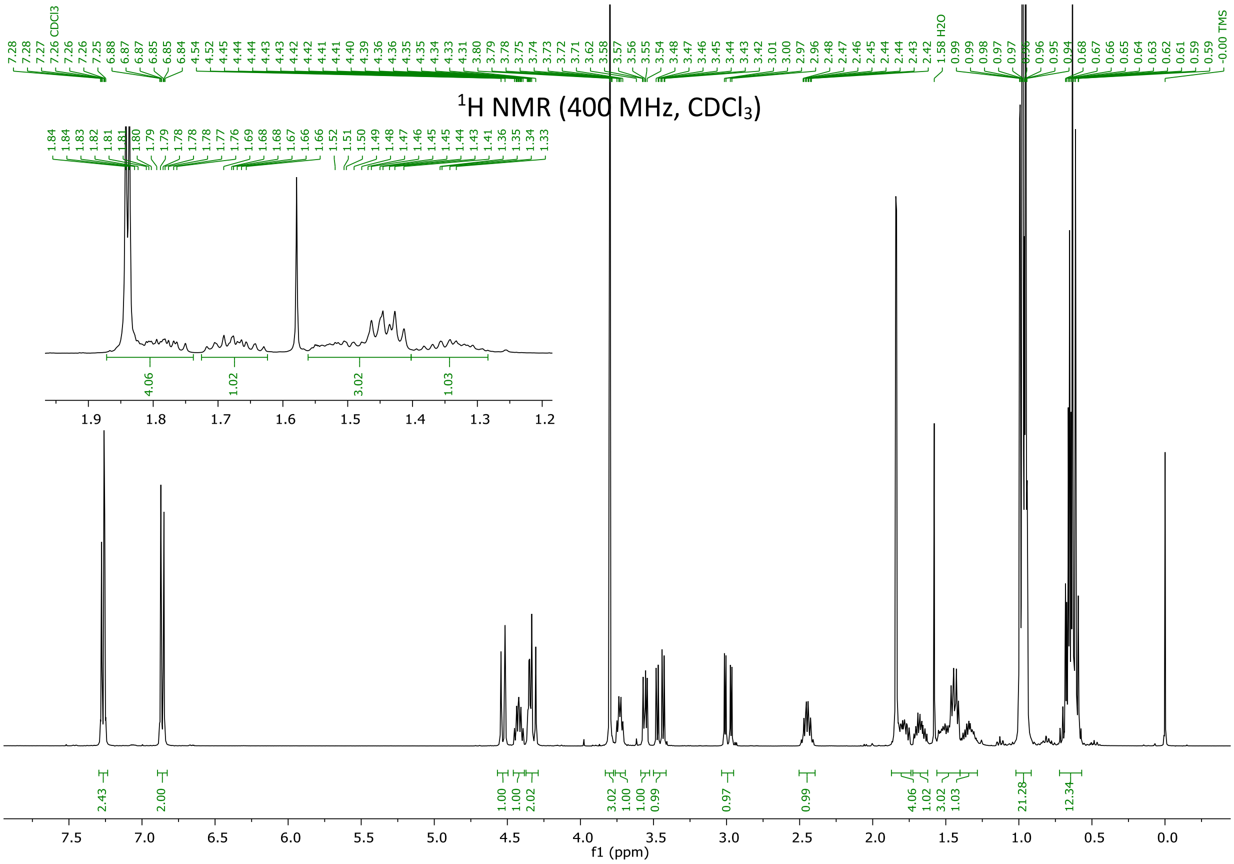

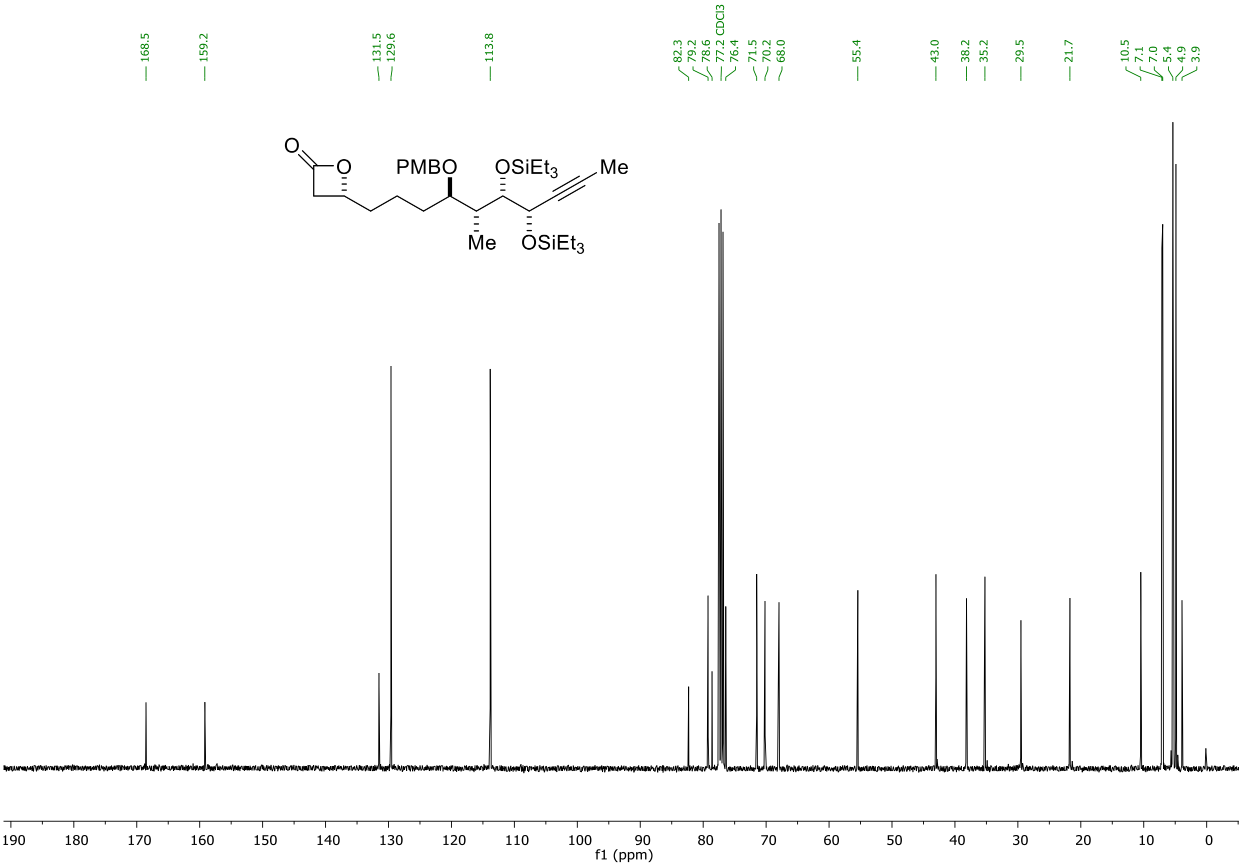

^{13}C NMR (101 MHz, $CDCl_3$)

β,γ-Enone 13

^{1}H NMR (400 MHz, $CDCl_3$)

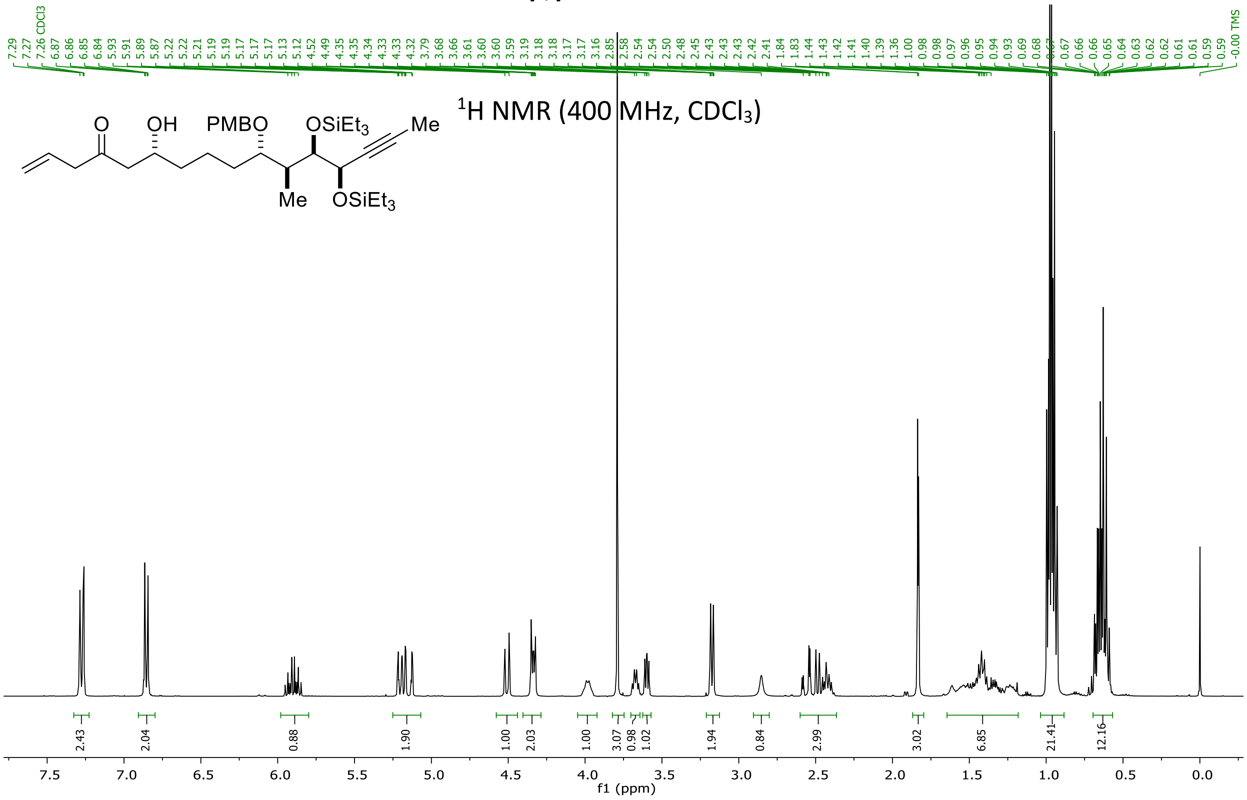

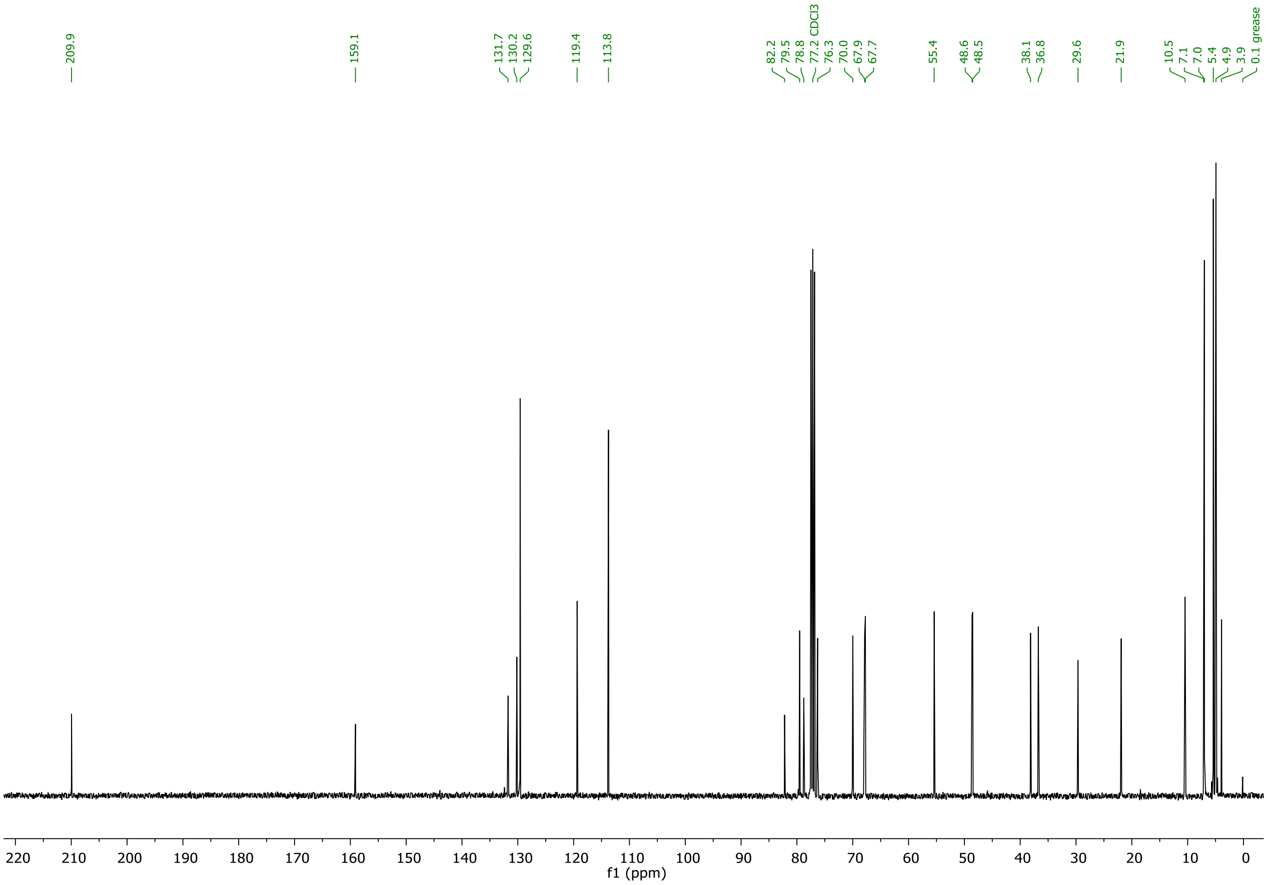

^{13}C NMR (101 MHz, $CDCl_3$)

Mosher Ester Analysis: (*R*)-S4

^{1}H NMR (400 MHz, $CDCl_3$)

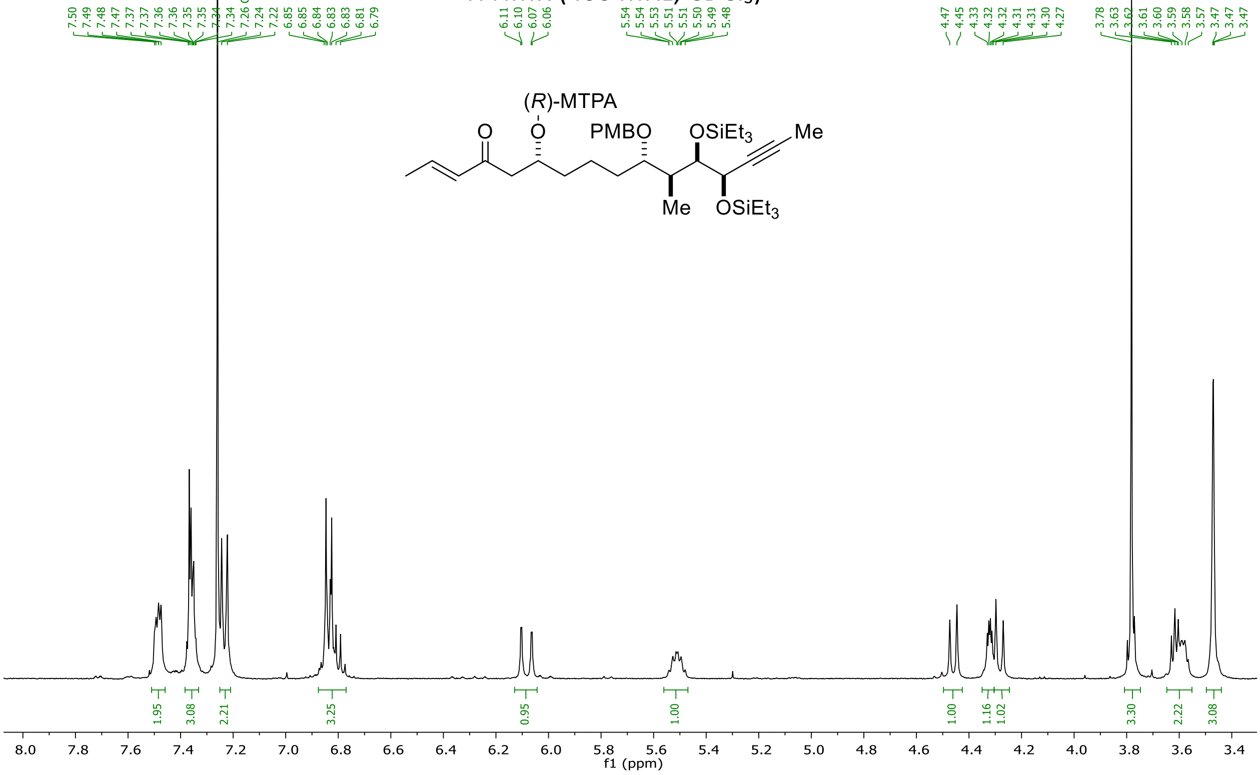

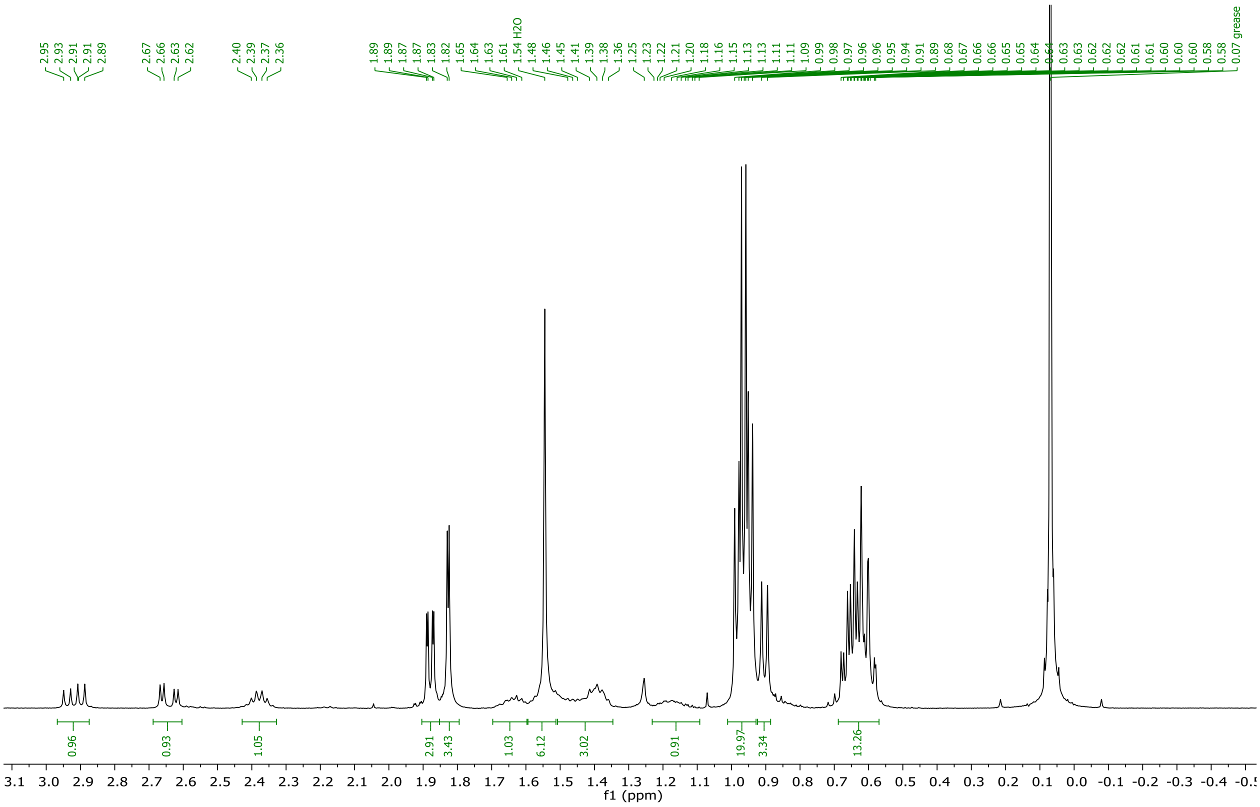

Mosher Ester Analysis: (*S*)-S4

^{1}H NMR (400 MHz, $CDCl_3$)

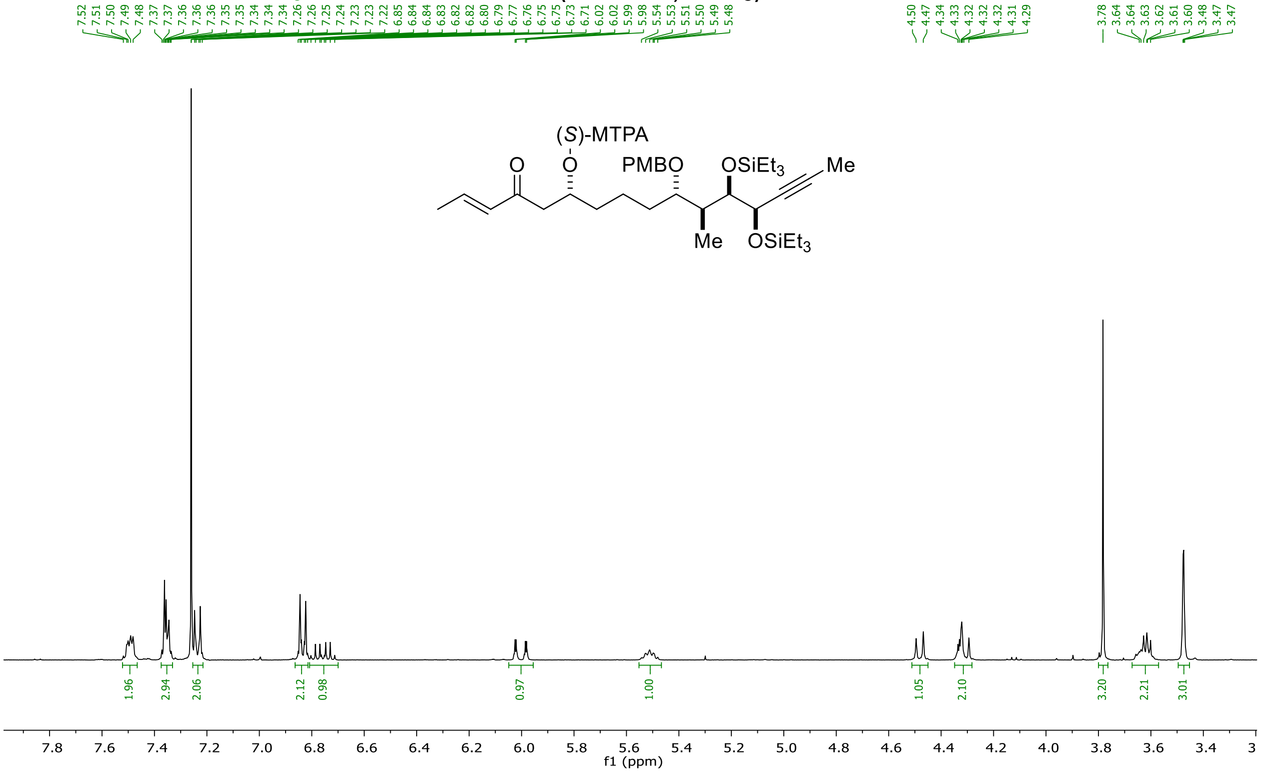

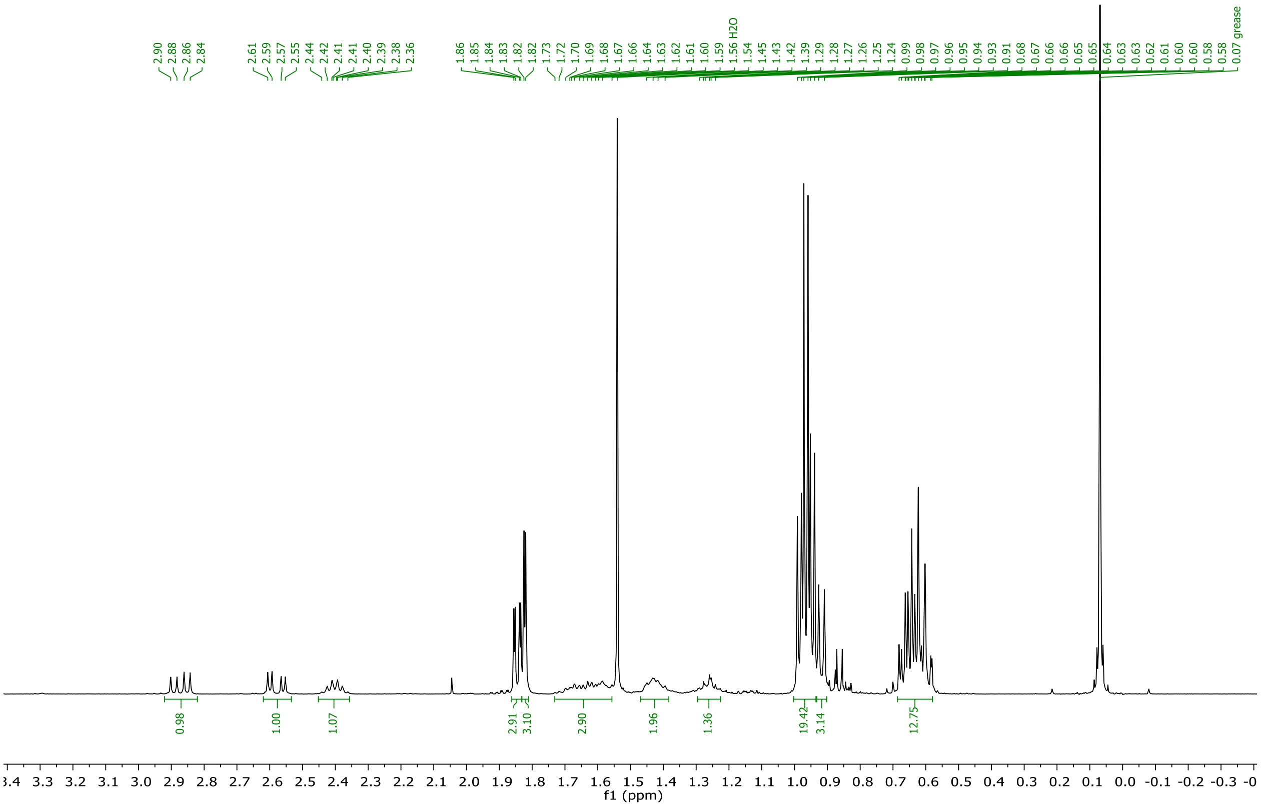

β,γ-Enone 11

^{1}H NMR (400 MHz, CDCl$_3$)

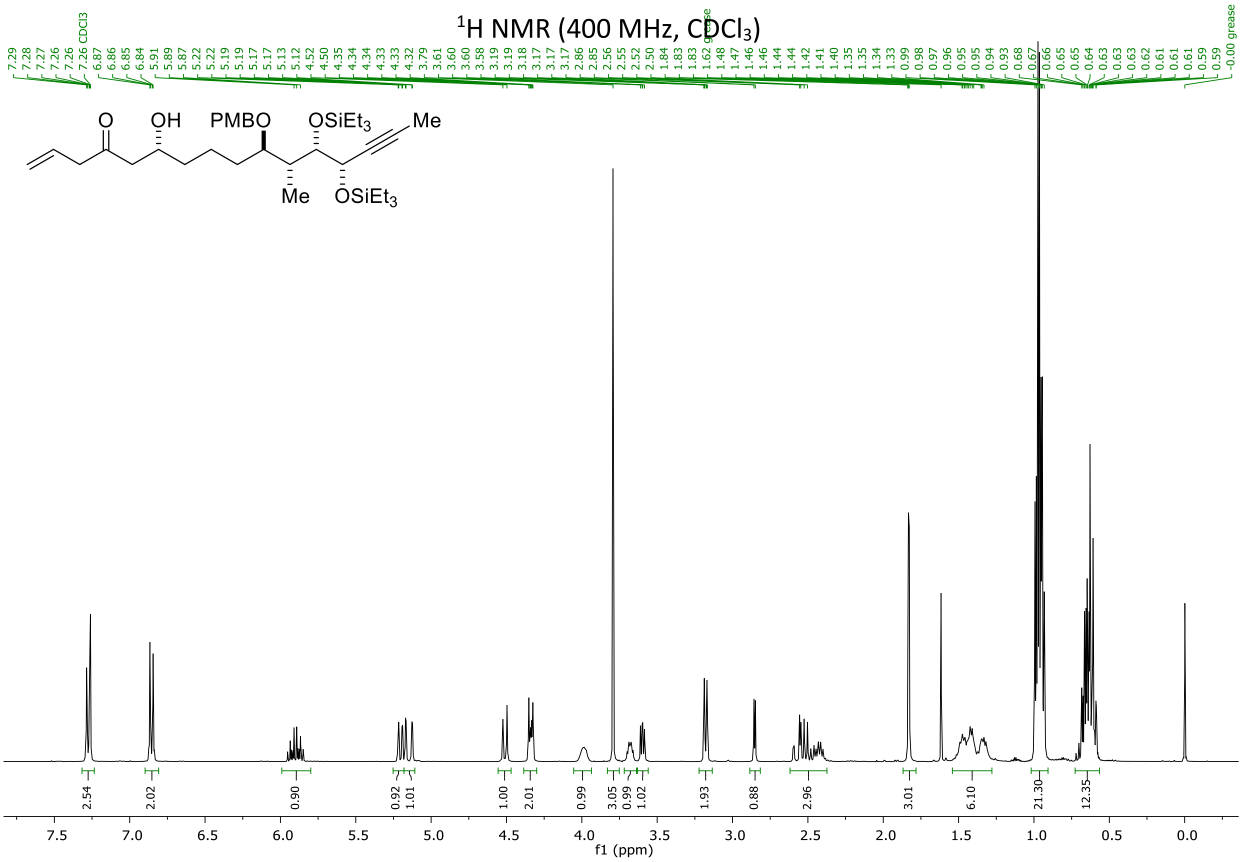

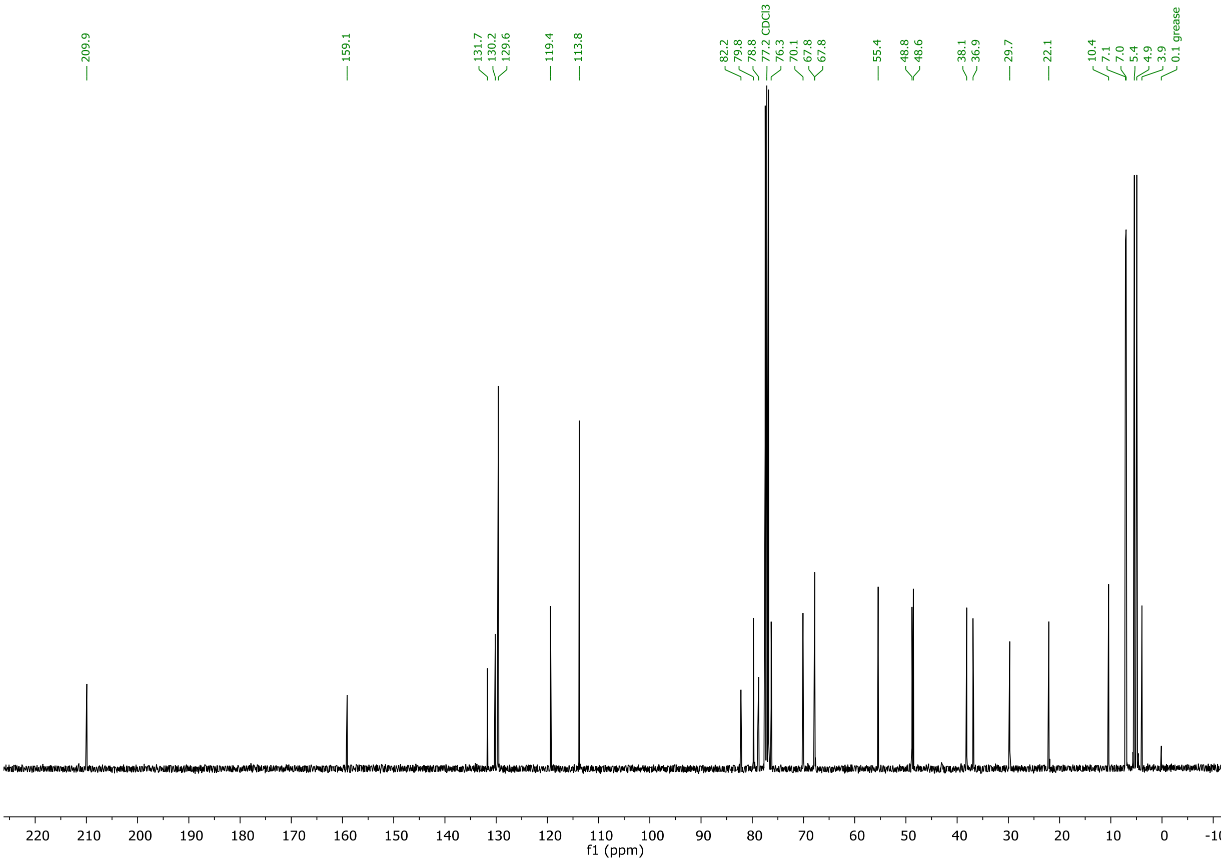

^{13}C NMR (101 MHz, CDCl$_3$)

α,β-Unsaturated Ketone S3

^{1}H NMR (600 MHz, $CDCl_3$)

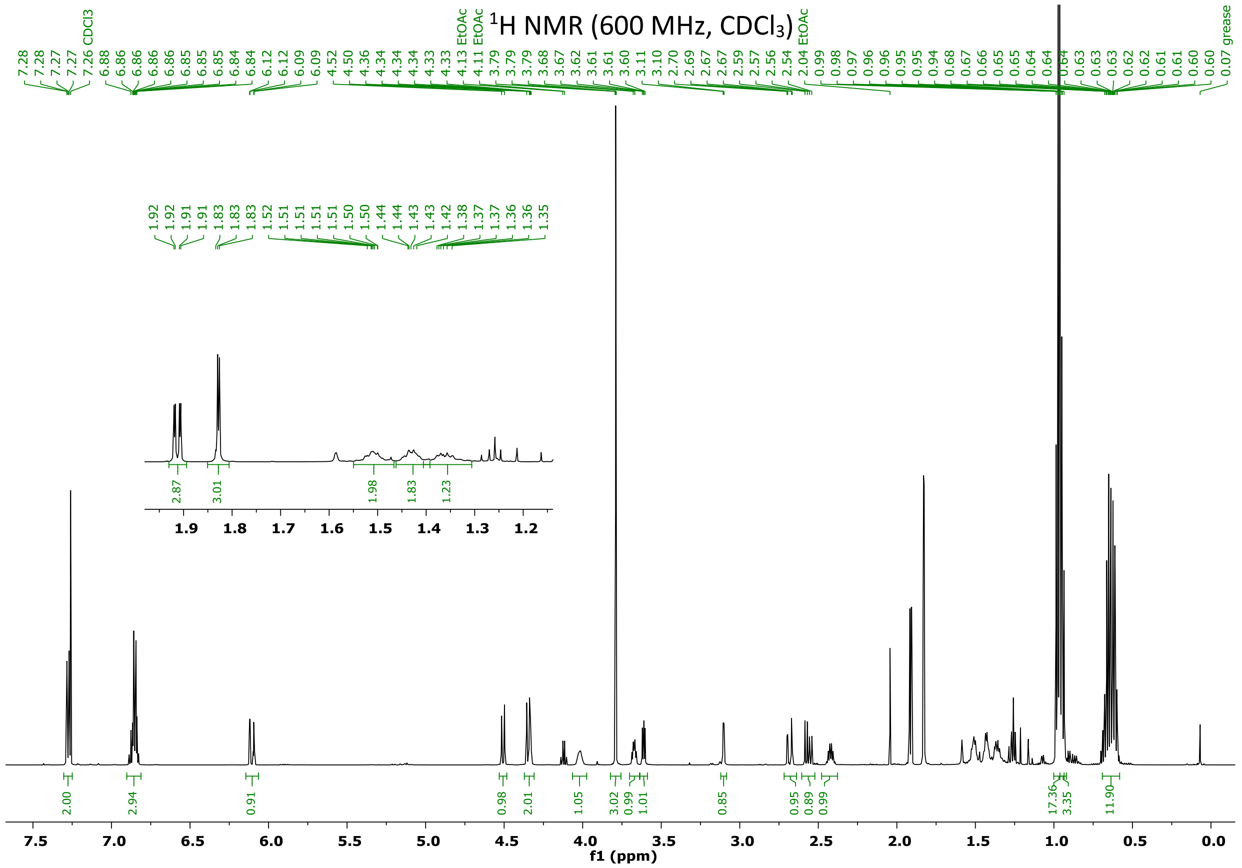

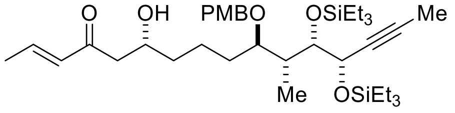

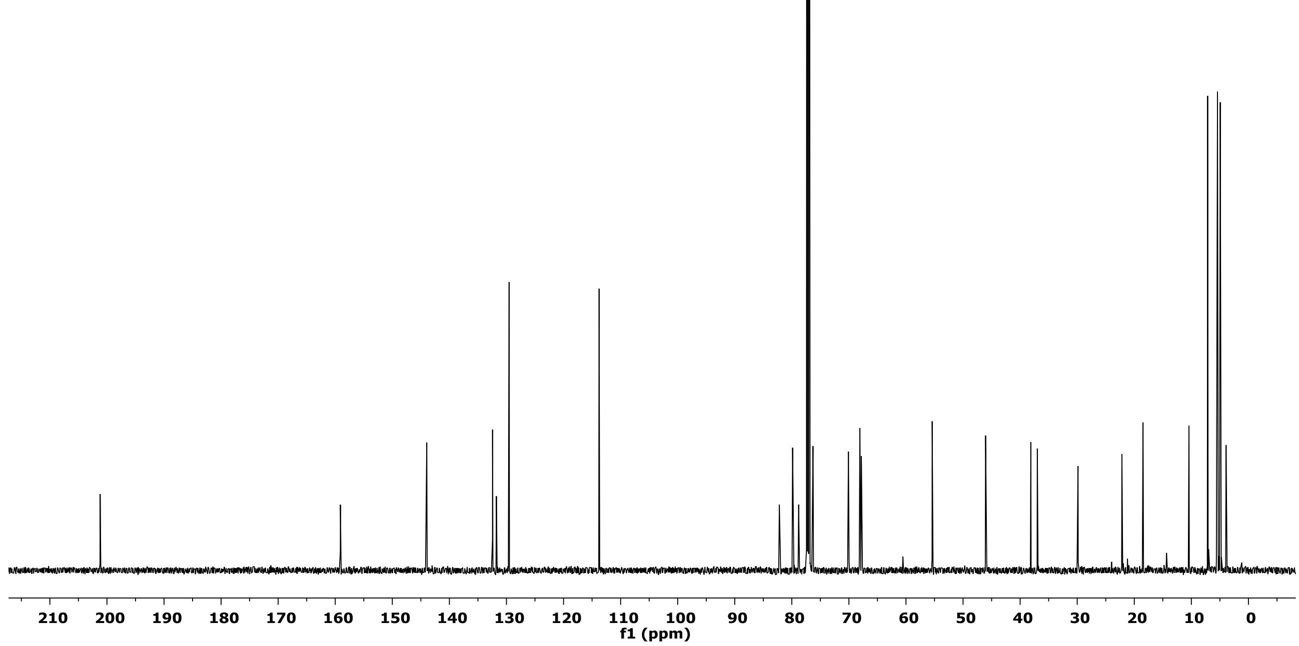

^{13}C NMR (151 MHz, $CDCl_3$)

Weinreb Amide S5

^{1}H NMR (400 MHz, $CDCl_3$)

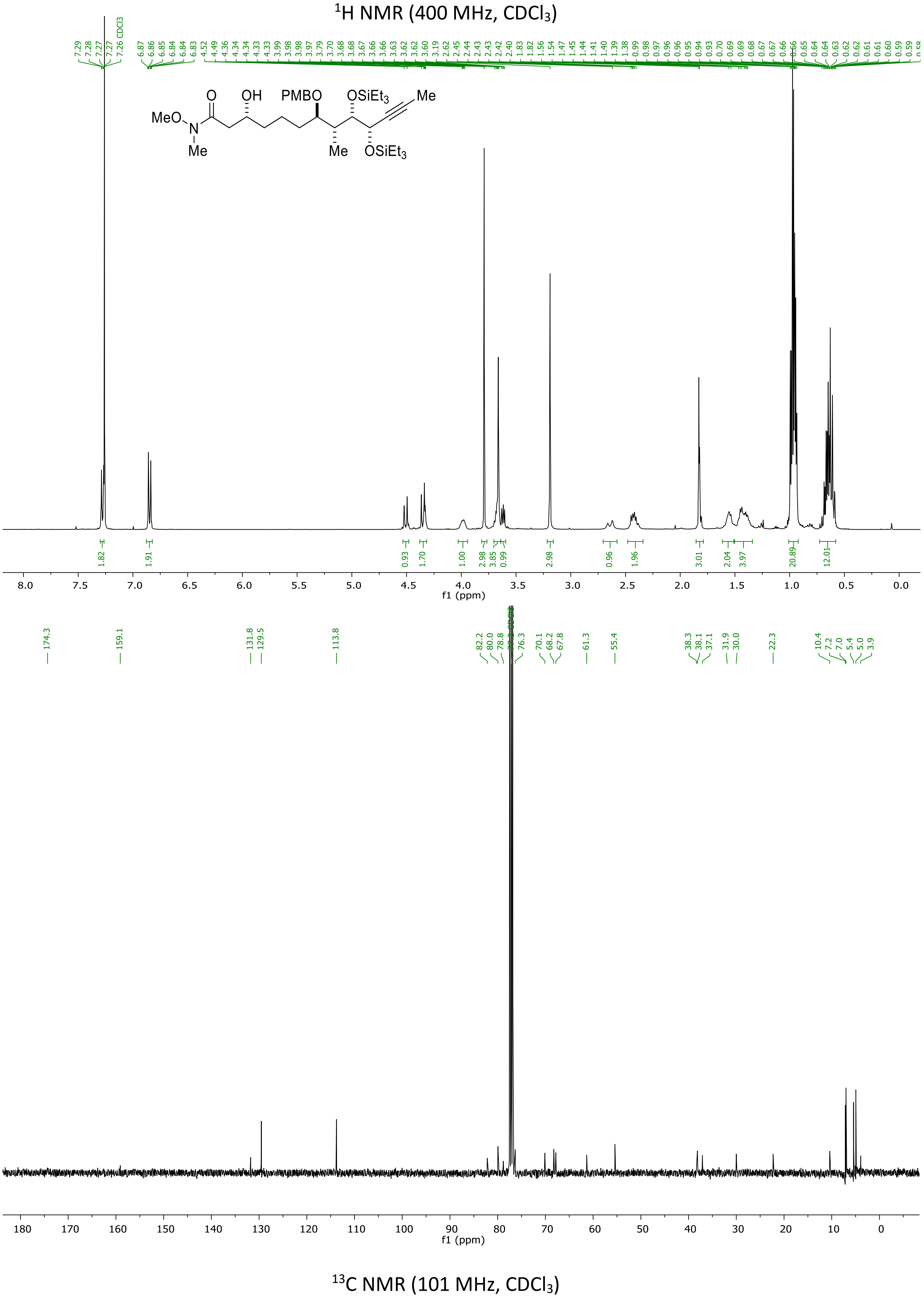

^{13}C NMR (101 MHz, $CDCl_3$)

Mosher Ester Analysis of Weinreb Amide: (*R*)-S6

^{1}H NMR (400 MHz, $CDCl_3$)

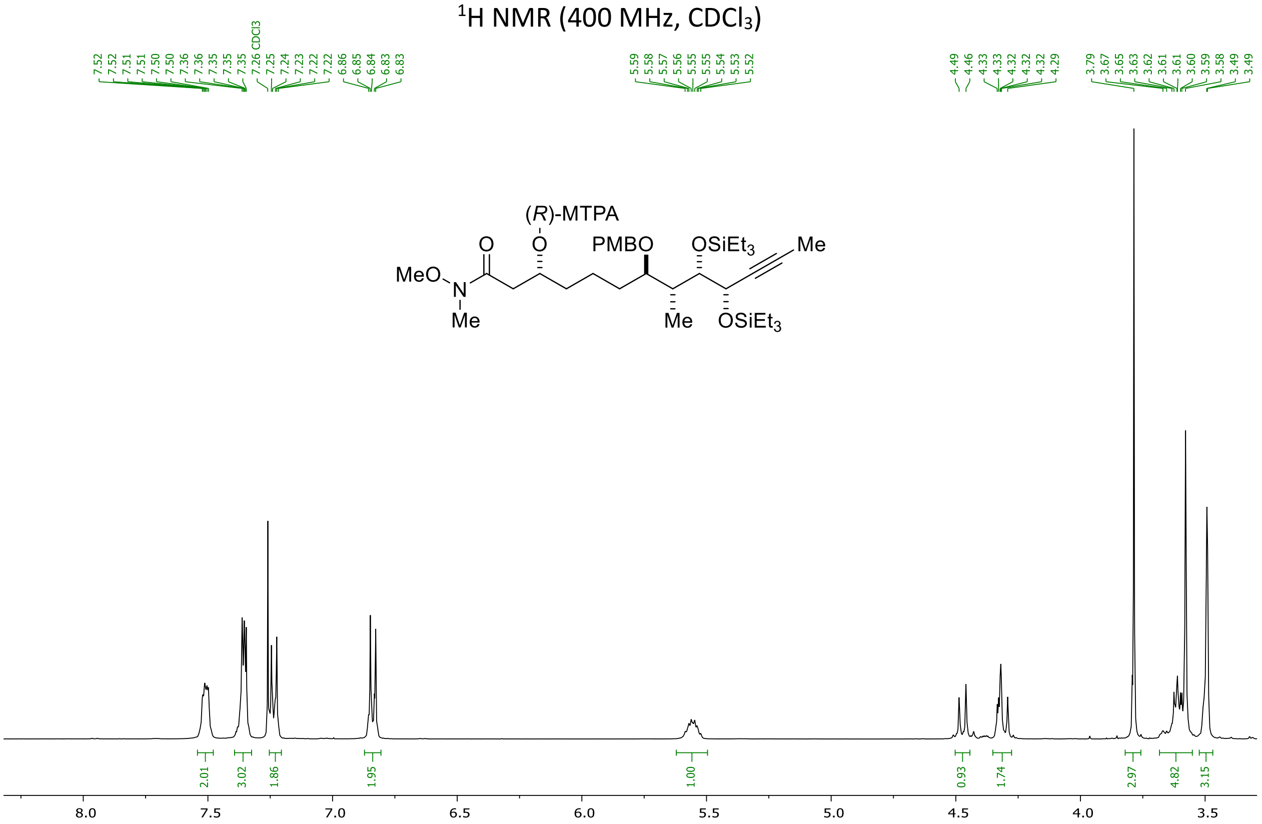

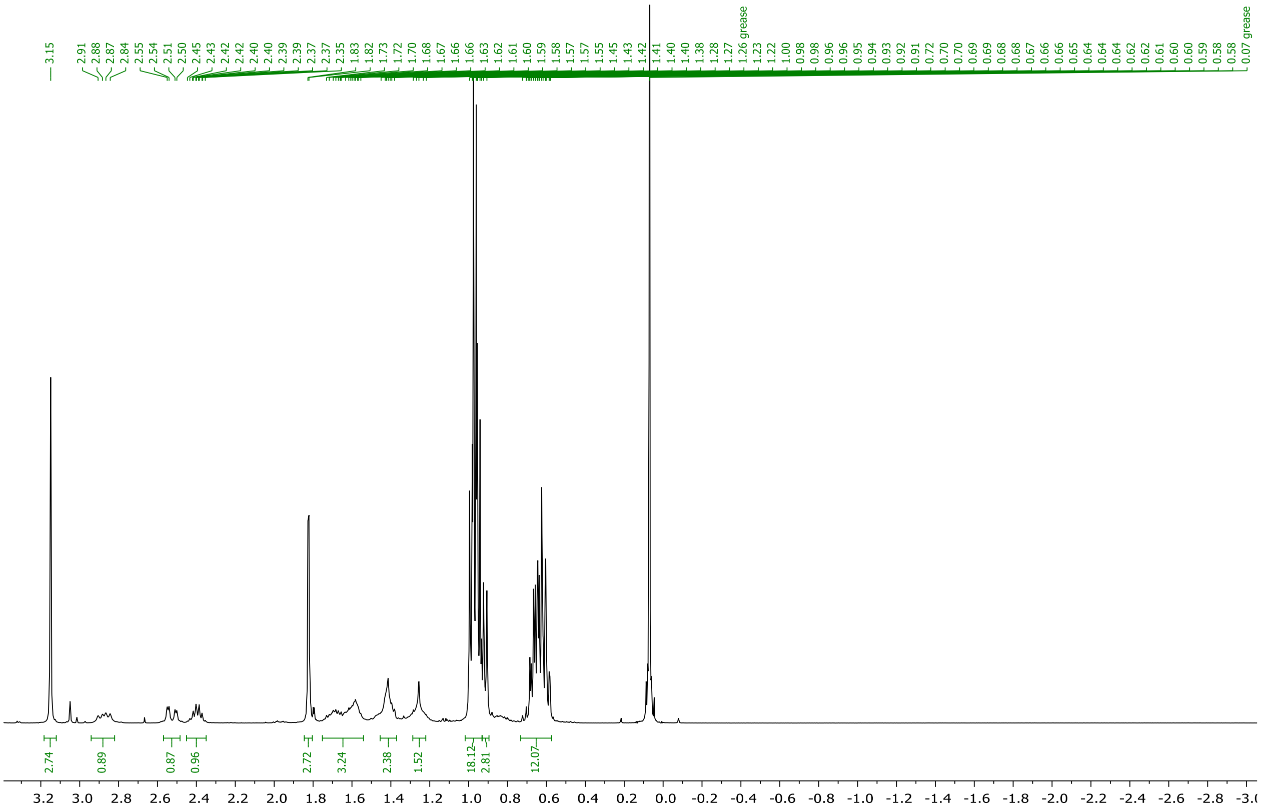

Mosher Ester Analysis of Weinreb Amide: (*S*)-S6

^{1}H NMR (400 MHz, $CDCl_3$)

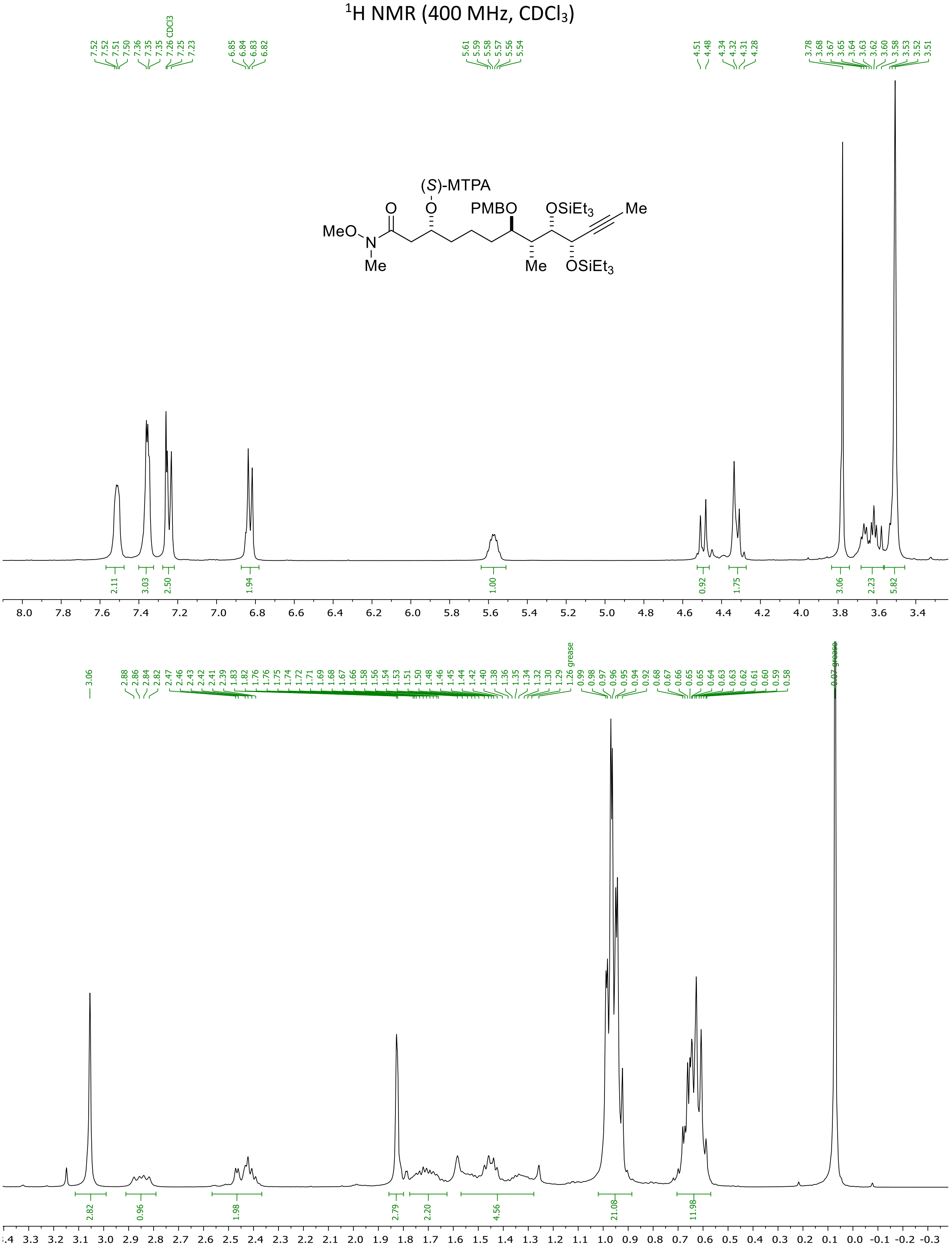

Diyne 23

^{1}H NMR (400 MHz, $CDCl_3$)

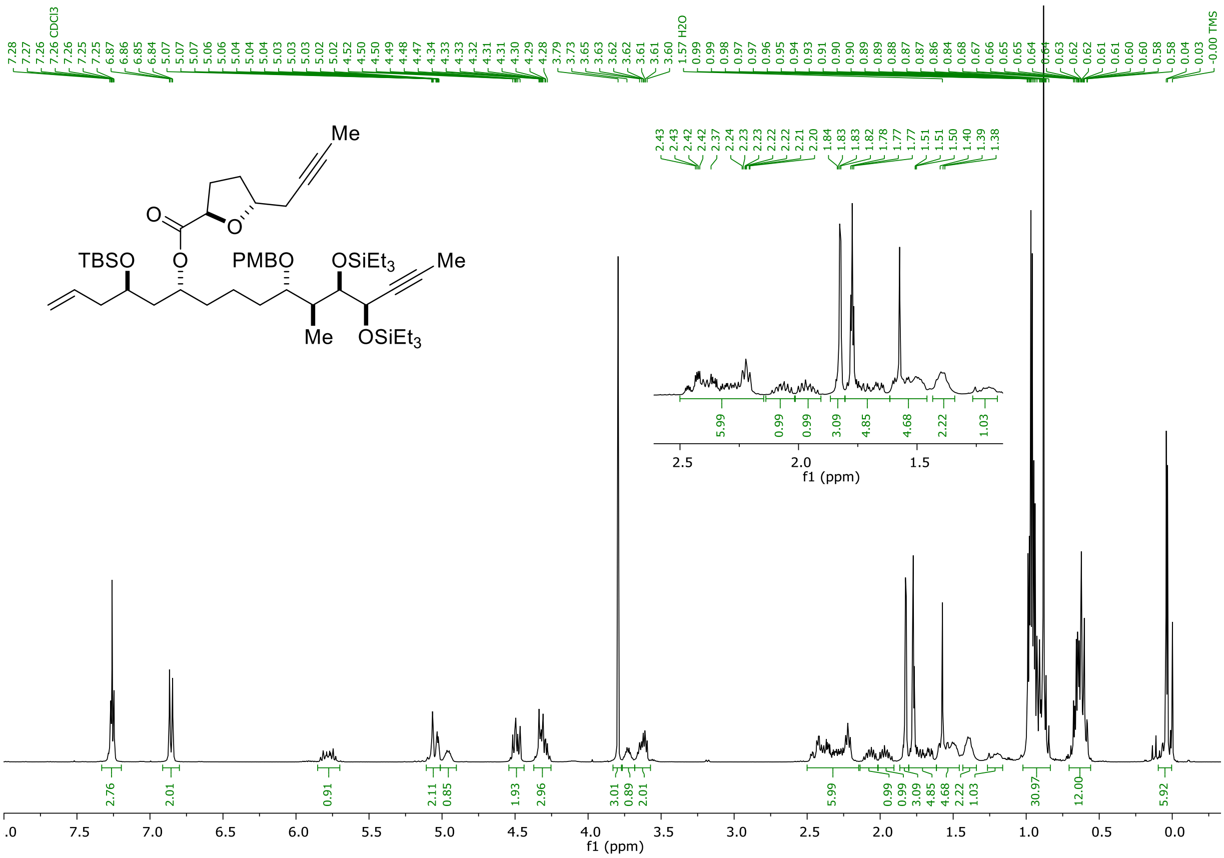

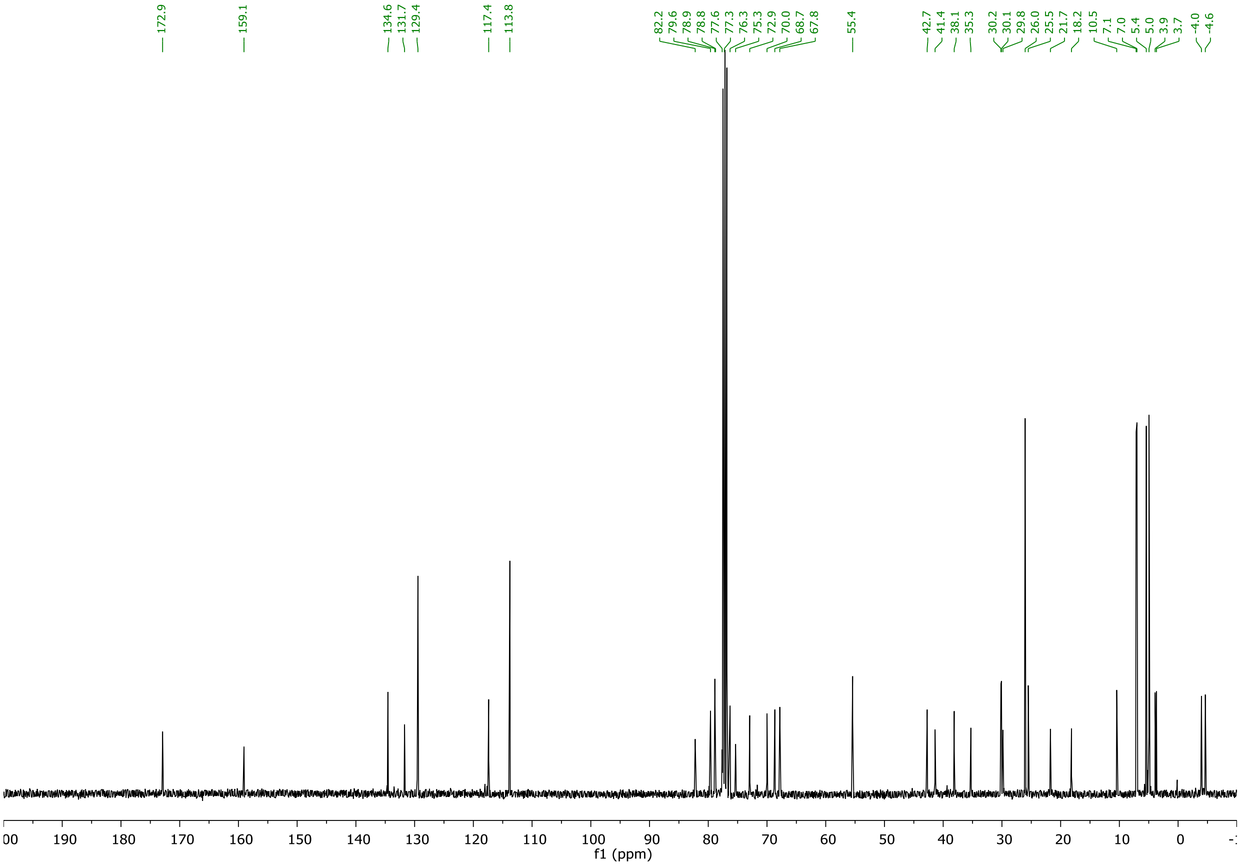

^{13}C NMR (101 MHz, $CDCl_3$)

Regioisomer 24

^{1}H NMR (600 MHz, $CDCl_3$)

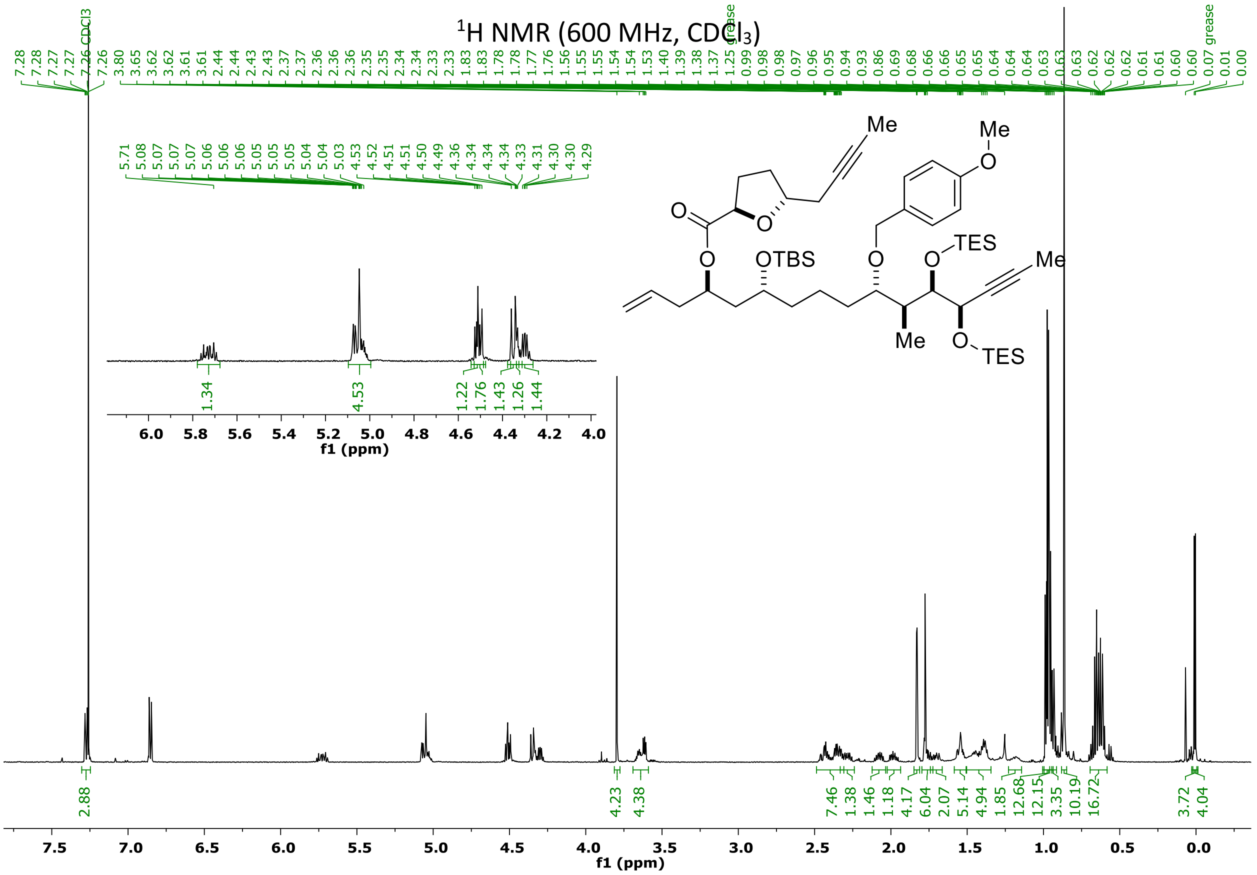

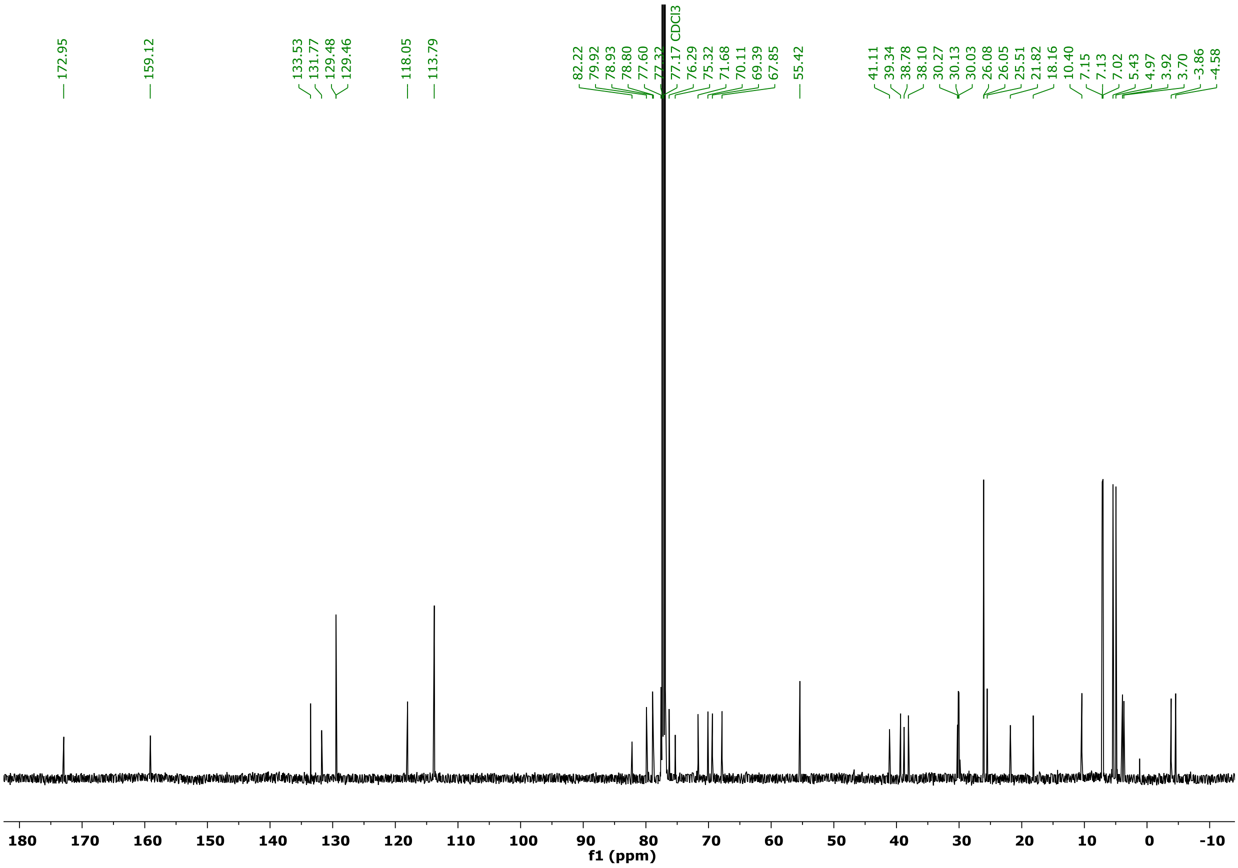

^{13}C NMR (151 MHz, $CDCl_3$)

Diyne 19

^{1}H NMR (400 MHz, $CDCl_3$)

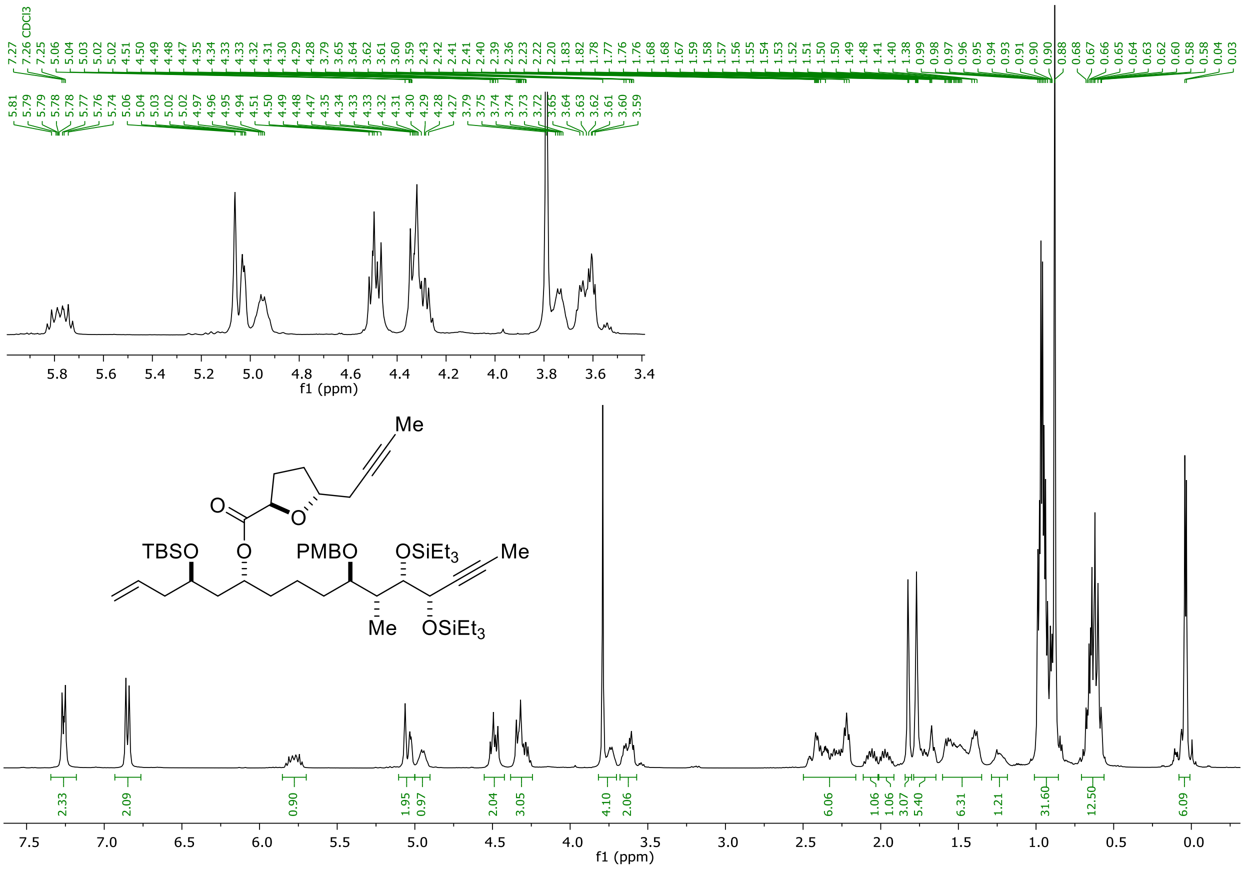

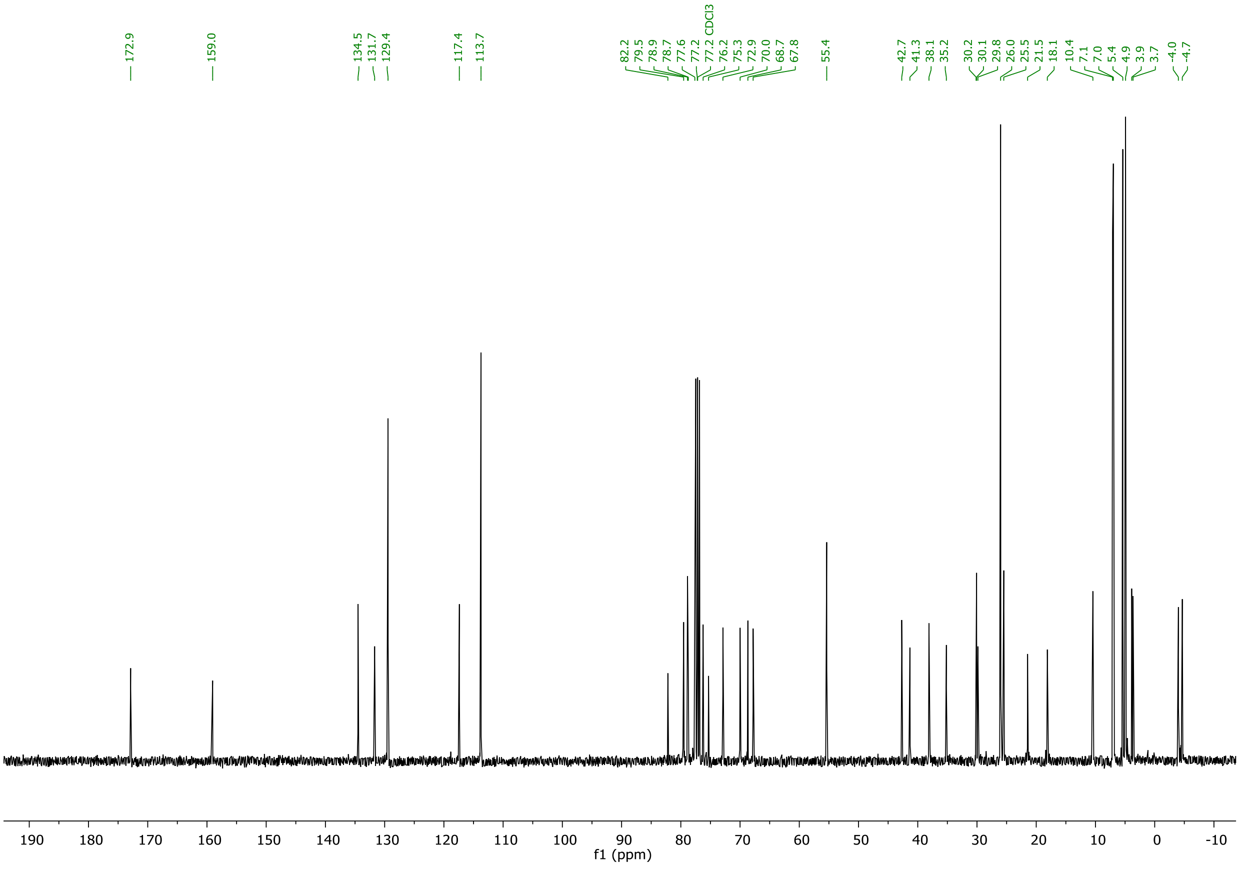

^{13}C NMR (101 MHz, $CDCl_3$)

Regioisomer 20

^{1}H NMR (600 MHz, $CDCl_3$)

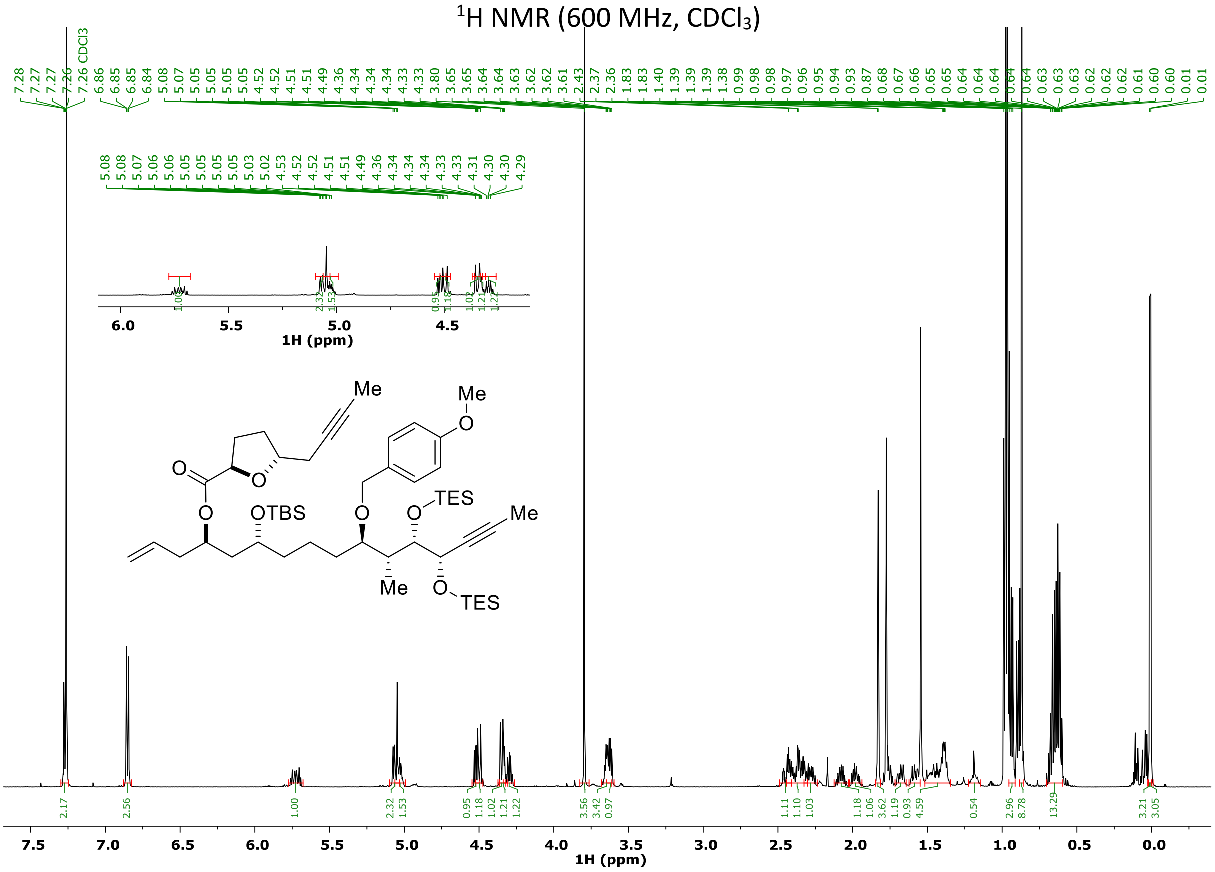

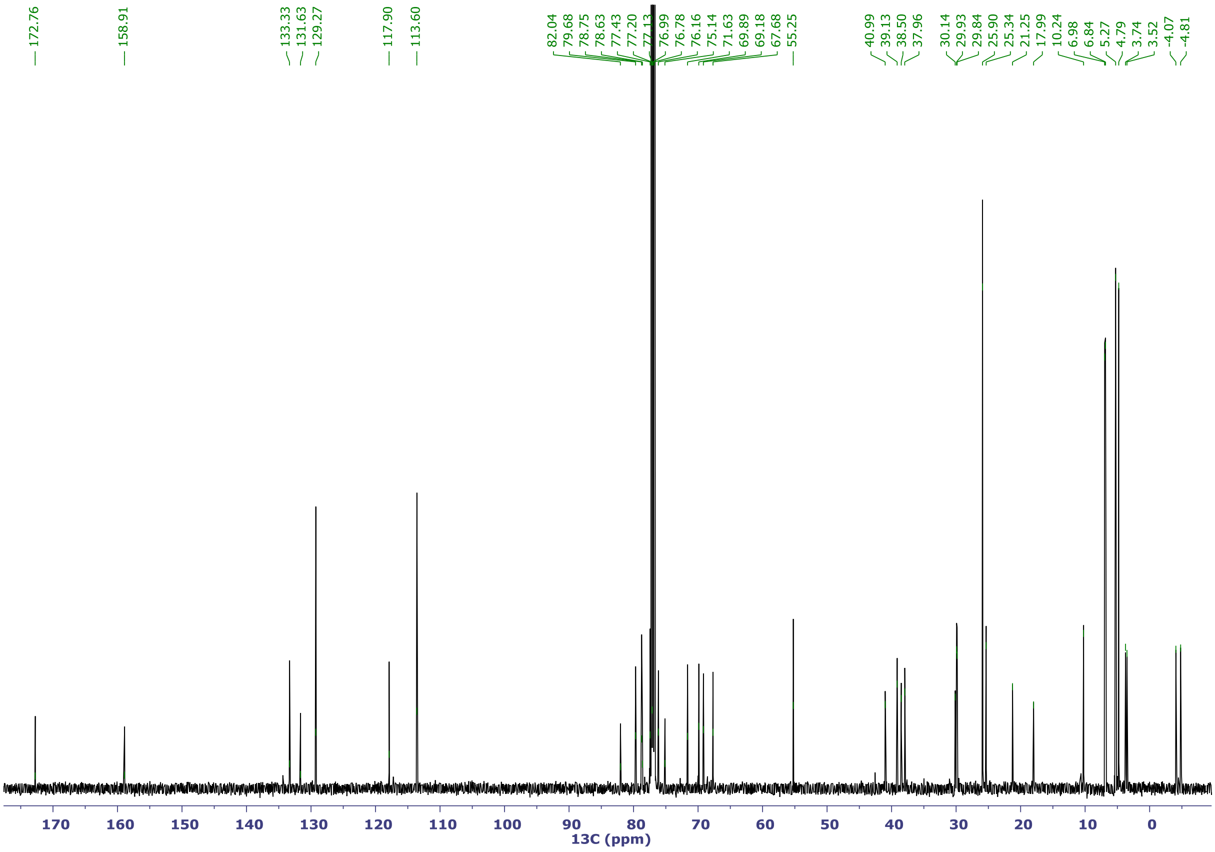

^{13}C NMR (151 MHz, $CDCl_3$)

4-Nitrobenzoate Ester S7

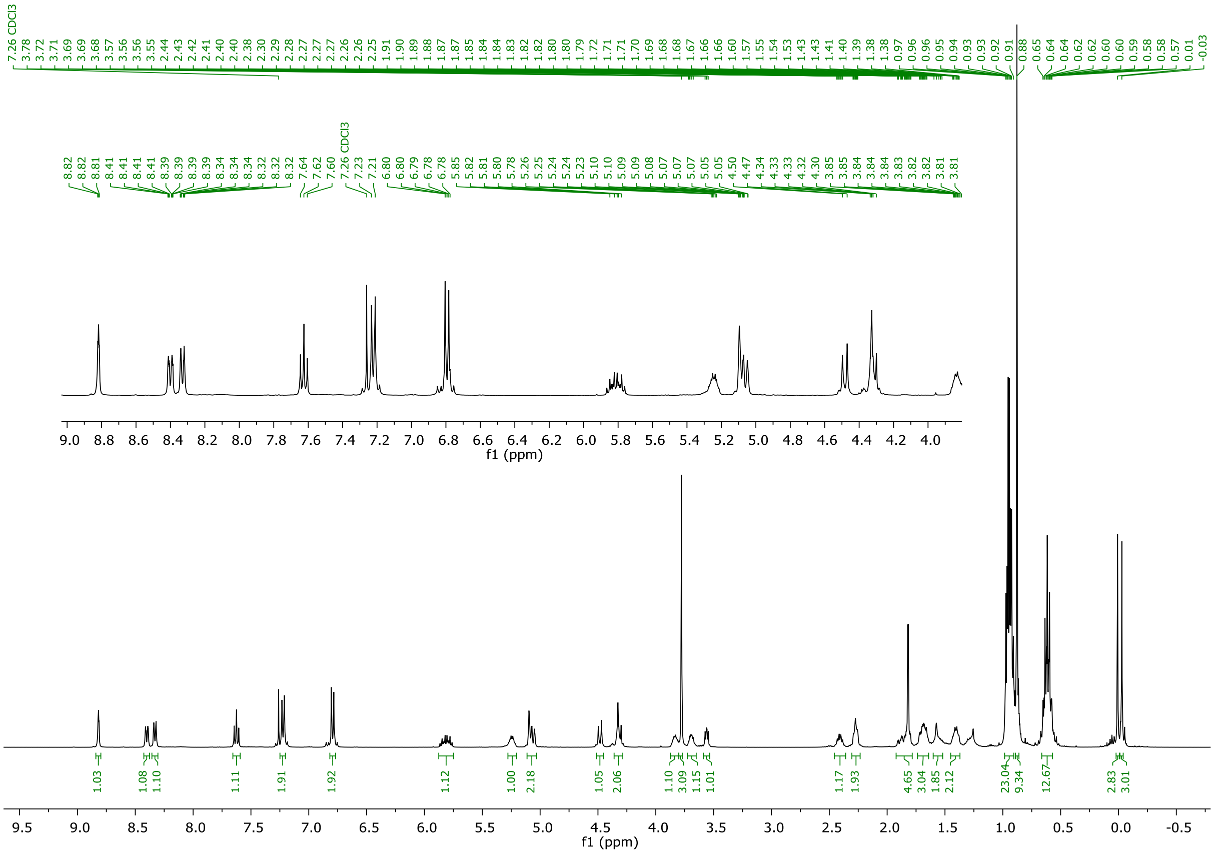

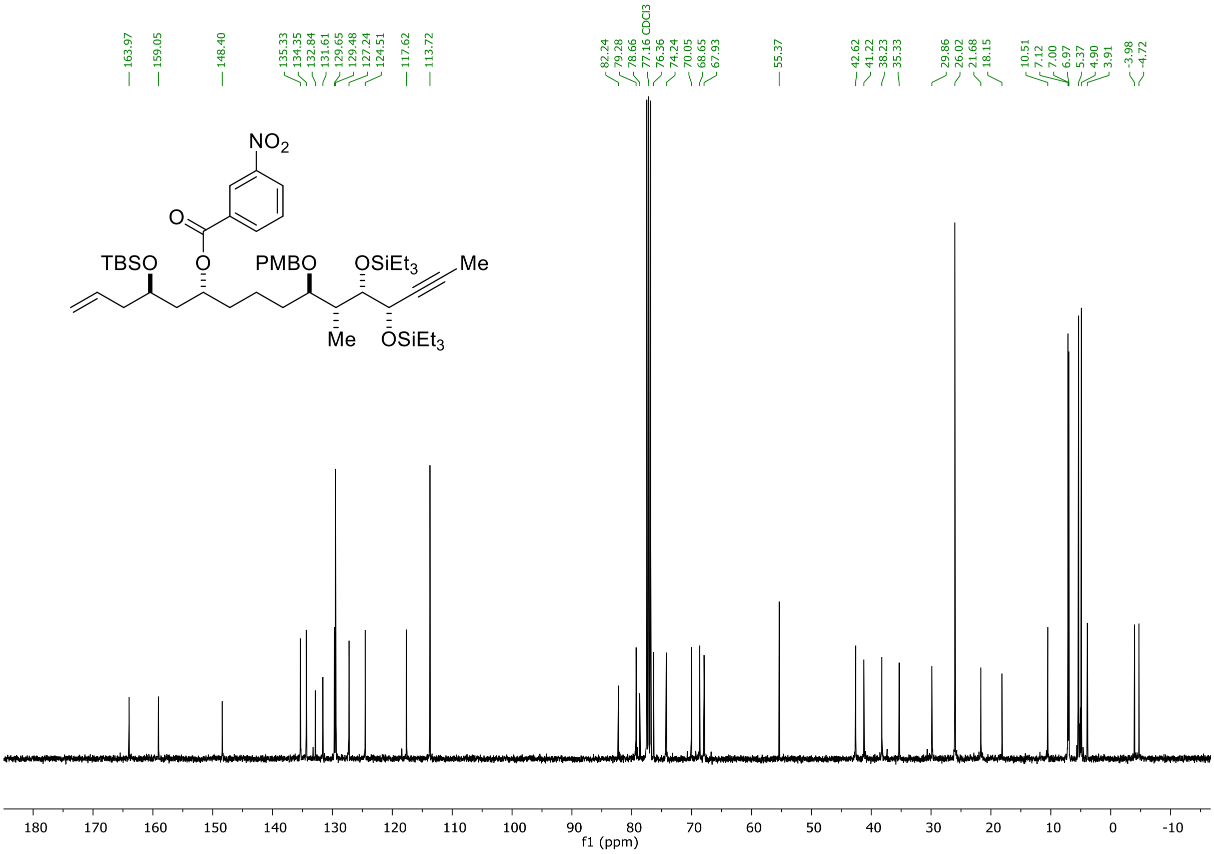

Saponification/Silyl Migration: Compound S8

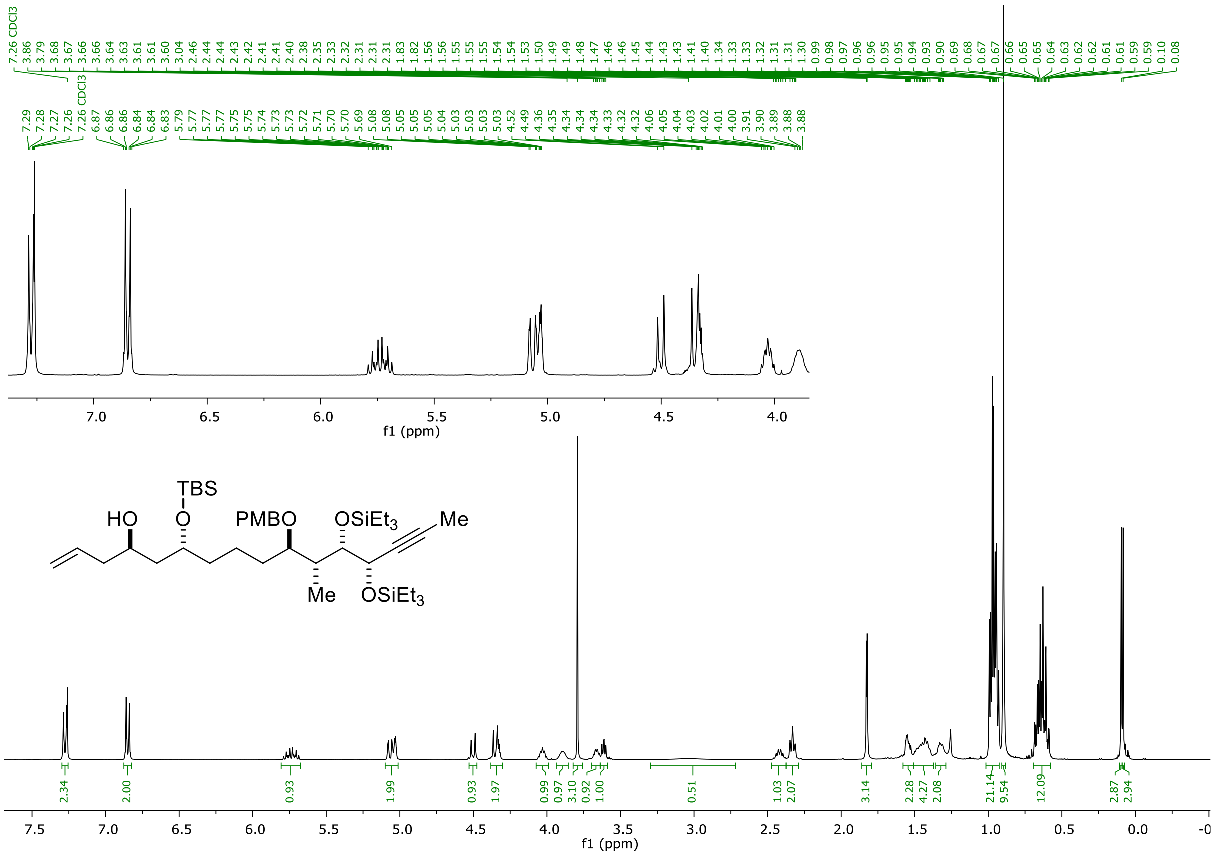

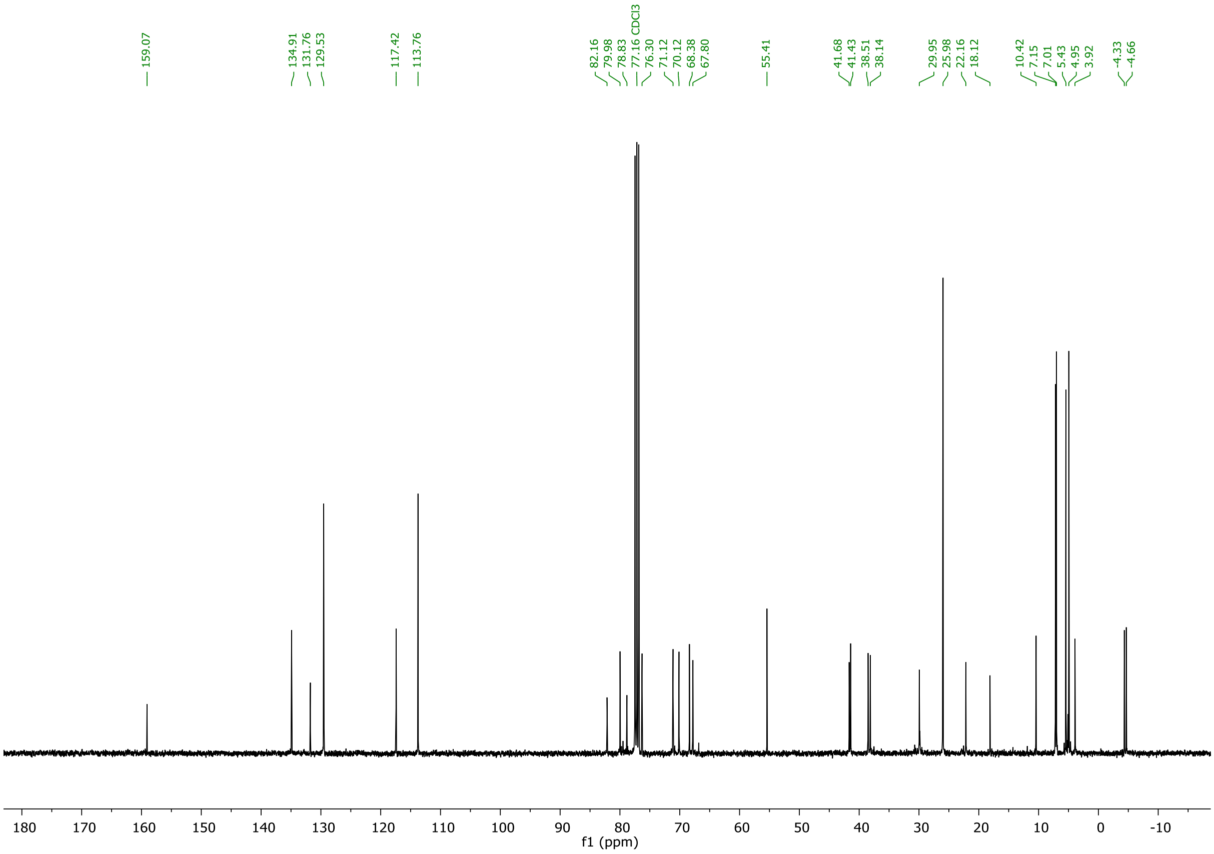

Macrocycle 25

^{1}H NMR (400 MHz, $CDCl_3$)

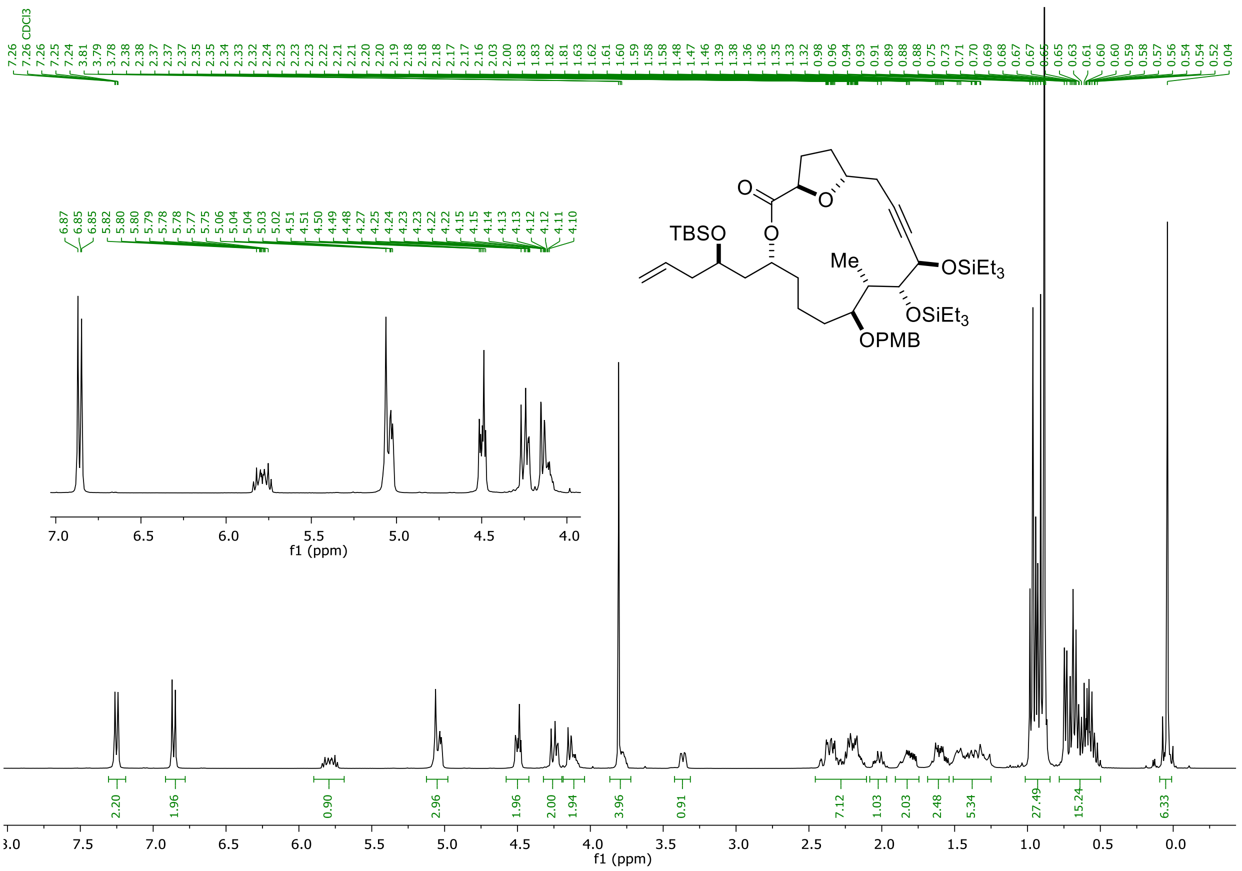

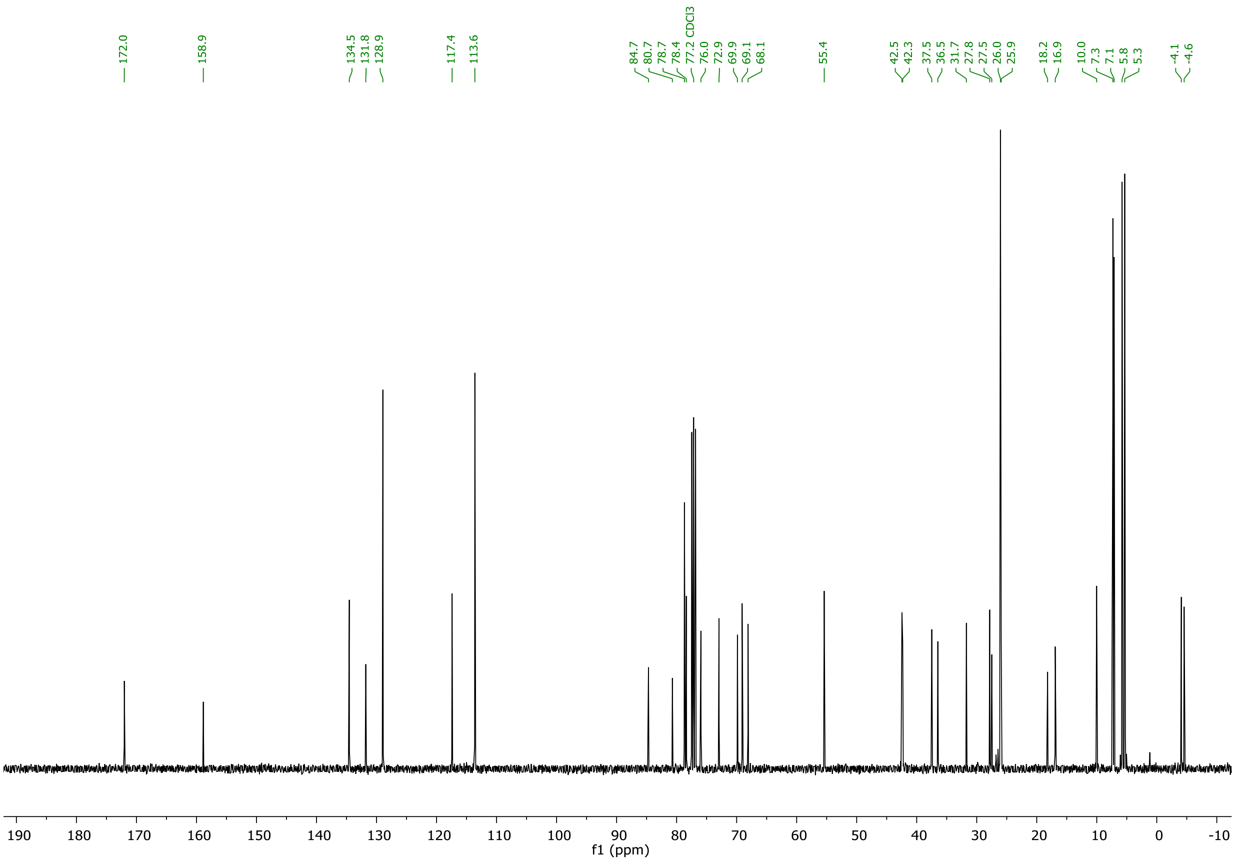

^{13}C NMR (101 MHz, $CDCl_3$)

Macrocycle 21

^1H NMR (400 MHz, $CDCl_3$)

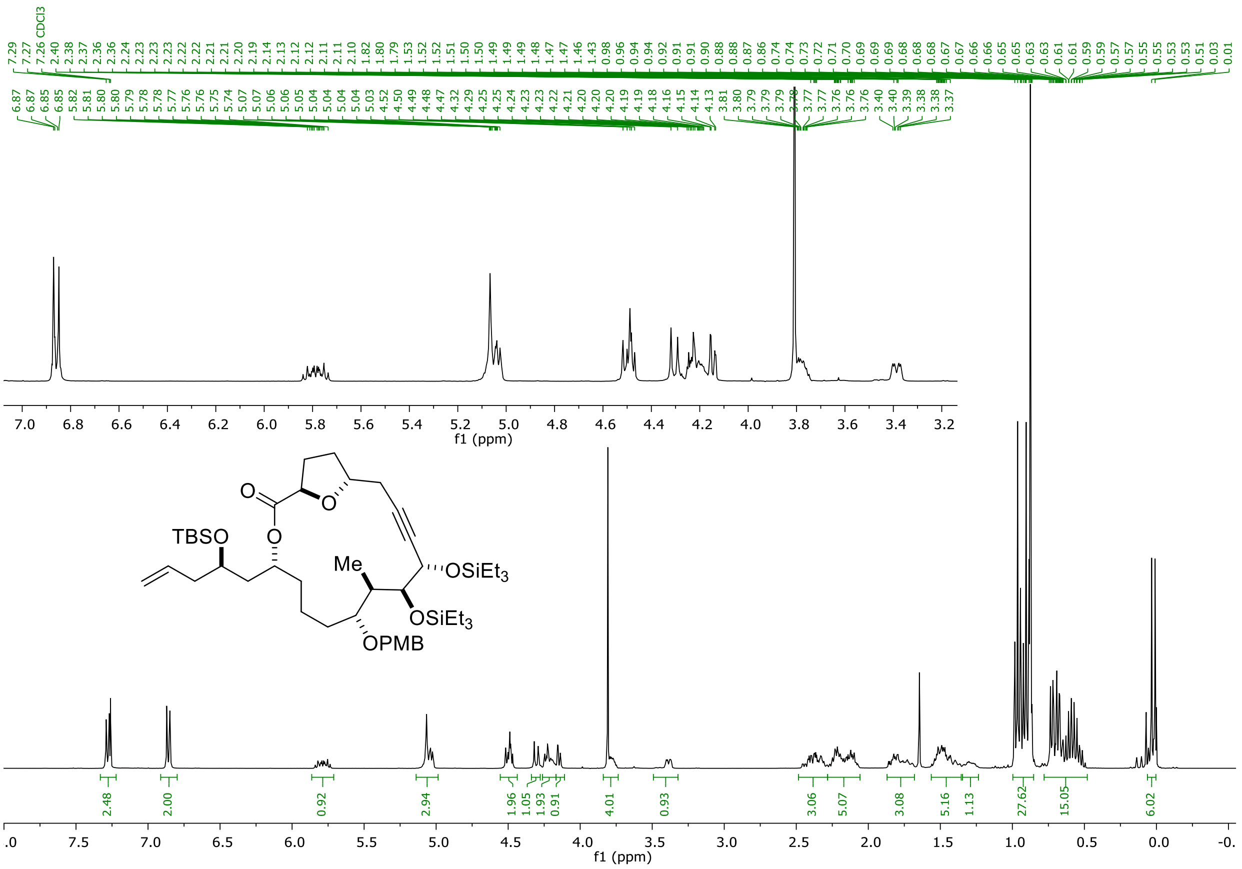

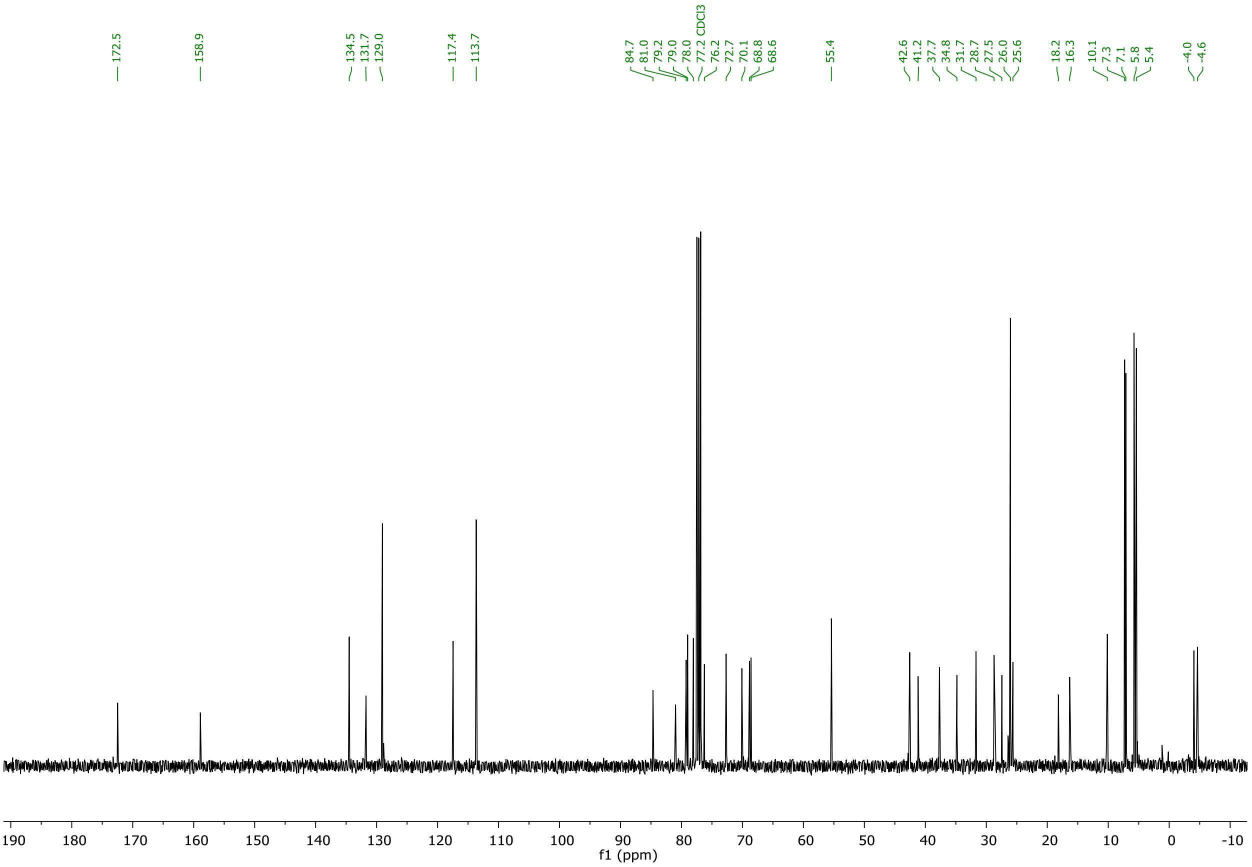

^{13}C NMR (101 MHz, $CDCl_3$)

Alcohol 33

^{1}H NMR (400 MHz, $CDCl_3$)

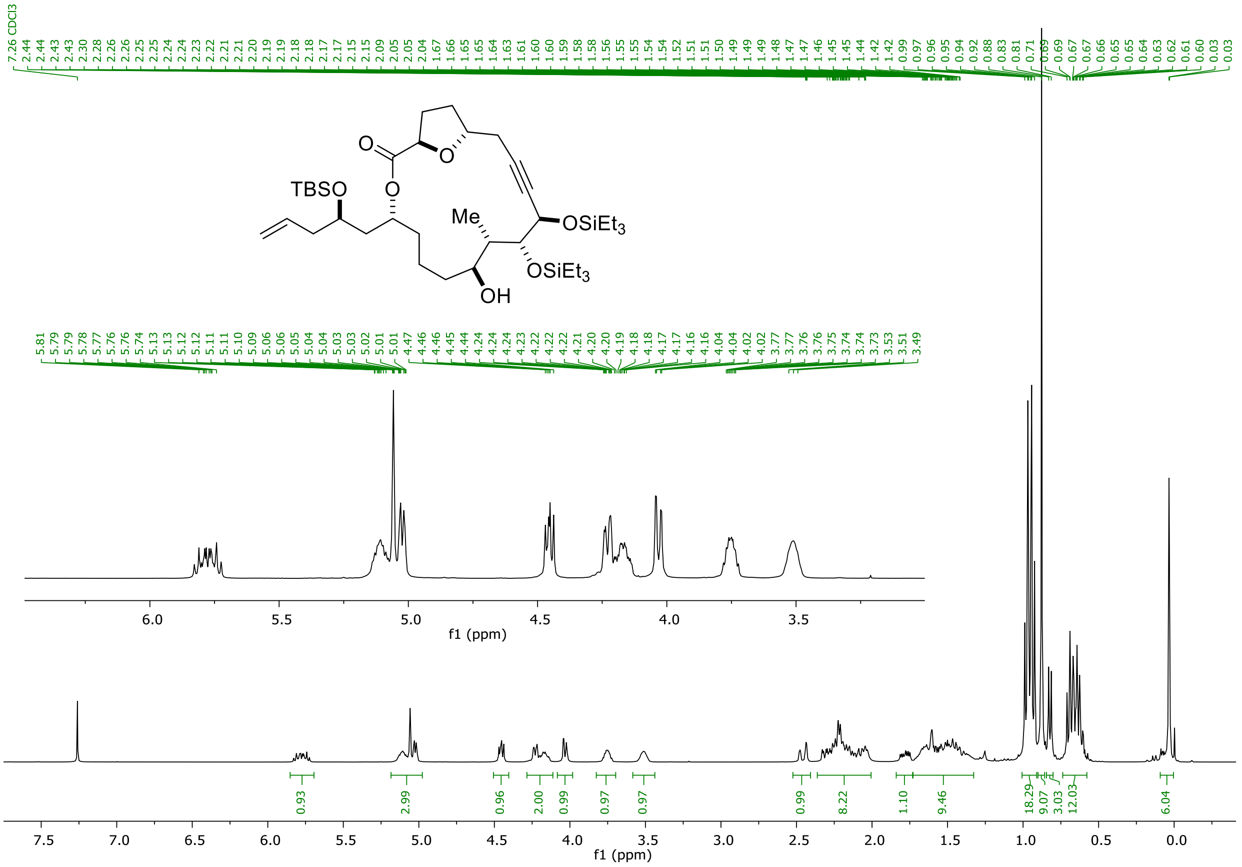

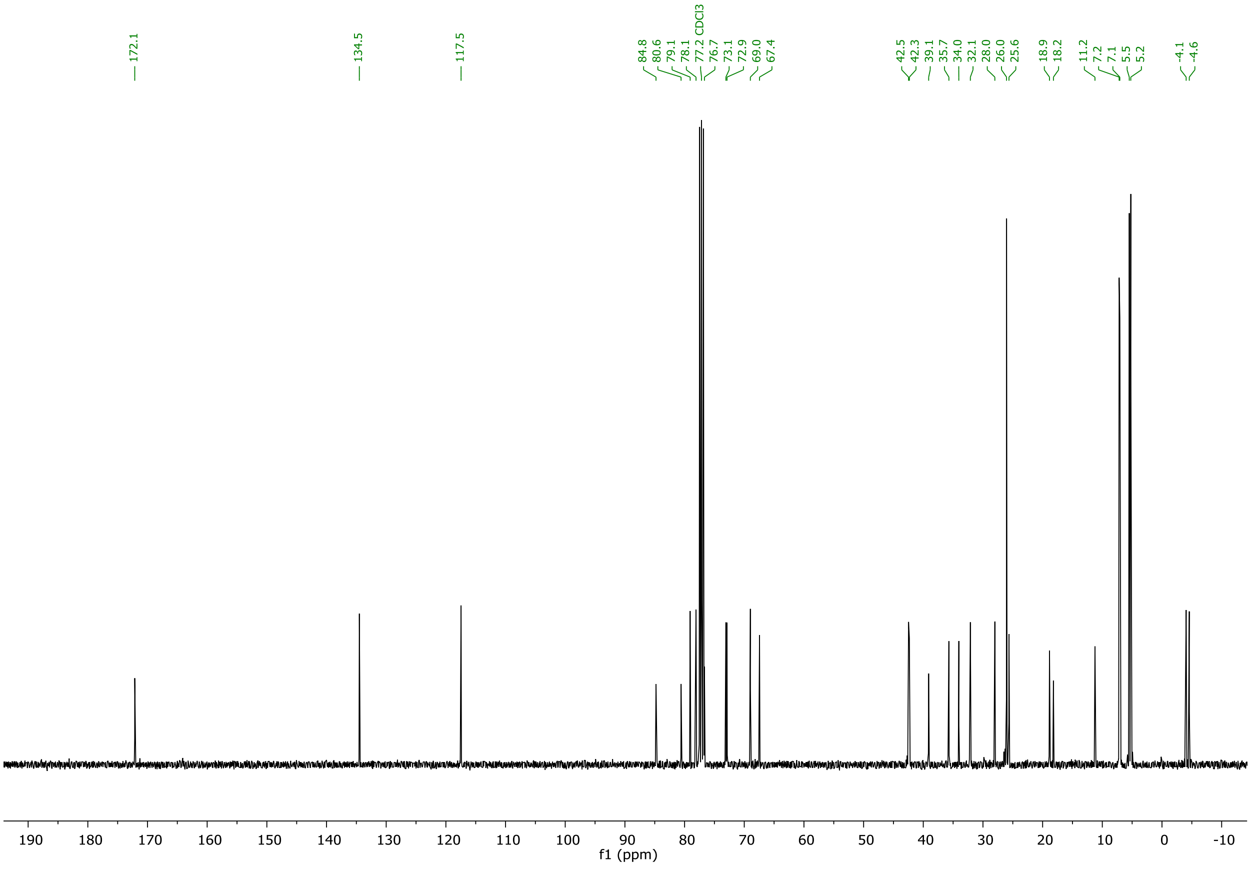

^{13}C NMR (101 MHz, $CDCl_3$)

Alcohol 30

^{1}H NMR (400 MHz, $CDCl_3$)

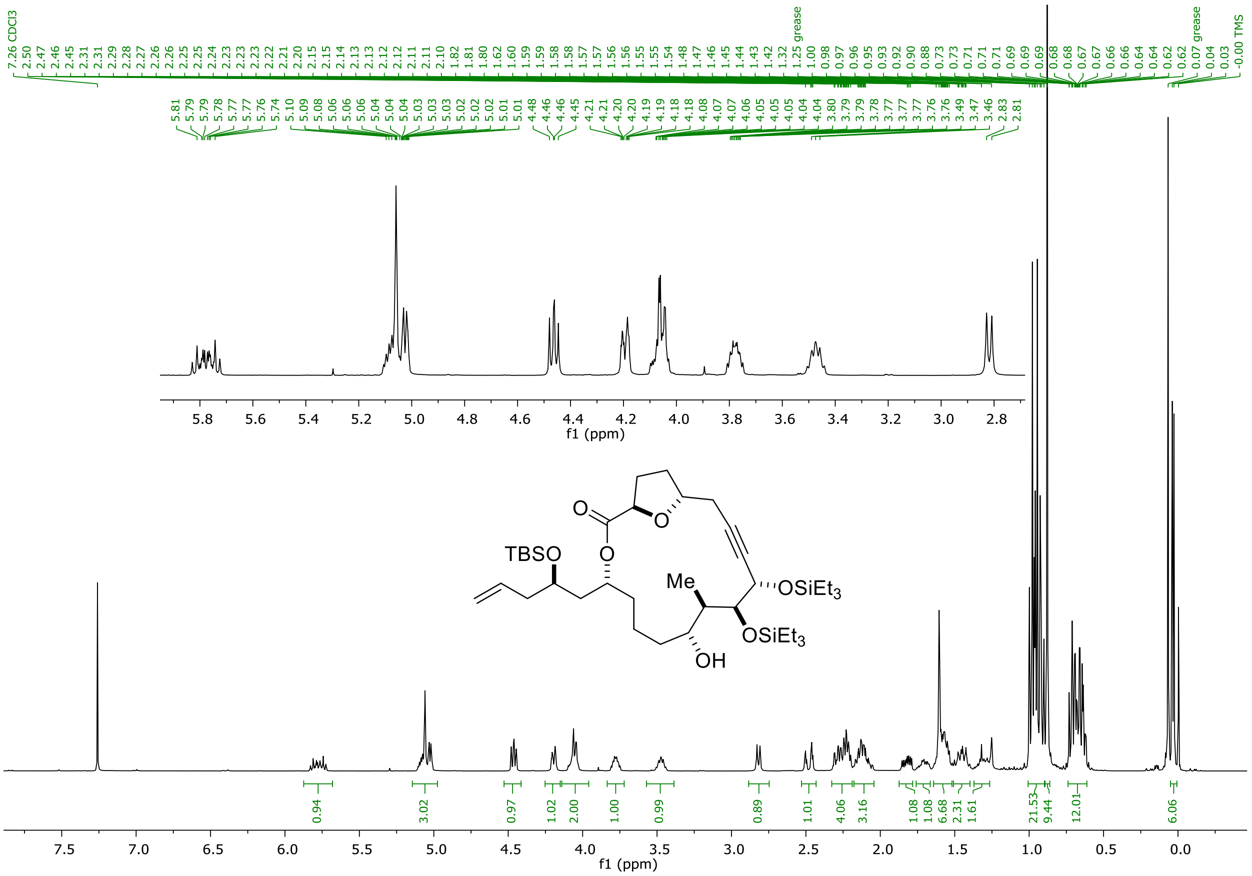

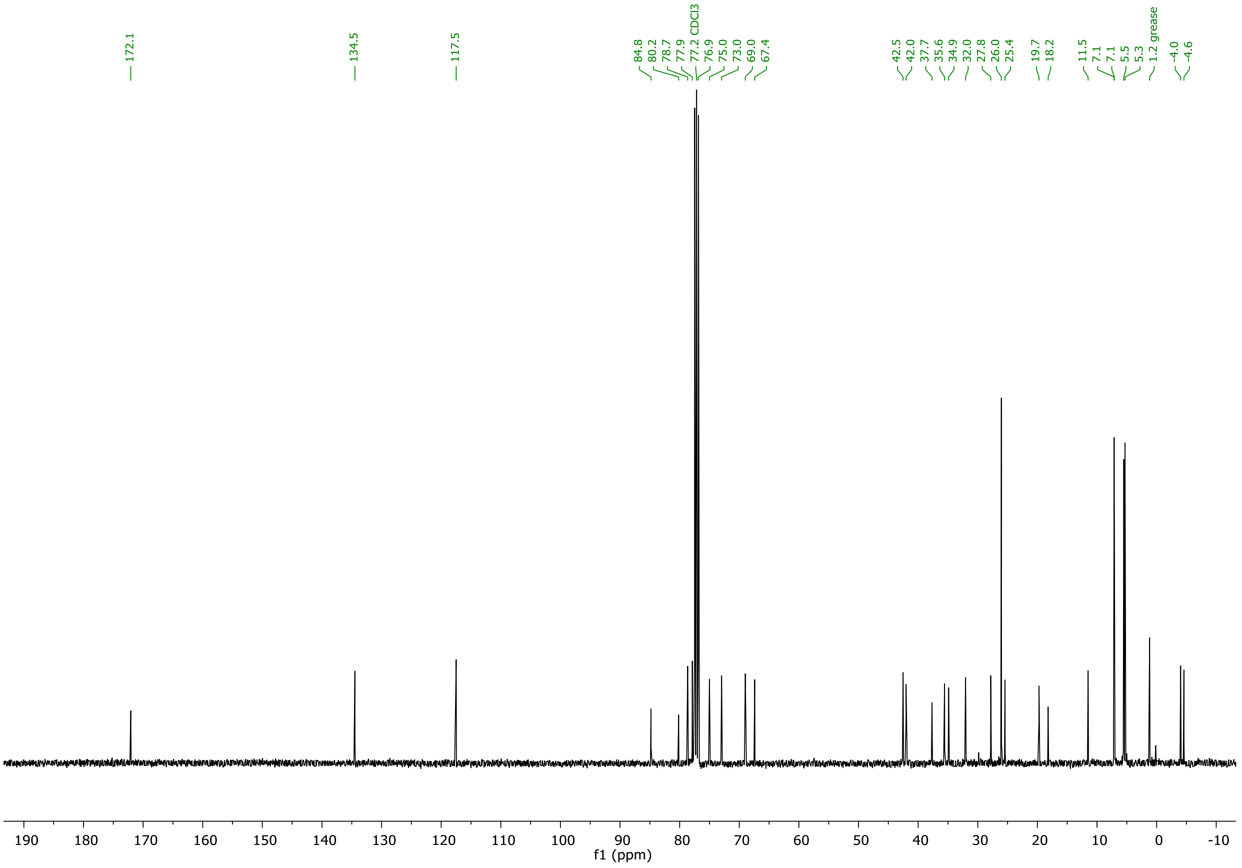

^{13}C NMR (101 MHz, $CDCl_3$)

Enol ether 34

^{1}H NMR (400 MHz, $CDCl_3$)

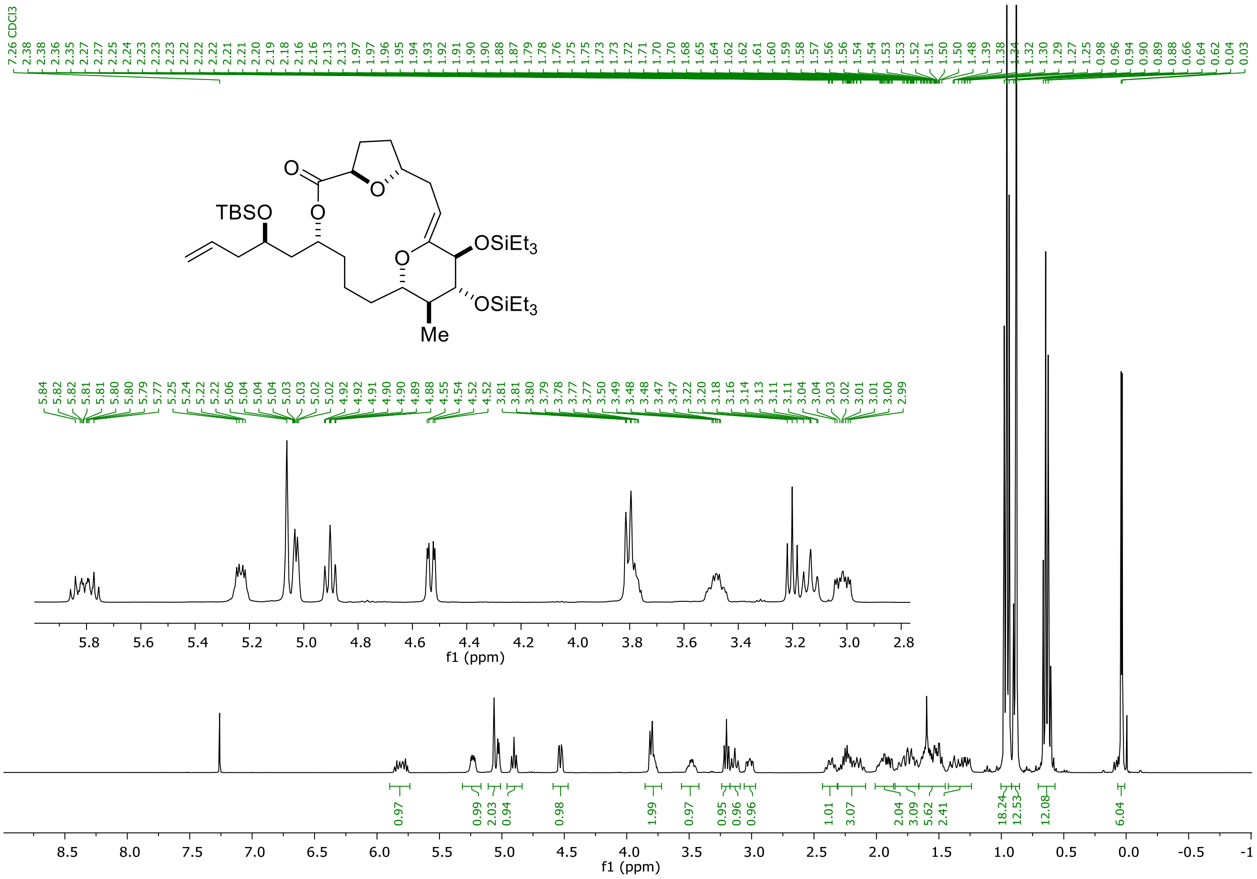

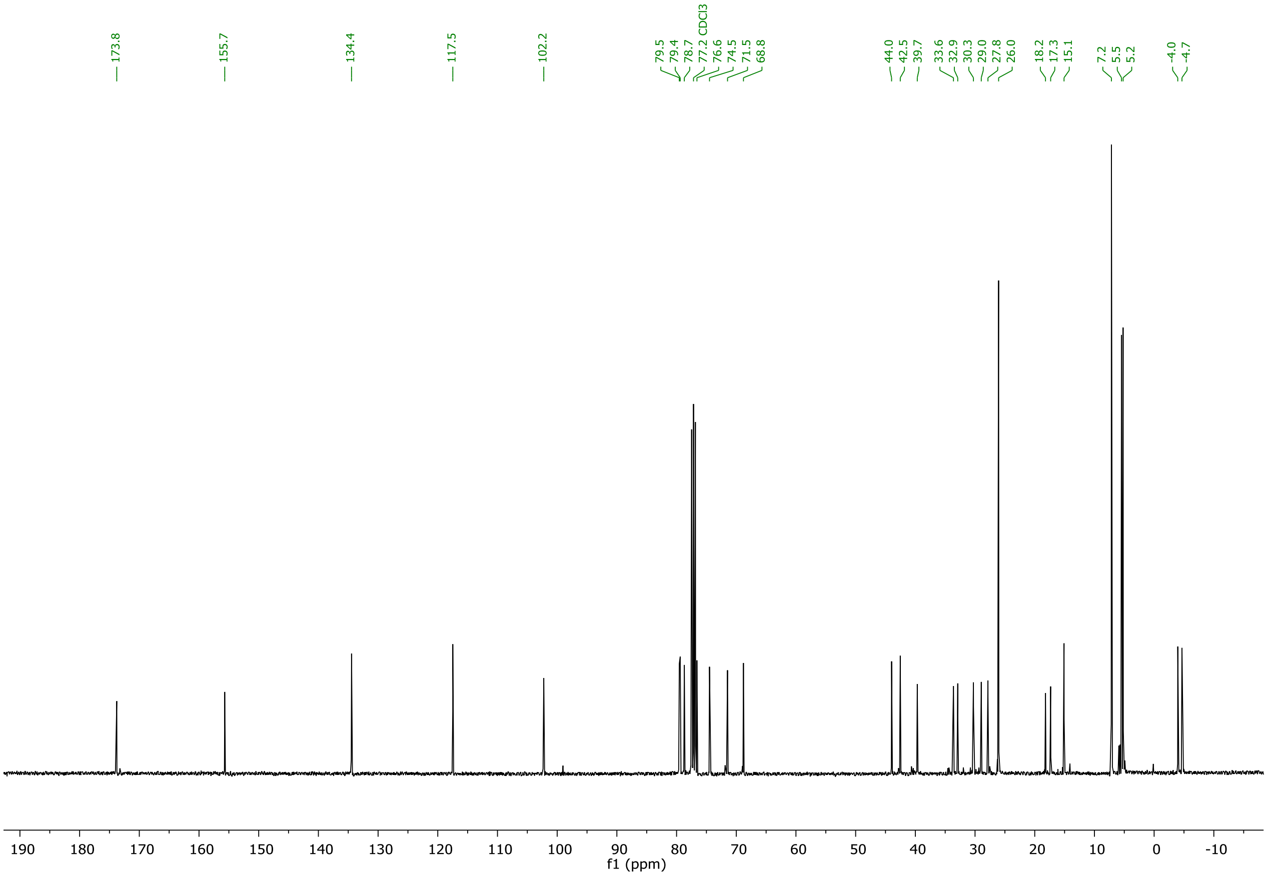

^{13}C NMR (101 MHz, $CDCl_3$)

Enol ether 31

^{1}H NMR (400 MHz, CD_2Cl_2)

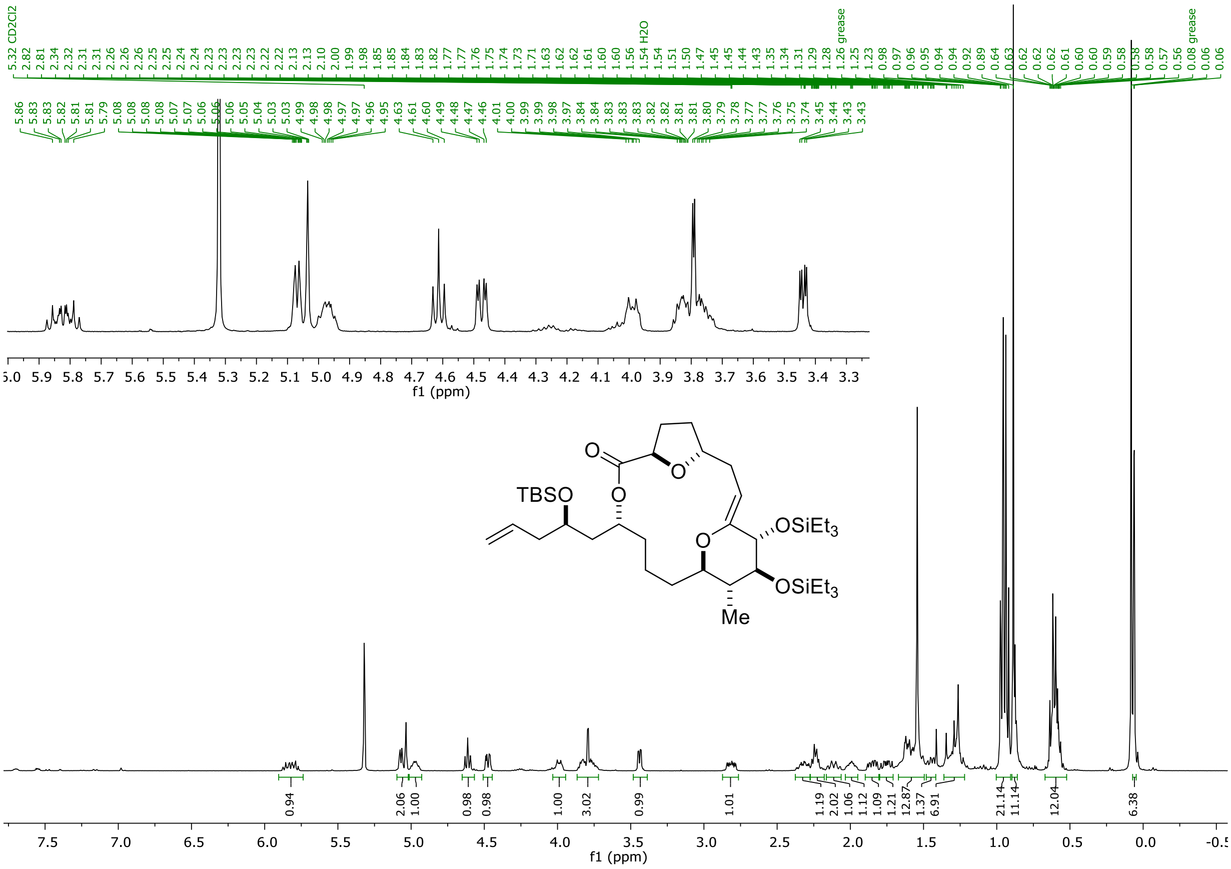

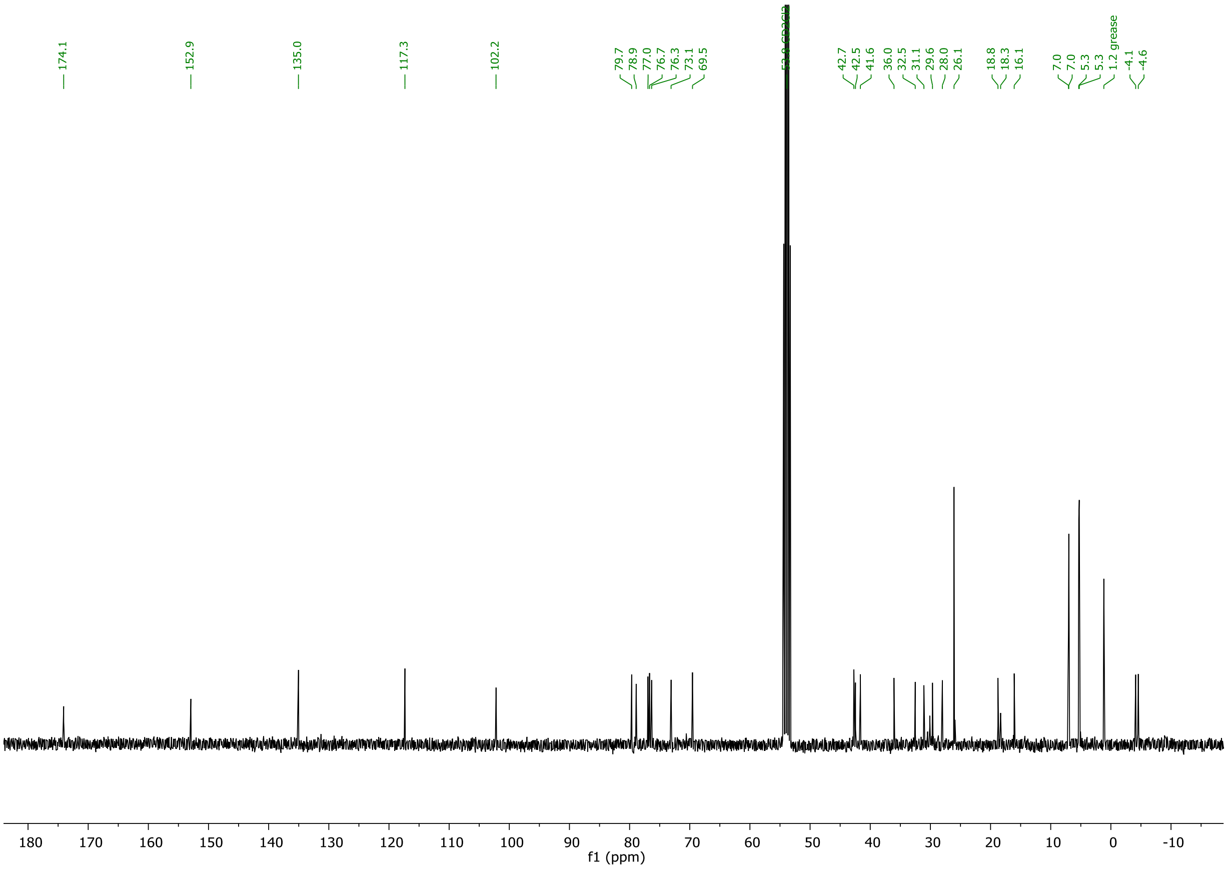

^{13}C NMR (101 MHz, CD_2Cl_2)

Triol 35

^{1}H NMR (600 MHz, $CDCl_3$)

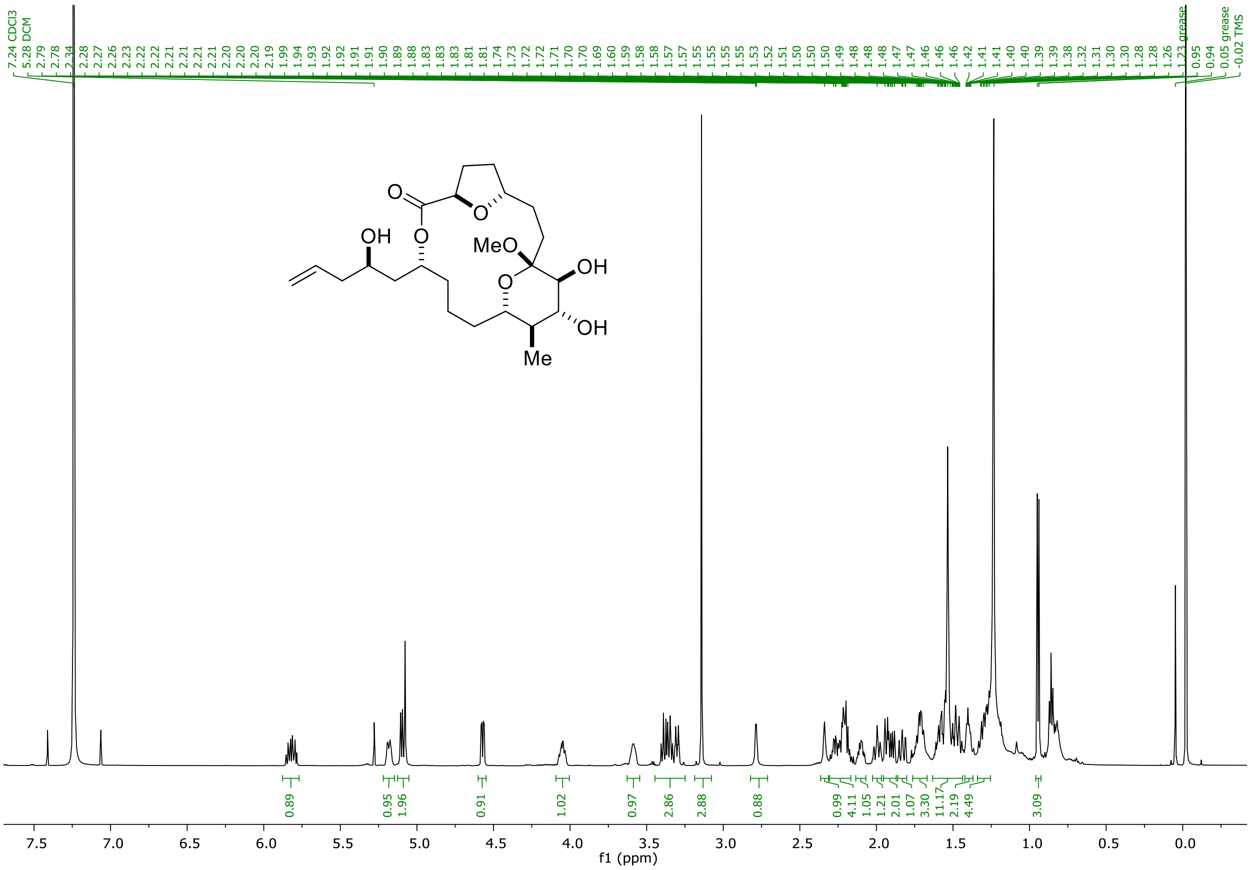

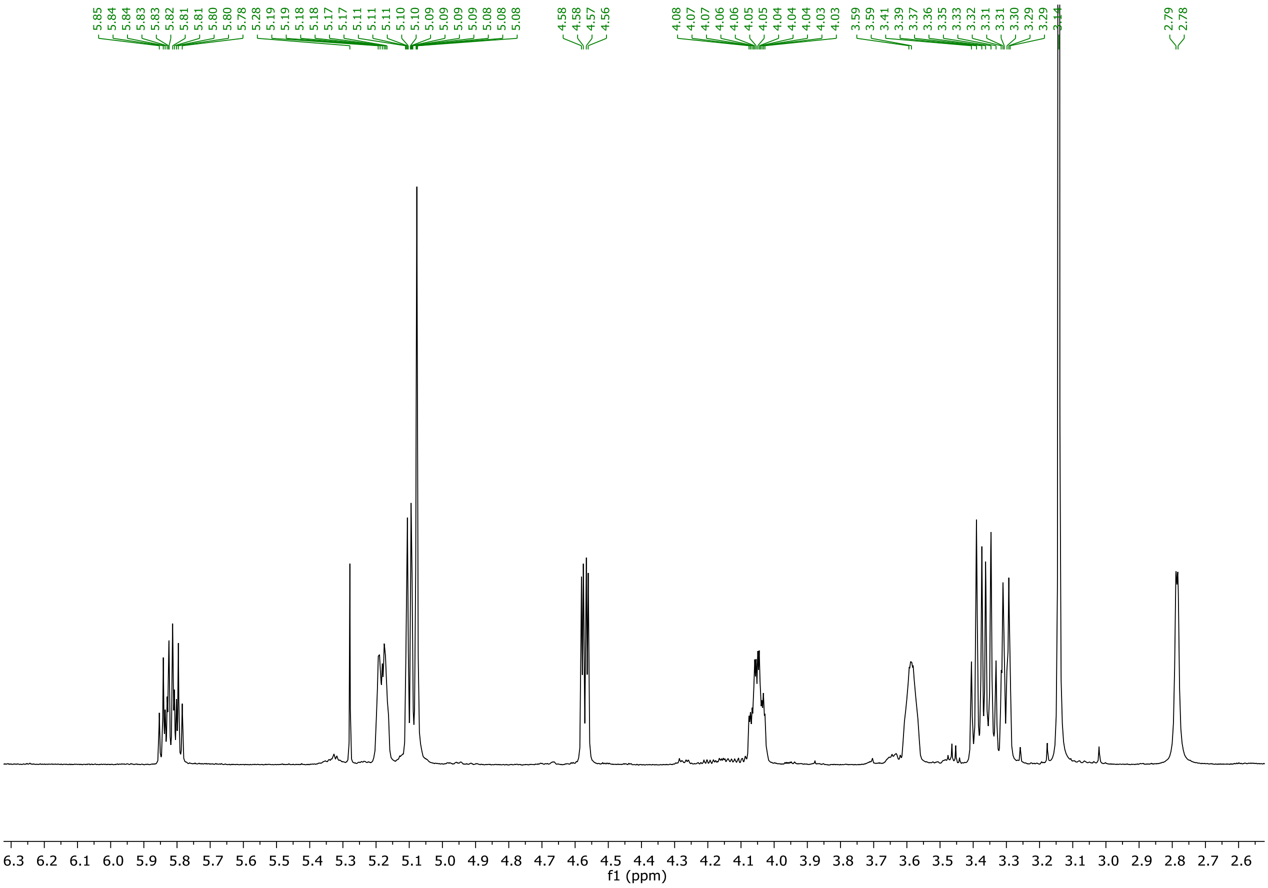

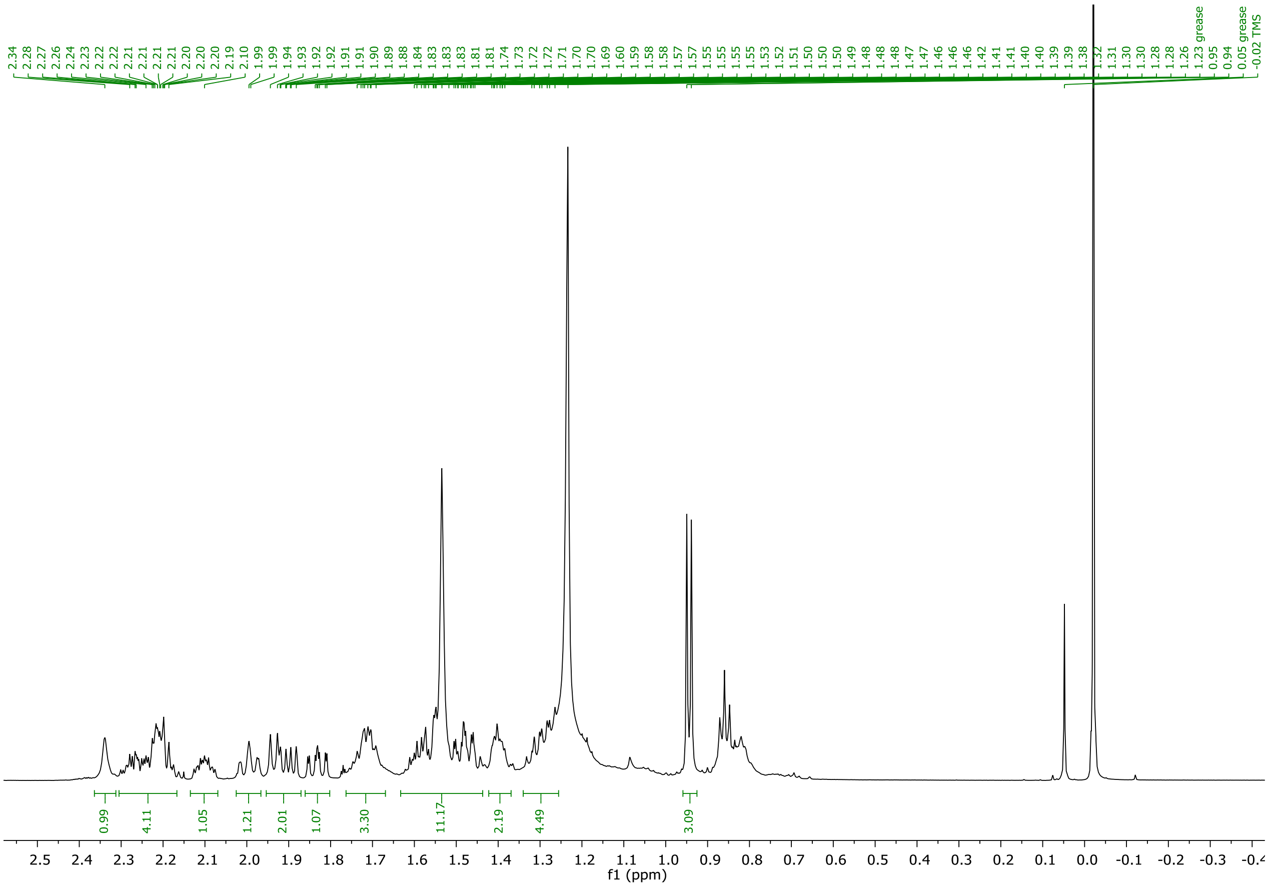

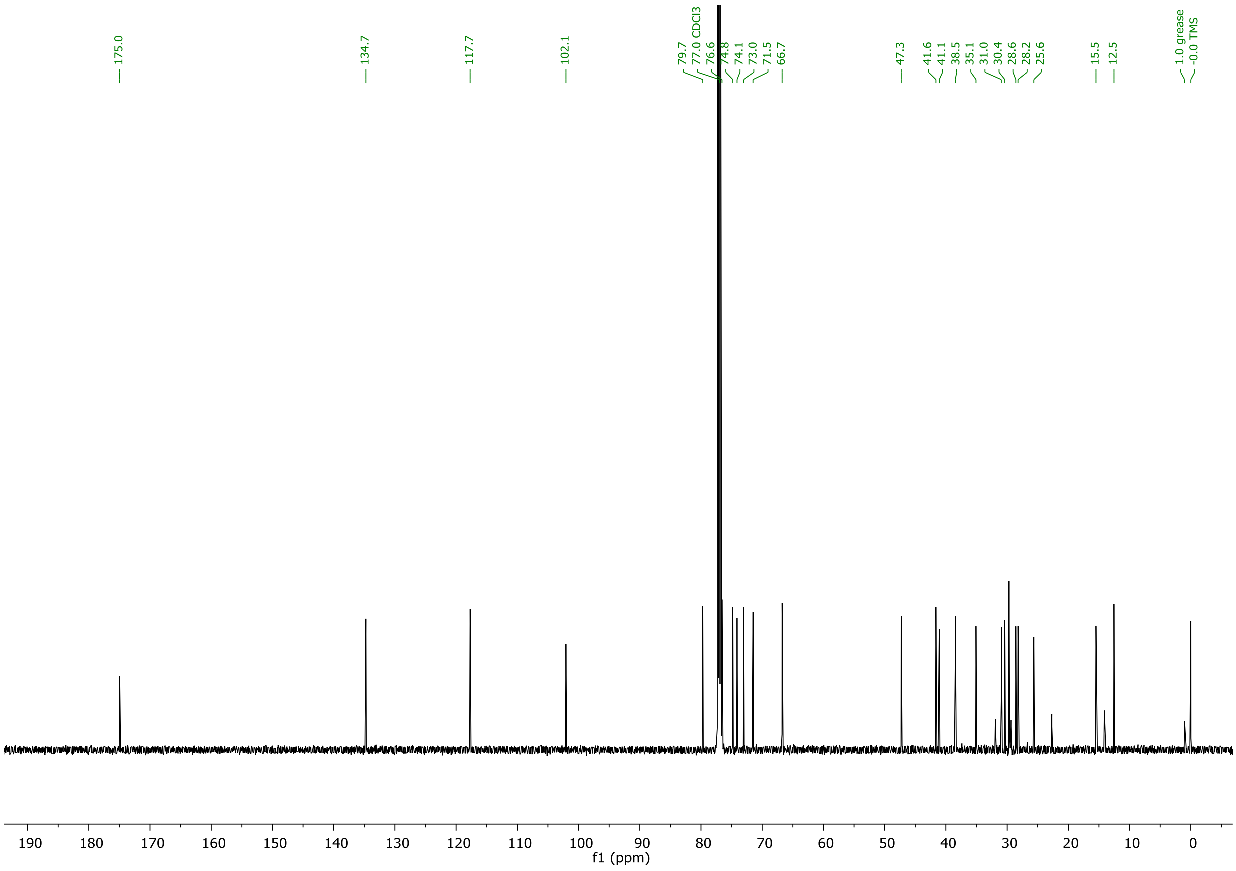

^{13}C NMR (151 MHz, $CDCl_3$)

Triol 32

^{1}H NMR (600 MHz, $CDCl_3$)

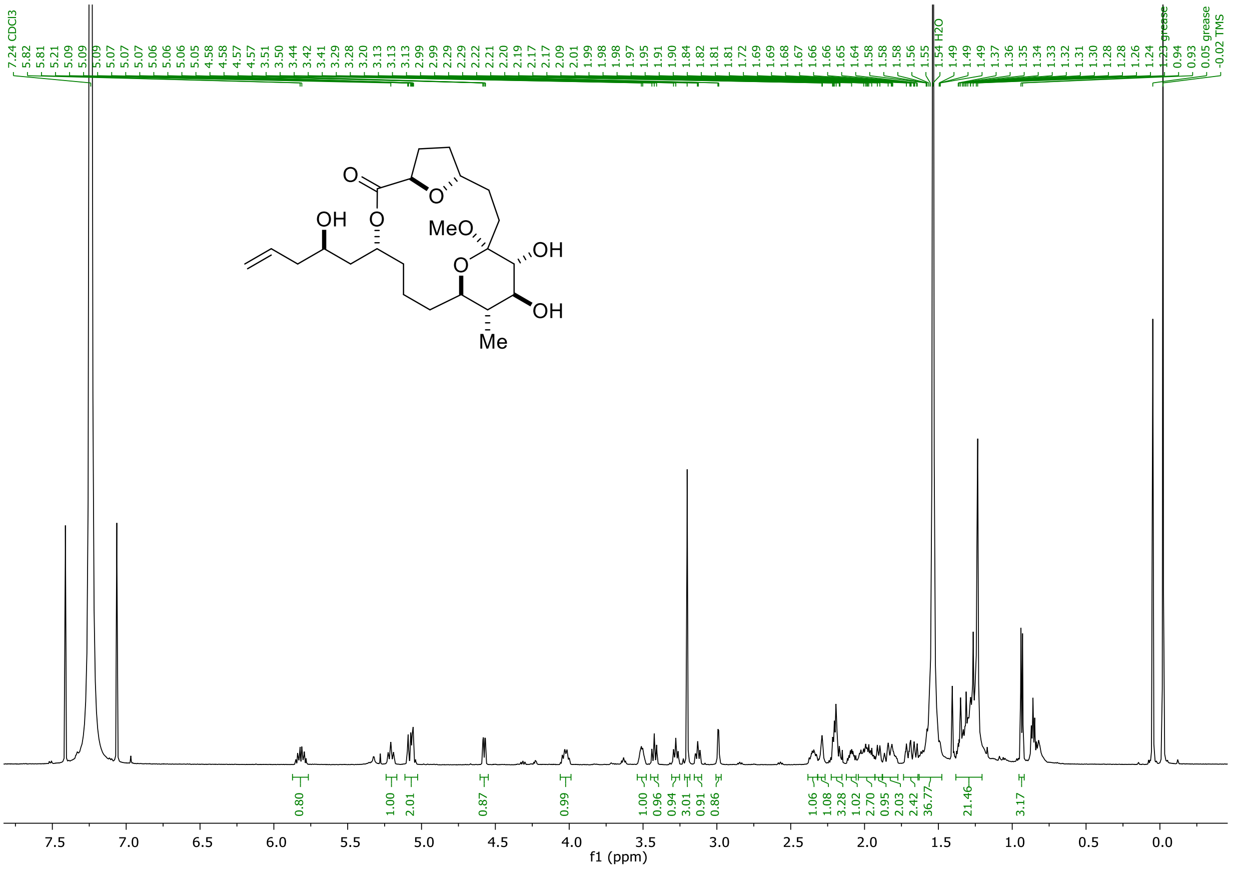

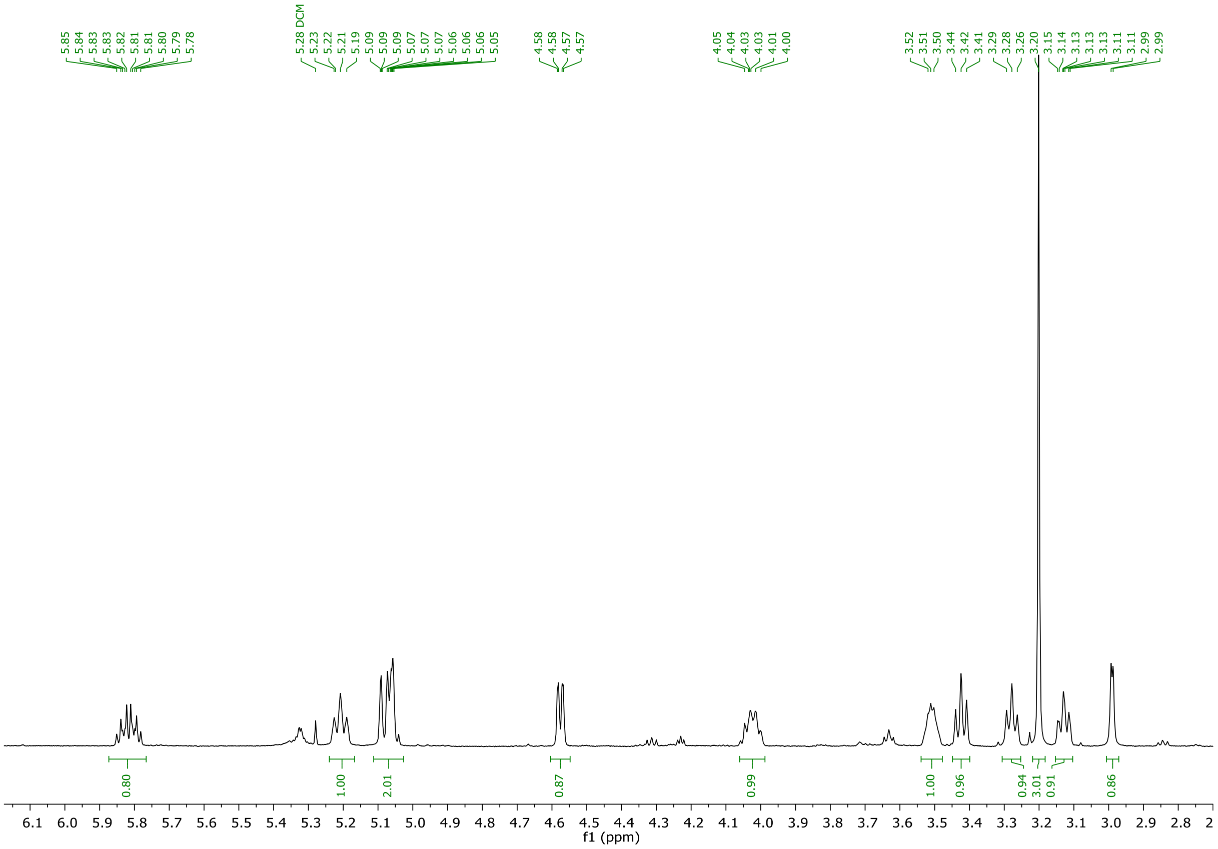

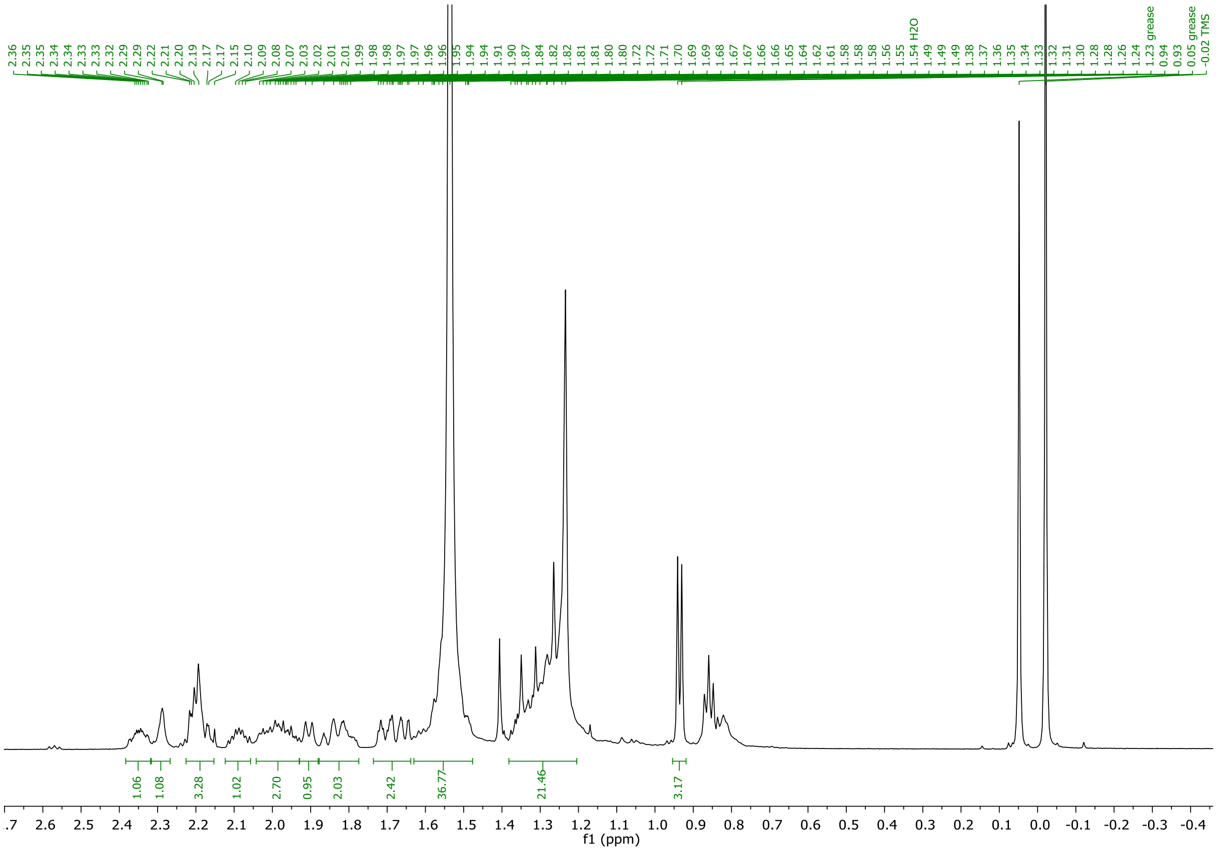

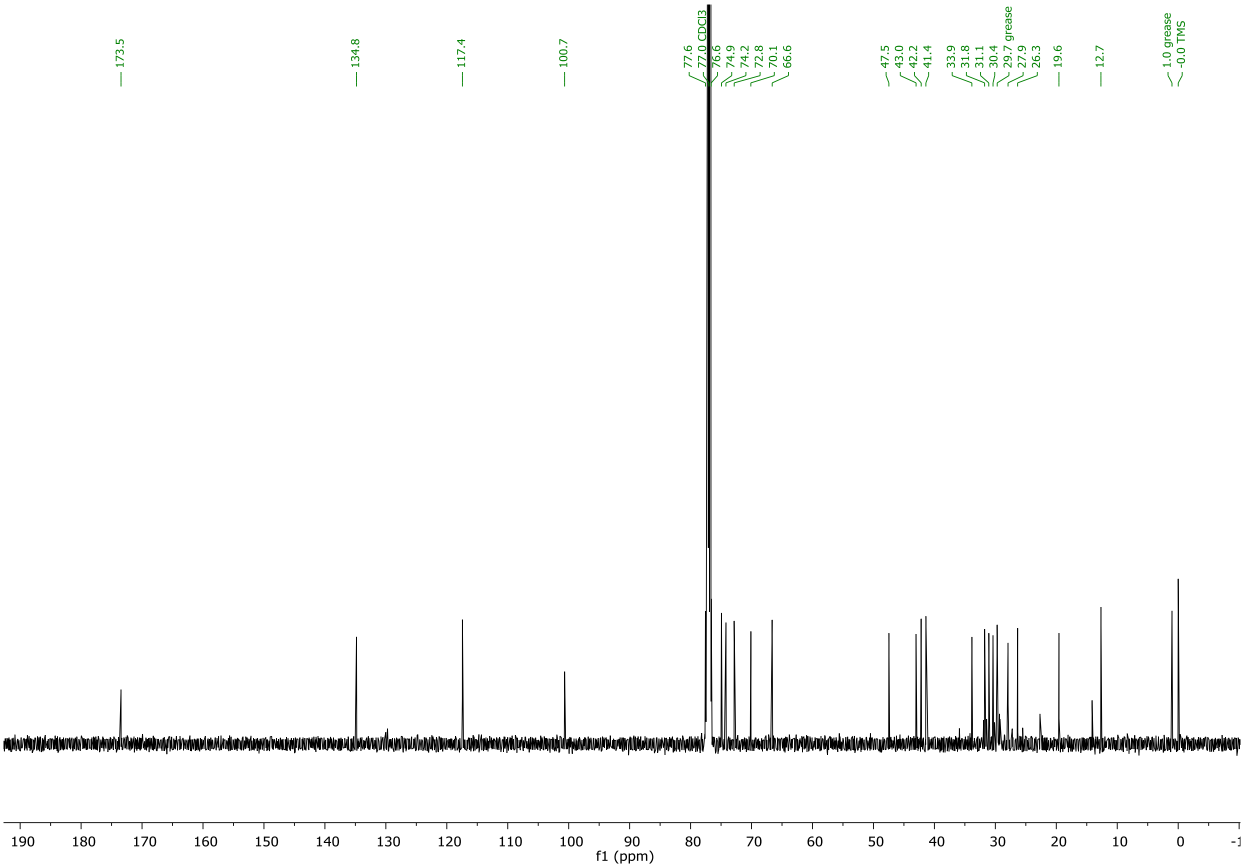

^{13}C NMR (151 MHz, $CDCl_3$)

(*Z*)-5-((*tert*-Butyldimethylsilyl)oxy)pent-2-enal (44)

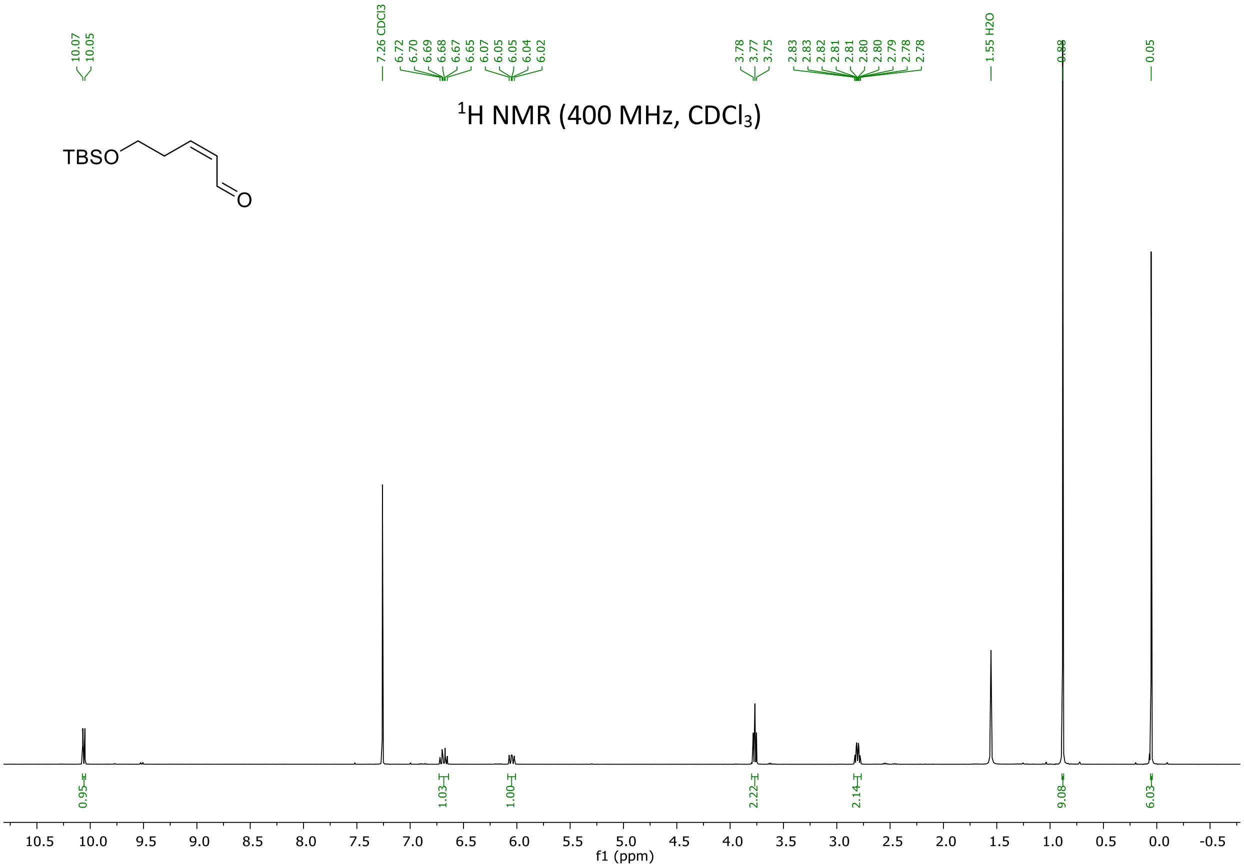

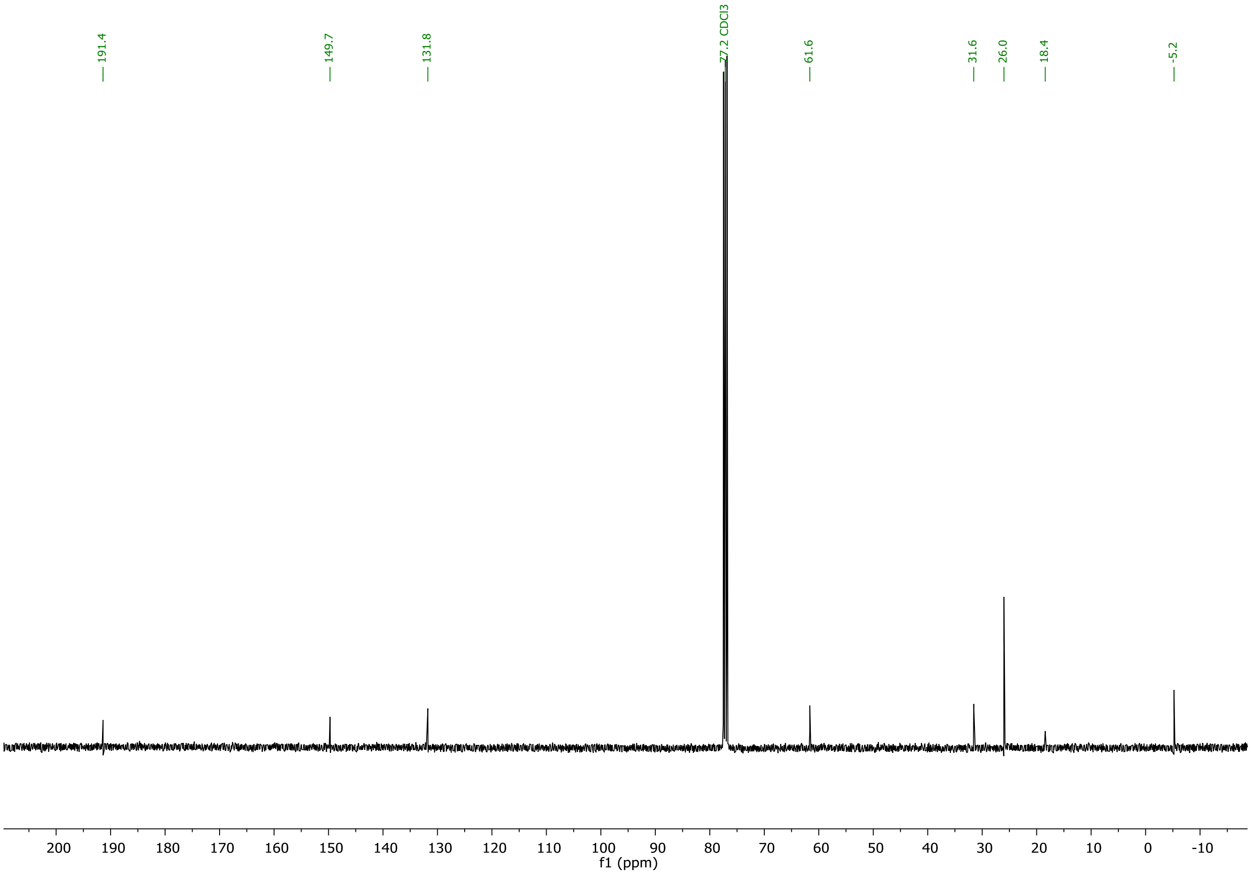

^{13}C NMR (101 MHz, $CDCl_3$)

Phosphonium Salt 43

^{1}H NMR (400 MHz, d_6-DMSO)

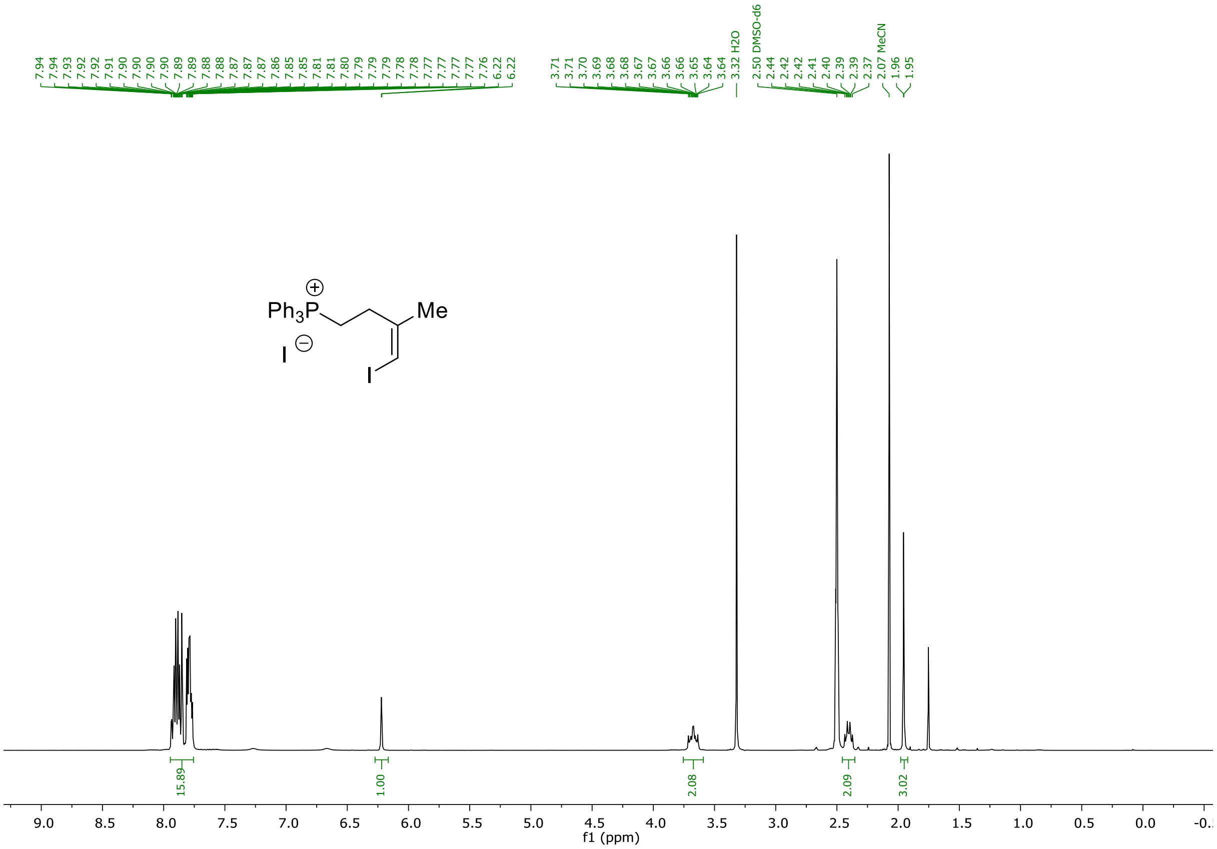

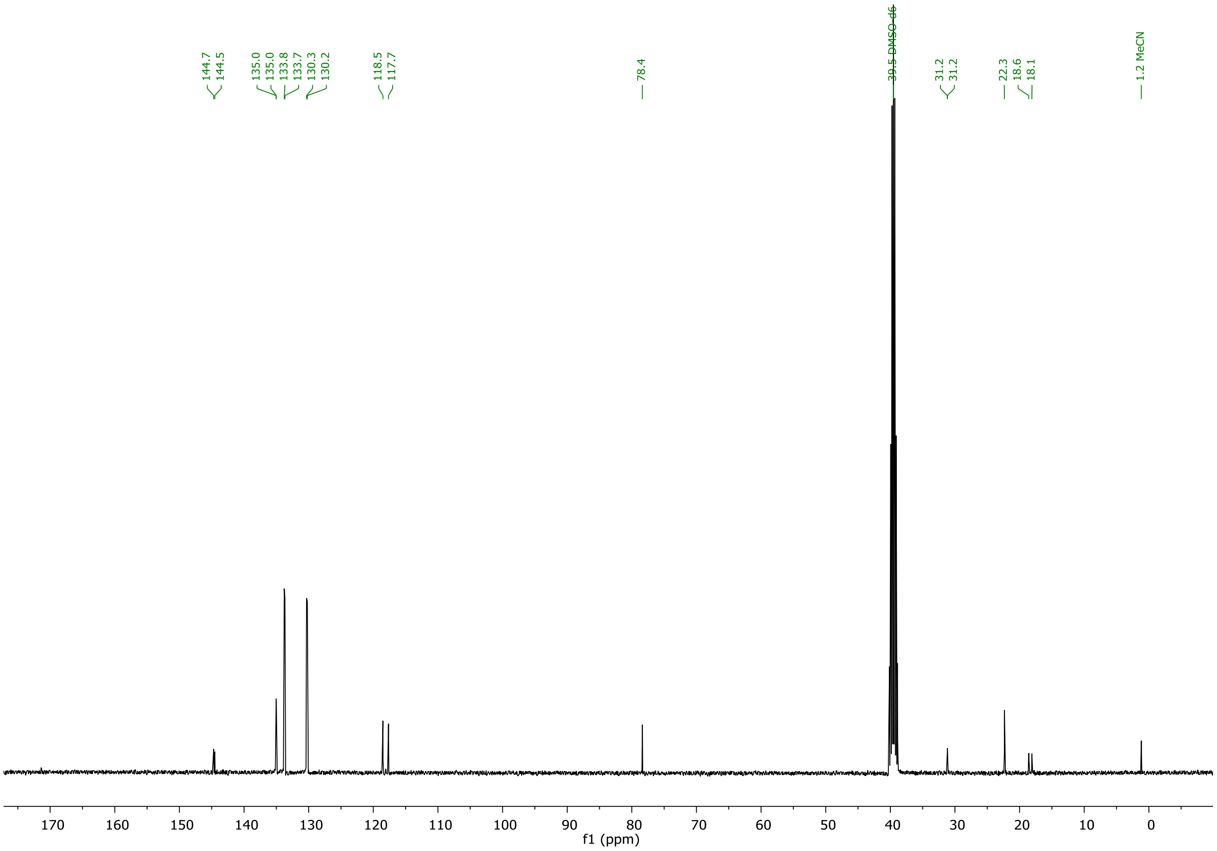

^{13}C NMR (101 MHz, d_6-DMSO)

Diene 45

^{1}H NMR (400 MHz, CD_2Cl_2)

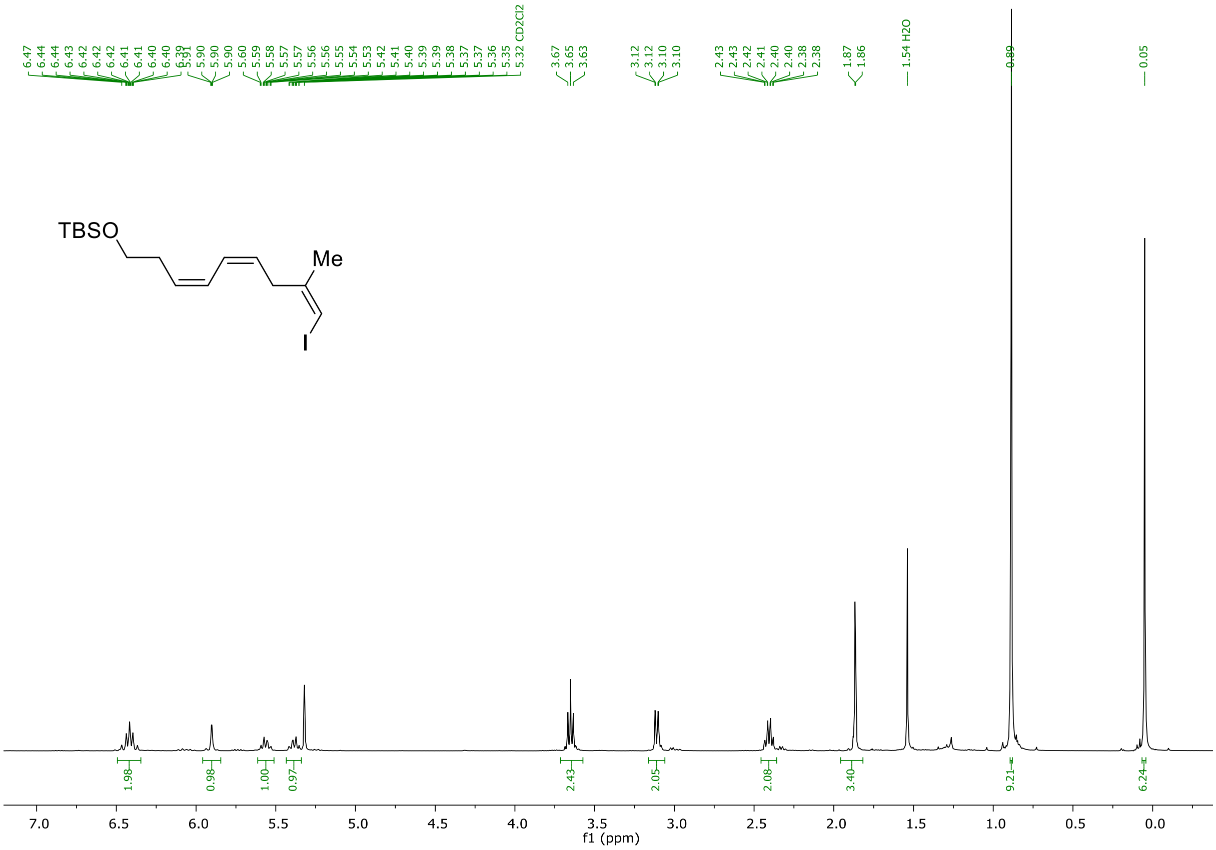

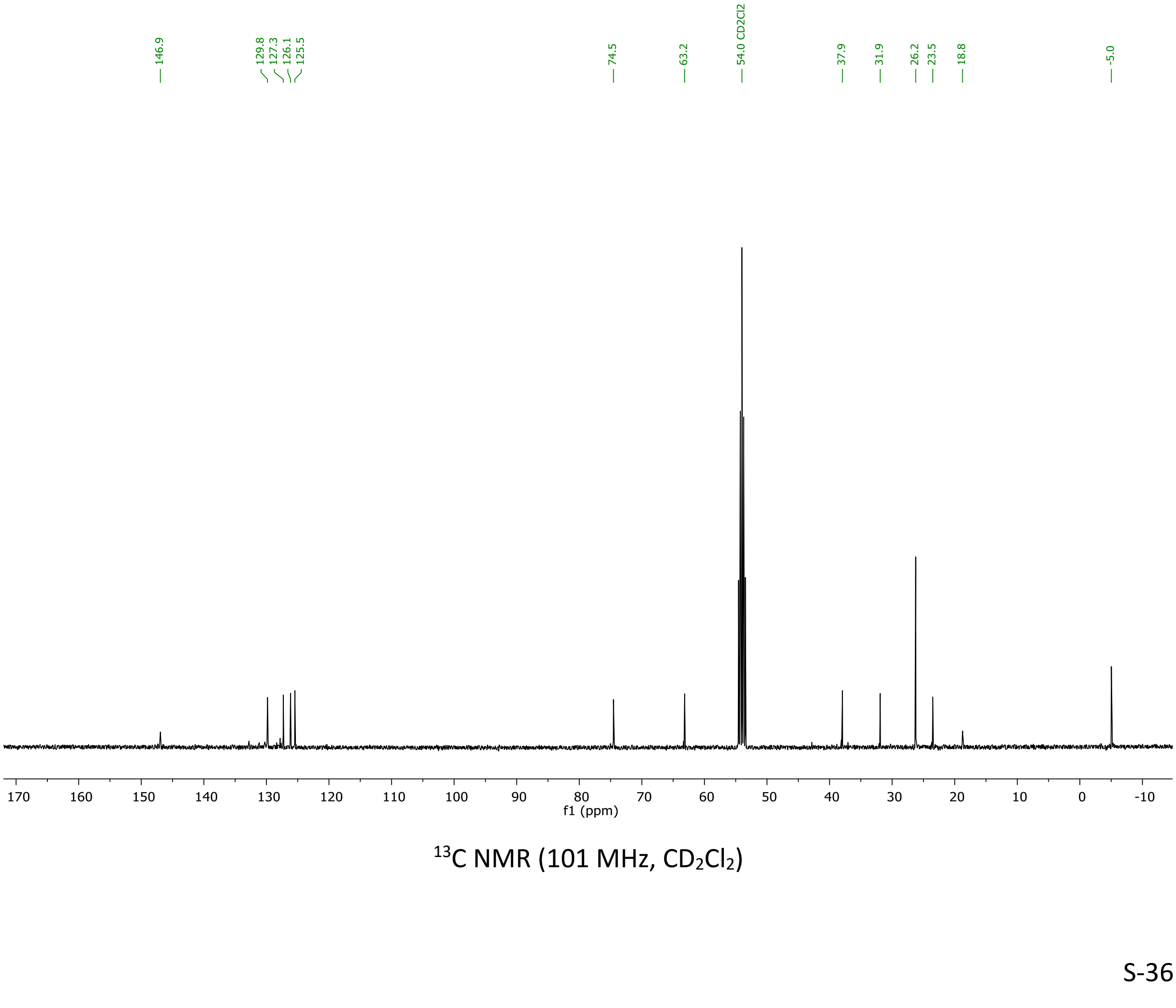

^{13}C NMR (101 MHz, CD_2Cl_2)

Alkenyl stannane 46

^{1}H NMR (400 MHz, CD_2Cl_2)

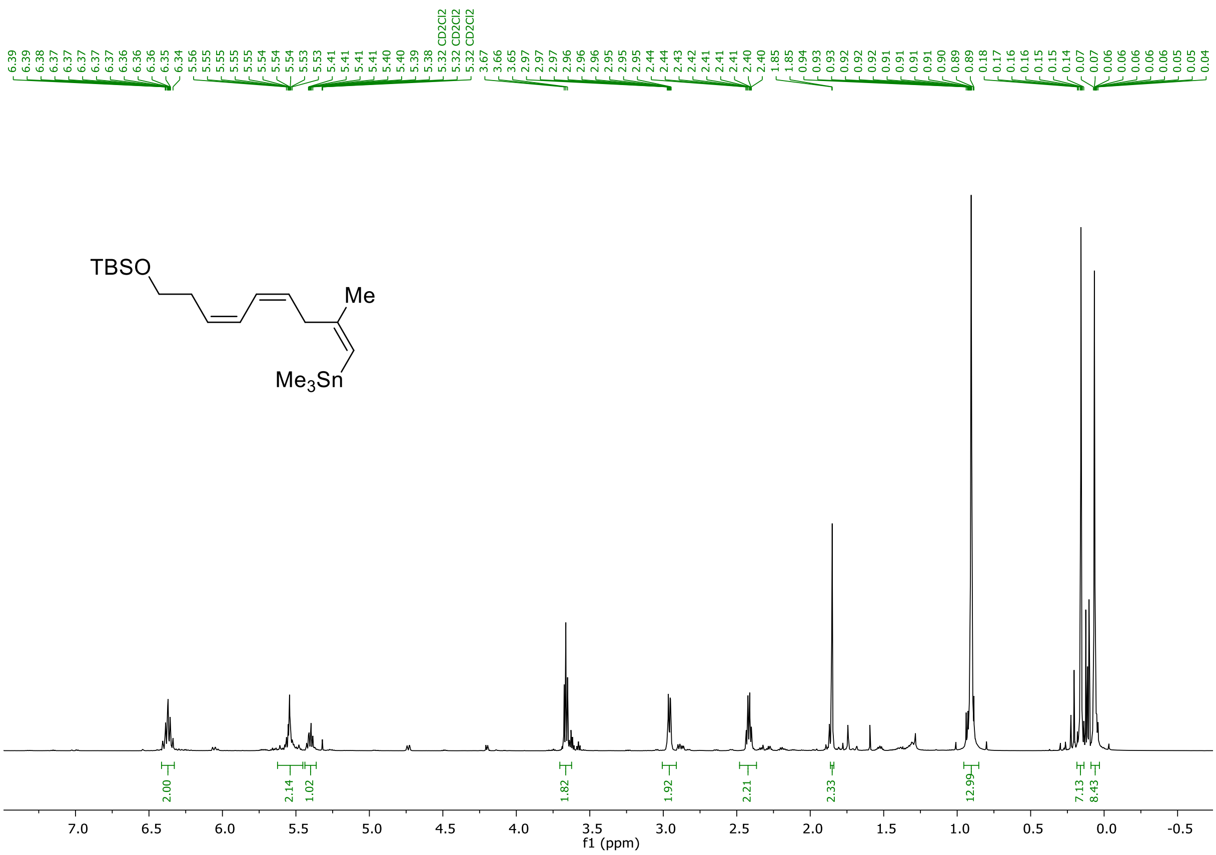

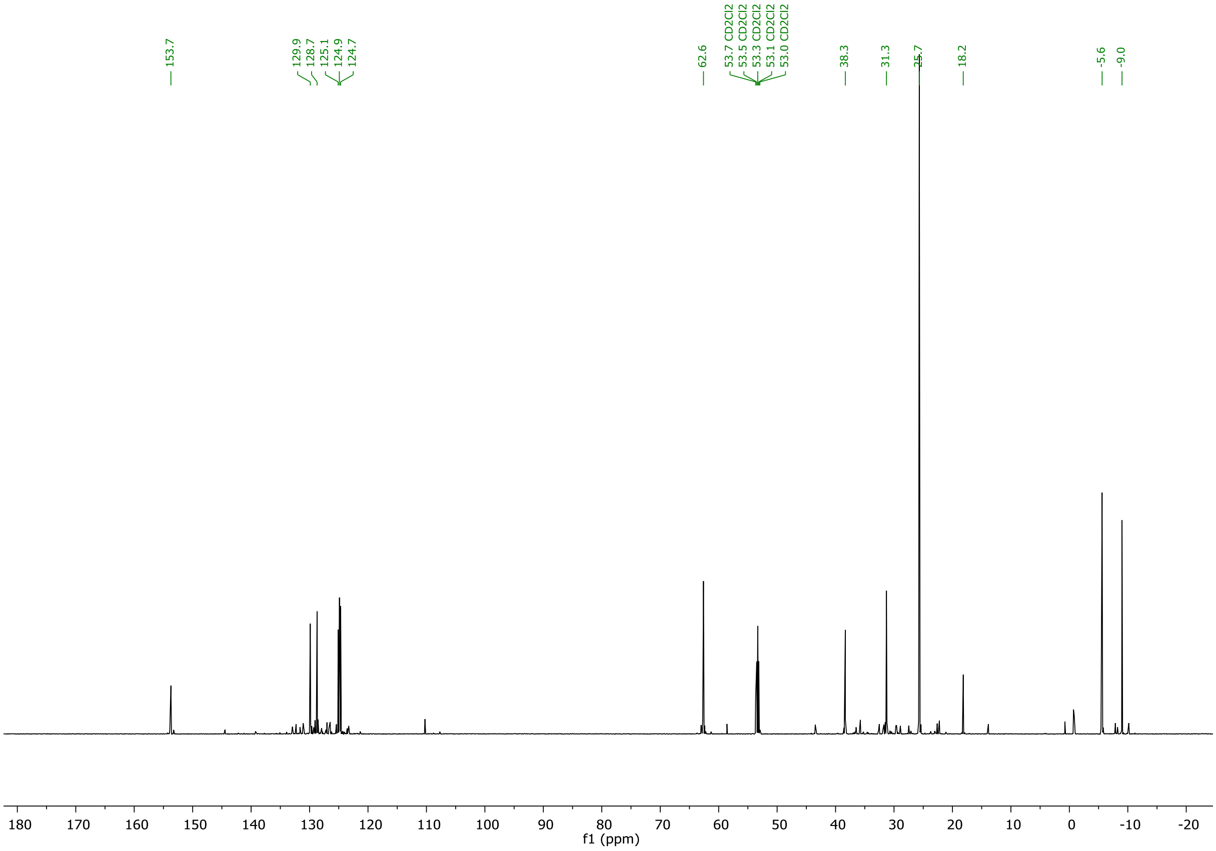

^{13}C NMR (101 MHz, CD_2Cl_2)

Aldehyde 36

^{1}H NMR (400 MHz, $CDCl_3$)

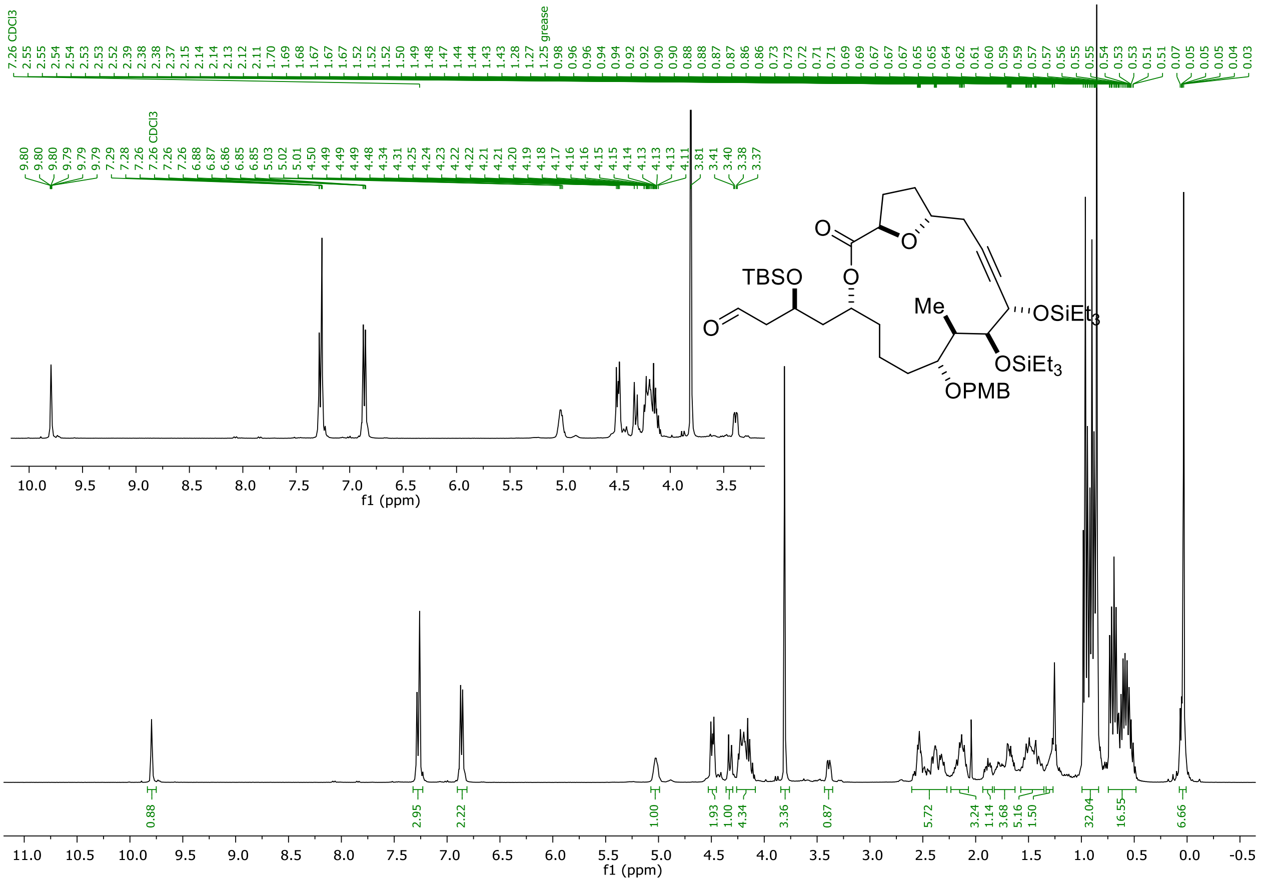

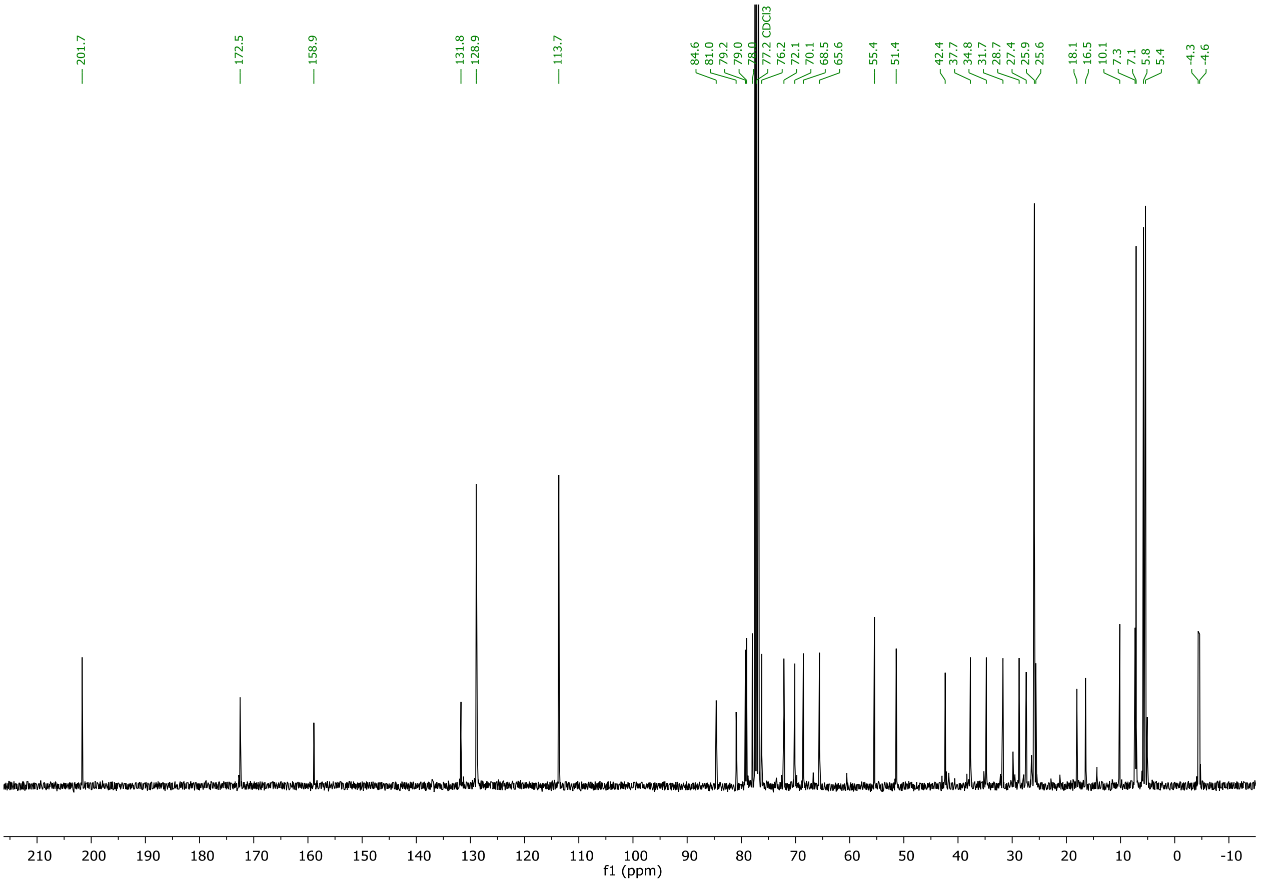

^{13}C NMR (101 MHz, $CDCl_3$)

Alkenyl Iodide 38

^1H NMR (600 MHz, $CDCl_3$)

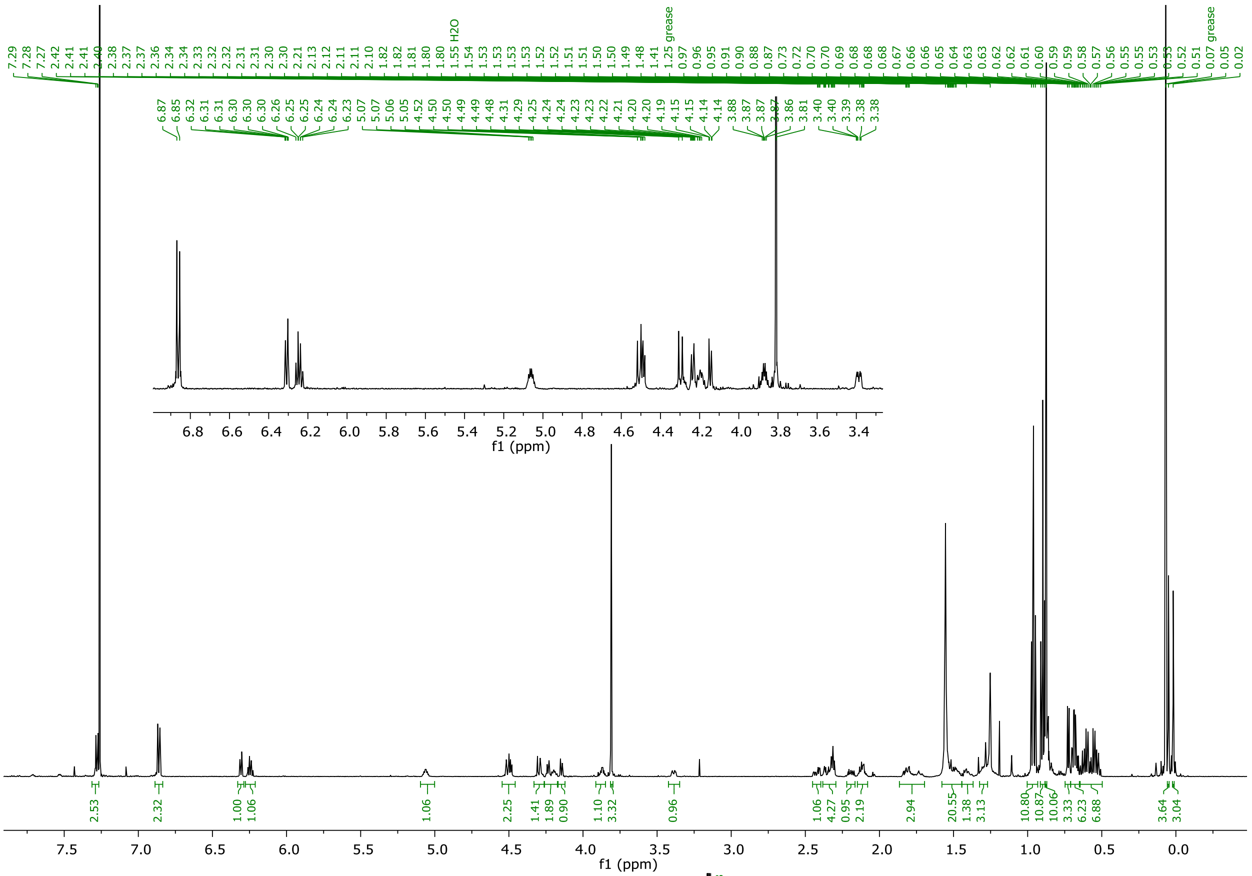

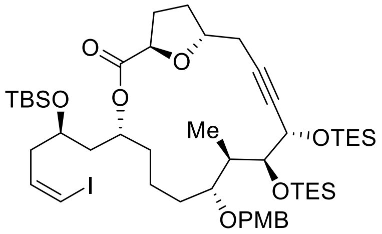

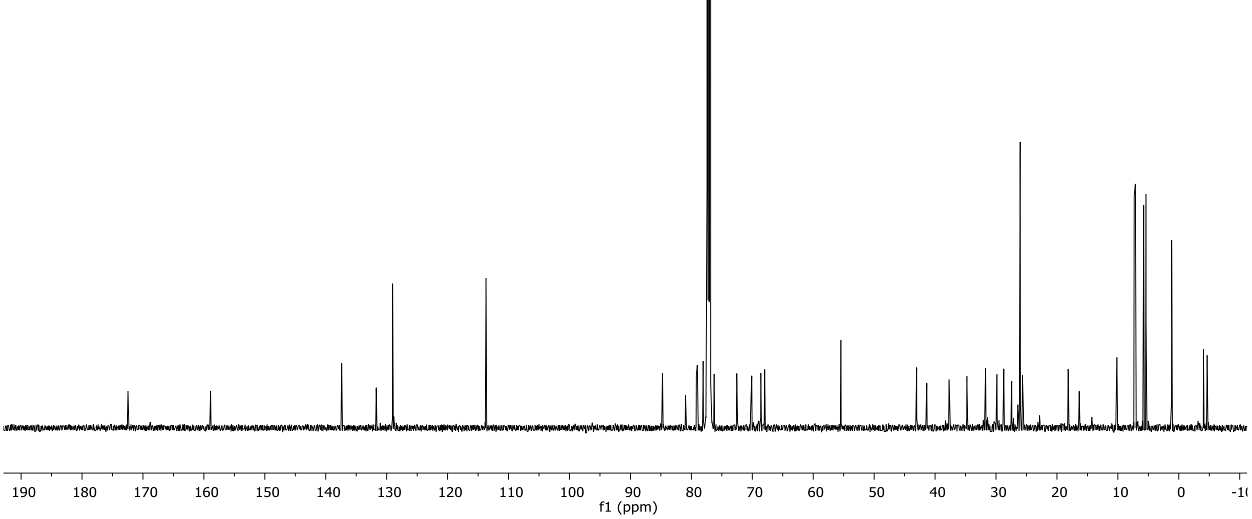

^{13}C NMR (151 MHz, $CDCl_3$)

Epoxide 37 (mixture of diastereomers) ^{1}H NMR (600 MHz, CD_2Cl_2)

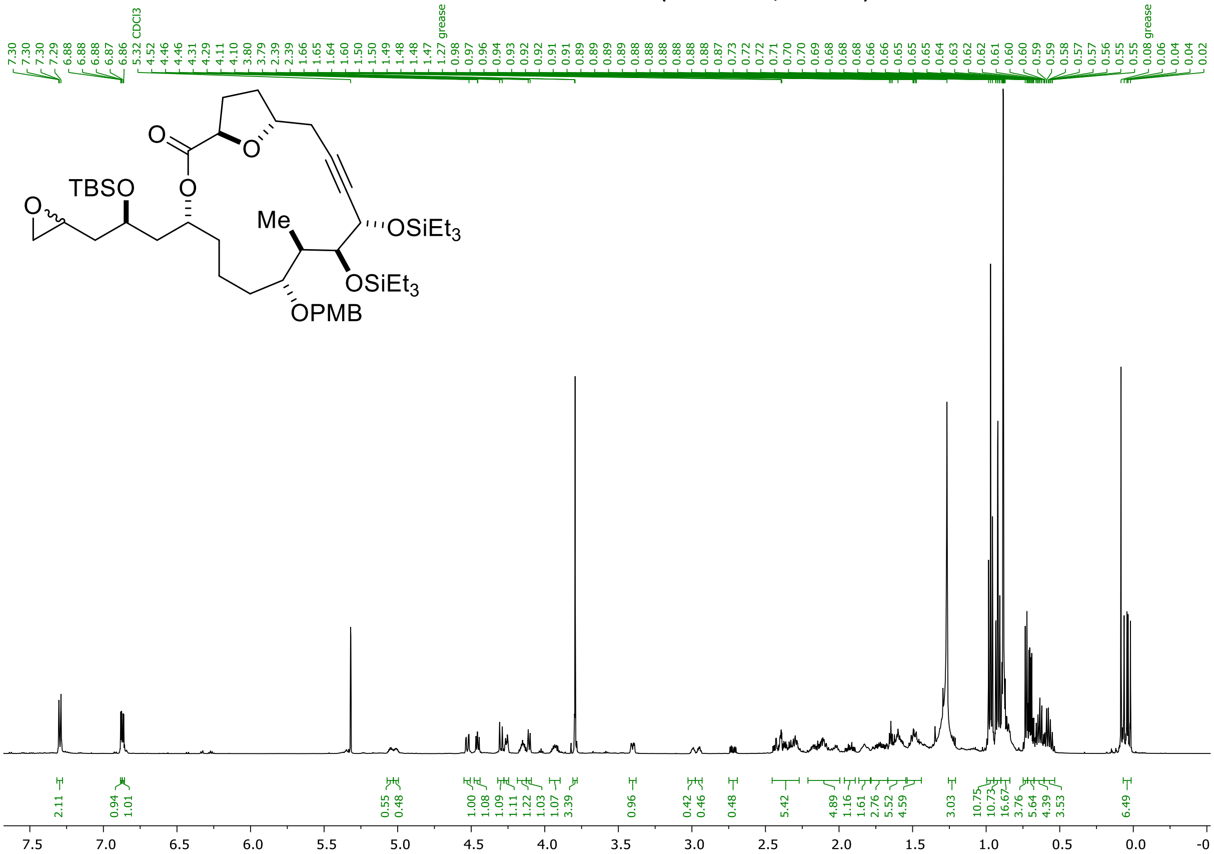

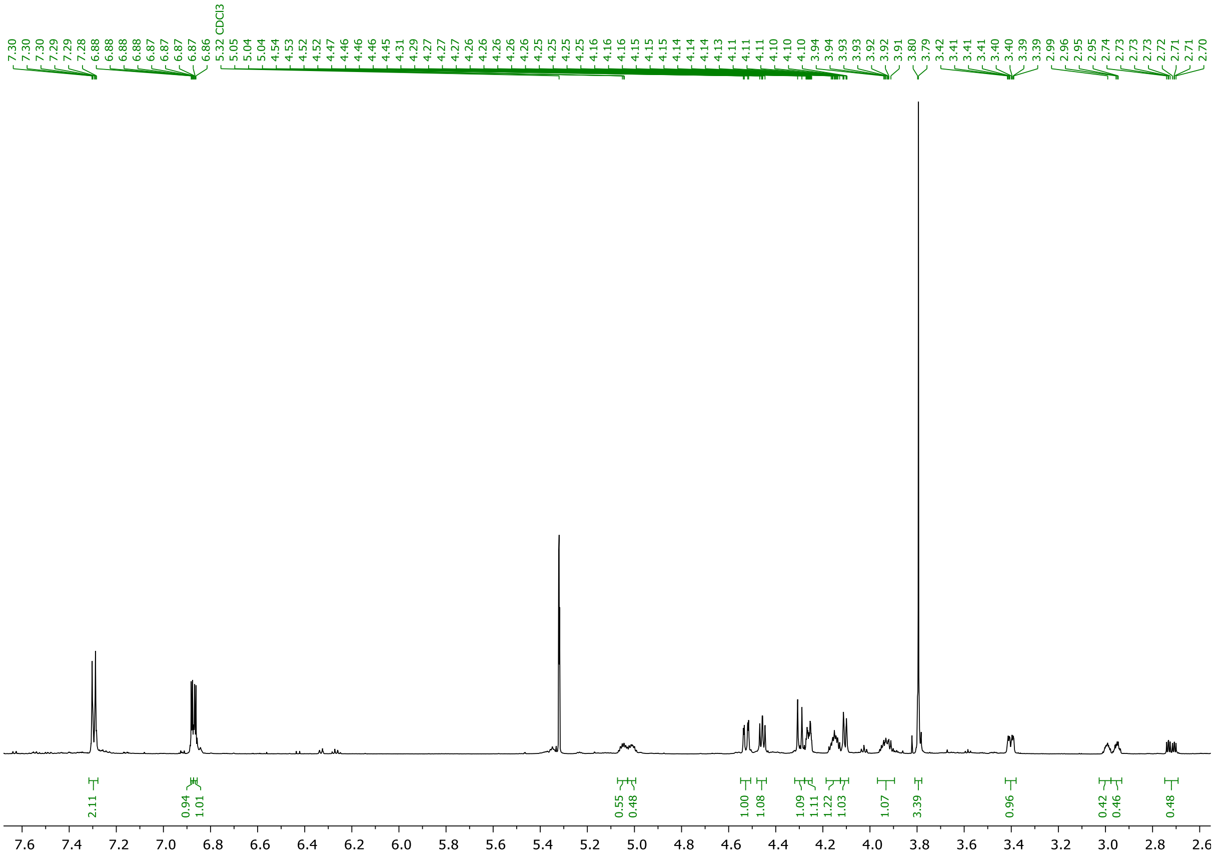

^{1}H NMR (600 MHz, CD_2Cl_2)

^1H NMR (600 MHz, CD_2Cl_2)

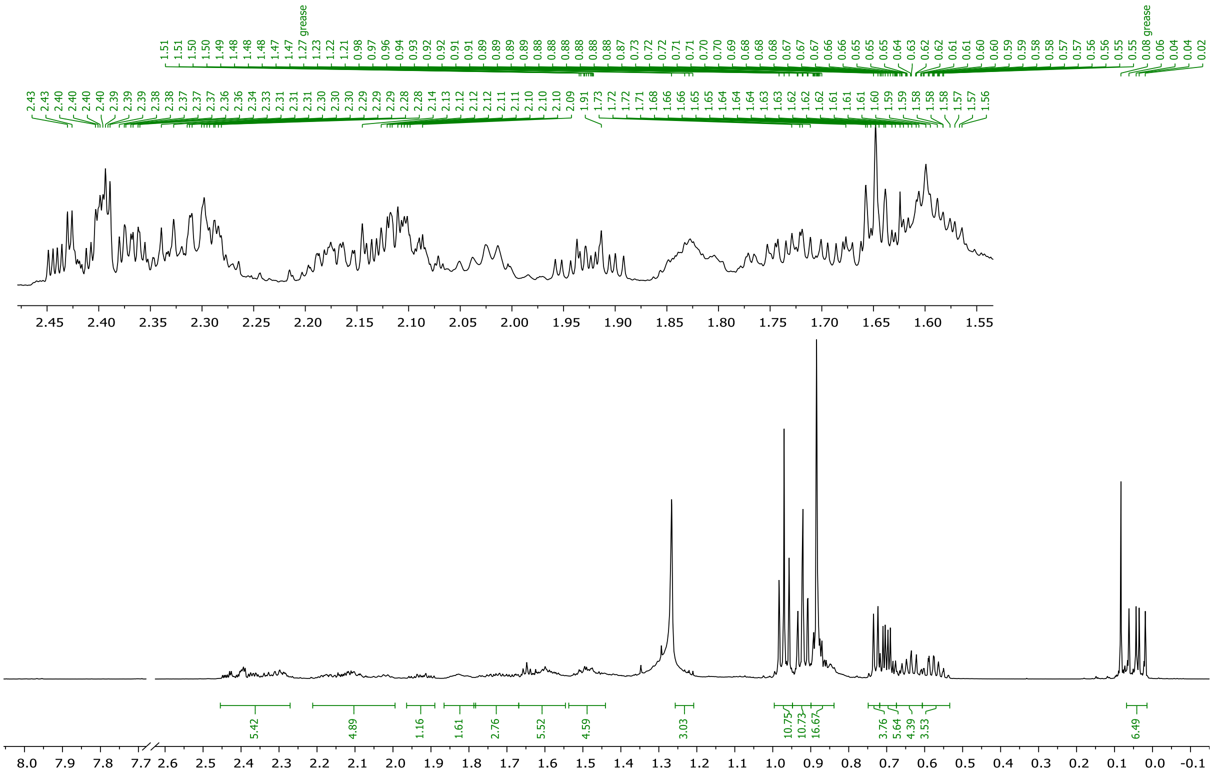

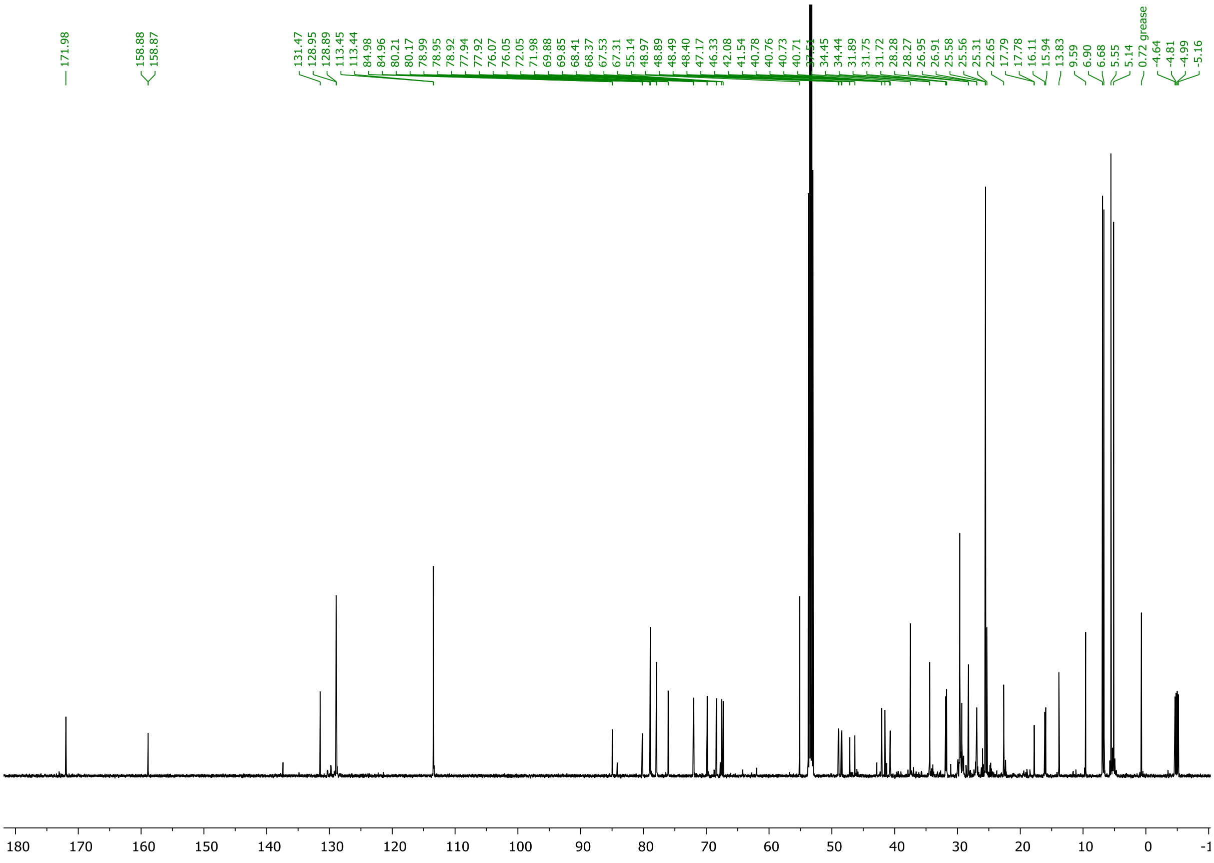

^{13}C NMR (151 MHz, CD_2Cl_2)

Alcohol 39

^{1}H NMR (400 MHz, $CDCl_3$)

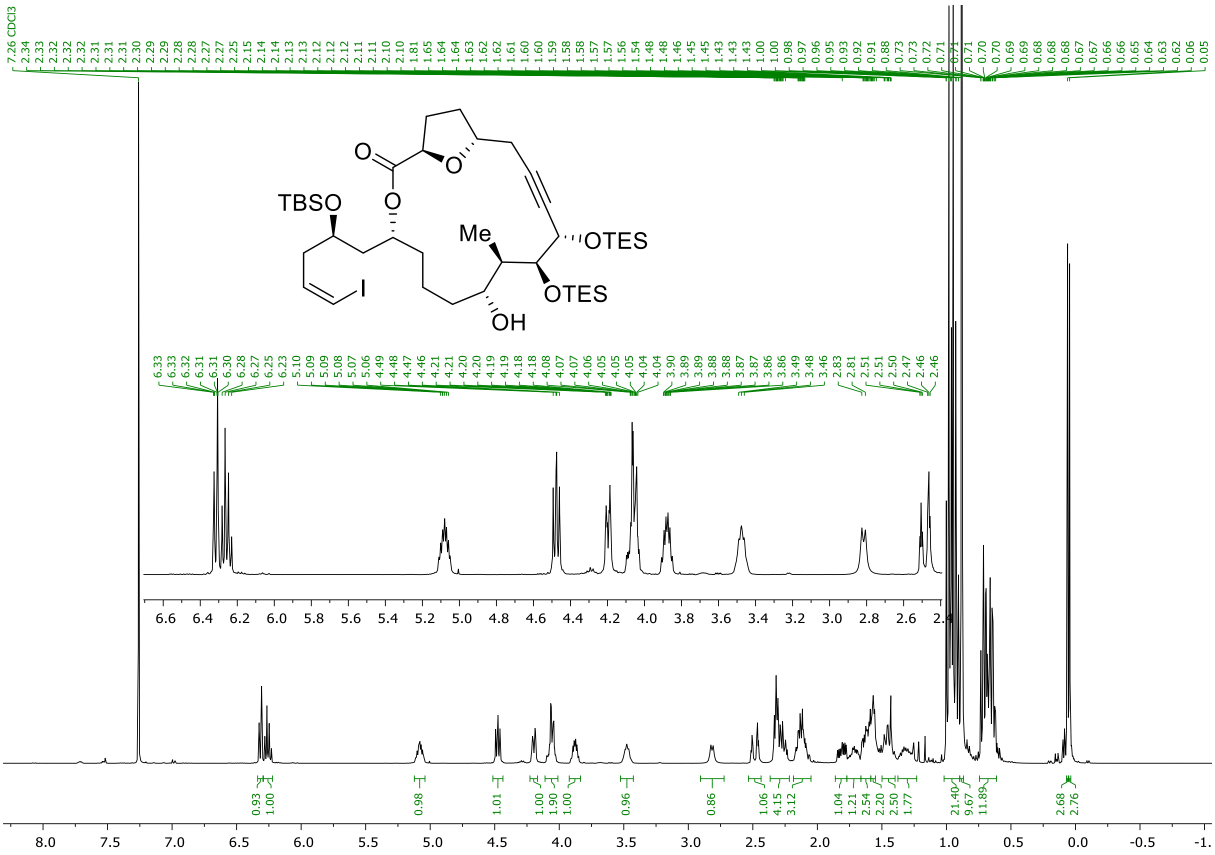

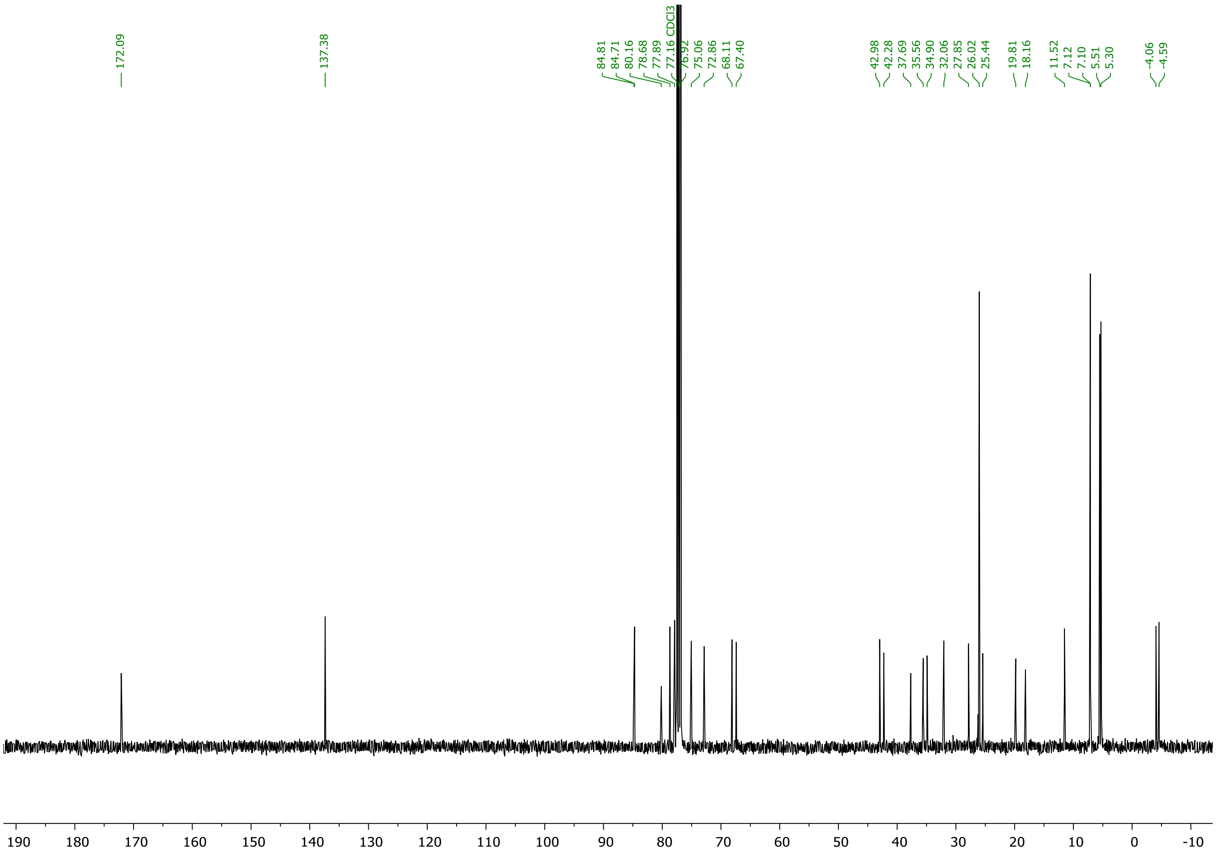

^{13}C NMR (101 MHz, $CDCl_3$)

Ketal 40

^{1}H NMR (600 MHz, $CDCl_3$)

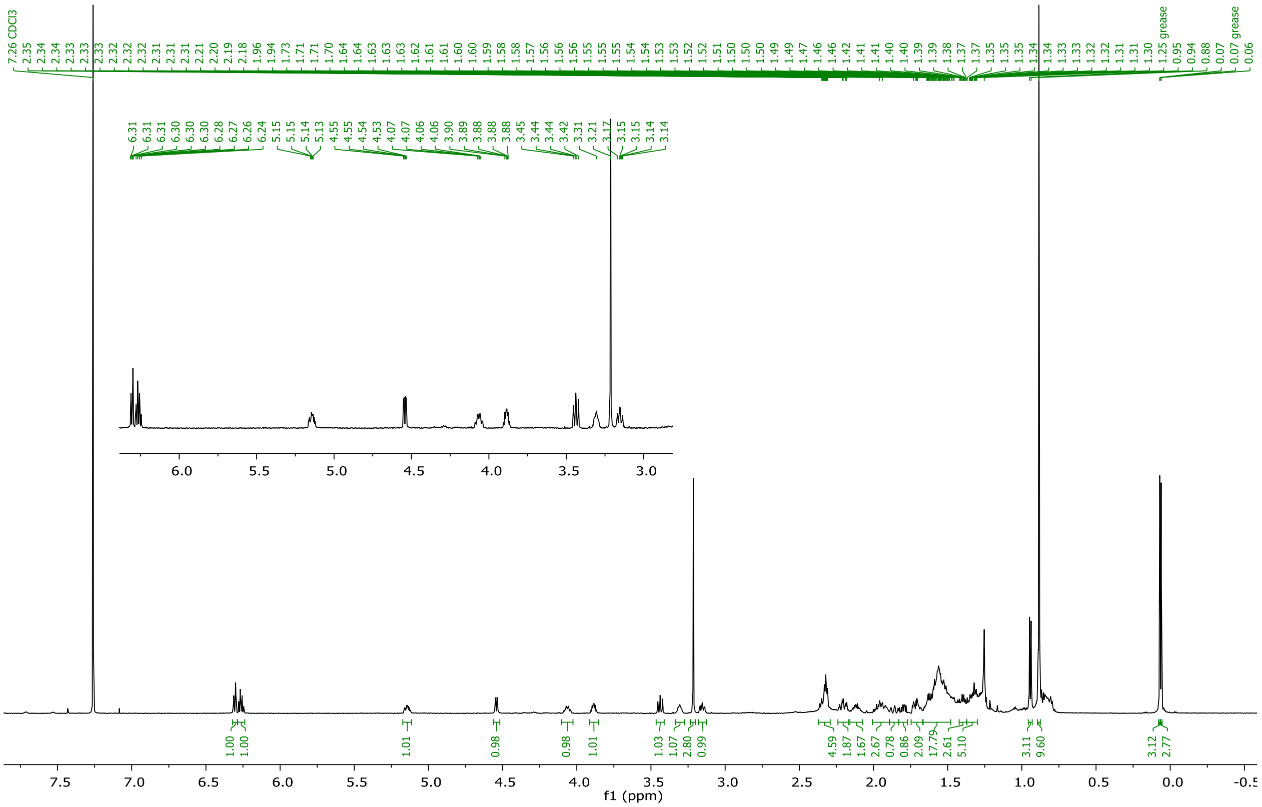

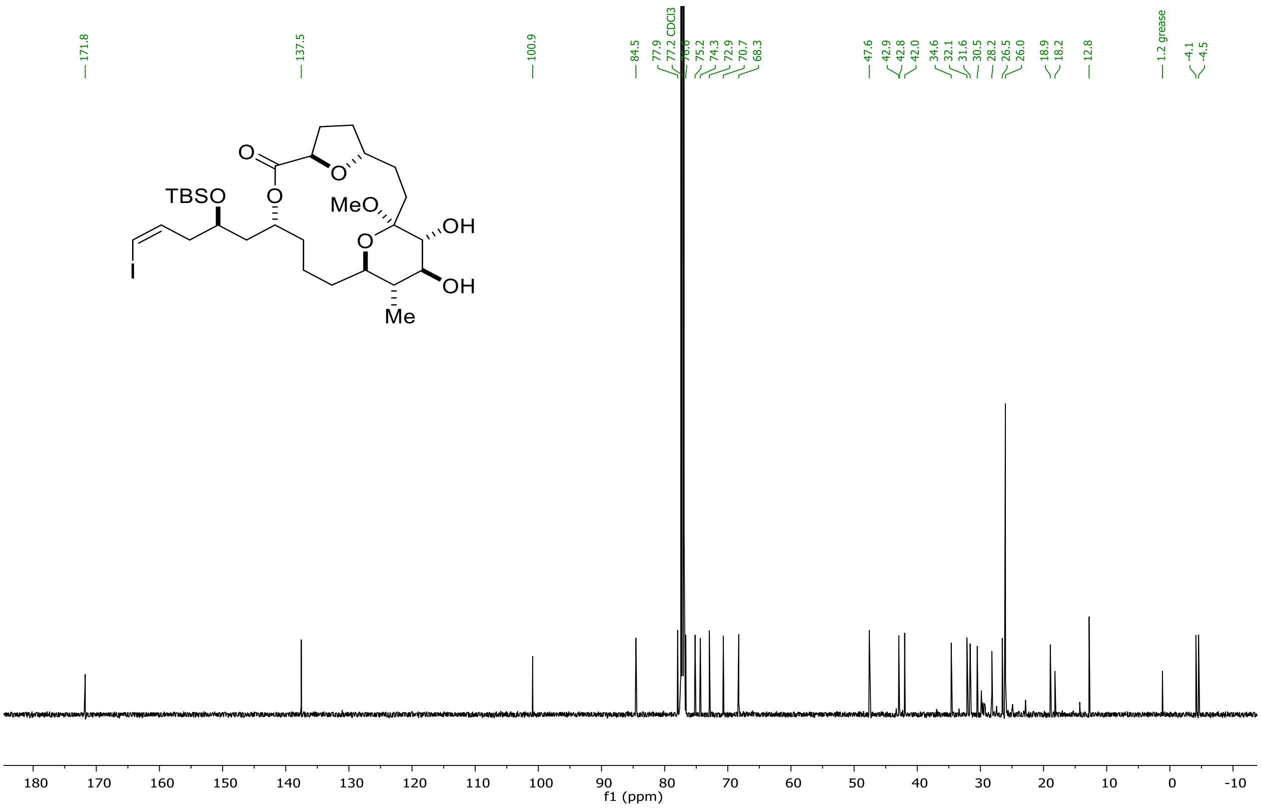

^{13}C NMR (151 MHz, $CDCl_3$)

Diene S12

^1H NMR (600 MHz, $CDCl_3$)

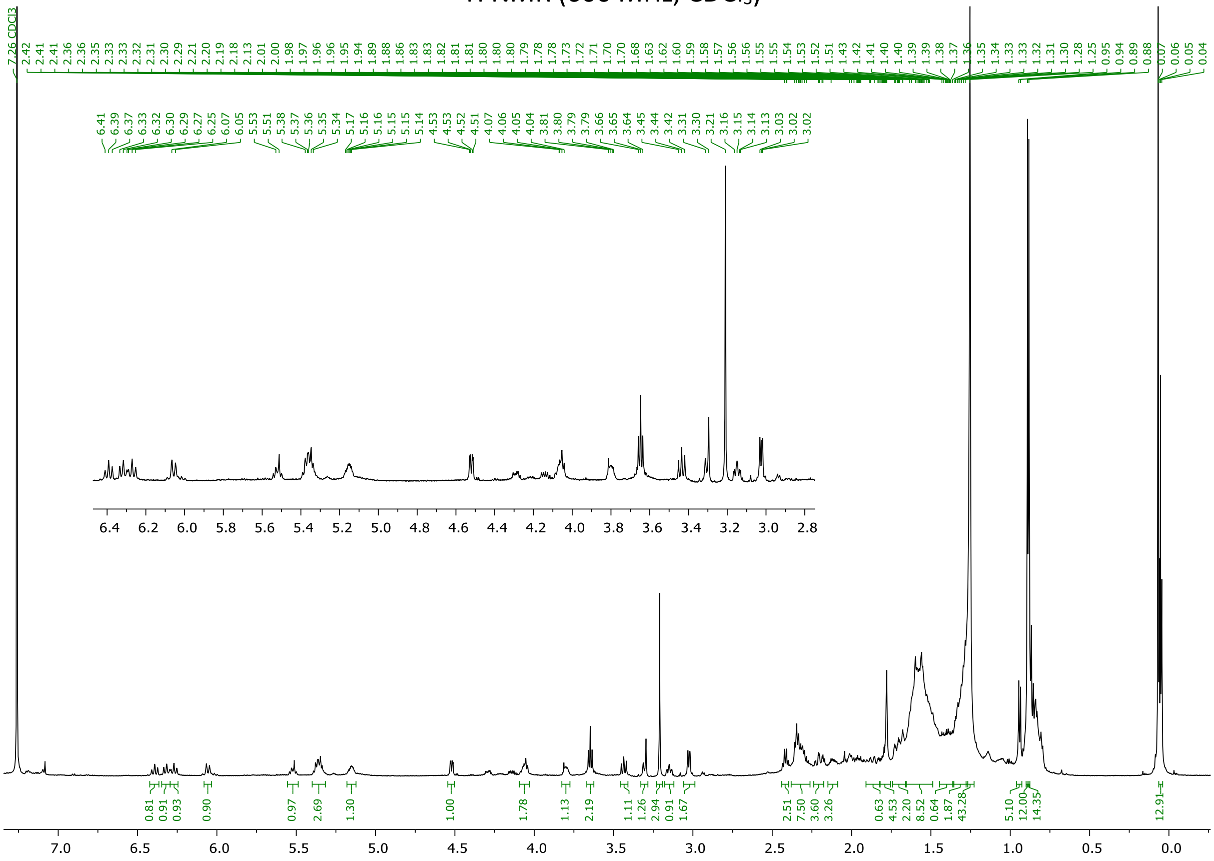

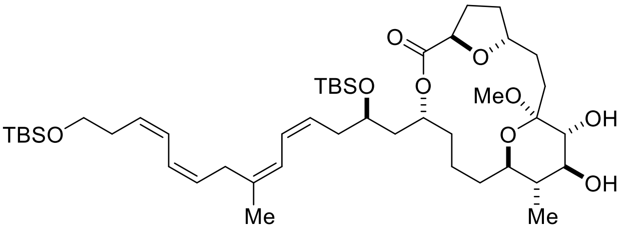

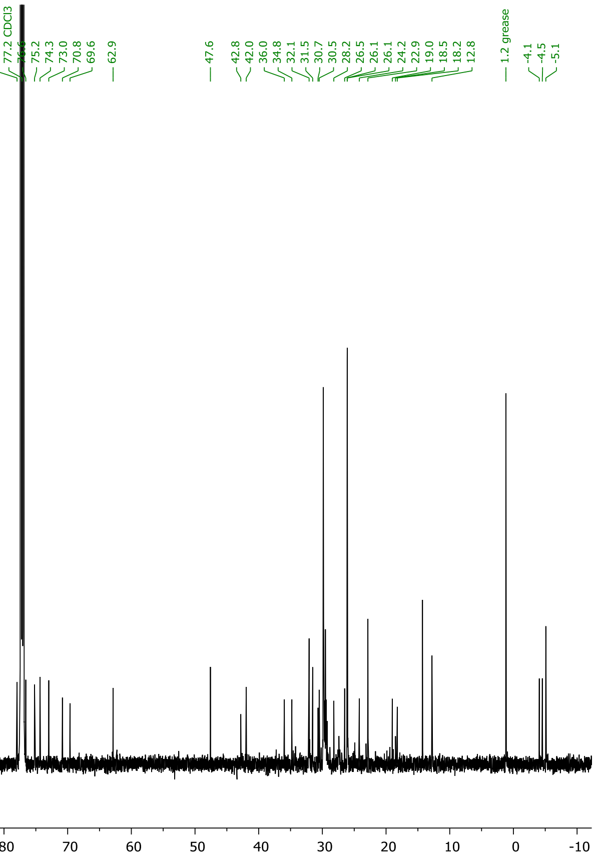

^{13}C NMR (151 MHz, $CDCl_3$)

Compound 2 (Formosalide B)

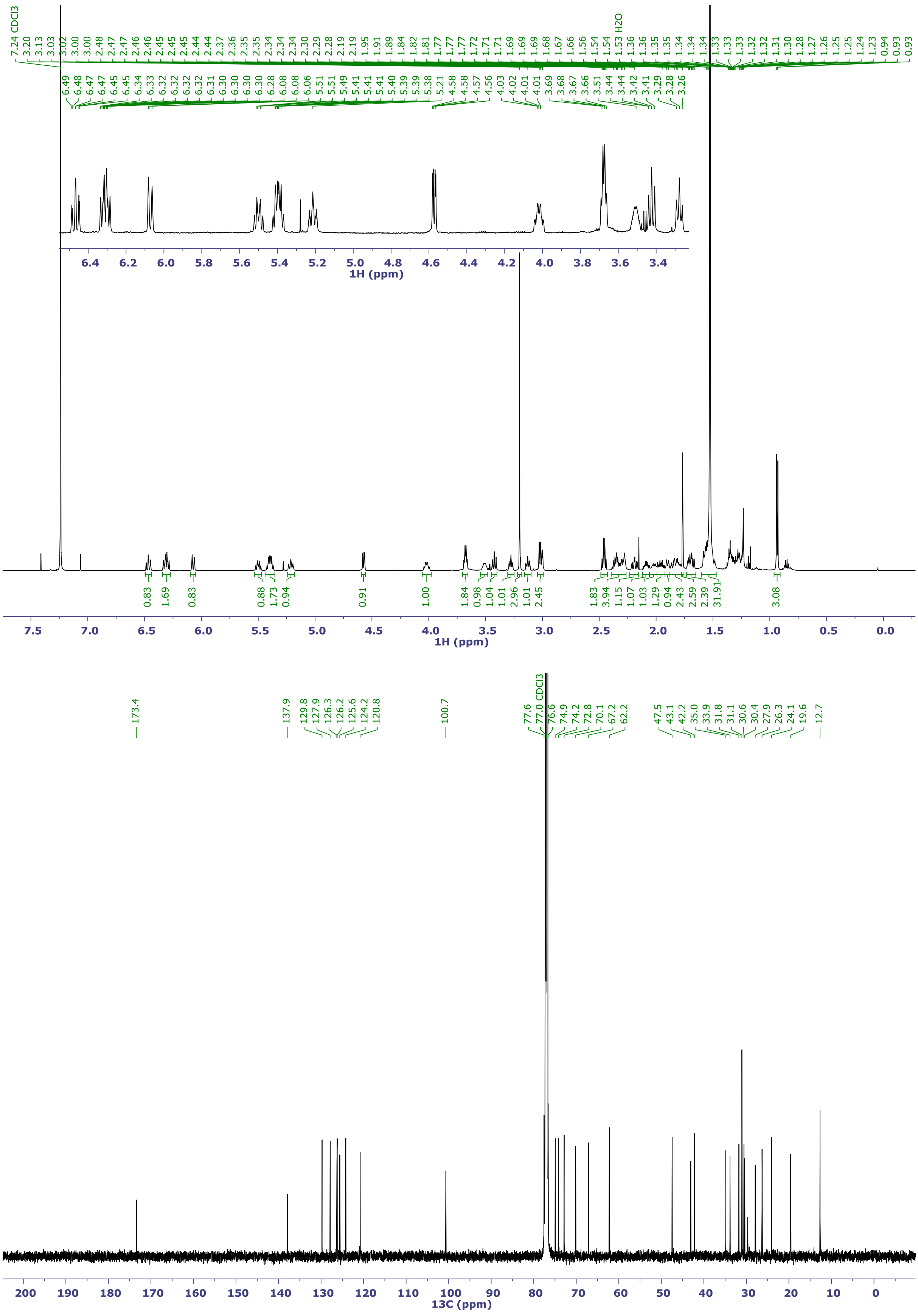

Graphical comparison of ^{13}C NMR spectra of synthetic **2** (black) and the simulated spectra of the literature shifts (red) of Formosalide B

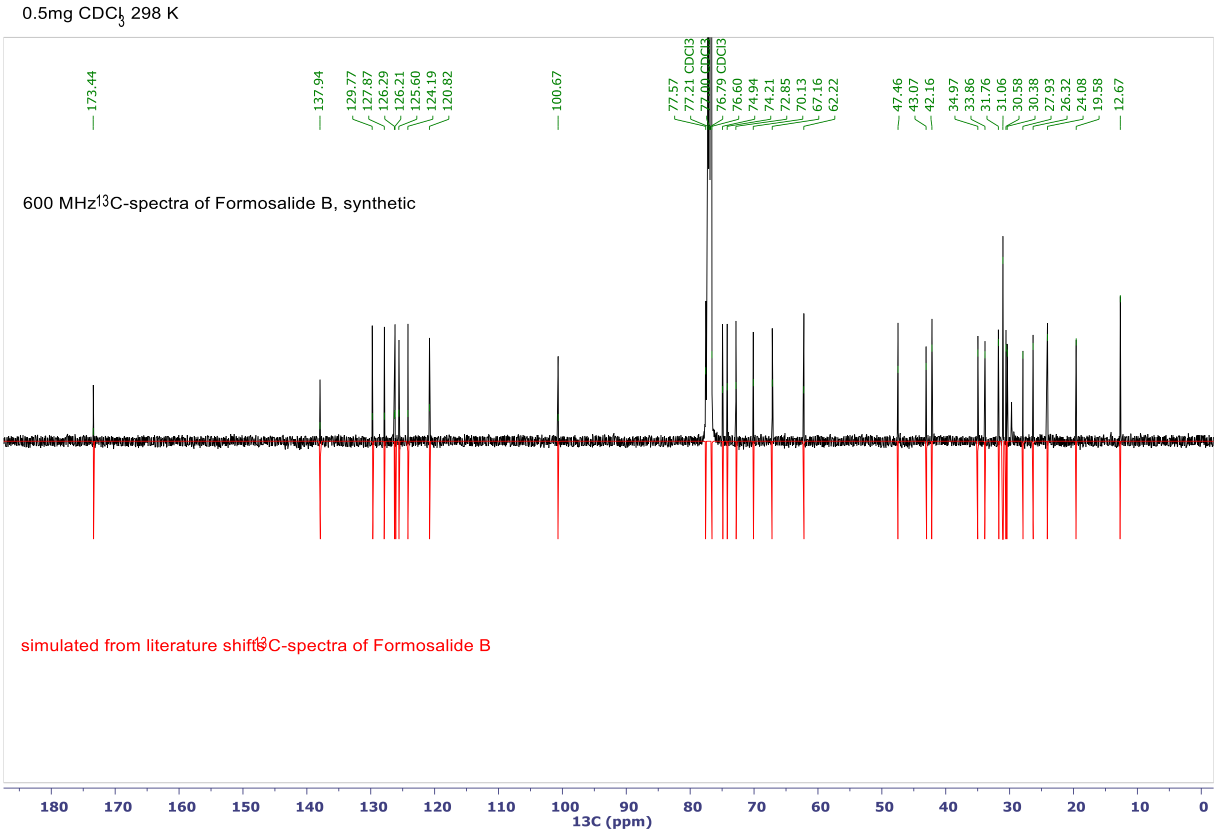

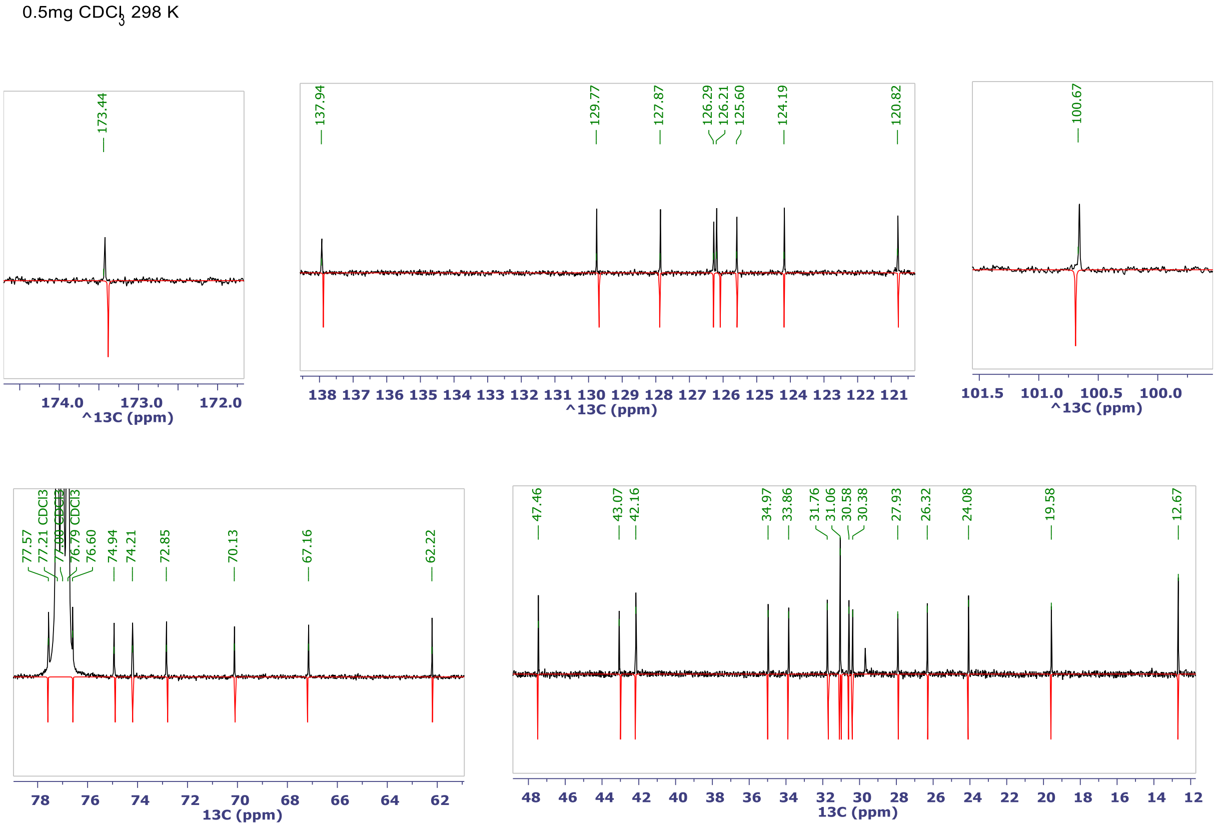

Compound 1 (Formosalide A)

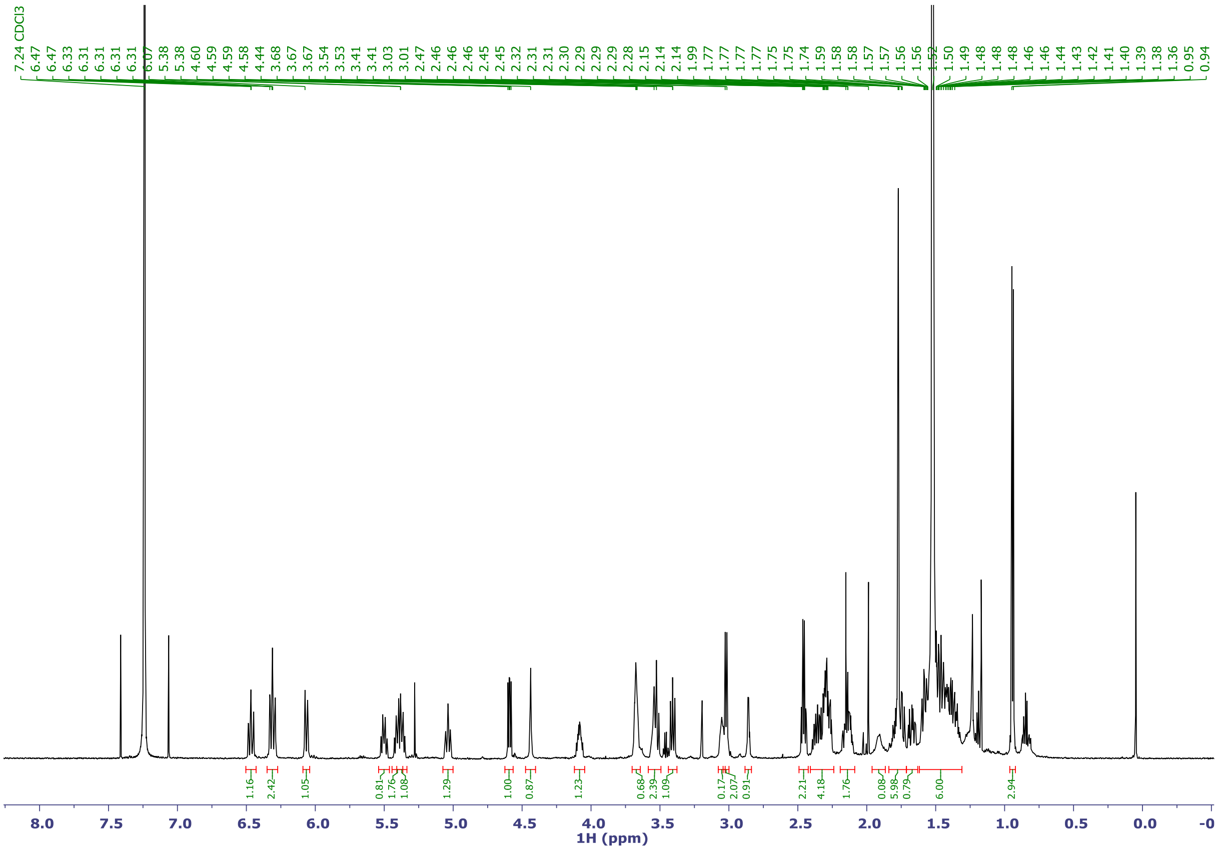

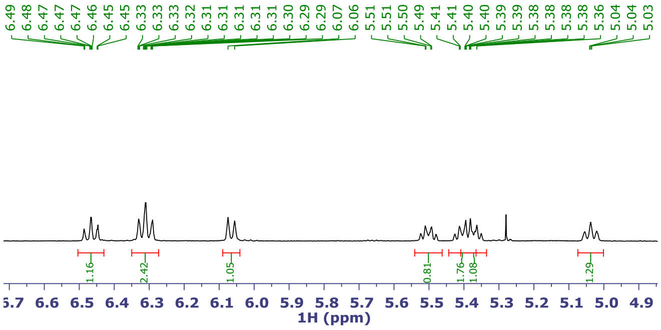

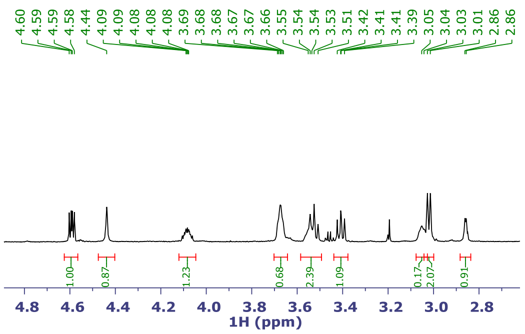

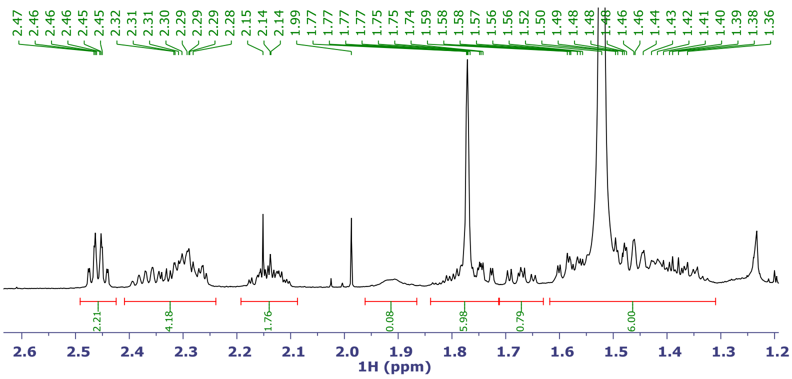

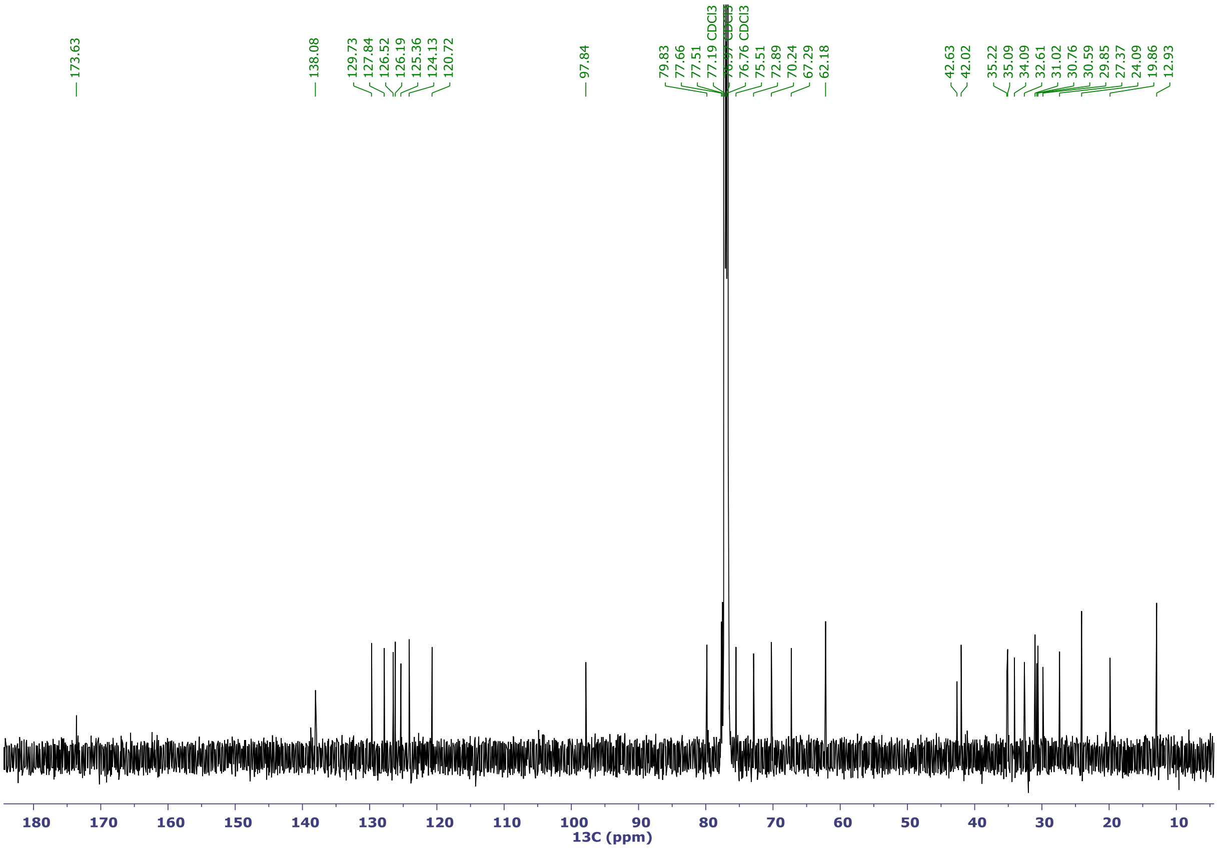

173.63
138.08
129.73
127.84
126.52
126.19
125.36
124.13
120.72
97.84
79.83
77.66
77.51
77.19 CDCl3
76.97 CDCl3
76.76 CDCl3
75.51
72.89
70.24
67.29
62.18
42.63
42.02
35.22
35.09
34.09
32.61
31.02
30.76
30.59
29.85
27.37
24.09
19.86
12.93
180 170 160 150 140 130 120 110 100 90 80 70 60 50 40 30 20 10
13C (ppm)

Graphical comparison of ^{13}C NMR spectra of synthetic **1** (black) and the simulated spectra of the literature shifts (red) of Formosalide A

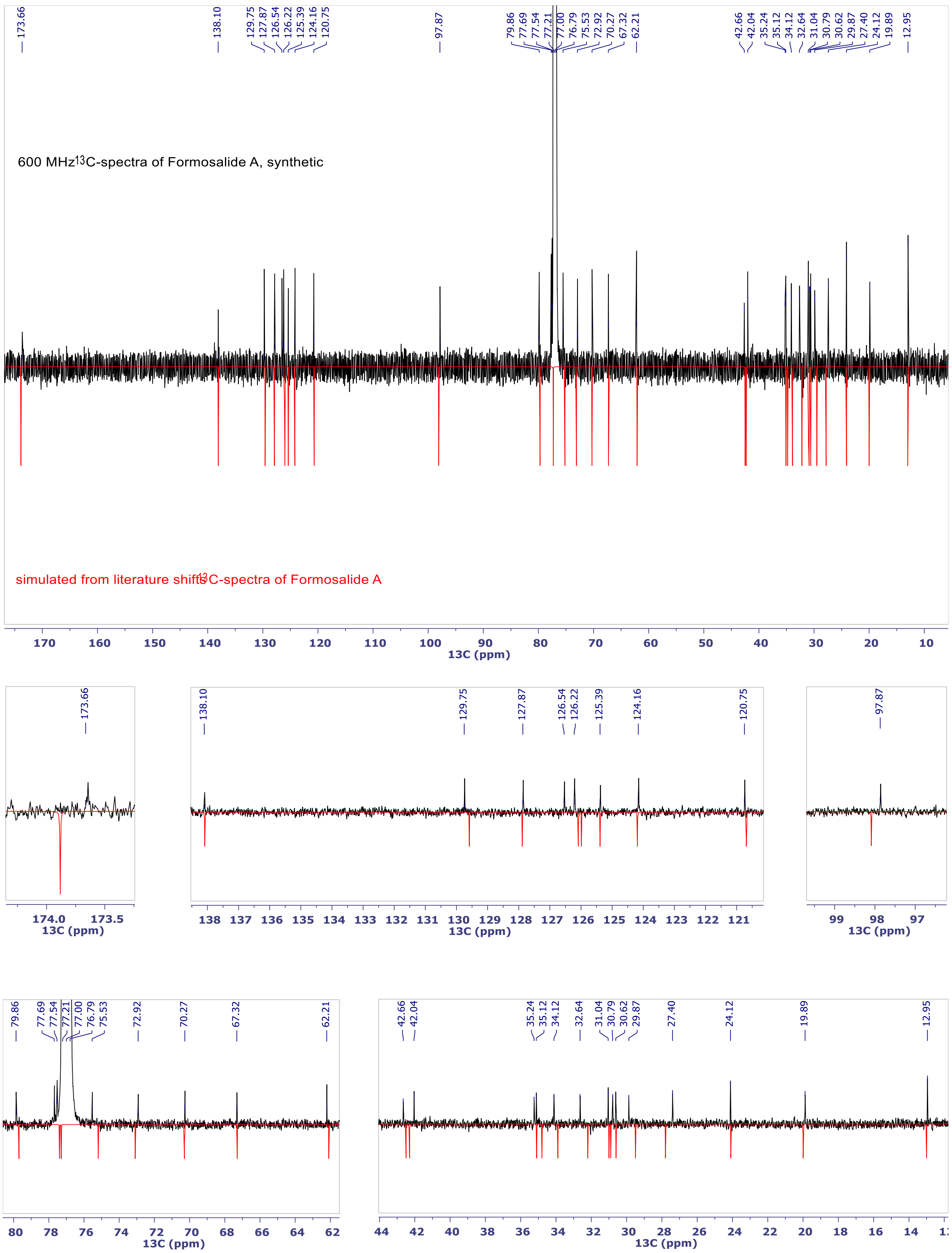

Supplement: Supplementary file 1 — Supplementary [file ANIE-60-446-s001.pdf]
