# Supplementary material for: The Formosalides: Structure Determination by Total Synthesis
Source: Angew Chem Int Ed Engl. 2020 Nov 3;60(1):446–54. doi: 10.1002/anie.202011472 (PMC7821135; doi:10.1002/anie.202011472)
Supplement: Supplementary file 2 — Supplementary [file ANIE-60-446-s002.pdf]

Angewandte Chemie
Eine Zeitschrift der Gesellschaft Deutscher Chemiker

Supporting Information

The Formosalides: Structure Determination by Total Synthesis

*Saskia Schulthoff[+], James Y. Hamilton[+], Marc Heinrich, Yonghoon Kwon, Conny Wirtz, and Alois Fürstner**

anie_202011472_sm_miscellaneous_information.pdf

Overview over the Panel of Possible Isomers

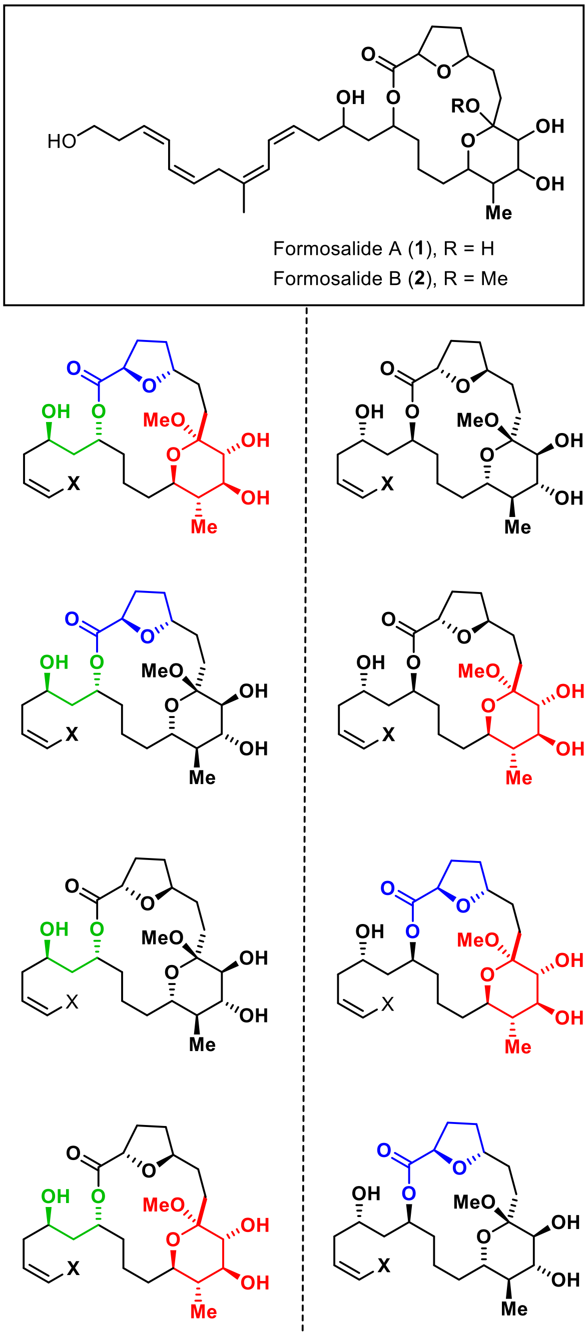

Scheme S-1. Structure of the formosalides: only the relative stereochemistry of the color-coded stereoclusters was determined by the isolation team, but their inter-relationships and the absolute configuration could not be established; therefore, eight isomers need to be considered.

Attempted Indirect Solution for Fragment Coupling via

Evans-Tishchenko Redox Esterification

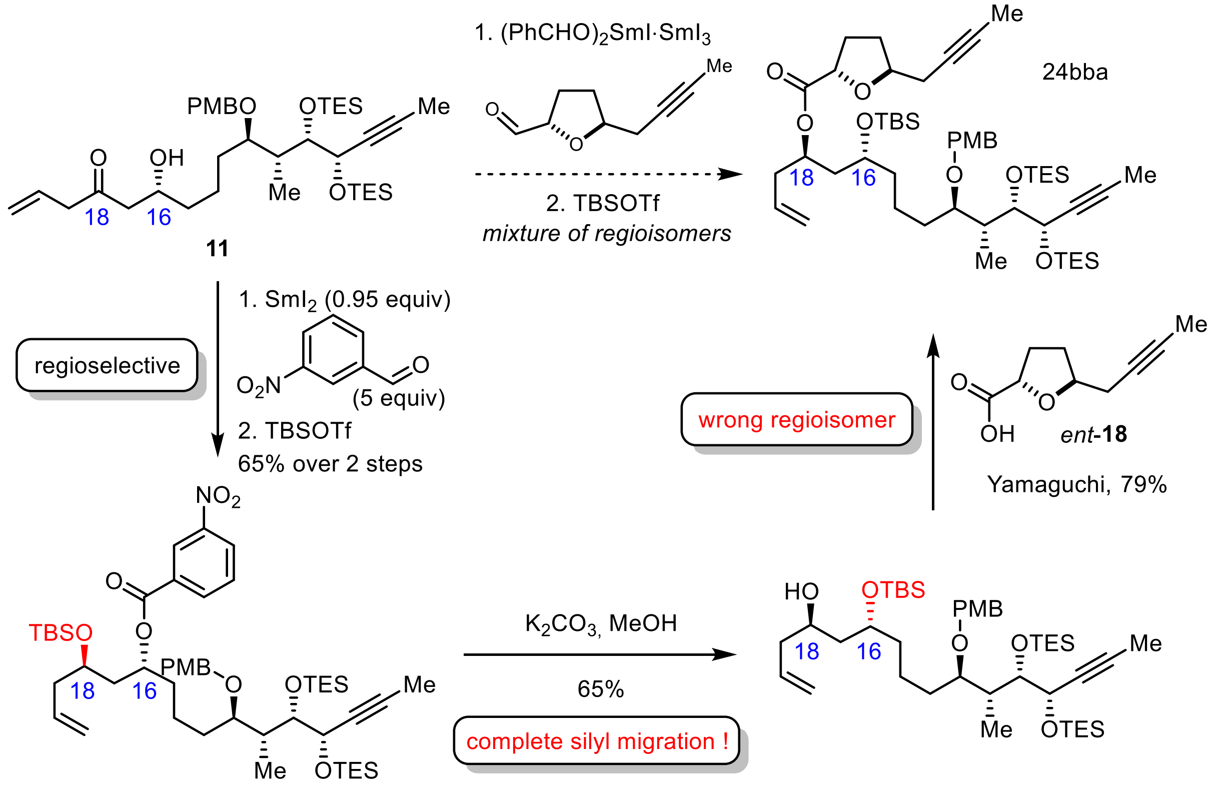

Scheme S-2. In contrast to the Evans-Tishchenko reactions using aldehyde **18** or *ent*-**18**, which invariably furnished product mixtures, the reaction with *p*-nitrobenzaldehyde proceeded selectively, providing the correct regioisomer. However, this favorable result was thwarted by the quantitative migration of the silyl group upon cleavage of the *p*-nitrobenzoate

General Experimental Methods

All reactions were carried out under Ar in flame-dried glassware unless water was used as solvent or it is otherwise noted. The following solvents and organic bases were purified by distillation over the drying agents indicated and were transferred under Ar: THF, Et_2O (Mg/anthracene); hexane, toluene (Na/K); Et_3N, diisopropylamine, diisopropylethylamine, 2,6-lutidine, HMPA, CH_2Cl_2, DMA, NMP (CaH_2); MeOH, EtOH, *i*-PrOH (Mg, stored over 3 Å MS). DMF, DMSO, 1,4-dioxane, MeCN and pyridine were dried by an adsorption solvent purification system based on molecular sieves. All other commercially available compounds (ABCR, Acros, Alfa Aesar, Aldrich, Fluka, STREM, TCI) were used as received unless otherwise noted.

The following compounds were prepared according to the cited literature: triisopropyl((2-methylhex-5-en-3-yn-2-yl)oxy)silane,[1] 5,5-dimethoxypentanal,[2-3] 1-iodopropoyne,[4] *O*-trimethylsilyl quinidine,[5] (*2R,5R*)-5-(but-2-yn-1-yl)tetrahydrofuran-2-carbaldehyde,[6-7] SmI_2,[8] complex **29**,[9] 5-((*tert*-butyldimethylsilyl)oxy)pent-2-yn-1-ol,[10] (*Z*)-1,4-diiodo-2-methylbut-1-ene,[11] TASF.[12]

Thin layer chromatography (TLC) was performed on Macherey-Nagel pre-coated plates (POLYGRAM® SIL/UV254). Detection was achieved under UV light (254 nm) and by staining with either acidic *p*-anisaldehyde, cerium ammonium molybdenate, or basic $KMnO_4$ solution.

Flash chromatography was performed with Merck silica gel 60 (40-63 µm pore size) using predistilled or HPLC-grade solvents. In some cases, fine Merck silica gel 60 (15-40 µm pore size) was necessary as indicated in the experimental procedures.

NMR-spectra were recorded on Bruker AV 300, AV 400, AV 500, AVIII 600 or AV600neo (the latter two both equipped with cryoprobes) spectrometers in the solvents indicated. Chemical shifts (δ) are reported in ppm relative to TMS; coupling constants (J) are given in Hz. Multiplets are indicated by the following abbreviations: s: singlet, d: doublet, t: triplet, q: quartet, p: pentet, h: hextet, hept: heptet, m: multiplet. The abbreviation br indicates a broad signal. ^{13}C spectra were recorded in {^1H}-decoupled manner and the values of the chemical shifts are rounded to one decimal point. Signal assignments were established using HSQC, HMBC, COSY, NOESY and other 2D experiments; numbering schemes as shown in the inserts.

IR spectra were recorded on Alpha Platinum ATR (Bruker) at ambient temperature, wavenumbers ($\tilde{\nu}$) are given in cm^{-1}.

Mass spectra were measured by the department for mass spectrometry at the Max-Planck-Institut für Kohlenforschung using the following devices: MS (EI): Finnigan MAT 8200 (70 eV), ESI-MS: Bruker ESQ3000, accurate mass determinations: Bruker APEX III FT-MS (7 T magnet) or MAT 95 (Finnigan). The

characteristic ion measured by high resolution mass spectrometry is given as the [M+Na$^+$]-adduct, unless otherwise noticed.

Optical rotations were measured with an A-Krüss Otronic Model P8000-t polarimeter at a wavelength of 589 nm. The values are given as specific optical rotation with exact temperature, concentration (c/(10 mg/mL)) and solvent.

LC-MS analyses were conducted on a Shimadzu LC-MS 2020 instrument (pumps LC-20AD, autosampler SIL-20AC, column oven CTO-20AC, diode array detector SPD-M20A, controller CBM-20A, ESI detector and software Labsolutions) with a ZORBAX Eclipse Plus column (C18 1.8 µm, 4.6 mm ID × 50 mm (Agilent)) or a YMC-ODS-A C18 column (S-5 µm, 120 Å, 4.6 mm ID × 150 mm). A binary gradient of MeCN or MeOH in water was used as eluent at a flow rate of 0.8 mL/min or 1.0 (4.6 mm ID). The oven temperature was kept at 35 °C and the detection wavelength at 250 nm; the conditions for each compound are specified below.

The Diastereomeric Southern Segments

General Procedure for Krische Propargylation. Triisopropyl((2-methylhex-5-en-3-yn-2-yl)oxy)silane (1.5 equiv.), 5,5-dimethoxypentanal (1 equiv.) and formic acid (1.5 equiv.) were added to a solution of [{Ir(cod)Cl}$_2$] (2.5 mol%), SEGPHOS (5.0 mol%) and Na_2SO_4 (1-3 equiv.) in THF (1.0 M). After stirring at 60 °C in an open flask attached to a condenser, the resulting mixture was filtered through a plug of silica, which was washed with *tert*-butyl methyl ether. The combined extracts were concentrated and the residue was purified by flash chromatography (hexane/EtOAc= 4:1) to afford the title compounds.

(5*S*,6*R*)-1,1-Dimethoxy-6,9-dimethyl-9-((triisopropylsilyl)oxy)dec-7-yn-5-ol (*ent*-4). According to the

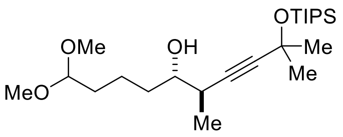

General Procedure, using 5,5-dimethoxypentanal (4.29 g, 29.3 mmol), (*R*)-SEGPHOS, Na_2SO_4 (1 equiv.); reaction time: 48 h. Yellow oil (7.39 g, 61%, dr > 20:1). $[\alpha]_D^{25}$ = +12.0 (c = 1.00, $CHCl_3$). ^{1}H NMR (400 MHz, $CDCl_3$) δ = 4.36 (t, *J* = 5.6 Hz, 1H), 3.42 – 3.34 (m, 1H), 3.31 (s, 6H), 2.57 – 2.47 (m, 1H), 1.72 (brs, 1H), 1.67 – 1.35 (m, 12H), 1.19 – 1.01 (m, 24H). ^{13}C NMR (101 MHz, $CDCl_3$) δ = 104.6, 89.3, 82.5, 74.4, 66.4, 52.8, 34.9, 33.8, 33.7, 33.4, 32.6, 21.1, 18.4, 17.2, 13.1. IR (film): $\tilde{\nu}$ = 3480, 2942, 2865, 1462, 1377, 1241, 1160, 1127, 1048, 919, 882, 745, 679 cm^{-1}. MS (ESIpos) *m/z* (%): 437.3 (100 (M+Na)). HRMS (ESI): *m/z* calcd for $C_{23}H_{46}O_4SiNa$ [M+Na]$^+$: 437.3058, found: 437.3055.

(5*R*,6*S*)-1,1-Dimethoxy-6,9-dimethyl-9-((triisopropylsilyl)oxy)dec-7-yn-5-ol (4). According to the

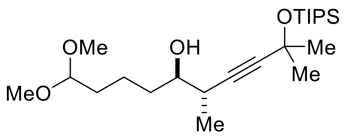

General Procedure, using 5,5-dimethoxypentanal (4.89 g, 33.5 mmol), (*S*)-SEGPHOS and Na_2SO_4 (3 equiv.); reaction time: 17.5 h. Yellow oil

(7.8 g, 56%, *ee* > 95%, dr > 20:1). The *ee* was determined by Mosher ester analysis.

Mosher Ester S1 Derived from Alcohol 4. Et_3N (11 µL, 80 µmol) and DMAP (0.3 mg, 3 µmol) were added to a solution of alcohol **4** (11.5 mg, 27.7 µmol) in CH_2Cl_2 (1.8 mL) followed by (*S*)-(–)-*α*-methoxy-*α*-trifluoromethyl-phenylacetyl chloride ((*S*)-MTPA-Cl) (8.0 µL, 43 µmol). The mixture was stirred at ambient temperature for 17 h before it was diluted with CH_2Cl_2 (2 mL) and saturated $NaHCO_3$ (2 mL). The aqueous phase was separated and extracted with CH_2Cl_2 (3 × 2 mL). The combined organic phases were dried over Na_2SO_4, filtered and concentrated. The residue was purified by flash chromatography (hexane/EtOAc = 10:1) to give the corresponding (*R)*-**S1** (15.3 mg, 87%), which analyzed as follows: $[\alpha]_D^{20}$ = +250 (c = 0.10, $CHCl_3$). ^1H NMR (400 MHz, $CDCl_3$) δ = 7.61 – 7.54 (m, 2H), 7.44 – 7.36 (m, 3H), 5.07 (td, *J* = 6.4, 4.0 Hz, 1H), 4.25 (t, *J* = 5.7 Hz, 1H), 3.58 (d, *J* = 1.3 Hz, 3H), 3.28 (s, 3H), 3.27 (s, 3H), 2.81 (qd, *J* = 7.1, 4.0 Hz, 1H), 1.73 – 1.63 (m, 2H), 1.61 – 1.46 (m, 2H), 1.45 (s, 6H), 1.32 – 1.18 (m, 2H), 1.18 – 1.07 (m, 6H), 1.09 – 1.03 (m, 18H). IR (neat): 2943, 2866, 1746, 1462, 1378, 1359, 1242, 1164, 1125, 1051, 1017, 995, 918, 882, 804, 765, 717, 681 cm^{-1}. MS (ESIpos) *m/z* (%): 653.3 (100 (M+Na)). HRMS (ESI): *m/z* calcd for $C_{11}H_{53}F_3O_6SiNa$ $[M+Na]^+$: 653.3256, found: 653.3452.

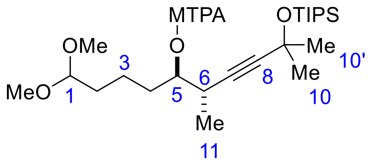

The corresponding Mosher ester (*S)*-**S1** (15.7 mg, 89%) was prepared analogously: $[\alpha]_D^{20}$ = +202 (c = 0.10, $CHCl_3$). ^1H NMR (400 MHz, $CDCl_3$) δ = 7.60 – 7.52 (m, 2H), 7.44 – 7.36 (m, 3H), 5.03 (ddd, *J* = 7.3, 5.6, 3.5 Hz, 1H), 4.31 (t, *J* = 5.7 Hz, 1H), 3.55 (d, *J* = 1.2 Hz, 3H), 3.30 (s, 6H), 2.79 (qd, *J* = 7.1, 3.5 Hz, 1H), 1.81 – 1.70 (m, 2H), 1.67 – 1.50 (m, 2H), 1.45 (s, 3H), 1.45 (s, 3H), 1.44 – 1.30 (m, 2H), 1.15 – 1.00 (m, 21H), 1.00 (d, *J* = 7.1 Hz, 3H). IR (neat): 2943, 2866, 1747, 1463, 1378, 1359, 1241, 1164, 1125, 1052, 1017, 994, 919, 882, 765, 718, 681 cm^{-1}. MS (ESIpos) *m/z* (%): 653.3 (100 (M+Na)). HRMS (ESI): *m/z* calcd for $C_{11}H_{53}F_3O_6SiNa$ $[M+Na]^+$: 653.3256, found: 653.3452.

Table S-1. Mosher ester analysis for product **4**; arbitrary numbering scheme as shown in the insert

**Assignment**	**4 [ppm]**	**(*S*)-S1 [ppm]**	**(*R*)-S1 [ppm]**	**Δ (δ(*S*–*R*)) [ppm]**
1	4.36	4.31	4.25	+0.06
2	1.60	1.60	1.56	+0.04
3	1.51	1.37	1.25	+0.12
4	1.48	1.75	1.68	+0.07
5	3.38	5.03	5.07	+0.04
6	2.52	2.79	2.81	–0.02
10,10'	1.49	1.45	1.45	+0.00
11	1.16	1.00	1.12	–0.12

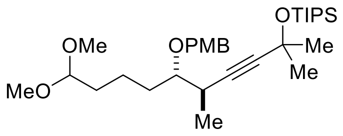

PMB-ether *ent*-S2. A solution of alcohol ***ent*-4** (7.37 g, 17.8 mmol) in DMF (27 mL) was added dropwise to a stirred solution of sodium hydride (469 mg, 19.5 mmol) in DMF (150 mL) at 0 °C. After stirring at 0 °C for 1 h, 4-methoxybenzyl chloride (2.65 mL, 19.5 mmol) and tetra-*n*-butylammonium iodide (0.656 g, 1.78 mmol) were added. The resulting mixture was warmed to room temperature and stirred for 24 h; at this point, additional 4-methoxybenzyl chloride (0.96 mL, 7.11 mmol) was introduced and stirring was continued for another 24 h. The reaction was quenched by careful addition of H_2O (15 mL) and the mixture was diluted with *tert*-butyl methyl ether (200 mL). The organic layer was separated and washed with H_2O (2 × 100 mL), dried over $MgSO_4$, and concentrated. The residue was purified by flash chromatography (CH_2Cl_2/EtOAc = 99:1 to 19:1) to afford the title compound as a colorless oil (8.02 g, 84%). $[\alpha]_D^{25}$ = −8.7 (c = 1.00, $CHCl_3$). ^1H NMR (400 MHz, $CDCl_3$) δ = 7.26 (d, *J* = 8.6 Hz, 2H), 6.87 (d, *J* = 8.6 Hz, 2H), 4.53 (d, *J* = 11.2 Hz, 1H), 4.39 (d, *J* = 11.2 Hz, 1H), 4.33 (t, *J* = 5.5 Hz, 1H), 3.80 (s, 3H), 3.40 – 3.34 (m, 1H), 3.30 (s, 6H), 2.82 (qd, *J* = 7.0, 4.6 Hz, 1H), 1.69 – 1.44 (m, 11H), 1.39 – 1.29 (m, 1H), 1.20 – 1.04 (m, 24H). ^{13}C NMR (101 MHz, $CDCl_3$) δ = 159.3, 130.9, 129.5, 113.8, 104.6, 87.2, 84.1, 81.0, 71.6, 66.4, 55.4, 52.8, 52.7, 33.70, 33.68, 32.7, 30.5, 28.9, 21.4, 18.5, 14.6, 13.1. IR (neat): 2941, 2864, 1613, 1513, 1462, 1376, 1358, 1301, 1245, 1160, 1037, 882, 821, 747, 680, 571, 514 cm^{-1}. MS (ESIpos) *m/z* (%): 557.4 (100 (M+Na)). HRMS (ESI): *m/z* calcd for $C_{31}H_{54}O_5SiNa$ $[M+Na]^+$: 557.3633, found: 557.3631.

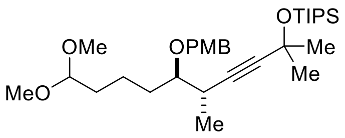

PMB-ether S2. A solution of alcohol **4** (7.80 g, 18.8 mmol) in DMF (45 mL) was added dropwise to a stirred solution of sodium hydride (0.90 g, 38 mmol) in DMF (18 mL) at 0 °C. After stirring at 0 °C for 1 h, 4-methoxybenzyl chloride (4.0 mL, 30 mmol) and tetra-*n*-butylammonium iodide (695 mg, 1.88 mmol) were added. The resulting mixture was warmed to room temperature and stirred for 72 h. The reaction was quenched by careful addition of H_2O (15 mL) and the mixture was diluted with *tert*-butyl methyl ether (200 mL). The organic layer was separated and washed with H_2O (2 × 100 mL), dried over Na_2SO_4, and concentrated. The residue was purified by flash chromatography (hexane/EtOAc 10:1) to afford the title compound as a colorless oil (9.11 g, 91%, 98% *ee*). $[\alpha]_D^{20}$ = +9.4 (c = 1.08, $CHCl_3$). MS (EI) *m/z* (%): 459 (1), 427 (2), 121 (100), 75 (5). HRMS (ESI): *m/z* calcd for $C_{31}H_{54}O_5SiNa$ $[M+Na]^+$: 557.3633, found: 557.3629. The spectral data are matching with the enantiomer ***ent*-S2**. The enantiomeric excess was determined by HPLC with a chiral stationary phase [Chiralcel OJ-3R, 150 × 4.6 mm, MeCN/H_2O = 75:25, 35 °C, 1.0 mL/min, λ = 225 nm].

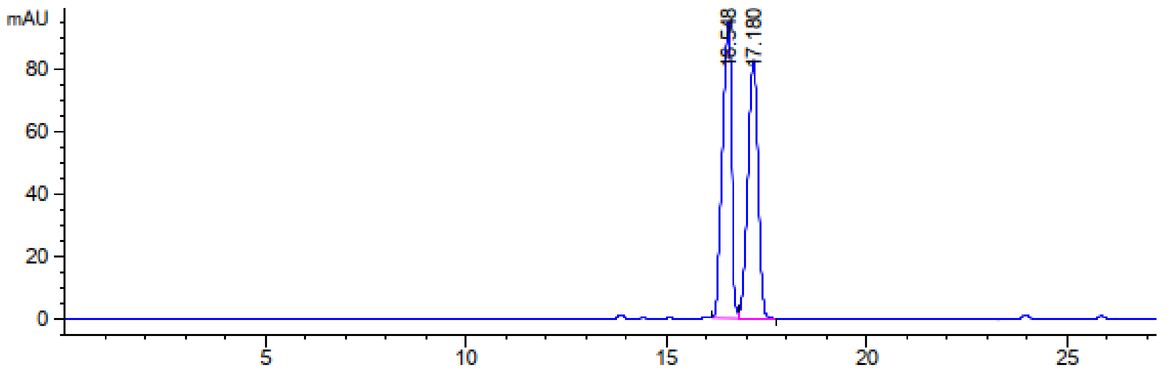

Peak #	RetTime [min]	Type	Width [min]	Area [mAU*s]	Height [mAU]	Area %	
1	16.548	BV	0.2441	1479.79944	94.80336	44.2435	1. diastereomer 1. enantiomer
2	17.180	VB	0.2872	1481.47205	82.60289	44.2935	1. diastereomer 2. enantiomer

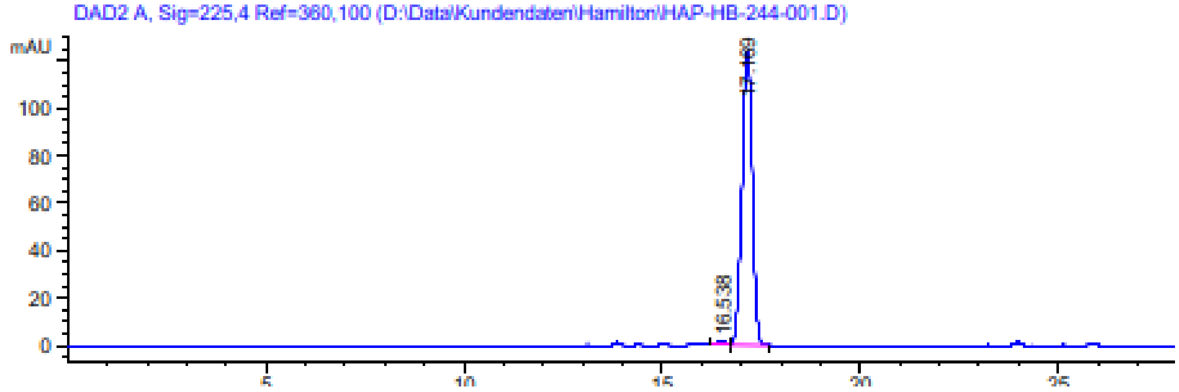

chiral

Peak #	RetTime [min]	Type	Width [min]	Area [mAU*s]	Height [mAU]	Area %		
1	16.538	BV	0.2421	23.02554	1.49135	0.9428	1. diastereomer 1. enantiomer	98.0 % e. e.
2	17.169	VB	0.2936	2270.38184	124.01891	92.9666	1. diastereomer 2. enantiomer	

General Procedure for Liberating the Terminal Alkyne. Tetra-*n*-butylammonium fluoride (1 M in THF, 1.5 equiv.) was added to a solution of silyl ether **S2** (1 equiv.) in THF (0.31 M), and the resulting mixture was stirred for 16 h. Toluene (0.31 M) and powdered sodium hydroxide (10 equiv.) were added and the mixture was vigorously stirred at 110 °C until the starting material was consumed. The mixture was diluted with *tert*-butyl methyl ether (100 mL) and washed with H_2O (3 × 50 mL), the organic phase was dried over $MgSO_4$ and concentrated *in vacuo*. The residue was purified by flash chromatography (hexane/EtOAc = 5:1 to 4:1) to afford the title compound.

1-((((3*R*,4*S*)-8,8-Dimethoxy-3-methyloct-1-yn-4-yl)oxy)methyl)-4-methoxybenzene (*ent*-5). Prepared

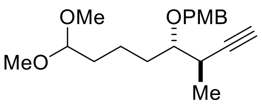

according to the General Procedure using silyl ether ***ent*-S2** (8.00 g, 15.0 mmol) as a colorless oil (4.39 g, 92%, *ee* = 98%). $[\alpha]_D^{25}$ = −14.7 (c = 1.00, $CHCl_3$). ^1H NMR (400 MHz, $CDCl_3$) δ = 7.27 (d, *J* = 8.6 Hz, 2H), 6.87 (d, *J* = 8.6 Hz, 2H), 4.53 (d, *J* = 11.2 Hz, 1H), 4.45 (d, *J* = 11.2 Hz, 1H), 4.34 (t, *J* = 5.7 Hz, 1H), 3.80 (s, 3H), 3.39 (dt, *J* = 8.3, 4.1 Hz,

1H), 3.31 (s, 3H), 3.30 (s, 3H), 2.84 – 2.75 (m, 1H), 2.07 (d, *J* = 2.5 Hz, 1H), 1.72 – 1.45 (m, 5H), 1.42 – 1.31 (m, 1H), 1.17 (d, *J* = 7.1 Hz, 3H). ^{13}C NMR (101 MHz, $CDCl_3$) δ = 159.3, 130.7, 129.6, 113.8, 104.6, 86.5, 80.7, 71.8, 69.7, 55.4, 52.9, 52.7, 32.6, 30.7, 29.1, 21.3, 15.5. IR (neat): 3292, 2938, 1612, 1513, 1459, 1372, 1246, 1174, 1125, 1035, 954, 820, 636, 582, 520 cm^{-1}. MS (ESIpos) *m/z* (%): 343.2 (100 (M+Na)). HRMS (ESI): *m/z* calcd for $C_{19}H_{28}O_4Na$ [M+Na]$^+$: 343.1880, found: 343.1876. The *ee* was determined by GC with a chiral stationary phase (30 m, i.D. 0.25 mm, BGB-176/SE-52; FID; Temperature: 220 °C (injector), 350 °C (detector), 140 °C (iso); flow rate 0.50 bar H_2: major enantiomer t_R = 308 min, minor enantiomer t_R = 315 min.

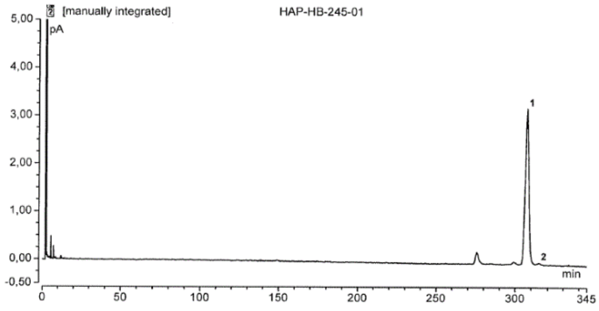

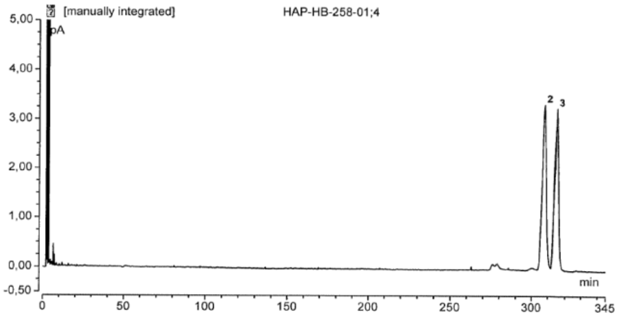

No.	Ret.Time min	Rel.Area %	Peak Name
1	307,61	99,12	
2	314,57	0,88	

No.	Ret.Time min	Rel.Area %	Peak Name
2	308,25	55,42	
3	315,04	44,58	

1-((((3*S*,4*R*)-8,8-Dimethoxy-3-methyloct-1-yn-4-yl)oxy)methyl)-4-methoxybenzene (5). According to the General Procedure using silyl ether **S2** (8.90 g, 16.6 mmol); colorless oil (4.58 g, 86%). $[\alpha]_D^{20}$ = +16.6 (c = 1.18, $CHCl_3$). MS (EI) *m/z* (%): 289 (2), 136 (4), 121 (100), 75 (5). HRMS (ESI): *m/z* calcd for $C_{19}H_{28}O_4Na$ [M+Na]$^+$: 343.1880, found: 343.1881. The spectral data are matching with those of the enantiomer described above.

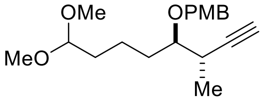

General Procedure for the Entrained Hydroboration. Dicyclohexylborane (10 mol%) and pinacolborane (3 equiv.) were added to alkyne **5** (1 equiv.). After stirring the neat mixture at 35 °C for 3 h, air was bubbled through for 1-2 h at room temperature to oxidize the residual borane. All volatile materials were then evaporated in vacuo and the residue was purified by flash chromatography (hexane/EtOAc = 10:1 to 4:1 to 3:1) to afford the title compound.

Alkenyl Boronate *ent*-6. Prepared according to the General Procedure using alkyne ***ent*-5** (3.78 g, 11.8 mmol) as a colorless oil (4.69 g, 89%). $[\alpha]_D^{25}$ = −5.4 (c = 1.00, $CHCl_3$). ^{1}H NMR (400 MHz, $CDCl_3$) δ = 7.26 (d, *J* = 8.6 Hz, 2H), 6.85 (d, *J* = 8.6 Hz, 2H), 6.60 (dd, *J* = 18.1, 6.8 Hz, 1H), 5.46 (dd, *J* = 18.1, 1.4 Hz, 1H), 4.48 (d, *J* = 11.1 Hz, 1H), 4.39 (d, *J* = 11.1 Hz, 1H), 4.31 (t, *J* = 5.5 Hz, 1H), 3.79 (s, 3H), 3.35 – 3.26 (m,

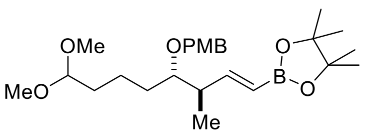

7H), 2.66 – 2.56 (m, 1H), 1.59 – 1.21 (m, 18H), 1.02 (d, *J* = 6.9 Hz, 3H). ^{13}C NMR (101 MHz, $CDCl_3$) δ = 159.2, 156.3, 131.1, 129.6, 118.7, 113.8, 104.7, 83.2, 82.0, 71.5, 55.4, 52.9, 52.7, 42.1, 32.7, 30.5, 25.0, 24.9, 21.2, 13.9. ^{11}B NMR (128 MHz, $CDCl_3$) δ = 29.8. IR (neat): 2935, 1635, 1613, 1513, 1460, 1358, 1320, 1246, 1143, 1036, 969, 849, 821, 656, 578, 518 cm^{-1}. MS (ESIpos) *m/z* (%): 471.3 (100 (M+Na)). HRMS (ESI): *m/z* calcd for $C_{25}H_{41}BO_6Na$ $[M+Na]^+$: 471.2888, found: 471.2892.

Alkenyl Boronate 6. Prepared according to the General Procedure using alkyne **5** (4.58 g, 14.3 mmol)

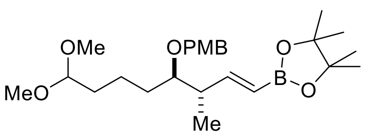

as a colorless oil (5.27 g, 82%). $[\alpha]_D^{20}$ = +1.27 (c = 0.90, $CHCl_3$). MS (ESIpos) *m/z* (%): 471.3 (100 (M+Na)). HRMS (ESI): *m/z* calcd for $C_{25}H_{41}BO_6Na$ $[M+Na]^+$: 471.2888, found: 471.2891. The spectral data are matching with those of the enantiomer described above.

General Procedure for Cross Coupling. $Pd(PPh_3)_4$ (5 mol%), an aqueous solution of sodium hydroxide (2 M, 5 equiv.), and 1-iodopropyne (1.5 equiv.) were added to a solution of alkenyl boronate **6** (1 equiv.) in THF (0.4 M). After stirring at 80 °C for 4 h, the mixture was diluted with H_2O (30 mL). The aqueous layer was separated and extracted with *tert*-butyl methyl ether (3 × 50 mL). The combined organic layers were dried over $MgSO_4$ and concentrated in vacuo. The residue was purified by flash chromatography (hexane/EtOAc = 4:1) to afford the title compound.

Enyne *ent*-7. Prepared according to the General Procedure using alkenyl boronate ***ent*-6** (4.67 g, 10.4

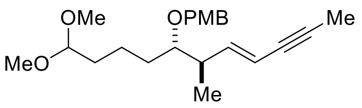

mmol); brown oil (2.35 g, 63%). $[\alpha]_D^{25}$ = −4.6 (c = 1.00, $CHCl_3$). ^1H NMR (400 MHz, $CDCl_3$) δ = 7.26 (d, *J* = 8.6 Hz, 2H), 6.87 (d, *J* = 8.6 Hz, 2H), 6.02 (dd, *J* = 16.0, 7.8 Hz, 1H), 5.48 – 5.41 (m, 1H), 4.47 (d, *J* = 11.0 Hz, 1H), 4.41 (d, *J* = 11.0 Hz, 1H), 4.32 (t, *J* = 5.6 Hz, 1H), 3.80 (s, 3H), 3.302 (s, 3H), 3.300 (s, 3H), 3.24 (dt, *J* = 7.8, 4.2 Hz, 1H), 2.57 – 2.46 (m, 1H), 1.93 (d, *J* = 2.1 Hz, 3H), 1.62 – 1.23 (m, 6H), 1.01 (d, *J* = 6.9 Hz, 3H) ppm. ^{13}C NMR (101 MHz, $CDCl_3$) δ 159.2, 145.3, 131.1, 129.5, 113.8, 110.2, 104.6, 84.8, 82.3, 78.5, 71.8, 55.4, 52.9, 52.7, 40.2, 32.7, 30.8, 21.1, 15.1, 4.3 ppm. IR (neat): 2937, 1612, 1513, 1458, 1362, 1302, 1247, 1172, 1125, 1034, 959, 825, 751, 666, 529 cm^{-1}. HRMS (ESI): *m/z* calcd for $C_{22}H_{32}O_4Na$ $[M+Na]^+$: 383.2193, found: 383.2190.

Enyne 7. Prepared according to the General Procedure using alkenyl boronate **6** (5.09 g, 11.4 mmol);

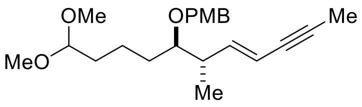

brown oil (2.82 g, 69%). $[\alpha]_D^{20}$ = −10.1 (c = 1.08, $CHCl_3$). MS (ESIpos) *m/z* (%): 378.3 (100 $(M+NH_4)$). HRMS (ESI): *m/z* calcd for $C_{22}H_{32}O_4Na$ $[M+Na]^+$: 383.2193, found: 383.2194.The spectral data are matching with those of the enantiomer described above.

General Procedure for Sharpless Asymmetric Dihydroxylation. A solution of $(DHQD)_2Pyr$ (or $(DHQ)_2Pyr$) (5 mol%), $K_3Fe(CN)_6$ (3 equiv.), potassium carbonate (3 equiv.), and $K_2OsO_4·2H_2O$ (2.1 mol%) in a 1:1 mixture of H_2O (0.4 M) and *tert*-butanol (0.4 M) was stirred in air for 30 min at room temperature before enyne **7** (or *ent*-**7**, respectively) (1 equiv.) and methanesulfonamide (2 equiv.) were added at 0 °C. After stirring at 0 °C for 4 h, the reaction was quenched by addition of saturated aqueous $Na_2S_2O_3$ solution (15 mL). The aqueous layer was separated and extracted with *tert*-butyl methyl ether (3 × 30 mL). The combined organic layers were concentrated and the residue was purified by flash chromatography (hexane/EtOAc = 1:1) to give the title compounds.

Diol *ent*-8. Prepared according to the General Procedure using $(DHQ)_2Pyr$ and enyne ***ent*-7** (2.30 g, 6.39 mmol); yellow viscous oil (2.05 g, 81%, dr = 9:1). The diasteromers were separated by preparative HPLC (YMC Triart C18, 5 µm, 150 × 30 mm, $MeCN/H_2O$ = 35:65, 35 °C, 42.5 mL/min) to afford the pure diastereoisomer ***ent*-8** as a colorless viscous oil (1.27 g, 50%). $[\alpha]_D^{25}$ = +1.6 (c = 1.00, $CHCl_3$). ^1H NMR (400 MHz, $CDCl_3$) δ = 7.23 (d, J = 8.6 Hz, 2H), 6.87 (d, J = 8.6 Hz, 2H), 4.56 (d, J = 10.9 Hz, 1H), 4.40 – 4.33 (m, 2H), 4.25 (dq, J = 8.4, 2.1 Hz, 1H), 3.87 (dd, J = 8.4, 1.9 Hz, 1H), 3.79 (s, 3H), 3.45 (td, J = 6.5, 4.3 Hz, 1H), 3.36 (s, 1H), 3.31 (s, 6H), 2.69 (s, 1H), 2.12 – 2.03 (m, 1H), 1.84 (d, J = 2.1 Hz, 3H), 1.78 – 1.58 (m, 4H), 1.46 – 1.34 (m, 2H), 0.98 (d, J = 7.1 Hz, 3H). ^{13}C NMR (101 MHz, $CDCl_3$) δ = 159.4, 130.4, 129.5, 114.0, 104.5, 83.7, 82.8, 77.1, 74.6, 72.3, 65.1, 55.4, 52.9, 36.4, 32.8, 31.2, 20.6, 11.7, 3.7. IR (neat): 3434, 2939, 1612, 1513, 1459, 1385, 1302, 1246, 1174, 1125, 1033, 819, 513 cm^{-1}. MS (ESIpos) m/z (%): 417.2 (100 (M+Na)). HRMS (ESI): m/z calcd for $C_{22}H_{34}O_6Na$ $[M+Na]^+$: 417.2248, found: 417.2250.

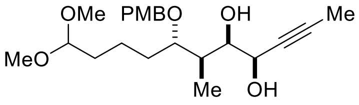

Diol 8. Prepared according to the General Procedure using $(DHQ)_2Pyr$ and enyne **7** (2.82 g, 7.82 mmol) as a yellow viscous oil (2.91 g, 94%, dr = 9:1). The diastereomers were separated by preparative HPLC (YMC Triart C18, 5 µm, 150 × 30 mm, $MeCN/H_2O$ = 35:65, 35 °C, 42 mL/min) to afford the pure diastereoisomer **8** as a colorless viscous oil (1.89 g, 61%).

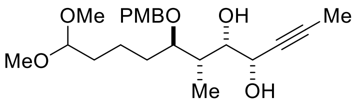

For preparative purposes, the diastereomeric mixtures were used without HPLC separation; the minor isomers were conveniently removed in the next step after TES-protection/acetal cleavage.

General Procedure for Acetal Cleavage/Diol Protection. 2,6-Lutidine (6-7 equiv.) and TESOTf (4-5 equiv.) were added to a solution of acetal **8** in CH_2Cl_2 (0.1 M) at 0 °C. The resulting mixture was stirred at 0 °C for 1 h before H_2O (10 mL) was added. After stirring at room temperature for another 10 min, the mixture was diluted with *tert*-butyl methyl ether (50 mL). The organic phase was separated and washed with H_2O (20 mL) and brine (20 mL), dried over $MgSO_4$, and concentrated in vacuo. Purification of the crude product by flash chromatography (hexane/EtOAc = 9:1) gave the title compound.

Aldehyde *ent*-9. Prepared according to the General Procedure using diol ***ent*-8** (1.27 g, 3.21 mmol), 2,6-lutidine (6 equiv.) and TESOTf (4 equiv.) as a colorless oil (1.13 g, 61%). $[\alpha]_D^{25}$ = −31.6 (c = 1.00, $CHCl_3$). ^{1}H NMR (400 MHz, $CDCl_3$) δ = 9.70 (t, J = 2.0 Hz, 1H), 7.28 (d, J = 8.6 Hz, 2H), 6.86 (d, J = 8.6 Hz, 2H), 4.52 (d, J = 11.0 Hz, 1H), 4.37 – 4.32 (m, 2H), 3.80 (s, 3H), 3.71 (dt, J = 7.4, 5.0 Hz, 1H), 3.58 (dd, J = 6.3, 4.5 Hz, 1H), 2.49 – 2.40 (m, 1H), 2.39 – 2.32 (m, 2H), 1.87 – 1.76 (m, 4H), 1.60 – 1.51 (m, 1H), 1.48 – 1.40 (m, 2H), 1.01 – 0.92 (m, 21H), 0.69 – 0.58 (m, 12H). ^{13}C NMR (101 MHz, $CDCl_3$) δ = 203.0, 159.1, 131.6, 129.5, 113.8, 82.3, 79.3, 78.7, 76.3, 70.1, 67.9, 55.4, 44.3, 38.1, 29.4, 18.9, 10.4, 7.1, 7.0, 5.4, 4.9, 3.9. IR (neat): 2953, 2876, 1726, 1613, 1513, 1459, 1246, 1069, 1037, 1004, 819, 725, 577, 518 cm^{-1}. MS (ESIpos) *m/z* (%): 599.4 (100 (M+Na)). HRMS (ESI): *m/z* calcd for $C_{32}H_{56}O_5Si_2Na$ [M+Na]$^+$: 599.3559, found: 599.3562.

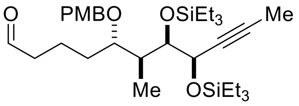

Aldehyde 9. Prepared according to the General Procedure using diol **8** (1.46 g, 3.70 mmol), 2,6-lutidine (7 equiv.) and TESOTf (5 equiv.) as a colorless oil (1.34 g, 63%). The analytical and spectral data are matching with those of the enantiomer described above.

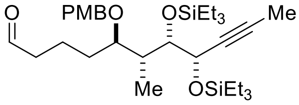

Lactone 10. A solution of *O*-trimethylsilyl quinidine (92 mg, 0.23 mmol) in CH_2Cl_2 (3.4 mL) was added to a stirred suspension of lithium perchlorate (247 mg, 2.32 mmol) in Et_2O (1.7 mL). After stirring until the mixture had become homogeneous, Hünig's base (1.0 mL, 5.8 mmol) and a solution of aldehyde **9** (1.34 g, 2.32 mmol) in a 2:1 mixture of CH_2Cl_2 and Et_2O (3.0 mL, a CH_2Cl_2/Et_2O-ratio of 2:1 is important for the solubility of $LiClO_4$) were added at −78 °C. After slow addition of a solution of acetyl chloride (0.30 mL, 4.3 mmol) in CH_2Cl_2 (0.9 mL) over the course of 5 h at −78 °C, the mixture was stirred at the same temperature for another 15 h before it was diluted with *tert*-butyl methyl ether (50 mL). After filtration through a plug of silica, which was carefully rinsed with *tert*-butyl methyl ether (50 mL), the combined filtrates were concentrated and the residue was purified by flash chromatography (hexane/EtOAc = 9:1) to afford the title compound as a colorless oil (1.12 g, 78%, dr = 19:1). $[\alpha]_D^{25}$ = +36.8 (c = 1.00, $CHCl_3$). ^{1}H NMR (400 MHz, $CDCl_3$) δ 7.27 (d, J = 8.7 Hz, 2H), 6.86 (d, J = 8.7 Hz, 2H), 4.53 (d, J = 11.0 Hz, 1H), 4.42 (dtd, J = 7.3, 5.8, 4.3 Hz, 1H), 4.37 – 4.29 (m, 2H), 3.80 (s, 3H), 3.76 – 3.70 (m, 1H), 3.56 (dd, J = 6.6, 4.4 Hz, 1H), 3.45 (dd, J = 16.2, 5.8 Hz, 1H), 2.99 (dd, J = 16.2, 4.3 Hz, 1H), 2.50 –

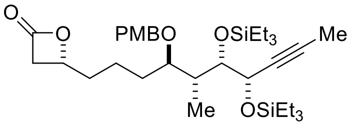

2.40 (m, 1H), 1.87 – 1.74 (m, 4H), 1.73 – 1.62 (m, 1H), 1.56 – 1.40 (m, 3H), 1.40 – 1.28 (m, 1H), 1.01 – 0.93 (m, 21H), 0.73 – 0.58 (m, 12H). ^{13}C NMR (101 MHz, $CDCl_3$) δ 168.5, 159.2, 131.5, 129.6, 113.8, 82.3, 79.2, 78.6, 76.4, 71.5, 70.2, 68.0, 55.4, 43.0, 38.2, 35.2, 29.5, 21.7, 10.5, 7.1, 7.0, 5.4, 4.9, 3.9. IR (neat): 2953, 2913, 2875, 1828, 1612, 1513, 1458, 1413, 1302, 1246, 1079, 1037, 1003, 865, 819, 725, 578, 514 cm^{-1}. MS (ESIpos) *m/z* (%): 641.4 (100 (M+Na)). HRMS (ESI): *m/z* calcd for $C_{34}H_{58}O_6Si_2Na$ [M+Na]$^+$: 641.3664, found: 641.3670.

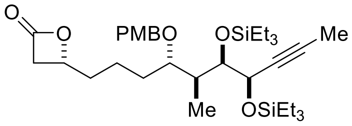

Lactone 12. A solution of *O*-trimethylsilyl quinidine (76.6 mg, 0.193 mmol) in CH_2Cl_2 (8.4 mL) was added to a stirred suspension of lithium perchlorate (103 mg, 0.966 mmol) in Et_2O (5.2 mL). After stirring until the mixture had turned homogeneous, Hünig's base (0.84 mL, 4.83 mmol) and a solution of aldehyde ***ent*-9** (1.11 g, 1.93 mmol) in CH_2Cl_2 (4.5 mL) were introduced at −78 °C, followed by slow addition of a solution of acetyl chloride (0.34 mL, 4.83 mmol) in CH_2Cl_2 (1.12 mL) over the course of 4 h. Once the addition was complete, stirring was continued at −78 °C for 15 h before additional lithium perchlorate (51.4 mg, 0.483 mmol) and Hünig's base (0.17 mL, 0.97 mmol) was introduced, followed by dropwise addition of a solution of additional acetyl chloride (68.7 µL, 0.966 mmol) in CH_2Cl_2 (0.22 mL). After stirring for another 4 h, the mixture was diluted with *tert*-butyl methyl ether (50 mL) and filtered through a plug of silica, which was rinsed with *tert*-butyl methyl ether (50 mL). The combined filtrates were concentrated and the residue was purified by flash chromatography (hexane/EtOAc = 6:1) to give the stereomerically pure title compound as a colorless oil (1.01 g, 84%; the crude product showed a dr ≈ 10:1 (^{1}H NMR). $[\alpha]_D^{25}$ = −27.7 (c = 1.00, $CHCl_3$). ^{1}H NMR (400 MHz, $CDCl_3$) δ = 7.27 (d, *J* = 8.6 Hz, 2H), 6.86 (d, *J* = 8.6 Hz, 2H), 4.53 (d, *J* = 11.1 Hz, 1H), 4.48 – 4.40 (m, 1H), 4.38 – 4.29 (m, 2H), 3.80 (s, 3H), 3.75 – 3.69 (m, 1H), 3.56 (dd, *J* = 6.5, 4.4 Hz, 1H), 3.44 (dd, *J* = 16.2, 5.8 Hz, 1H), 2.96 (dd, *J* = 16.2, 4.2 Hz, 1H), 2.50 – 2.40 (m, 1H), 1.88 – 1.77 (m, 4H), 1.71 – 1.50 (m, 2H), 1.49 – 1.40 (m, 2H), 1.31 – 1.19 (m, 1H), 1.02 – 0.91 (m, 21H), 0.70 – 0.57 (m, 12H). ^{13}C NMR (101 MHz, $CDCl_3$) δ = 168.5, 159.2, 131.5, 129.6, 113.8, 82.3, 79.0, 78.6, 76.4, 71.4, 70.1, 68.0, 55.4, 42.8, 38.1, 34.9, 29.2, 21.4, 10.5, 7.1, 7.0, 5.4, 4.9, 3.9. IR (neat): 2953, 2876, 1828, 1613, 1513, 1459, 1414, 1301, 1246, 1066, 1004, 865, 818, 726, 578, 514 cm^{-1}. MS (ESIpos) *m/z* (%): 641.4 (100 (M+Na)). HRMS (ESI): *m/z* calcd for $C_{34}H_{58}O_6Si_2Na$ [M+Na]$^+$: 641.3664, found: 641.3666.

General Procedure for the Allylation of the β-Lactone. Trimethylaluminum (2 M in toluene, 1 equiv.) was added at 0 °C to a stirred solution of *N*,*O*-dimethylhydroxylamine hydrochloride (2 equiv.) in CH_2Cl_2 (0.27 M). After stirring for 15 min at room temperature, a solution of the respective β-lactone (0.911 g, 1.47 mmol) in CH_2Cl_2 (0.27 M) was added at 0 °C and stirring was continued at this temperature for 0.5 h. The reaction was quenched by addition of a 1:1 mixture of saturated aqueous Rochelle salt solution and saturated aqueous NH_4Cl solution (15 mL). The aqueous phase was separated and extracted with CH_2Cl_2 (3 × 10 mL). The combined organic layers were dried over $MgSO_4$ and concentrated in vacuo to afford the corresponding Weinreb amide (quant.) as a colorless oil, which was used directly in the next step. For analytical purposes an aliquot was purified by flash chromatography (hexane/EtOAc = 1:1); for the analytical data, see below.

Allylmagnesium chloride (2 M in THF, 3 equiv.) was added to a stirred solution of the crude Weinreb amide in THF (0.1 M) at −78 °C and stirring was continued at 0 °C for 2 h. The reaction was quenched by addition of saturated aqueous NH_4Cl solution (20 mL), and the aqueous phase was separated and extracted with *tert*-butyl methyl ether (3 × 20 mL). The combined organic layers were dried over $MgSO_4$ and concentrated in vacuo. Purification of the crude product by flash chromatography (hexane/EtOAc = 4:1) gave the corresponding β,γ-enone and a small amount of the isomerized α,β-unsaturated ketone **S3**

β,γ-Enone 13. Prepared according to the General Procedure using lactone **12** (911 mg, 1.47 mmol) as a colorless oil (928 mg, 95% over 2 steps). $[\alpha]_D^{25}$ = −42.2 (c = 1.00, $CHCl_3$).

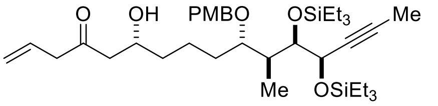

^{1}H NMR (400 MHz, $CDCl_3$) δ 7.28 (d, *J* = 8.7 Hz, 2H), 6.85 (d, *J* = 8.7 Hz, 2H), 5.90 (ddt, *J* = 17.1, 10.2, 7.0 Hz, 1H), 5.20 (dq, *J* = 10.2, 1.4 Hz, 1H), 5.15 (dq, *J* = 17.1, 1.4 Hz, 1H), 4.51 (d, *J* = 11.0 Hz, 1H), 4.36 – 4.31 (m, 2H), 4.03 – 3.94 (m, 1H), 3.79 (s, 3H), 3.70 – 3.64 (m, 1H), 3.60 (dd, *J* = 6.1, 4.6 Hz, 1H), 3.17 (dt, *J* = 7.0, 1.4 Hz, 2H), 2.85 (s, 1H), 2.60 – 2.37 (m, 3H), 1.83 (d, *J* = 2.2 Hz, 3H), 1.64 – 1.18 (m, 6H), 1.01 – 0.91 (m, 21H), 0.69 – 0.58 (m, 12H). ^{13}C NMR (101 MHz, $CDCl_3$) δ 209.9, 159.1, 131.7, 130.2, 129.6, 119.4, 113.8, 82.2, 79.5, 78.8, 76.3, 70.0, 67.9, 67.7, 55.4, 48.6, 48.5, 38.1, 36.8, 29.6, 21.9, 10.5, 7.1, 7.0, 5.4, 4.9, 3.9. IR (neat): 3473, 2952, 2876, 1710, 1613, 1513, 1459, 1245, 1072, 1037, 1004, 819, 726, 578, 515 cm^{-1}. HRMS (ESI): *m*/*z* calcd for $C_{37}H_{64}O_6Si_2Na$ [M+Na]$^+$: 683.4134, found: 683.4139.

Mosher Ester S4 derived from 13 (with Concomitant C=C Bond Isomerization). Et_3N (6.1 µL, 80 µmol) and DMAP (0.2 mg, 1.5 µmol) were added to a solution of **13** (10 mg, 15 µmol) in CH_2Cl_2 (1.0 mL) followed by (*S*)-(–)-*α*-methoxy-*α*-trifluoromethyl-phenylacetyl chloride ((*S*)-MTPA-Cl) (4.2 µL, 22 µmol). The mixture was stirred at ambient temperature for 17 h before it was diluted with CH_2Cl_2 (2 mL) and saturated $NaHCO_3$ (2 mL).

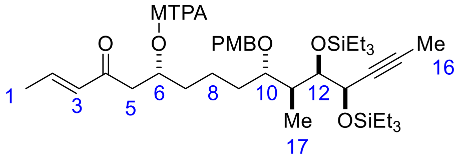

The aqueous phase was separated and extracted with CH_2Cl_2 (3 × 2 mL). The combined organic phases were dried over Na_2SO_4, filtered and concentrated. The residue was purified by flash chromatography (hexane/EtOAc = 4:1) to give the corresponding (*R*)-Mosher ester (*R*)-**S4** (4.9 mg, 37%), which analyzed as follows: $[\alpha]_D^{20}$ = +17.7 (c = 0.31, $CHCl_3$). ^{1}H NMR (400 MHz, $CDCl_3$) δ = 7.52 – 7.45 (m, 2H), 7.40 – 7.32 (m, 3H), 7.27 – 7.20 (m, 2H), 6.88 – 6.77 (m, 3H), 6.08 (dd, *J* = 15.8, 1.7 Hz, 1H), 5.57 – 5.45 (m, 1H), 4.46 (d, *J* = 11.0 Hz, 1H), 4.32 (dq, *J* = 4.3, 2.2 Hz, 1H), 4.28 (d, *J* = 11.0 Hz, 1H), 3.78 (s, 3H), 3.60 (dt, *J* = 10.7, 5.1 Hz, 2H), 3.47 (d, *J* = 1.2 Hz, 3H), 2.92 (dd, *J* = 16.4, 8.1 Hz, 1H), 2.64 (dd, *J* = 16.4, 4.6 Hz, 1H), 2.38 (q, *J* = 6.1 Hz, 1H), 1.88 (dd, *J* = 6.8, 1.6 Hz, 3H), 1.83 (d, *J* = 2.2 Hz, 3H), 1.63 (q, *J* = 6.1, 5.6 Hz, 1H), 1.59 – 1.50 (m, 1H), 1.51 – 1.33 (m, 3H), 1.25 – 1.07 (m, 1H), 0.96 (td, *J* = 7.9, 5.0 Hz, 18H), 0.90 (d, *J* = 6.9 Hz, 3H), 0.71 – 0.55 (m, 12H). IR (neat): 2954, 2876, 1748, 1678, 1613, 1514, 1459, 1415, 1381, 1248, 1169, 1079, 1015, 866, 798, 720 cm^{-1}. MS (ESIpos) *m/z* (%): 899.5 (100 (M+Na)). HRMS (ESI): *m/z* calcd for $C_{47}H_{71}F_3O_8Si_2Na$ $[M+Na]^+$: 899.4532, found: 899.4529.

The corresponding Mosher ester (*S*)-**S4** (7.9 mg, 60%) was prepared analogously: $[\alpha]_D^{20}$ = –51.8 (c = 0.22, $CHCl_3$). ^{1}H NMR (400 MHz, $CDCl_3$) δ = 7.54 – 7.46 (m, 2H), 7.40 – 7.31 (m, 3H), 7.27 – 7.19 (m, 2H), 6.88 – 6.79 (m, 2H), 6.76 (dq, *J* = 15.8, 6.8 Hz, 1H), 6.00 (dd, *J* = 15.8, 1.7 Hz, 1H), 5.57 – 5.46 (m, 1H), 4.48 (d, *J* = 10.9 Hz, 1H), 4.36 – 4.27 (m, 2H), 3.78 (s, 3H), 3.67 – 3.57 (m, 2H), 3.50 – 3.45 (m, 3H), 2.87 (dd, *J* = 16.2, 7.6 Hz, 1H), 2.58 (dd, *J* = 16.2, 5.2 Hz, 1H), 2.40 (dt, *J* = 7.2, 5.8 Hz, 1H), 1.85 (dd, *J* = 6.8, 1.7 Hz, 3H), 1.82 (d, *J* = 2.2 Hz, 3H), 1.74 – 1.56 (m, 3H), 1.42 (q, *J* = 8.2 Hz, 2H), 1.31 – 1.22 (m, 1H), 0.97 (td, *J* = 7.9, 5.0 Hz, 18H), 0.92 (d, *J* = 6.9 Hz, 3H), 0.71 – 0.56 (m, 12H). IR (neat): 2954, 2876, 1748, 1675, 1633, 1513, 1457, 1415, 1257, 1169, 1078, 1015, 863, 798, 722, 577, 511 cm^{-1}. MS (ESIpos) *m/z* (%): 899.5 (100 (M+Na)). HRMS (ESI): *m/z* calcd for $C_{47}H_{71}F_3O_8Si_2Na$ $[M+Na]^+$: 899.4532, found: 899.4535.

Table S-2. Analysis of the Mosher esters **S4**; arbitrary numbering scheme as shown in the insert

Assignment	13 [ppm]	(*S*)-S4 [ppm]	(*R*)-S4 [ppm]	Δ (δ(*S*–*R*)) [ppm]
1^{trans}, 1^{cis}	5.15, 5.20	1.85	1.88	–0.03
2	5.90	6.77	6.82	–0.05
3	3.17	6.00	6.08	–0.08
5	2.56	2.87	2.92	–0.05
5’	2.47	2.58	2.64	–0.06
6	3.99	5.51	5.51	0.00
7	1.64-1.18	1.65	1.59	+0.06
8	1.64-1.18	1.59	1.45	+0.06
8’	1.64-1.18	1.25	1.17	+0.08
9	1.64-1.18	1.42	1.39	+0.03
10	3.67	3.62	3.61	+0.01
11	2.42	2.40	2.38	+0.02

12	3.60	3.62	3.61	+0.01
13	4.34	4.33	4.32	+0.01
16	1.83	1.83	1.83	0.00
17	0.94	0.92	0.90	+0.02

β,γ-Enone 11. Prepared according to the General Procedure using lactone **10** (1.05 g, 1.70 mmol); the reaction yielded **β,γ-enone 11** (988 mg, 88% over 2 steps) and the corresponding α,β-unsaturated ketone **S3** (13 mg, 2% over 2 steps) as a colorless oil each. Analytical and spectral data for **11**: $[\alpha]_D^{25}$ = +12.2 (c = 1.00, $CHCl_3$). ^{1}H NMR (400 MHz, $CDCl_3$) δ = 7.27 (d, *J* = 8.7 Hz, 2H), 6.86 (d, *J* = 8.6 Hz, 2H), 5.90 (ddt, *J* = 17.1, 10.2, 7.0 Hz, 1H), 5.20 (dq, *J* = 10.2, 1.4 Hz, 1H), 5.15 (dq, *J* = 17.1, 1.4 Hz, 1H), 4.51 (d, *J* = 11.0 Hz, 1H), 4.37 – 4.31 (m, 2H), 4.03 – 3.95 (m, 1H), 3.79 (s, 3H), 3.71 – 3.65 (m, 1H), 3.60 (dd, *J* = 6.1, 4.6 Hz, 1H), 3.18 (dt, *J* = 7.0, 1.2 Hz, 2H), 2.85 (d, *J* = 3.7 Hz, 1H), 2.61 – 2.37 (m, 3H), 1.83 (d, *J* = 2.2 Hz, 3H), 1.53 – 1.29 (m, 6H), 1.01 – 0.92 (m, 21H), 0.73 – 0.57 (m, 12H). ^{13}C NMR (101 MHz, $CDCl_3$) δ = 209.9, 159.1, 131.7, 130.2, 129.6, 119.4, 113.8, 82.2, 79.8, 78.8, 76.3, 70.1, 67.84, 67.82, 55.4, 48.8, 48.6, 38.1, 36.9, 29.7, 22.1, 10.4, 7.1, 7.0, 5.4, 4.9, 3.9. IR (neat): 3456, 2952, 2911, 2875, 1711, 1613, 1513, 1458, 1414, 1302, 1246, 1071, 1037, 1003, 863, 820, 725, 576, 519 cm^{-1}. MS (ESIpos) *m/z* (%): 683.4 (100 (M+Na)). HRMS (ESI): *m/z* calcd for $C_{37}H_{64}O_6Si_2Na$ [M+Na]$^+$: 683.4134, found: 683.4137.

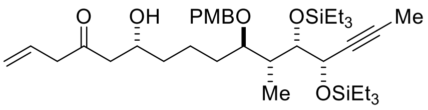

Analytical and spectral data for **S3.** $[\alpha]_D^{20}$ = +12.1 (c = 1.31, $CHCl_3$). ^{1}H NMR (600 MHz, $CDCl_3$) δ = 7.29 – 7.26 (m, 2H), 6.89 – 6.82 (m, 3H), 6.11 (dq, *J* = 15.7, 1.6 Hz, 1H), 4.51 (d, *J* = 11.0 Hz, 1H), 4.37 – 4.32 (m, 2H), 4.07 – 3.98 (m, 1H), 3.79 (s, 3H), 3.70 – 3.65 (m, 1H), 3.61 (dd, *J* = 6.0, 4.7 Hz, 1H), 3.10 (d, *J* = 3.5 Hz, 1H), 2.68 (dd, *J* = 17.2, 2.7 Hz, 1H), 2.56 (dd, *J* = 17.2, 9.1 Hz, 1H), 2.47 – 2.38 (m, 1H), 1.91 (dd, *J* = 6.8, 1.7 Hz, 3H), 1.83 (d, *J* = 2.2 Hz, 3H), 1.50 (tdd, *J* = 16.3, 8.8, 3.2 Hz, 2H), 1.46 – 1.39 (m, 2H), 1.41 – 1.31 (m, 2H), 0.97 (td, *J* = 8.0, 5.7 Hz, 18H), 0.94 (d, *J* = 6.9 Hz, 3H), 0.68 – 0.59 (m, 12H). ^{13}C NMR (151 MHz, $CDCl_3$) δ = 201.2, 159.1, 144.0, 132.4, 131.8, 129.6, 113.8, 82.2, 79.9, 78.8, 77.4, 77.2, 77.0, 76.3, 70.1, 68.1, 67.8, 55.4, 46.1, 38.1, 37.0, 29.9, 22.2, 18.5, 10.4, 7.1, 7.0, 5.4, 4.9, 3.9. IR (neat): 2952, 2912, 2875, 1664, 1630, 1613, 1513, 1459, 1246, 1073, 1037, 1004, 970, 806, 726 cm^{-1}. MS (EI) *m/z* (%): 459 (1), 393 (1), 293 (2), 257 (1), 121 (100), 87 (5). HRMS (ESI): *m/z* calcd for $C_{37}H_{64}O_6Si_2Na$ [M+Na]$^+$: 683.4134, found: 683.4131.

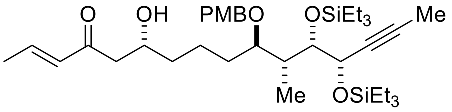

Analytical and spectral data of the Weinreb amide **S5** derived from **10**: $[\alpha]_D^{20}$ = −7.4 (c = 0.93, $CHCl_3$). ^{1}H NMR (400 MHz, $CDCl_3$) δ = 7.31 – 7.24 (m, 2H), 6.89 – 6.80 (m, 2H), 4.51 (d, *J* = 10.9 Hz, 1H), 4.39 – 4.30 (m, 2H), 4.03 –

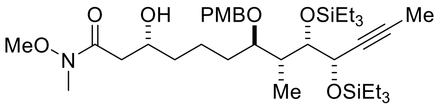

3.94 (m, 1H), 3.79 (s, 3H), 3.70 – 3.64 (m, 4H), 3.62 (dd, J = 5.9, 4.7 Hz, 1H), 3.19 (s, 3H), 2.64 (d, J = 16.9 Hz, 1H), 2.48 – 2.34 (m, 2H), 1.83 (d, J = 2.2 Hz, 3H), 1.55 (d, J = 7.8 Hz, 2H), 1.51 – 1.34 (m, 4H), 1.01 – 0.92 (m, 21H), 0.73 – 0.58 (m, 12H). ^{13}C NMR (101 MHz, $CDCl_3$) δ = 174.3, 159.1, 131.8, 129.5, 113.8, 82.2, 80.0, 78.8, 76.3, 70.1, 68.2, 67.8, 61.3, 55.4, 38.3, 38.1, 37.1, 31.9, 30.0, 22.3, 10.4, 7.2, 7.0, 5.4, 5.0, 3.9. IR (neat): 3447, 2952, 2912, 2875, 1648, 1613, 1587, 1513, 1459, 1415, 1385, 1350, 1301, 1245, 1172, 1073, 1037, 1003, 866, 820, 790, 726, 673, 578, 514, 433 cm^{-1}. MS (ESIpos) *m/z* (%): 702.4 (100 (M+Na)). HRMS (ESI): *m/z* calcd for $C_{36}H_{66}O_7Si_2N$ $[M+H]^+$: 680.4372, found: 680.4371.

Mosher Ester S6 Derived from Weinreb amide S5. Et_3N (5.1 µL, 36 µmol) and DMAP (0.2 mg, 1.5 µmol) were added to a solution of alcohol **S5** (8.5 mg, 13 µmol) in CH_2Cl_2 (0.8 mL) followed by (*S*)-(–)-*α*-methoxy-*α*-trifluoromethyl-phenylacetyl chloride ((*S*)-MTPA-Cl) (3.5 µL, 19 µmol). The mixture was stirred at ambient temperature for 17 h before it was diluted with CH_2Cl_2 (2 mL) and saturated $NaHCO_3$ (2 mL). The aqueous phase was separated and extracted with CH_2Cl_2 (3 × 2 mL). The combined organic phases were dried over Na_2SO_4, filtered and concentrated. The residue was purified by flash chromatography (hexane/EtOAc = 10:1) to give the corresponding (*R*)-Mosher ester (*R*)-**S6** (8.7 mg, 79%), which analyzed as follows: $[\alpha]_D^{20}$ = +33.7 (c = 0.87, $CHCl_3$). ^{1}H NMR (400 MHz, $CDCl_3$) δ = 7.55 – 7.47 (m, 2H), 7.39 – 7.32 (m, 3H), 7.27 – 7.19 (m, 2H), 6.88 – 6.80 (m, 2H), 5.61 – 5.50 (m, 1H), 4.47 (d, J = 11.0 Hz, 1H), 4.36 – 4.27 (m, 2H), 3.79 (s, 3H), 3.69 – 3.55 (m, 5H), 3.49 (d, J = 1.1 Hz, 3H), 3.15 (s, 3H), 2.87 (dd, J = 16.0, 9.0 Hz, 1H), 2.53 (dd, J = 16.1, 3.8 Hz, 1H), 2.39 (dqd, J = 12.3, 6.9, 5.5 Hz, 1H), 1.82 (d, J = 2.2 Hz, 3H), 1.76 – 1.53 (m, 3H), 1.48 – 1.36 (m, 2H), 1.31 – 1.19 (m, 1H), 1.02 – 0.91 (m, 18H), 0.91 (d, J = 6.9 Hz, 3H), 0.75 – 0.55 (m, 12H). IR (neat): 2954, 2914, 2876, 1748, 1667, 1613, 1513, 1459, 1416, 1389, 1247, 1169, 1079, 1009, 863, 796, 742, 698, 667, 579, 511 cm^{-1}. MS (ESIpos) *m/z* (%): 918.5 (100 (M+Na)). HRMS (ESI): *m/z* calcd for $C_{46}H_{72}O_9Si_2NF_3Na$ $[M+Na]^+$: 918.4590, found: 918.4602.

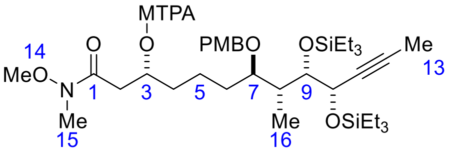

The corresponding Mosher ester (*S*)-**S6** (8.3 mg, 74%) was prepared analogously: $[\alpha]_D^{20}$ = +7.5 (c = 0.83, $CHCl_3$). ^{1}H NMR (400 MHz, $CDCl_3$) δ = 7.57 – 7.48 (m, 2H), 7.40 – 7.32 (m, 3H), 7.28 – 7.21 (m, 2H), 6.88 – 6.79 (m, 2H), 5.57 (dq, J = 10.4, 5.6 Hz, 1H), 4.50 (d, J = 11.1 Hz, 1H), 4.36 – 4.26 (m, 2H), 3.78 (s, 3H), 3.69 – 3.56 (m, 2H), 3.51 (s, 6H), 3.06 (s, 3H), 2.85 (dd, J = 15.9, 8.8 Hz, 1H), 2.57 – 2.37 (m, 2H), 1.83 (d, J = 2.1 Hz, 3H), 1.77 – 1.63 (m, 2H), 1.56 – 1.29 (m, 4H), 1.02 – 0.89 (m, 21H), 0.71 – 0.56 (m, 12H). IR (neat): 2955, 2914, 2876, 1749, 1668, 1513, 1247, 1169, 1010, 1009, 798, 718 cm^{-1}. MS (ESIpos) *m/z* (%): 918.5 (100 (M+Na)). HRMS (ESI): *m/z* calcd for $C_{46}H_{72}O_9Si_2NF_3Na$ $[M+Na]^+$: 918.4590, found: 918.4604.

Table S-3. Mosher ester analysis for **S5**; arbitrary numbering scheme as shown in the insert

Assignment	S5 [ppm]	(*S*)-S6 [ppm]	(*R*)-S6 [ppm]	Δ (δ(*S*–*R*)) [ppm]
2	2.64	2.85	2.87	–0.02
2′	2.52	2.43	2.53	–0.10
3	3.99	5.57	5.56	+0.01
4	1.57	1.74	1.69	+0.05
4′	1.43	1.69	1.62	+0.07
5	1.57	1.54	1.41	+0.13
5′	1.43	1.33	1.26	+0.07
6	1.46	1.45	1.41/1.26	+0.04/+0.19
7	3.70	3.65	3.61	+0.04
8	2.44	2.43	2.39	+0.04
9	3.62	3.62	3.61	+0.01
10	4.34	4.32	4.32	0.00
13	1.83	1.82	1.82	+0.00
14	3.79	3.78	3.79	–0.01
15	3.66	3.51	3.58	–0.07
16	0.97	0.94	0.91	+0.03

Fragment Assembly and Synthesis of Diastereomeric Core Macrocycles

General Procedure for the Evans-Tishchenko Redox Esterification. Benzaldehyde (1.5 – 8 equiv.) was added to a solution freshly prepared samarium diiodide (0.1 M in THF, 1.5 – 8 equiv.) and the resulting mixture was stirred for 0.5 h at room temperature. After cooling to the indicated temperature, a solution of aldehyde **18** (0.5 M in THF, 2 – 4 equiv.) and a solution of the corresponding hydroxyketone (1 equiv.) in THF (0.15 M with rinses) were added and the mixture was stirred for 24 h at the same temperature. The reaction was quenched with saturated aqueous Rochelle salt (2 mL) and the aqueous layer was extracted with *tert*-butyl methyl ether (3 × 3 mL). The combined organic layers were washed with H_2O (3 mL) and concentrated in vacuo. Purification of the crude product by flash chromatography (hexane/EtOAc = 4:1) gave a mixture of the constitutional isomers, which were used in the next step without further purification.

2,6-Lutidine (220 mol%) and TBSOTf (200 mol%) were added to a solution of the crude alcohol mixture at 0 °C. After stirring at 0 °C for 1 h, the reaction was quenched with saturated aqueous NH_4Cl solution (5 mL). The aqueous phase was separated and extracted with *tert*-butyl methyl ether (3 × 5 mL). The combined organic layers were washed with brine (10 mL), dried over Na_2SO_4 and concentrated in vacuo. Purification of the crude product by flash chromatography (hexane/EtOAc = 14:1) gave a

mixture of constitutional isomers, which could be separated after RCAM. For analytical purposes an aliquot was purified by preparative TLC (hexane/EtOAc = 14:1).

Compound 23. Prepared according to the General Procedure using ketone **13** (100 mg, 0.151 mmol), aldehyde **18** (2 equiv.), SmI_2 (1.5 equiv.) and benzaldehyde (1.5 equiv.) at −15 °C. Colorless oil (90.5 mg, *mixture of constitutional isomers*, 64% over 2 steps). Analytical and spectroscopic data for pure **23**: $[\alpha]_D^{25}$ = −23.3 (c = 1.00, $CHCl_3$).

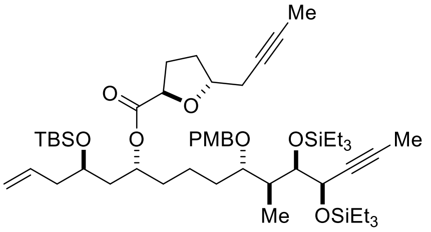

^{1}H NMR (400 MHz, $CDCl_3$) δ = 7.26 (d, *J* = 8.6 Hz, 2H), 6.86 (d, *J* = 8.6 Hz, 2H), 5.78 (ddt, *J* = 19.7, 9.4, 7.1 Hz, 1H), 5.09 – 5.01 (m, 2H), 5.00 – 4.92 (m, 1H), 4.54 – 4.45 (m, 2H), 4.37 – 4.25 (m, 3H), 3.79 (s, 3H), 3.77 – 3.69 (m, 1H), 3.67 – 3.58 (m, 2H), 2.49 – 2.18 (m, 6H), 2.12 – 2.02 (m, 1H), 2.01 – 1.91 (m, 1H), 1.82 (d, *J* = 2.2 Hz, 3H), 1.80 – 1.63 (m, 5H), 1.62 – 1.46 (m, 4H), 1.43 – 1.35 (m, 2H), 1.27 – 1.15 (m, 1H), 1.01 – 0.84 (m, 30H), 0.69 – 0.57 (m, 12H), 0.04 (s, 3H), 0.03 (s, 3H). ^{13}C NMR (101 MHz, $CDCl_3$) δ = 172.9, 159.1, 134.6, 131.7, 129.4, 117.4, 113.8, 82.2, 79.6, 78.9, 78.8, 77.6, 77.3, 76.3, 75.3, 72.9, 70.0, 68.7, 67.8, 55.4, 42.7, 41.4, 38.1, 35.3, 30.2, 30.1, 29.8, 26.0, 25.5, 21.7, 18.2, 10.5, 7.1, 7.0, 5.4, 5.0, 3.9, 3.7, −4.0, −4.6. IR (neat): 2952, 2876, 1746, 1613, 1513, 1461, 1362, 1247, 1182, 1070, 1004, 971, 835, 775, 727, 578 cm^{-1}. MS (ESIpos) *m/z* (%): 949.6 (100 (M+Na)). HRMS (ESI): *m/z* calcd for $C_{52}H_{90}O_8Si_3Na$ $[M+Na]^+$: 949.5836, found: 949.5837.

Analytical and spectroscopic data of the constitutional isomer **24**: $[\alpha]_D^{25}$ = −11.1 (c = 0.27, $CHCl_3$). ^{1}H NMR (600 MHz, $CDCl_3$): *see Table S-4*. ^{13}C NMR (150 MHz, $CDCl_3$): *see Table S-4*. ^{29}Si NMR (119 MHz, $CHCl_3$): δ 20.7, 17.5, 16.5. IR (neat): 2954, 2935, 2877, 1738, 1613, 1514, 1462, 1416, 1378, 1248, 1182, 1137, 1083, 1007, 973, 836, 807, 775, 743 cm^{-1}. MS (ESIpos) *m/z* (%): 949.6 (100 (M+Na)). HRMS (ESI): *m/z* calcd for $C_{52}H_{90}O_8Si_3Na$ $[M+Na]^+$: 949.5836, found: 949.5845.

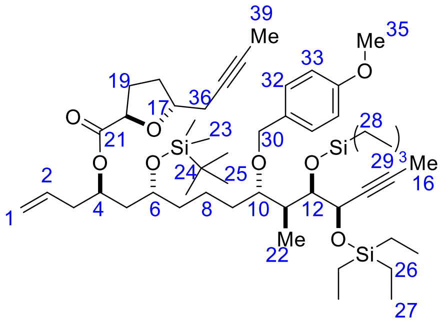

Table S-4. NMR data of compound **24**; arbitrary numbering scheme as shown in the insert

atom n°	^{1}H NMR (600 MHz, CDCl$_3$)					^{13}C NMR (150 MHz, CDCl$_3$)	
	δ [ppm]	m	*J* [Hz]	COSY	NOESY	δ [ppm]	HMBC
1^{cis}	5.06	m	-	2	-	118.1	3
1^{trans}	5.06	m	-	2	-		
2	5.73	ddt	16.3, 10.8, 7.1	1, 3	4	133.5	3, 4
3	2.35	m	-	2, 4	-	39.3	1, 2, 5ab
4	5.03	m	-	3, 5ab	2, 5ab, 6, 23, 23’	71.7	3, 5a
5a	1.70	ddd	14.3, 9.3, 3.0	4, 6	4, 5b, 6	41.1	3, 7
5b	1.55	m	-	4, 6	4, 5a		
6	3.63	m	-	5ab, 7	4, 5a, 8a, 23, 23’, 25	69.4	4, 5b, 7
7	1.41	m	-	6, 8ab	-	38.8	5b, 9
8a	1.47	m	-	7, 8b, 9	8b	21.8	9, 10
8b	1.18	m	-	7, 8a, 9	8a, 10		
9	1.39	m	-	8ab, 10	10, 12, 22	30.3	10, 11
10	3.65	m	-	9, 11	9, 11, 22, 30ab, 32	79.9	9, 11, 12 22, 30ab
11	2.42	m	-	10, 12, 22	10, 12, 22, 30ab, 32	38.1	12, 22
12	3.61	m	-	11	9, 11, 22, 32	76.3	10, 11, 16, 22
13	4.33	m	-	16	12, 26, 27, 28, 29	67.8	11, 12, 16
14	-	-	-	-	-	78.8	12, 13, 16
15	-	-	-	-	-	82.2	12, 16
16	1.83	d	2.2	13	27, 29, 32	3.9	-
17	4.30	qd	6.8, 4.8	18ab, 36ab, 39	18a	78.9	19ab
18a	2.08	ddt	12.5, 8.3, 6.3	17, 18b, 19ab	17, 18b	30.0	19ab, 20
18b	1.76	m	-	17, 18a, 19ab	18a		
19a	2.29	dtd	12.6, 8.4, 6.2	18ab, 20	19b, 20	30.1	17, 18ab, 20
19b	1.98	dddd	12.4, 8.1, 6.7, 5.3	18ab, 20	19a		
20	4.51	dd	8.0, 5.4	19ab	19a	77.6	18ab, 19ab
21	-	-		-	-	172.9	4, 19ab, 20
22	0.94	d	7.0	11	9, 10, 11, 12	10.4	10, 11, 12
23	0.00, 0.01	s	-	-	25	−3.9	23’
23’	0.00, 0.01	s	-	-	25	−4.6	23
24	-	-	-	-		18.2	23, 23’, 25
25	0.86	s	-	-	23, 23’	26.1	-
26	0.71, 0.59	m	-	-	13	5.4, 5.0	-
27	0.98	t	7.9	-	13, 16	7.0	-

28	0.70, 0.60	m	-	-	13, 32	5.4, 5.0	-
29	0.97	t	8.0	-	13, 16	7.2	-
30a	4.50	d	11.0	30b	10, 11, 32	70.1	10, 32
30b	4.35	d	10.6	30a	10, 11, 32		
31	-	-	-	-	-	131.8	30ab, 33
32	7.28	m	-	-	10, 11, 12, 16, 28, 30ab	129.5	30ab
33	6.86	m	-	-	-	113.8	-
34	-	-	-	-	-	159.1	32, 33, 35
35	3.80	s	-	-	-	55.4	-
36a	2.45	m	-	17, 39	-	25.5	18ab, 39
36b	2.34	m	-	17, 39	-		
37	-	-	-	-	-	75.3	17, 36b, 39
38	-	-	-	-	-	77.3	39
39	1.78	t	2.5	17, 36ab	-	3.7	-

Compound 19. Prepared according to the General Procedure using ketone **11** (330 mg, 0.499 mmol), aldehyde **18** (2.14 equiv.), SmI_2 (4 equiv.) and benzaldehyde (3.95 equiv.) at −40 °C as a colorless oil (316 mg, 68% over 2 steps, mixture of constitutional isomers **19**/**20** = 3:1). Analytical and spectroscopic data of **19:** $[\alpha]_D^{25}$ = −31.5 (c = 1.00, $CHCl_3$). ^1H NMR (400 MHz, $CDCl_3$) δ 7.26 (d, *J* = 8.6 Hz, 2H), 6.85 (d, *J* = 8.6 Hz, 2H), 5.85 – 5.71 (m, 1H), 5.09 – 5.00 (m, 2H), 5.00 – 4.90 (m, 1H), 4.53 – 4.44 (m, 2H), 4.37 – 4.25 (m, 3H), 3.82 – 3.70 (m, 4H), 3.68 – 3.58 (m, 2H), 2.48 – 2.18 (m, 6H), 2.12 – 2.02 (m, 1H), 2.01 – 1.91 (m, 1H), 1.82 (d, *J* = 2.2 Hz, 3H), 1.80 – 1.63 (m, 5H), 1.62 – 1.35 (m, 6H), 1.29 – 1.18 (m, 1H), 1.01 – 0.85 (m, 30H), 0.71 – 0.55 (m, 12H), 0.04 (s, 3H), 0.03 (s, 3H). ^{13}C NMR (101 MHz, $CDCl_3$) δ 172.9, 159.0, 134.5, 131.7, 129.4, 117.4, 113.7, 82.2, 79.5, 78.9, 78.7, 77.6, 77.2, 76.2, 75.3, 72.9, 70.0, 68.7, 67.8, 55.4, 42.7, 41.3, 38.1, 35.2, 30.2, 30.1, 29.8, 26.0, 25.5, 21.5, 18.1, 10.4, 7.1, 7.0, 5.4, 4.9, 3.9, 3.7, −4.0, −4.7. IR (neat): 2953, 2876, 1731, 1613, 1514, 1461, 1361, 1247, 1181, 1070, 1003, 968, 835, 775, 739, 675, 575 cm^{-1}. MS (ESIpos) *m/z* (%): 949.6 (100 (M+Na)). HRMS (ESI): *m/z* calcd for $C_{52}H_{90}O_8Si_3Na$ $[M+Na]^+$: 949.5836, found: 949.5841.

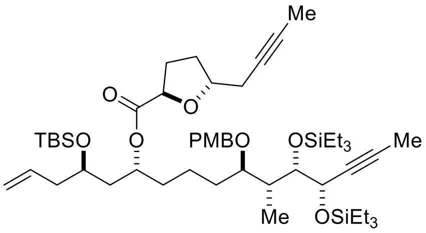

Analytical and spectroscopic data for constitutional isomer **20:** $[\alpha]_D^{20}$ = −3.1 (c = 0.1, $CHCl_3$). ^1H NMR (600 MHz, $CDCl_3$): *see Table S-5*. ^{13}C NMR (150 MHz, $CDCl_3$): *see Table S-5*. IR (neat): 2953, 2935, 2876, 1748, 1613, 1514, 1461, 1417, 1379, 1362, 1300, 1247, 1198, 1181, 1139, 1076, 1040, 1005, 972, 918, 865, 836, 807, 775, 740 cm^{-1}. MS (ESIpos) *m/z* (%): 949.6 (100 (M+Na)). HRMS (ESI): *m/z* calcd for $C_{52}H_{90}O_8Si_3Na$ $[M+Na]^+$: 949.5827, found: 949.5836.

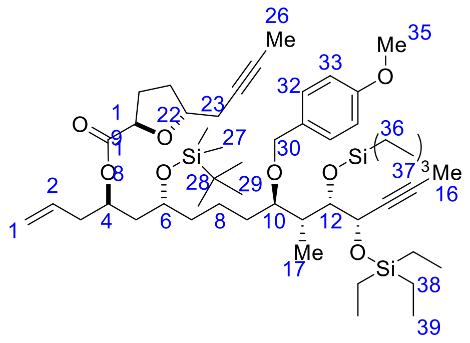

Table S-5. NMR data of compound **20**; arbitrary numbering scheme as shown in the insert

atom n°	^{1}H NMR (600 MHz, $CDCl_3$)					^{13}C NMR (150 MHz, $CDCl_3$)	
	**δ** [ppm]	**m**	***J*** [Hz]	**COSY**	**NOESY**	**δ** [ppm]	**HMBC**
1	5.06	m	-	-	-	117.9	3ab
2	5.73	ddt	16.3, 10.8, 7.1	1, 3ab	-	133.33	1, 3ab, 4
3a	2.37	m	-	2, 4	-	39.13	1, 2, 4, 5ab
3b	2.33	m	-	2, 4	-		
4	5.03	m	-	3ab, 5ab	3ab , 5ab, 6, 27, 27’, 29	71.63	3ab, 5a
5a	1.68	ddd	14.4, 9.1, 3.2	4, 5b, 6	4	40.99	3ab, 7ab
5b	1.59	ddd	14.4, 8.8, 3.3	4, 5a, 6	4		
6	3.65	m	-	5ab	4, 27	69.18	5b, 7b
7a	1.43	m	-	8ab	-	38.5	5ab, 9
7b	1.40	m	-	8ab	-		
8a	1.48	m	-	7ab, 8b, 9	-	21.25	7ab, 9, 10
8b	1.18	m	-	7ab, 8a, 9	-		
9	1.39	m	-	8ab, 10	10, 12, 30b	30.14	7a, 10, 11
10	3.65	m	-	9, 11	9, 11, 17, 30ab, 32	79.68	9, 11, 12, 17, 30ab
11	2.41	m	-	10, 12, 17	10, 12, 17, 30ab	37.96	10, 12, 13, 17
12	3.62	dd	5.9, 4.7	11, 13	9, 11, 13, 17, 32	76.16	10, 11, 13, 16, 17
13	4.34	dq	-	12, 16	12	67.68	11, 12, 16
14	-	-	-	-	-	78.63	12, 13, 16
15	-	-	-	-	-	82.04	12, 13, 16
16	1.83	d	2.2	13	-	3.74	-
17	0.93	d	6.9	11	10, 11, 12	10.24	10, 11, 12
18	-	-	-	-	-	172.76	4, 19, 20ab
19	4.52	dd	8.1, 5.3	20ab	-	77.43	20ab, 21ab, 22
20a	2.28	dtd	12.5, 8.4, 8.3, 6.0	19, 20b, 21ab	-	29.93	19, 21ab, 22

20b	1.98	dddd	12.4, 8.1, 6.7, 5.3	19, 20a, 21ab	22		
21a	2.08	ddt	12.5, 8.2, 6.3	20ab, 21b, 22	22	29.84	19, 20ab, 23b
21b	1.76	-	-	20ab, 21a, 22	22		
22	4.30	qd	6.7, 4.7	21ab, 23ab	20b, 21ab	78.75	19, 20ab, 21b, 23b
23a	2.45	ddq	16.5, 5.1, 2.6	22, 23b, 26	-	25.34	21ab
23b	2.34	m	-	22, 23a, 26	-		
24	-	-	-	-		75.14	22, 23ab, 26
25	-	-	-	-	-	77.13	23b, 26
26	1.78	-	2.6	23ab	-	3.52	-
27	0.01	s	-	-	4, 6	-4.07	27’
27’	0.01	s	-	-	4	-4.81	27
28	-	-	-	-	13, 32	17.99	27, 27’, 29
29	0.87	s	-	-	4	25.9	-
30a	4.50	d	11.0	30b	10, 11, 32	69.89	10, 32
30b	4.35	d	10.6	30a	9, 10, 11, 32		
31	-	-	-	-	-	131.63	30ab, 33
32	7.27	m	-	33	10, 12, 30ab, 33	129.27	30ab
33	6.85	m	-	32	32, 35	113.6	-
34	-	-	-	-	-	158.91	32, 33, 35
35	3.80	s	-	-	33	55.25	-
36	0.63	m	-	-	-	4.79, 5.27	-
37	0.97, 0.98	-	-	-	-	6.84, 6.98	-
38	0.63	m	-	-	-	4.79, 5.27	-
39	0.97, 0.98	-	-	-	-	6.84, 6.98	-

4-Nitrobenzoate ester S7. 3-Nitrobenzaldehyde was added to a solution of ketone **11** (40 mg, 0.061 mmol) in THF (0.2 mL) at RT. After stirring for 30 min and cooling to −20 °C, a solution of SmI_2 (0.1 M in THF, 0.57 mL, 0.057 mmol) was added and stirring continued for 2 h at the same temperature. The mixture was poured into saturated Rochelle salt and was diluted with *tert*-butyl methyl ether (5 mL). The aqueous layer was separated and extracted with *tert*-butyl methyl ether (2 × 5 mL). The combined organic layers were washed with brine, dried over Na_2SO_4 and concentrated to afford the crude alcohol as a yellow oil, which was used without further purification.

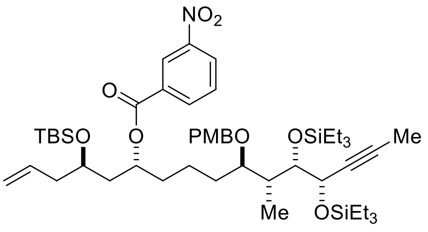

2,6-Lutidine (0.03 mL, 0.3 mmol) and TBSOTf (0.04 mL, 0.2 mmol) were added to a solution of the crude alcohol in CH_2Cl_2 (0.5 mL) at 0 °C. After stirring for 2 h and allowing the mixture to warm to

ambient temperature, the reaction was quenched with sat. NH_4Cl (2 mL). The aqueous layer was extracted with *tert*-butyl methyl ether (3 × 5 mL), and the combined organic layers were washed with brine, dried over Na_2SO_4 and concentrated. The residue was purified by flash chromatography (hexane/*tert*-butyl methyl ether = 10:1) to afford the title compound as a yellow oil (30 mg, 54%). $[\alpha]_D^{25}$ = 7.7 (c = 1.00, $CHCl_3$). ^1H NMR (400 MHz, $CDCl_3$) δ = 8.85 – 8.79 (m, 1H), 8.40 (ddd, *J* = 8.3, 2.4, 1.2 Hz, 1H), 8.33 (dt, *J* = 7.7, 1.4 Hz, 1H), 7.62 (t, *J* = 8.0 Hz, 1H), 7.22 (d, *J* = 8.7 Hz, 2H), 6.83 – 6.75 (m, 2H), 5.81 (ddt, *J* = 17.4, 10.5, 7.1 Hz, 1H), 5.25 (dp, *J* = 9.0, 2.9 Hz, 1H), 5.13 – 5.02 (m, 2H), 4.48 (d, *J* = 10.9 Hz, 1H), 4.36 – 4.28 (m, 2H), 3.87 – 3.80 (m, 1H), 3.78 (s, 3H), 3.73 – 3.65 (m, 1H), 3.56 (dd, *J* = 6.4, 4.3 Hz, 1H), 2.42 (qd, *J* = 6.8, 5.1 Hz, 1H), 2.31 – 2.23 (m, 2H), 1.92 – 1.78 (m, 4H), 1.73 – 1.64 (m, 3H), 1.60 – 1.52 (m, 1H), 1.40 (dt, *J* = 8.5, 4.9 Hz, 2H), 1.33 – 1.25 (m, 1H), 0.98 – 0.90 (m, 21H), 0.88 (s, 9H), 0.66 – 0.57 (m, 12H), 0.01 (s, 3H), −0.03 (s, 3H). ^{13}C NMR (101 MHz, $CDCl_3$) δ = 164.0, 159.1, 148.4, 135.3, 134.3, 132.8, 131.6, 129.6, 129.5, 127.2, 124.5, 117.6, 113.7, 82.2, 79.3, 78.7, 76.4, 74.2, 70.0, 68.6, 67.9, 55.4, 42.6, 41.2, 38.2, 35.3, 29.9, 26.0, 21.7, 18.2, 10.5, 7.1, 7.0, 7.0, 5.4, 4.9, 3.9, −4.0, −4.7. IR (neat): 2954, 2876, 1725, 1615, 1536, 1514, 1462, 1351, 1294, 1250, 1138, 1071, 1005, 836, 775, 742, 720 cm^{-1}. MS (ESIpos) *m/z* (%): 948.5 (100 (M+Na)). HRMS (ESI): *m/z* calcd for $C_{50}H_{83}O_9NSi_3Na$ $[M+Na]^+$: 948.5268, found: 948.5276.

Saponification/Silyl Migration: Alcohol S8. Potassium carbonate (11.9 mg, 0.086 mmol) was added to

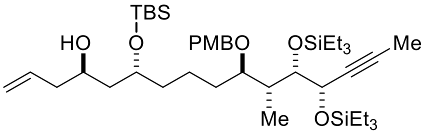

a solution of ester **S7** (29 mg, 0.031 mmol) in a mixture of MeOH (0.15 mL) and THF (0.15 mL) at 0 °C. After removing the ice bath, the mixture was stirred for 13 h at ambient temperature. The reaction was quenched with sat. NH_4Cl (1 mL). The aqueous layer was separated and extracted with *tert*-butyl methyl ether (2 × 5 mL). The combined organic layers were washed with brine, dried over Na_2SO_4 and concentrated. The residue was purified by flash chromatography (hexane/*tert*-butyl methyl ether = 10:1) to afford the title compound as a yellow oil (15 mg, 62%). $[\alpha]_D^{25}$ = 15.9 (c = 1.50, $CHCl_3$). ^1H NMR (400 MHz, $CDCl_3$) δ = 7.30 – 7.26 (m, 2H), 6.88 – 6.82 (m, 2H), 5.74 (ddt, *J* = 17.2, 10.1, 7.2 Hz, 1H), 5.11 – 5.01 (m, 2H), 4.50 (d, *J* = 11.0 Hz, 1H), 4.39 – 4.30 (m, 2H), 4.06 – 3.99 (m, 1H), 3.93 – 3.86 (m, 1H), 3.79 (s, 3H), 3.70 – 3.63 (m, 1H), 3.61 (dd, *J* = 6.0, 4.7 Hz, 1H), 3.04 (br. s, 1H), 2.46 – 2.37 (m, 1H), 2.37 – 2.29 (m, 2H), 1.83 (d, *J* = 2.2 Hz, 3H), 1.57 – 1.51 (m, 2H), 1.51 – 1.38 (m, 4H), 1.36 – 1.29 (m, 2H), 1.01 – 0.92 (m, 21H), 0.90 (s, 9H), 0.69 – 0.58 (m, 12H), 0.10 (s, 3H), 0.08 (s, 3H). ^{13}C NMR (101 MHz, $CDCl_3$) δ = 159.1, 134.9, 131.8, 129.5, 117.4, 113.8, 82.2, 80.0, 78.8, 76.3, 71.1, 70.1, 68.4, 67.8, 55.4, 41.7, 41.4, 38.5, 38.1, 29.9, 26.0, 22.2, 18.1, 10.4, 7.2, 7.0, 5.4, 4.9, 3.9, −4.3, −4.7. IR (neat): 2955, 2928, 2877, 2856, 1514, 1463, 1248, 1137, 1081, 1039, 1006, 838, 807, 737 cm^{-1}. MS (ESIpos) *m/z* (%): 799.5 (100 (M+Na)). HRMS (ESI): *m/z* calcd for $C_{43}H_{80}O_6Si_3Na$ $[M+Na]^+$: 799.5154, found: 799.5159.

General Procedure for Ring Closing Alkyne Metathesis (RCAM). Complex **29** (30 mol%) was dissolved in CH_2Cl_2 and the mixture stirred for 15 min before the resulting red solution was added to a solution of the respective diyne (mixture of constitutional isomers) in toluene (2 µM). The reaction flask attached to a reflux condenser was placed in a preheated oil bath (110°C) and the mixture was stirred until the starting material and transiently formed dimer were consumed (ca. 24 h). After cooling to room temperature, the mixture was filtered through a plug of silica which was carefully rinsed with EtOAc (10 mL). The combined filtrates were concentrated and the residue was purified by flash chromatography (hexane/EtOAc 20:1) to give the mixture comprising the 17-membered and the 19-membered macrocyclic products, which could be separated by flash chromatography.

Macrocycle 25. Prepared according to the General Procedure as a colorless oil (64.8 mg, 77% (**25**+**26**)).

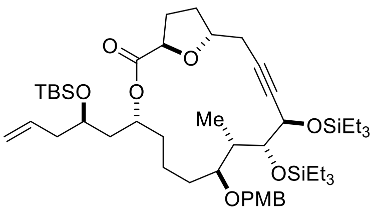

$[\alpha]_D^{25}$ = −66.7 (c = 1.00, $CHCl_3$). ^{1}H NMR (400 MHz, $CDCl_3$) δ = 7.25 (d, J = 8.7 Hz, 2H), 6.86 (d, J = 8.7 Hz, 2H), 5.79 (ddt, J = 18.8, 9.1, 7.1 Hz, 1H), 5.09 – 5.00 (m, 3H), 4.53 – 4.46 (m, 2H), 4.29 – 4.21 (m, 2H), 4.17 – 4.07 (m, 2H), 3.83 – 3.74 (m, 4H), 3.36 (dd, J = 10.2, 3.1 Hz, 1H), 2.44 – 2.11 (m, 7H), 2.07 – 1.96 (m, 1H), 1.90 – 1.75 (m, 2H), 1.67 – 1.53 (m, 2H), 1.53 – 1.23 (m, 5H), 1.00 – 0.86 (m, 27H), 0.76 – 0.49 (m, 15H), 0.04 (s, 6H). ^{13}C NMR (101 MHz, $CDCl_3$) δ = 172.0, 158.9, 134.5, 131.8, 128.9, 117.4, 113.6, 84.7, 80.7, 78.7, 78.7, 78.4, 76.0, 72.9, 69.9, 69.1, 68.1, 55.4, 42.5, 42.3, 37.5, 36.5, 31.7, 27.8, 27.5, 26.0, 25.9, 18.2, 16.9, 10.0, 7.3, 7.1, 5.8, 5.3, −4.1, −4.6. IR (neat): 2952, 2875, 1735, 1614, 1514, 1461, 1384, 1247, 1192, 1076, 1004, 822, 775, 737, 574, 467 cm^{-1}. MS (ESIpos) *m/z* (%): 895.5 (100 (M+Na)). HRMS (ESI): *m/z* calcd for $C_{48}H_{84}O_8Si_3Na$ [M+Na]$^+$: 895.5366, found: 895.5371.

Macrocycle 21. Prepared according to the General Procedure as a colorless oil (60.2 mg, 64% (pure **21**)).

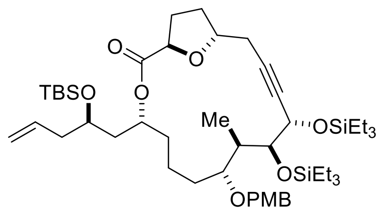

$[\alpha]_D^{25}$ = −2.9 (c = 1.00, $CHCl_3$). ^{1}H NMR (400 MHz, $CDCl_3$) δ 7.28 (d, J = 8.7 Hz, 2H), 6.86 (d, J = 8.7 Hz, 2H), 5.79 (ddt, J = 15.9, 11.5, 7.1 Hz, 1H), 5.10 – 5.01 (m, 3H), 4.53 – 4.46 (m, 2H), 4.31 (d, J = 11.1 Hz, 1H), 4.26 – 4.17 (m, 2H), 4.15 (dd, J = 7.6, 1.5 Hz, 1H), 3.83 – 3.74 (m, 4H), 3.39 (dd, J = 9.9, 2.6 Hz, 1H), 2.47 – 2.28 (m, 3H), 2.28 – 2.06 (m, 5H), 1.87 – 1.68 (m, 3H), 1.57 – 1.35 (m, 5H), 1.35 – 1.23 (m, 1H), 1.00 – 0.85 (m, 27H), 0.75 – 0.49 (m, 15H), 0.03 (s, 3H), 0.01 (s, 3H). ^{13}C NMR (101 MHz, $CDCl_3$) δ 172.5, 158.9, 134.5, 131.7, 129.0, 117.4, 113.7, 84.7, 81.0, 79.2, 79.0, 78.0, 76.2, 72.7, 70.1, 68.8, 68.6, 55.4, 42.6, 41.2, 37.7, 34.8, 31.7, 28.7, 27.5, 26.0, 25.6, 18.2, 16.3, 10.1, 7.3, 7.1, 5.8, 5.4, −4.0, −4.6. IR (neat): 2953, 2875, 1735, 1614, 1514, 1462, 1247, 1195, 1139, 1077, 1060, 1003, 914, 835, 775, 735, 571 cm^{-1}. MS (ESIpos) *m/z* (%): 895.5 (100 (M+Na)). HRMS (ESI): *m/z* calcd for $C_{48}H_{84}O_8Si_3Na$ [M+Na]$^+$: 895.5366, found: 895.5369.

Analytical and spectroscopic data of the 19-membered macrocycle **22**: $[\alpha]_D^{25}$ = +18.9 (c = 1.26, $CHCl_3$).

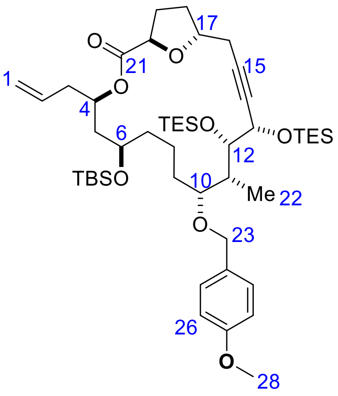

^{1}H NMR (600 MHz, $CDCl_3$) δ *see Table S-6*. ^{13}C NMR (151 MHz, $CDCl_3$) δ *see Table S-6*. IR (neat): 2952, 2935, 2876, 1735, 1514, 1462, 1248, 1137, 1081, 1040, 1005, 836, 776, 739 cm^{-1}. MS (ESIpos) *m/z* (%): 895.5 (100 (M+Na)). HRMS (ESI): *m/z* calcd for $C_{48}H_{84}O_8Si_3Na$ [M+Na]$^+$: 895.5366, found: 895.5370.

Table S-6. NMR data of macrocycle **22**; arbitrary numbering scheme as shown in the insert.*

atom n°	^{1}H NMR (600 MHz, $CDCl_3$)					^{13}C NMR (151 MHz, $CDCl_3$)	
	δ [ppm]	m	*J* [Hz]	COSY	NOESY	δ [ppm]	HMBC
1^{cis}	5.07	m		2, 3	3	118.2	3
1^{trans}	5.07	m		2, 3	3		
2	5.73	ddt	17.4, 10.4, 7.1	1, 3	3, 4	133.4	1, 3, 4
3a	2.38	m		1, 2, 4	1, 2, 5ab	39.3	1, 2, 4, 5ab
3b	2.38	m		1, 2, 4	1, 2, 5ab		
4	4.94	dtd	10.3, 5.9, 1.6	3, 5ab	2, 5ab, 6	72.5	1, 2, 3, 6
5a	1.78	ddd	14.9, 10.3, 1.7	4, 5b, 6	3, 4, 5b, 6, 8b	42.4	3, 4, 7ab
5b	1.58	ddd	14.7, 8.3, 1.6	4, 5a, 6	3, 4, 5a, 6		
6	3.54	ddt	10.6, 8.6, 2.1	5ab, 7ab	4, 5ab, 7ab, 8ab, 9ab, 17	70.8	4
7a	1.50	m		6, 8ab	6, 7b, 8b	39.2	5ab, 8ab
7b	1.32	m		6, 8ab	6, 7a		
8a	1.39	m		7ab, 8b, 9ab	6, 8b, 9a, 11	19.0	6, 7ab, 9ab, 10
8b	1.15	m		7ab, 8a, 9ab	5, 6, 8a, 9a, 11		
9a	1.69	m		8ab, 10	6, 8a, 9b, 10, 23a	28.6	8b, 11
9b	1.49	m		8ab, 10	6, 9a, 10, 22		
10	3.37	dt	9.7, 3.4	9ab, 11	9ab, 11, 12, 13, 22 23ab, 25	79.1	22
11	2.24	m		10, 22	8ab, 10, 12, 13, 22	37.9	9b, 10, 12, 22
12	4.13	dd	8.1, 1.5	13	10, 11, 22, 28	75.8	22
13	4.21	dt	8.2, 2.3	12, 16ab	10, 11, 22	67.6	-
14	-	-				81.4	13, 16ab
15	-	-				84.1	13, 16ab, 17
16a	2.47	ddd	16.1, 5.5, 2.6	13, 16b, 17	16b, 17, 18b	26.5	18ab
16b	2.26	m		13, 16a, 17	16a, 17		
17	4.25	m		16ab, 18ab	6, 16ab, 18ab, 19ab	79.1	-
18a	2.17	m		17, 18b, 19ab	17, 18b, 20	30.8	16ab, 19ab, 20
18b	1.63	m		17, 18a, 19ab	16a, 17, 18a, 20		
19a	2.16	m		18ab, 19b, 20	17, 19b, 20	29.5	17, 18ab, 20

19b	1.98	m		18ab, 19a, 20	17, 19a, 20		
20	4.52	dd	7.4, 4.6	19ab	18ab, 19ab	78.9	-
21	-	-				173.0	4, 19ab, 20
22	0.78	d	6.9	11	8b, 9b, 10, 11, 12, 13	10.1	10, 11, 12
23a	4.48	d	11.0		9a, 10, 25	70.1	10, 25
23b	4.33	d	11.1		10, 25		
24	-	-				131.8	23ab, 26
25	7.26	m			10, 12, 26	128.9	23ab
26	6.85	m			10, 12	113.7	25
27	-	-				159.0	28, 25
28	3.80	s			26	55.4	

* The signals of the TBS- and TES-groups are not listed and appear as follows: ^{1}H NMR (600 MHz, $CDCl_3$) δ 0.98 (t, *J* = 7.9 Hz, 9H), 0.92 (t, *J* = 8.0 Hz, 9H), 0.86 (s, 9H), 0.68 (m, 6H), 0.60 (m, 6H), 0.02 (s, 3H), 0.01 (s, 3H). ^{13}C NMR (151 MHz, $CDCl_3$) δ 26.0, 7.3, 7.0, 5.8, 5.3, −4.1, −4.7.

General Procedure for PMB-Deprotection. DDQ (2 equiv.) was added to a solution of the PMB-ether in a 4:1 mixture of CH_2Cl_2 and pH 7 phosphate buffer (0.04 M) at 0 °C. After stirring at room temperature until the starting material was consumed, the reaction was quenched with a 1:1 mixture of sat. NaS_2O_3 and sat. $NaHCO_3$ solution (4 mL). The aqueous layer was separated and extracted with CH_2Cl_2 (4 x 5 mL). The combined organic layers were washed with half sat. brine, dried over Na_2SO_4, filtered and concentrated in vacuo. Purification of the crude product by flash column chromatography gave the title compound.

Alcohol 33. Prepared according to the General Procedure using PMB-ether **25** (58.0 mg, 66.4 µmol);

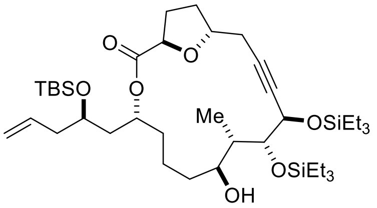

purification by flash column chromatography (hexane/EtOAc 14:1) gave the title compound as a colorless oil (48.2 mg, 96%). $[\alpha]_D^{25}$ = −74.8 (c = 1.00, $CHCl_3$). ^{1}H NMR (400 MHz, $CDCl_3$) δ 5.78 (ddt, *J* = 15.9, 11.8, 7.1 Hz, 1H), 5.16 – 4.99 (m, 3H), 4.45 (dd, *J* = 7.5, 5.4 Hz, 1H), 4.26 – 4.12 (m, 2H), 4.03 (dd, *J* = 8.0, 1.7 Hz, 1H), 3.79 – 3.71 (m, 1H), 3.55 – 3.47 (m, 1H), 2.49 – 2.42 (m, 1H), 2.34 – 2.00 (m, 8H), 1.78 (ddd, *J* = 14.4, 8.0, 3.9 Hz, 1H), 1.72 – 1.32 (m, 8H), 1.01 – 0.91 (m, 18H), 0.88 (s, 9H), 0.82 (d, *J* = 6.9 Hz, 3H), 0.72 – 0.56 (m, 12H), 0.034 (s, 3H), 0.030 (s, 3H). ^{13}C NMR (101 MHz, $CDCl_3$) δ 172.1, 134.5, 117.5, 84.8, 80.6, 79.1, 78.1, 76.7, 73.1, 72.9, 69.0, 67.4, 42.5, 42.3, 39.1, 35.7, 34.0, 32.1, 28.0, 26.0, 25.6, 18.9, 18.2, 11.2, 7.2, 7.1, 5.5, 5.2, −4.1, −4.6. IR (neat): 2952, 2876, 1734, 1460, 1384, 1250, 1194, 1141, 1078, 1003, 915, 836, 774, 736, 544 cm^{-1}. MS (ESIpos) *m/z* (%): 775.5 (100 (M+Na)). HRMS (ESI): *m/z* calcd for $C_{40}H_{76}O_7Si_3Na$ $[M+Na]^+$: 775.4791, found: 775.4795.

Alcohol 30. Prepared according to the General Procedure using PMB-ether **21** (43.3 mg, 49.6 µmol) as

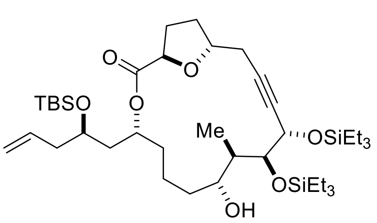

a colorless oil (17.4 mg, 47%). $[\alpha]_D^{25}$ = +2.0 (c = 1.00, $CHCl_3$). ^{1}H NMR (400 MHz, $CDCl_3$) δ 5.78 (ddt, *J* = 18.6, 9.1, 7.1 Hz, 1H), 5.11 – 5.00 (m, 3H), 4.46 (dd, *J* = 7.5, 5.9 Hz, 1H), 4.19 (ddd, *J* = 7.9, 2.7, 1.7 Hz, 1H), 4.11 – 4.02 (m, 2H), 3.82 – 3.74 (m, 1H), 3.52 – 3.43 (m, 1H), 2.82 (d, *J* = 7.9 Hz, 1H), 2.48 (dt, *J* = 16.8, 2.8 Hz, 1H), 2.33 – 2.19 (m, 4H), 2.19 – 2.04 (m, 3H), 1.82 (ddd, *J* = 14.4, 8.0, 3.8 Hz, 1H), 1.76 – 1.66 (m, 1H), 1.65 – 1.52 (m, 4H), 1.52 – 1.39 (m, 2H), 1.37 – 1.24 (m, 1H), 1.01 – 0.89 (m, 21H), 0.88 (s, 9H), 0.74 – 0.61 (m, 12H), 0.04 (s, 3H), 0.03 (s, 3H). ^{13}C NMR (101 MHz, $CDCl_3$) δ 172.1, 134.5, 117.5, 84.8, 80.2, 78.7, 77.9, 76.9, 75.0, 73.0, 69.0, 67.4, 42.5, 42.0, 37.7, 35.6, 34.9, 32.0, 27.8, 26.0, 25.4, 19.7, 18.2, 11.5, 7.12, 7.10, 5.5, 5.3, −4.0, −4.6. IR (neat): 3532, 2953, 2876, 1733, 1461, 1415, 1381, 1252, 1083, 1003, 835, 808, 775, 730, 676 cm^{-1}. MS (ESIpos) *m/z* (%): 775.5 (100 (M+Na)). HRMS (ESI): *m/z* calcd for $C_{40}H_{76}O_7Si_3Na$ $[M+Na]^+$: 775.4791, found: 775.4792.

Enol ether 34. A solution of $[PtCl_2(C_2H_4)]_2$ (0.38 mg, 0.64 µmol) in Et_2O (0.21 mL) was added to a solution

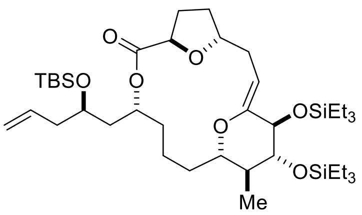

of alcohol **33** (48.2 mg, 64.0 µmol) in Et_2O (0.43 mL). The resulting mixture was stirred for 1.5 h before additional $[PtCl_2(C_2H_4)]_2$ (0.76 mg, 1.28 µmol) was introduced. After stirring for another 3.5 h, the mixture was filtered through a plug of Florisil©, rinsing with *tert*-butyl methyl ether (2 mL). The combined filtrates were concentrated and the crude product was purified by flash chromatography (hexane/EtOAc 19:1) gave the title compound as a colorless oil (37.2 mg, 77%). $[\alpha]_D^{25}$ = −75.2 (c = 0.5, $CHCl_3$). ^{1}H NMR (400 MHz, $CDCl_3$) δ 5.81 (ddt, *J* = 18.7, 9.2, 7.1 Hz, 1H), 5.27 – 5.20 (m, 1H), 5.08 – 5.01 (m, 2H), 4.90 (t, *J* = 7.4 Hz, 1H), 4.53 (dd, *J* = 8.9, 2.5 Hz, 1H), 3.83 – 3.75 (m, 2H), 3.53 – 3.43 (m, 1H), 3.20 (t, *J* = 7.3 Hz, 1H), 3.13 (t, *J* = 10.1 Hz, 1H), 3.02 (ddd, *J* = 10.7, 7.4, 3.0 Hz, 1H), 2.42 – 2.32 (m, 1H), 2.31 – 2.09 (m, 3H), 2.01 – 1.86 (m, 2H), 1.85 – 1.66 (m, 3H), 1.66 – 1.45 (m, 5H), 1.43 – 1.23 (m, 2H), 0.99 – 0.92 (m, 18H), 0.91 – 0.86 (m, 12H), 0.68 – 0.59 (m, 12H), 0.04 (s, 3H), 0.03 (s, 3H). ^{13}C NMR (101 MHz, $CDCl_3$) δ 173.8, 155.7, 134.4, 117.5, 102.2, 79.5, 79.4, 78.7, 76.6, 74.5, 71.5, 68.8, 44.0, 42.5, 39.7, 33.6, 32.9, 30.3, 29.0, 27.8, 26.0, 18.2, 17.3, 15.1, 7.2, 7.2, 5.5, 5.2, −4.0, −4.7. IR (neat): 2953, 2929, 2877, 1728, 1461, 1377, 1255, 1199, 1077, 1004, 968, 913, 834, 808, 775, 726, 688 cm^{-1}. MS (ESIpos) *m/z* (%): 775.5 (100 (M+Na)). HRMS (ESI): *m/z* calcd for $C_{40}H_{76}O_7Si_3Na$ $[M+Na]^+$: 775.4791, found: 775.4795.

Enol ether 31. 2,6-Di-*tert*-butylpyridine (5.2 µL, 23 µmol) and $[PtCl_2(C_2H_4)]_2$ (6.8 mg, 11.5 µmol) were

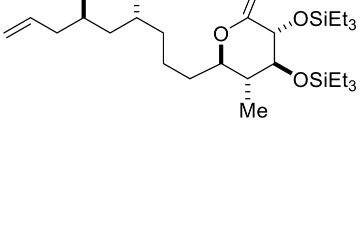

added to a solution of alcohol **30** (17.4 mg, 23.1 µmol) in Et_2O (0.23 mL). The resulting mixture was stirred for 0.5 h and then filtered through a plug of silica, rinsing with *tert*-butyl methyl ether (2 mL). The combined filtrates were concentrated and the residue was

purified by flash chromatography (hexane/EtOAc 24:1) to give the title compound as a colorless oil (4.8 mg, 28%). $[\alpha]_D^{25}$ = −5.8 (c = 0.5, $CHCl_3$). ^{1}H NMR (400 MHz, CD_2Cl_2) δ 5.82 (ddt, *J* = 16.3, 11.0, 7.1 Hz, 1H), 5.09 – 5.02 (m, 2H), 5.01 – 4.93 (m, 1H), 4.61 (t, *J* = 7.4 Hz, 1H), 4.47 (dd, *J* = 9.1, 3.0 Hz, 1H), 4.02 – 3.96 (m, 1H), 3.87 – 3.72 (m, 3H), 3.44 (dd, *J* = 6.3, 2.4 Hz, 1H), 2.81 (ddd, *J* = 13.1, 7.4, 3.3 Hz, 1H), 2.38 – 2.27 (m, 1H), 2.27 – 2.20 (m, 2H), 2.17 – 2.06 (m, 1H), 2.04 – 1.94 (m, 1H), 1.85 (ddd, *J* = 14.2, 7.2, 4.3 Hz, 1H), 1.75 (ddd, *J* = 13.1, 9.8, 7.4 Hz, 1H), 1.68 – 1.49 (m, 6H), 1.48 – 1.41 (m, 1H), 1.35 – 1.21 (m, 2H), 0.99 – 0.90 (m, 21H), 0.89 (s, 9H), 0.65 – 0.56 (m, 12H), 0.06 (s, 3H), 0.06 (s, 3H). ^{13}C NMR (101 MHz, CD_2Cl_2) δ 174.1, 152.9, 135.0, 117.3, 102.2, 79.7, 78.9, 77.0, 76.7, 76.3, 73.1, 69.5, 42.7, 42.5, 41.6, 36.0, 32.5, 31.1, 29.6, 28.0, 26.1, 18.8, 18.3, 16.1, 7.04, 6.99, 5.34, 5.26, −4.13, −4.56. IR (neat): 2955, 2927, 2876, 1729, 1462, 1361, 1258, 1203, 1084, 1006, 970, 884, 835, 796, 775, 743 cm^{-1}. MS (ESIpos) *m/z* (%): 775.5 (100 (M+Na)). HRMS (ESI): *m/z* calcd for $C_{40}H_{76}O_7Si_3Na$ [M+Na]$^+$: 775.4791, found: 775.4794.

Because of significant loss of material upon rigorous purification, the crude product was directly elaborated into **32**.

General Procedure for TBS-Deprotection and Ketalization. A solution of TBAF (1 M in THF, 8 equiv.) was added to enol ether in THF (0.09 M) at 0 °C. After stirring at room temperature until the substrate was consumed, the mixture was filtered through a short pad of silica, which was carefully rinsed with EtOAc (5 mL). The combined filtrates were concentrated to afford the crude alcohol as a colorless oil, which was immediately used in the next step.

A solution of TMSCl (0.06 M in MeOH, 1 equiv.) was added dropwise to a solution of the crude alcohol (1 equiv.) in MeOH (0.06 M) at 0 °C. After stirring at 0 °C for 2 h, the reaction was quenched with saturated $NaHCO_3$ (1 mL). The aqueous layer was separated and extracted with EtOAc (3 × 1 mL). The combined organic layers were concentrated in vacuo and the residue was purified by flash chromatography (EtOAc) gave the title compounds.

Triol 35. Prepared according to the General Procedure as a colorless oil (1.7 mg, 44% over 2 steps). ^{1}H NMR (600 MHz, $CDCl_3$, δ ($CDCl_3$) = 7.24 ppm[13]) δ 5.82 (ddt, *J* = 17.4, 10.4, 7.1 Hz, 1H), 5.21 – 5.15 (m, 1H), 5.14 – 5.09 (m, 2H), 4.57 (dd, *J* = 8.8, 3.4 Hz, 1H), 4.05 (ddt, *J* = 10.1, 5.3, 2.9 Hz, 1H), 3.62 – 3.55 (m, 1H), 3.42 – 3.28 (m, 3H), 3.14 (s, 3H), 2.79 (d, *J* = 3.1 Hz, 1H), 2.34 (s, 1H), 2.31 – 2.15 (m, 4H), 2.14 – 2.07 (m, 1H), 2.03 – 1.96 (m, 1H), 1.96 – 1.87 (m, 2H), 1.86 – 1.80 (m, 1H), 1.78 – 1.66 (m, 3H), 1.63 – 1.43 (m, 5H), 1.43 – 1.36 (m, 2H), 1.34 – 1.24 (m, 1H), 0.94 (d, *J* = 6.5 Hz, 3H). ^{13}C NMR (151 MHz, $CDCl_3$, δ($CDCl_3$) = 77.0 ppm[13]) δ 175.0, 134.7, 117.7, 102.1, 79.7, 76.6, 74.8, 74.1, 73.0, 71.5, 66.7, 47.3, 41.6, 41.1, 38.5, 35.1, 31.0, 30.4, 28.6, 28.2, 25.6, 15.5, 12.5. IR (neat): 3437, 2922, 2854,

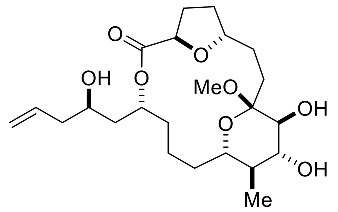

1723, 1463, 1381, 1342, 1211, 1089, 1048, 1002, 974, 911, 821, 773, 735 cm^{-1}. MS (ESIpos) *m/z* (%): 465.2 (100 (M+Na)). HRMS (ESI): *m/z* calcd for $C_{23}H_{38}O_8Na$ $[M+Na]^+$: 465.2459, found: 465.2461.

Triol 32. Prepared analogously as a colorless oil (0.6 mg, 19% over 3 steps). $[\alpha]_D^{25}$ = +34.0 (c = 0.05, CHCl₃).

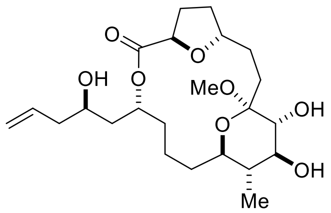

^{1}H NMR (600 MHz, $CDCl_3$, δ($CDCl_3$) = 7.24 ppm[13]) δ 5.82 (ddt, *J* = 17.2, 10.3, 7.1 Hz, 1H), 5.21 (t, *J* = 11.0 Hz, 1H), 5.10 – 5.04 (m, 2H), 4.58 (dd, *J* = 8.2, 2.3 Hz, 1H), 4.07 – 3.99 (m, 1H), 3.54 – 3.48 (m, 1H), 3.42 (t, *J* = 9.5 Hz, 1H), 3.28 (t, *J* = 9.3 Hz, 1H), 3.20 (s, 3H), 3.14 (t, *J* = 9.6 Hz, 1H), 2.99 (d, *J* = 3.6 Hz, 1H), 2.39 – 2.31 (m, 1H), 2.29 (s, 1H), 2.25 – 2.14 (m, 3H), 2.08 (dddd, *J* = 12.3, 9.1, 5.6, 3.6 Hz, 1H), 2.05 – 1.92 (m, 2H), 1.90 (d, *J* = 10.3 Hz, 1H), 1.88 – 1.77 (m, 2H), 1.73 – 1.63 (m, 2H), 1.59 – 1.47 (m, 4H), 1.38 – 1.20 (m, 4H), 0.94 (d, *J* = 6.5 Hz, 3H). ^{13}C NMR (151 MHz, $CDCl_3$ δ($CDCl_3$) = 77.0 ppm[13]) δ 173.5, 134.8, 117.4, 100.7, 77.6, 76.6, 74.9, 74.2, 72.8, 70.1, 66.6, 47.5, 43.0, 42.2, 41.4, 33.9, 31.8, 31.1, 30.4, 27.9, 26.3, 19.6, 12.7. IR (neat): 3424, 2923, 2854, 1728, 1460, 1377, 1260, 1200, 1093, 1033, 1016, 911, 801, 757 cm^{-1}. MS (ESIpos) *m/z* (%): 465.2 (100 (M+Na)). HRMS (ESI): *m/z* calcd for $C_{23}H_{38}O_8Na$ $[M+Na]^+$: 465.2459, found: 465.2461.

Spectral Comparison of the Core Macrocycles with the Isolated Natural Product Formosalide B

Table S-7. Comparison of the ^{13}C NMR spectra of **32** and **35** with the signals corresponding the macrocyclic core of Formosalide B.[13]

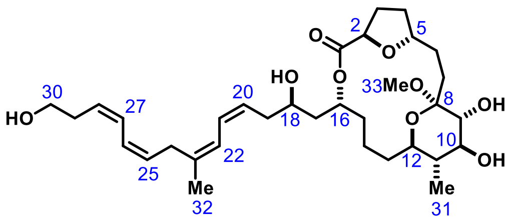

Formosalide B (**2**)

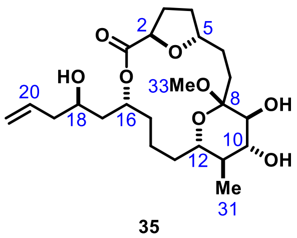

35

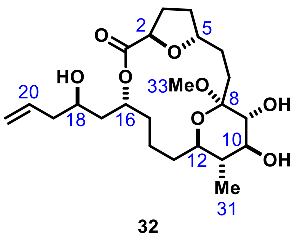

32

Position	δ (2)	δ (35)	Δδ (2–35)	δ (32)	Δδ (2–32)
1	173.4	175.0	−1.6	173.5	−0.1
2	77.6	76.6	1.0	77.6	0
3	26.3	28.2	−1.9	26.3	0
4	31.7	30.4	1.3	31.8	−0.1
5	76.6	79.7	−3.1	76.6	0
6	27.9	28.6	−0.7	27.9	0
7	33.9	25.6	8.3	33.9	0
8	100.7	102.1	−1.4	100.7	0
9	74.2	74.1	0.1	74.2	0
10	74.9	73	1.9	74.9	0
11	42.2	38.5	3.7	42.2	0
12	72.8	74.8	−2.0	72.8	0
13	30.4	31	−0.6	30.4	0
14	19.6	15.5	4.1	19.6	0
15	31.1	35.1	−4.0	31.1	0
16	70.1	71.5	−1.4	70.1	0
17	43	41.1	1.9	43	0
18	67.2	66.7	0.5	66.6	0.6
19	35	41.6	−6.6	41.4	−6.4
20	125.6	134.7	−9.1	134.8	−9.2
31	12.7	12.5	0.2	12.7	0
33	47.5	47.3	0.2	47.5	0

Table S-8. Comparison of the ^{1}H NMR spectra of **32** and **35** with the signals corresponding the macrocyclic core of Formosalide B.[13]

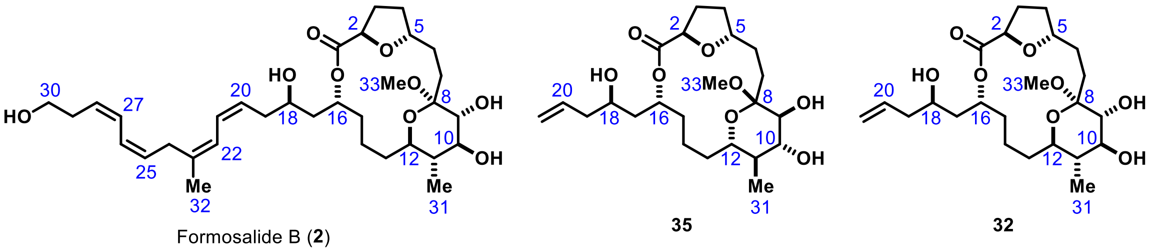

Position	δ (2)	δ (35)	Δδ (2–35)	δ (32)	Δδ (2–32)
2	4.55	4.57	−0.02	4.58	−0.03
3a	2.33	2.22	0.11	2.34	−0.01
3b	1.95	2.22	−0.27	1.95	0.00
4a	2.08	2.1	−0.02	2.08	0.00
4b	1.32	1.3	0.02	1.33	−0.01
5	4.02	4.05	−0.03	4.02	0.00
6a	2.19	1.99	0.20	2.19	0.00
6b	1.70	1.48	0.22	1.72	−0.02
7a	1.80	1.92	−0.12	1.81	−0.01
7b	1.55	1.54	0.01	1.56	−0.01
9	3.28	3.35	−0.07	3.28	0.00
10	3.42	3.35	0.07	3.42	0.00
11	1.34	1.58	−0.24	1.33	0.01
12	3.13	3.35	−0.22	3.14	−0.01
13a	1.83	1.72	0.11	1.84	−0.01
13b	1.55	1.41	0.14	1.55	0.00
14a	2.01	1.71	0.30	2.02	−0.01
14b	1.28	1.42	−0.14	1.28	0.00
15a	1.51	1.71	−0.21	1.51	−0.01
15b	1.27	1.54	−0.27	1.26	0.01
16	5.2	5.18	0.02	5.21	−0.01
17a	1.67	1.83	−0.16	1.67	0.00
17b	1.57	1.48	0.09	1.55	0.02
18	3.50	3.59	−0.09	3.50	0.00
19	2.32	2.22	0.10	2.20	0.12
31	0.94	0.94	0.00	0.94	0.00
33	3.20	3.14	0.06	3.20	0.00

Table S-9. Comparison of relevant ^{13}C NMR data of ***2-epi*-5-*epi*-35** with the signals corresponding the macrocyclic core of Formosalide B, which show a clear mismatch.[13]

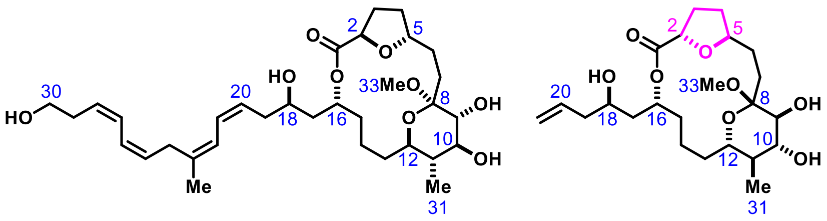

position	δ_H (ppm; *J* in Hz)		
	**Formosalide B**	***2-epi*-5-*epi*-35**	**Δδ**
16	5.20 (br t; 10.1)	5.16 (m)	+0.04
2	4.55 (dd; 8.1, 2.1)	4.55 (dd, 8.3, 5.0)	-
5	4.02 (m)	4.15 (m)	–0.13
18	3.50 (m)	3.51 (m)	–0.01
10	3.42 (dd, 9, 9.7)	3.41 (t, 9.5)	+0.01
9	3.28 (d, 9)	3.32 (t, 9.6)	–0.04
12	3.13 (t, 9.1)	3.09 (t, 9.6)	+0.04

position	δ_C (ppm)		
	**Formosalide B**	***2-epi*-5-*epi*-35**	**Δδ**
8	100.7	101.8	–1.1
2	77.6	77.3	+0.3
5	76.6	79.1	–2.5
10	74.9	75.1	–0.2
9	74.2	74.2	0.0
12	72.8	74.9	–2.1
16	70.1	71.6	–1.5
18	67.2	67.3	–0.1

Synthesis of the Side Chain

(*Z*)-5-((*tert*-Butyldimethylsilyl)oxy)pent-2-en-1-ol (S9). Sodium borohydride (178 mg, 4.70 mmol) was

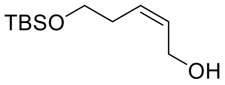

added to a solution of $Ni(OAc)_2 \cdot 4H_2O$ (1.17 g, 4.70 mmol) in MeOH (24 mL) at 0 °C. The mixture was stirred at room temperature for 5 min, followed by addition of ethylenediamine (0.63 mL, 9.4 mmol). After stirring for another 5 min, a solution of 5-((*tert*-

butyldimethylsilyl)oxy)pent-2-yn-1-ol (4.04 g, 18.8 mmol)[10] in MeOH (8 mL) was added. The resulting mixture was stirred under H_2 atmosphere for 20 h. After filtration through a pad of Celite, which was carefully rinsed with CH_2Cl_2, the combined filtrates were concentrated. The residue was re-dissolved in Et_2O (20 mL), the organic phase was washed with water (2 x 15 mL), dried over $MgSO_4$, and concentrated in vacuo. Purification of the residue by flash chromatography (hexane/EtOAc 4:1) gave the title compound as a colorless oil (3.45 g, 85%). ^{1}H NMR (400 MHz, $CDCl_3$) δ 5.89 – 5.75 (m, 1H), 5.62 – 5.53 (m, 1H), 4.14 (dd, *J* = 6.9, 1.2 Hz, 2H), 3.64 (t, *J* = 6.1 Hz, 2H), 2.34 (dtd, *J* = 7.6, 6.1, 1.4 Hz, 2H), 2.02 (brs, 1H), 0.89 (s, 9H), 0.06 (s, 6H). ^{13}C NMR (101 MHz, $CDCl_3$) δ 130.9, 129.8, 62.2, 58.0, 30.9, 26.0, 18.5, −5.4. IR (neat): 3332, 2954, 2929, 2885, 2857, 1472, 1388, 1361, 1254, 1093, 1036, 1004, 926, 833, 812, 773, 738, 662 cm^{-1}. MS (ESIpos) *m/z* (%): 239.1 (100 (M+Na)). HRMS (ESI): *m/z* calcd for $C_{11}H_{24}O_2SiNa$ [M+Na]$^+$: 239.1438, found: 239.1438. The analytical data are in accordance with literature values.[14]

(*Z*)-5-((*tert*-Butyldimethylsilyl)oxy)pent-2-enal (44). A solution of alcohol **S9** (3.30 g, 15.3 mmol) in

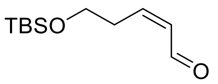

CH_2Cl_2 (4.4 mL) was added to a solution of Dess-Martin periodinane (7.25 g, 17.1 mmol) in CH_2Cl_2 (134 mL) at room temperature. After stirring for 1.5 h, the reaction was quenched with saturated $NaHCO_3$ (70 mL) and saturated $Na_2S_2O_3$ (70 mL). The aqueous layer was separated and extracted with CH_2Cl_2 (100 mL). The combined organic layers were dried over $MgSO_4$, filtered and concentrated in vacuo. The crude product was used in the next step without further purification as the use of silica gel column chromatography resulted in isomerization. Analytical data recorded from the crude product: ^{1}H NMR (400 MHz, $CDCl_3$) δ 10.06 (d, *J* = 8.0 Hz, 1H), 6.69 (dt, *J* = 11.2, 8.0 Hz, 1H), 6.05 (ddt, *J* = 11.2, 8.0, 1.5 Hz, 1H), 3.77 (t, *J* = 6.1 Hz, 2H), 2.81 (dtd, *J* = 8.0, 6.1, 1.5 Hz, 2H), 0.88 (s, 9H), 0.05 (s, 6H). ^{13}C NMR (101 MHz, $CDCl_3$) δ 191.4, 149.7, 131.8, 61.6, 31.6, 26.0, 18.4, −5.2. IR (neat): 2955, 2929, 2885, 2857, 1685, 1472, 1464, 1385, 1361, 1257, 1127, 1100, 1007, 929, 836, 812, 777 cm^{-1}. MS (ESIpos) *m/z* (%): 237.1 (100 (M+Na)). HRMS (ESI): *m/z* calcd for $C_{11}H_{22}O_2SiNa$ [M+Na]$^+$: 237.1281, found 237.1283.

(*Z*)-4-Iodo-3-methylbut-3-en-1-yl 4-methylbenzenesulfonate (S10). Et_3N (0.72 g, 7.1 mmol) and tosyl

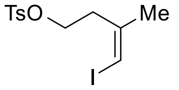

chloride (0.67 g, 3.5 mmol) were added to a solution of (*Z*)-4-Iodo-3-methylbut-3-en-1-ol (**42**, 0.50 g, 2.4 mmol)[11] in CH_2Cl_2 (10 mL) at 0 °C. After removing the ice bath, the resulting mixture was stirred for 6 h at room temperature. The reaction was quenched with saturated $NaHCO_3$ solution (10 mL). The aqueous layer was separated and extracted with CH_2Cl_2 (2 x 10 mL). The combined organic layers were washed with brine, dried over Na_2SO_4, filtrated and concentrated using a rotary evaporator (350 mbar) keeping the temperature of the water bath temperature ≤ 35 °C. The residue was purified by flash chromatography (hexane/*tert*-butyl methyl ether = 10:1) to give the title compound as a clear colorless oil (0.67 g, 78%). ^{1}H NMR (400 MHz, $CDCl_3$) δ 7.82 – 7.78 (m, 2H), 7.37 –

7.33 (m, 2H), 5.98 (q, J = 1.5 Hz, 1H), 4.12 (t, J = 6.9 Hz, 2H), 2.56 (t, J = 6.9 Hz, 2H), 2.45 (s, 3H), 1.86 (d, J = 1.5 Hz, 3H). IR (film): 3064, 2959, 2915, 1598, 1438, 1357, 1188, 1173, 962, 898 cm^{-1}. HRMS (ESI): *m/z* calcd for $C_{12}H_{15}O_3ISNa$ $[M+Na]^+$: 388.9678, found: 388.9678.

Phosphonium salt 43. Na I (1.3 g, 8.5 mmol) was added to a solution of tosylate S8 (0.62 g, 1.7 mmol) in acetone (17 mL). The resulting mixture was stirred at 60°C (bath temperature) for 5 h. After reaching ambient temperature, all volatile materials were evaporated and the residue directly used in the next step.

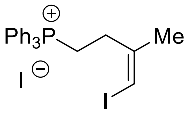

A solution of crude (*Z*)-1,4-diiodo-2-methylbut-1-ene (**S11**) and PPh_3 (4.4 g, 16.9 mmol) in MeCN (14 mL) was stirred at reflux temperature for 24 h. After reaching room temperature, the suspension was poured into hexane causing the formation of a white precipitate (20 mL). The supernatant hexane layer was repeatedly extracted with MeCN (20 mL). The combined MeCN layers were washed with hexane (3 × 10 mL). The MeCN phase was concentrated and the residue triturated with hexane (3 × 5 mL) and pentane (2 × 10 mL) until TLC control showed that all PPh_3 had been removed. The remaining solid material consisted of the title compound (0.81 g, 82%) which was used in the next step without further purification. White solid; mp = 184−186 °C; ^1H NMR (400 MHz, $[D_6]$-DMSO) δ 7.94 − 7.76 (m, 15H), 6.23 − 6.21 (m, 1H), 3.71 − 3.64 (m, 2H), 2.44 − 2.37 (m, 2H), 1.95 (d, J = 1.4 Hz, 3H). ^{13}C NMR (101 MHz, $[D_6]$-DMSO) δ 144.6, (d, J = 16.8 Hz), 135.0 (d, J = 3.1 Hz), 133.7 (d, J = 10.1 Hz), 130.3 (d, J = 12.4 Hz), 118.5, 117.7, 78.4, 31.2 (d, J = 2.8 Hz), 18.3 (d, J = 49 Hz). IR (neat): 3366, 3190, 3052, 3027, 3007, 2960, 2869, 2252, 1660, 1435, 1396, 1109 cm^{-1}. HRMS (ESI): *m/z* calcd for $C_{23}H_{23}IP$ $[M-I]^+$: 457.0577, found: 457.0573.

Diene 45. *This reaction has to be carried out in the dark*. A solution of LiHMDS (0.46 M in THF, 1.9 mL, 0.86 mmol) was added to a solution of phosphonium salt **43** (0.50 g, 0.86 mmol) in THF (10 mL) at −78 °C, and the resulting mixture was stirred for 2 h at this temperature. After addition of DMPU (0.17 mL, 1.5 mmol) and a solution of crude aldehyde **$$** (290 mg, 1.40 mmol) in THF (2.0 mL) the resulting mixture was stirred for another 2 h at –78°C. The reaction was quenched with water (5.0 mL) and the resulting mixture allowed to warm to room temperature. The aqueous layer was separated and extracted with hexane (2 × 10 mL). The combined organic phases were washed with brine (10 mL), dried over Na_2SO_4 and concentrated. The residue was diluted with cold hexane and then filtered through a cotton plug. After concentrating, the residue was purified by flash column chromatography (SiO_2 was pre-treated with 5 % Et_3N in hexane before the crude material was loaded on top of the column; eluent: Et_3N/EtOAc/hexane 1:1:100) to afford the title compound (198 mg, 59%) as a colorless oil. ^1H NMR (400 MHz, CD_2Cl_2) δ 6.51 – 6.31 (m, 2H), 5.90 (s, 1H), 5.61 – 5.53 (m, 1H), 5.42 – 5.35 (m, 1H), 3.65 (t, J = 6.7 Hz, 2H), 3.11 (d, J = 7.5 Hz, 2H), 2.48 – 2.24 (m, 2H), 1.87 (d, J = 1.4 Hz, 3H), 0.89 (s, 9H), 0.05 (s,

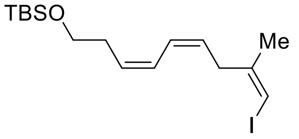

6H). ^{13}C NMR (101 MHz, CD_2Cl_2) δ 146.9, 129.8, 127.3, 126.1, 125.5, 74.5, 63.2, 37.9, 31.9, 26.2, 23.5, 18.8, –5.0. IR (film): 3037, 3005, 2953, 2928, 2856, 1471, 1437, 1385, 1255, 1098, 836 cm^{-1}. HRMS: not detected (decomp.)

Alkenylstannane 46. *This reaction has to be carried out in the dark*. Hexamethylditin (0.24 g, 0.72 mmol) and $[(Ph_3P)_2PdCl_2]$ (17 mg, 0.024 mmol) were added to a solution of alkenyl iodide **45** (0.19 g, 0.48 mmol) in THF (5 mL) at room temperature and the mixture was stirred for 20 h at 35 °C (*Note: the temperature must never exceed 40 °C to avoid double bond isomerization*). After cooling to room temperature, the mixture was concentrated and the residue was purified by flash chromatography (Et_3N/EtOAc/hexane 1:1:98) to give the title compound as a colorless oil (0.16 g, 79%). ^{1}H NMR (600 MHz, CD_2Cl_2) δ *see Table S-10.* ^{13}C NMR (150 MHz, CD_2Cl_2) δ *see Table S-10*. IR (film): 2955, 2928, 2857, 1609, 1471, 1463, 1437, 1253, 1096, 834, 773 cm^{-1}. HRMS (ESI): *m/z* calcd for $C_{19}H_{38}OSiSnNa$ $[M+Na]^+$: 453.1606, found: 453.1606.

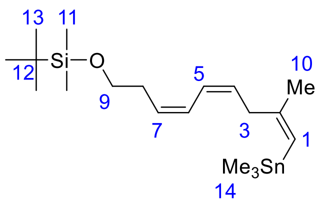

Table S-10. NMR data of alkenylstannane **46**; arbitrary numbering scheme as shown in the insert.

atom n°	^{1}H NMR (600 MHz, CD_2Cl_2)					^{13}C NMR (150 MHz, CD_2Cl_2)	
	δ [ppm]	m	*J* [Hz]	COSY	NOESY	δ [ppm]	HMBC
1	5.55	m			10	125.1	3, 10, 14
2	-	-				153.7	1, 3, 4, 10
3	2.96	m		4	6	38.3	1, 4, 5, 10
4	5.41	m		3, 5	5	129.9	3
5	6.36	m		4, 6	4, 6, 8	124.7	3, 7
6	6.37	m		5, 7	3, 5, 7	124.9	4, 8
7	5.55	m		6, 8	6	128.7	8, 9
8	2.42	qd	6.8, 1.4	7, 9	7, 9	31.3	6, 7, 9
9	3.66	t	6.8	8	8	62.6	7, 8
10	1.85	d	1.5		1	25.7	1, 3
11	0.06	s				−5.6	
12	-	-				18.2	11, 13
13	0.90	s				25.7	
14	0.16	s				−9.0	

Completion of the Total Synthesis of the Formosalides

Aldehyde 36. 2,6-Lutidine (13 µL, 0.11 mmol) and OsO_4 (0.2 M in CH_2Cl_2, 8.6 µL, 1.7 µmol) were added

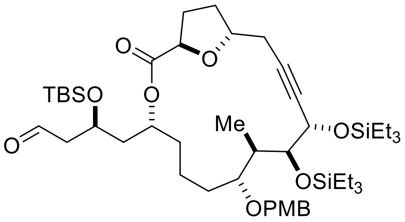

to a solution of alkyne **30** (50 mg, 57 µmol) in a 3:1 mixture of 1,4-dioxane and water (400 µL). After the color of the mixture changed to dark grey, sodium periodate (49 mg, 0.23 mmol) was added and stirring was continued for 3 h at room temperature. The reaction was diluted with water (2 mL) and CH_2Cl_2 (2 mL). The aqueous layer was separated and extracted with CH_2Cl_2 (3 x 2 mL). The combined organic layers were washed with brine (2 mL), dried over Na_2SO_4, filtered and concentrated in vacuo to yield the crude aldehyde **36** as a colorless oil (43.9 mg, 88%). The crude aldehyde was used in the next step without further purification. An aliquot was purified by flash chromatography (hexane/EtOAc 7:1 to 5:1) for analytical purposes. $[\alpha]_D^{25}$ = +4.0 (c = 1.68, $CHCl_3$). ^1H NMR (400 MHz, $CDCl_3$) δ = 9.83 – 9.76 (m, 1H), 7.32 – 7.23 (m, 2H), 6.90 – 6.82 (m, 2H), 5.07 – 4.98 (m, 1H), 4.53 – 4.45 (m, 2H), 4.32 (d, *J* = 11.2 Hz, 1H), 4.27 – 4.07 (m, 4H), 3.81 (s, 3H), 3.39 (dd, *J* = 10.0, 3.7 Hz, 1H), 2.62 – 2.26 (m, 5H), 2.23 – 2.06 (m, 3H), 1.89 (ddd, *J* = 13.5, 8.5, 4.3 Hz, 1H), 1.84 – 1.61 (m, 3H), 1.59 – 1.35 (m, 4H), 1.32 – 1.27 (m, 1H), 1.01 – 0.83 (m, 27H), 0.75 – 0.48 (m, 15H), 0.07 – 0.01 (m, 6H). ^{13}C NMR (101 MHz, $CDCl_3$) δ = 201.7, 172.5, 158.9, 131.8, 128.9, 113.7, 84.6, 81.0, 79.2, 79.0, 78.0, 76.2, 72.1, 70.1, 68.5, 65.6, 55.4, 51.4, 42.4, 37.7, 34.8, 31.7, 28.7, 27.4, 25.9, 25.6, 18.1, 16.5, 10.1, 7.3, 7.1, 5.8, 5.4, −4.3, −4.6. IR (neat): 2953, 2934, 2876, 1731, 1514, 1461, 1415, 1383, 1301, 1248, 1201, 1140, 1078, 1062, 1005, 968, 836, 777, 740 cm^{-1}. MS (ESIpos) *m/z* (%): 897.5 (100 (M+Na)). HRMS (ESI): *m/z* calcd for $C_{47}H_{82}O_9Si_3Na$ $[M+Na]^+$: 897.5159, found: 897.5168.

***Z*-Alkenyl iodide 38**. NaHMDS (23.3 mg, 127 µmol) was added to a solution of iodomethyl-

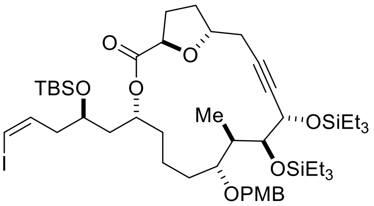

triphenylphosphonium iodide (77 mg, 0.14 mmol) in THF (1 mL). Upon stirring at room temperature for 10 min, the color of the solution changed to bright yellow. After cooling to −78°C, a 1:4 mixture of DMPU and THF (1.0 mL) was added, followed by the dropwise addition of crude aldehyde **36** (43.9 mg, 57.2 µmol) in THF (1 mL). The resulting mixture was stirred for 4 h at the same temperature before the reaction was quenched with H_2O (4 mL) and the mixture warmed to room temperature. The aqueous layer was separated and extracted with EtOAc (3 x 6 mL). The combined organic layers were washed with brine (2 mL), dried over Na_2SO_4, filtered and concentrated in vacuo. Purification of the residue by flash chromatography (hexane/EtOAc/Et_3N 100:10:1) gave the title compound (30.2 mg, 53%, *Z/E* > 20:1) as a colorless oil; a second fraction consisted of a mixture of epimeric epoxides **37** (4.4 mg, 9%) as a colorless oil. Spectral and analytical

data of compound **38**: $[\alpha]_D^{20}$ = −17.5 (c = 0.2, CH_2Cl_2). ^{1}H NMR (600 MHz, $CDCl_3$): δ 7.30 – 7.27 (m, 2H), 6.88 – 6.85 (m, 2H), 6.31 (dt, *J* = 7.4, 1.4 Hz, 1H), 6.24 (app. q, *J* = 6.9 Hz, 1H), 5.09 – 5.03 (m, 1H), 4.53 – 4.48 (m, 2H), 4.30 (d, *J* = 11.0 Hz, 1H), 4.24 (dt, *J* = 7.6, 2.7 Hz, 1H), 4.22 – 4.17 (m, 1H), 4.14 (dd, *J* = 7.5, 1.6 Hz, 1H), 3.89 – 3.84 (m, 1H), 3.81 (s, 3H), 3.39 (ddd, *J* = 9.6, 4.1, 1.7 Hz, 1H), 2.42 (ddd, *J* = 16.3, 7.3, 2.9 Hz, 1H), 2.36 (ddd, *J* = 16.4, 5.1, 2.7 Hz, 1H), 2.35 – 2.29 (m, 3H), 2.24 – 2.16 (m, 1H), 2.16 – 2.07 (m, 2H), 1.82 – 1.67 (m, 3H), 1.55 – 1.37 (m, 5H), 1.35 – 1.19 (m, 1H), 0.96 (t, *J* = 7.9 Hz, 9H), 0.90 (t, *J* = 7.9 Hz, 9H), 0.87 (s, 9H), 0.73 (d, *J* = 6.8 Hz, 3H), 0.68 (qd, *J* = 7.9, 3.1 Hz, 6H), 0.64 – 0.52 (m, 6H), 0.05 (s, 3H), 0.02 (s, 3H). ^{13}C NMR (151 MHz, $CDCl_3$): δ 172.5, 158.9, 137.4, 131.7, 129.0, 113.7, 84.7, 84.7, 81.0, 79.2, 79.0, 78.0, 76.3, 72.6, 70.1, 68.6, 68.0, 55.5, 43.0, 41.4, 37.7, 34.8, 31.7, 28.7, 27.4, 26.0, 25.7, 18.2, 16.3, 10.2, 7.3, 7.1, 5.8, 5.4, 1.2, −4.1, −4.7. IR (film): 2954, 2932, 2876, 2017, 1736, 1514, 1462, 1249, 1077 cm^{-1}. MS (ESIpos) *m/z* (%): 1021.4.5 (100 (M+Na)). HRMS (ESI): m/z calcd for $C_{48}H_{83}IO_8Si_3Na$ [M+Na]$^+$: 1021.4333, found: 1021.4339.

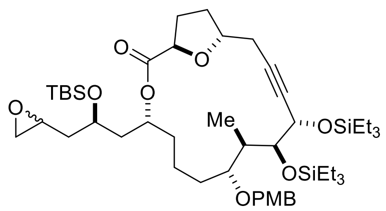

Analytical and spectroscopic data of epoxide **37**. ^{1}H NMR (600 MHz, CD_2Cl_2, major epimer): δ 7.33 – 7.27 (m, 2H), 6.91 – 6.84 (m, 2H), 5.05 (dq, *J* = 9.0, 4.5 Hz, 1H), 4.52 (d, *J* = 11.0 Hz, 1H), 4.46 (dd, *J* = 7.2, 5.9 Hz, 1H), 4.30 (d, *J* = 10.9 Hz, 1H), 4.26 (dtd, *J* = 7.7, 2.7, 1.3 Hz, 1H), 4.15 (tdd, *J* = 9.6, 6.7, 4.4 Hz, 1H), 4.10 (dt, *J* = 7.6, 1.2 Hz, 1H), 3.97 – 3.88 (m, 1H), 3.79 (s, 3H), 3.40 (ddd, *J* = 10.1, 4.3, 1.7 Hz, 1H), 3.00 (dtd, *J* = 7.0, 4.4, 2.6 Hz, 1H), 2.71 (dd, *J* = 5.2, 4.0 Hz, 1H), 2.42 – 2.39 (m, 2H), 2.37 (ddd, *J* = 8.1, 3.9, 2.9 Hz, 1H), 2.29 (ddd, *J* = 10.1, 6.8, 1.7 Hz, 1H), 2.20 – 2.08 (m, 3H), 1.96 – 1.88 (m, 1H), 1.86 – 1.76 (m, 1H), 1.76 – 1.66 (m, 3H), 1.59 (ddd, *J* = 10.2, 6.9, 3.5 Hz, 1H), 1.53 – 1.45 (m, 3H), 1.44 – 1.40 (m, 1H), 1.27 – 1.25 (m, 1H), 0.97 (t, *J* = 8.0 Hz, 18H), 0.88 (s, 9H), 0.73 (d, *J* = 7.0 Hz, 3H), 0.68 – 0.55 (m, 12H) 0.04 (s, 3H), 0.02 (s, 3H). ^{1}H NMR (600 MHz, CD_2Cl_2, minor epimer): δ 7.33 – 7.27 (m, 2H), 6.91 – 6.84 (m, 2H), 5.04 – 4.97 (m, 1H), 4.52 (d, *J* = 11.0 Hz, 1H), 4.46 (dd, *J* = 7.2, 5.9 Hz, 1H), 4.30 (d, *J* = 10.9 Hz, 1H), 4.26 (dtd, *J* = 7.7, 2.7, 1.3 Hz, 1H), 4.15 (tdd, *J* = 9.6, 6.7, 4.4 Hz, 1H), 4.10 (dt, *J* = 7.6, 1.2 Hz, 1H), 3.97 – 3.88 (m, 1H), 3.79 (s, 3H), 3.40 (ddd, *J* = 10.1, 4.3, 1.7 Hz, 1H), 2.99 – 2.92 (m, 1H), 2.73 (dd, *J* = 5.2, 3.9 Hz, 1H), 2.44 (dd, *J* = 5.2, 2.7 Hz, 1H), 2.42 – 2.37 (m, 1H), 2.37 – 2.31 (m, 1H), 2.29 (ddd, *J* = 10.1, 6.8, 1.7 Hz, 1H), 2.20 – 2.08 (m, 3H), 1.96 – 1.88 (m, 1H), 1.86 – 1.76 (m, 1H), 1.76 – 1.69 (m, 1H), 1.66 – 1.63 (m, 3H), 1.53 – 1.44 (m, 4H), 1.27 – 1.25 (m, 1H), 0.97 (t, *J* = 8.0 Hz, 18H), 0.88 (s, 9H), 0.73 (d, *J* = 7.0 Hz, 3H), 0.68 – 0.55 (m, 12H) 0.06 (s, 3H), 0.03 (s, 3H). ^{13}C NMR (151 MHz, CD_2Cl_2, major epimer): δ 172.0, 158.9, 131.5, 129.0, 113.5, 85.0, 80.2, 79.0, 78.9, 77.9, 76.1, 72.1, 69.9, 68.4, 67.3, 55.1, 48.5, 46.4, 41.5, 40.8, 37.5, 34.4, 31.8, 28.3, 27.0, 25.6, 25.3, 17.8, 15.9, 9.6, 6.9, 6.7, 5.6, 5.1, −4.7, −5.2.^{13}C NMR (151 MHz, CD_2Cl_2, minor epimer): 172.0, 158.9, 131.5, 128.9, 113.4, 85.0, 80.2, 79.0, 78.9, 77.9, 76.1, 72.0, 69.9, 68.4, 67.5, 55.1, 49.0, 47.2, 42.1, 40.7, 37.5, 34.4, 31.8, 28.3, 26.9, 25.6, 25.3, 17.8, 16.1, 9.6, 6.9, 6.7, 5.6, 5.1, −4.8, −5.0. IR (film):

2960, 2925, 2874, 2854, 1731, 1514, 1462, 1257, 1077, 1011, 793, 741 cm^{-1}. MS (ESIpos) *m/z* (%): 911.5 (100 (M+Na)). HRMS (ESI): m/z calcd for $C_{48}H_{84}O_9Si_3Na$ [M+Na]$^+$: 911.5315, found: 911.5316.

Alcohol 39. Prepared according to the General Procedure for PMB-deprotection from PMB-ether **38**.

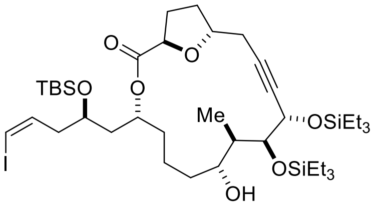

Purification by flash column chromatography (hexane/EtOAc 10:1) afforded a colorless oil (16.3 mg, 62 %). $[\alpha]_D^{25}$ = −5.5 (c = 0.44, $CHCl_3$). ^{1}H NMR (400 MHz, $CDCl_3$) δ 6.32 (dt, *J* = 7.4, 1.2 Hz, 1H), 6.26 (q, *J* = 6.5 Hz, 1H), 5.08 (tt, *J* = 8.0, 3.8 Hz, 1H), 4.48 (dd, *J* = 7.5, 6.0 Hz, 1H), 4.20 (ddd, *J* = 7.9, 2.7, 1.7 Hz, 1H), 4.11 – 4.01 (m, 2H), 3.92 – 3.83 (m, 1H), 3.48 (t, *J* = 6.1 Hz, 1H), 2.82 (d, *J* = 7.7 Hz, 1H), 2.48 (dt, *J* = 16.8, 2.7 Hz, 1H), 2.36 – 2.21 (m, 4H), 2.18 – 2.05 (m, 3H), 1.81 (ddd, *J* = 14.3, 8.1, 4.0 Hz, 1H), 1.77 – 1.67 (m, 1H), 1.62 (ddd, *J* = 14.4, 8.0, 4.2 Hz, 1H), 1.58 – 1.55 (m, 3H), 1.50 – 1.40 (m, 2H), 1.38 – 1.23 (m, 1H), 1.02 – 0.90 (m, 21H), 0.88 (s, 9H), 0.74 – 0.61 (m, 12H), 0.06 (s, 3H), 0.05 (s, 3H). ^{13}C NMR (100 MHz, $CDCl_3$): δ 172.1, 137.4, 84.8, 84.7, 80.2, 78.7, 77.9, 76.9, 75.1, 72.9, 68.1, 67.4, 43.0, 42.3, 37.7, 35.6, 34.9, 32.1, 27.9, 26.0, 25.4, 19.8, 18.2, 11.5, 7.1, 7.1, 5.5, 5.3, −4.1, −4.6. IR (film): 2953, 2926, 2875, 2855, 1736, 1461, 1416, 1379, 1256, 1196, 1081, 1006 cm^{-1}. MS (ESIpos) *m/z* (%): 901.4 (100 (M+Na)). HRMS (ESI): m/z calcd for $C_{40}H_{75}O_7ISi_3Na$ [M+Na]$^+$: 901.3758, found: 901.3762.

Ketal 40. 2,6-Di-*tert*-butyl pyridine (5.2 µL, 23 µmol) and $[PtCl_2(C_2H_4)]_2$ (6.8 mg, 12 µmol) were added

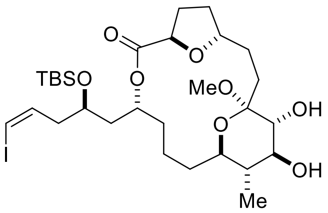

to a solution of alcohol **39** (17 mg, 19 µmol) in Et_2O (0.76 mL). The resulting mixture was stirred for 1.5 h before it was filtered through a plug Florisil©, rinsing with Et_2O (0.8 mL). The combined filtrates were evaporated and the resulting crude vinyl ether was used in the next step without further purification.

The crude alkenyl ether was dissolved in MeOH (0.1 mL) and treated with TMSCl (2.6 µL, 20 µmol) at 0 °C. After stirring for 1.5 h, the mixture was diluted with MeOH (1.0 mL) and filtered through a plug of silica, which was rinsed with additional MeOH (5 mL). The combined filtrates were concentrated and the residue was purified by flash chromatography (hexane/EtOAc 1:2 to 1:4) to afford ketal **40** as a yellow oil; because of the sensitivity of the transient alkenyl ether, the yields were highly variable, ranging from 15-85%. $[\alpha]_D^{20}$ = + 12.9 (c = 0.07, CH_2Cl_2). ^{1}H NMR (600 MHz, $CDCl_3$) δ 6.33 – 6.28 (m, 1H), 6.26 (q, *J* = 6.8 Hz, 1H), 5.14 (ddt, *J* = 11.1, 7.7, 3.9 Hz, 1H), 4.54 (dd, *J* = 8.2, 2.5 Hz, 1H), 4.07 (qd, *J* = 9.0, 2.5 Hz, 1H), 3.89 (dq, *J* = 9.8, 5.2 Hz, 1H), 3.44 (dd, *J* = 10.2, 8.8 Hz, 1H), 3.31 (s, 1H), 3.21 (s, 3H), 3.15 (td, *J* = 9.6, 8.5, 2.9 Hz, 1H), 2.38 – 2.29 (m, 3H), 2.21 (td, *J* = 13.7, 3.5 Hz, 1H), 2.12 (tdd, *J* = 11.5, 5.4, 3.3 Hz, 1H), 2.02 – 1.88 (m, 2H), 1.90 – 1.81 (m, 1H), 1.80 (ddd, *J* = 14.4, 7.8, 4.3 Hz, 1H), 1.75 – 1.65 (m, 2H), 1.67 – 1.44 (m, 4H), 1.42 – 1.37 (m, 1H), 1.37 – 1.30 (m, 3H), 0.94 (d, *J* = 6.5 Hz, 3H), 0.88 (s, 9H), 0.07 (s, 3H), 0.06 (s, 3H). ^{13}C NMR (151 MHz, $CDCl_3$) δ 171.8, 137.5, 100.9, 84.5, 77.9, 76.6, 75.2,

74.3, 72.9, 70.7, 68.3, 47.6, 42.9, 42.8, 42.0, 34.6, 32.1, 31.6, 30.5, 28.2, 26.5, 26.0, 18.9, 18.2, 12.8, −4.1, −4.5. IR (film): 2955, 2925, 2854, 1728, 1666, 1462, 1377, 1365, 1259, 1197, 1039 cm^{-1}. MS (ESIpos) *m/z* (%): 705.2 (100 (M+Na)). HRMS (ESI): m/z calcd for $C_{29}H_{51}O_8SiNa$ $[M+Na]^+$: 705.2294, found: 705.2290.

(*Z,Z*)-Diene S12. Alkenyl stannane **46** (1.8 mg, 4.2 µmol) was added to a solution of alkenyl iodide **40** (1.9 mg, 2.8 µmol) in degassed DMF (0.1 mL). After cooling to 0 °C, a stock solution comprising [Pd(PPh$_3$)$_4$] (0.6 mg, 0.6 µmol), [Bu$_4$N][OPOPh$_2$] (3.8 mg, 8.3 µmol) and CuTC (1.1 mg, 5.6 µmol) in DMF (0.1 mL) was added at 0 °C and the resulting mixture was stirred for 2 h at room temperature. The reaction was quenched with pH 7 phosphate buffer (1 mL) and the resulting mixture was diluted with EtOAc (2 mL). The aqueous layer was separated and extracted with EtOAc (3 x 2 mL). The combined filtrates were washed with brine (5 mL), dried over Na_2SO_4 and concentrated. The residue was purified by flash chromatography (hexane/EtOAc 1:4) to afford the title compound as a yellow oil (1.1 mg, 62%). $[\alpha]_D^{20}$ = −11.7 (c = 0.06, CH_2Cl_2). ^{1}H NMR (600 MHz, $CDCl_3$) δ 6.39 (t, *J* = 11.1 Hz, 1H), 6.32 (t, *J* = 11.1 Hz, 1H), 6.27 (t, *J* = 11.1 Hz, 1H), 6.06 (d, *J* = 11.6 Hz, 1H), 5.52 (q, *J* = 8.1 Hz, 1H), 5.36 (dt, *J* = 17.6, 7.3 Hz, 2H), 5.15 (dq, *J* = 7.5, 3.8 Hz, 1H), 4.52 (dd, *J* = 8.2, 2.5 Hz, 1H), 4.09 – 4.03 (m, 1H), 3.83 – 3.77 (m, 1H), 3.65 (t, *J* = 6.9 Hz, 2H), 3.46 – 3.41 (m, 1H), 3.30 (d, *J* = 10.4 Hz, 1H), 3.21 (s, 3H), 3.17 – 3.12 (m, 1H), 3.06 – 3.00 (m, 1H), 2.45 – 2.38 (m, 1H), 2.33 (dt, *J* = 22.3, 7.6 Hz, 5H), 2.21 (td, *J* = 13.5, 3.3 Hz, 1H), 2.12 (tt, *J* = 10.6, 5.6 Hz, 1H), 1.90 – 1.81 (m, 1H), 1.84 – 1.76 (m, 4H), 1.75 – 1.66 (m, 2H), 1.65 – 1.49 (m, 5H), 1.45 – 1.35 (m, 1H), 1.37 – 1.27 (m, 2H), 1.27 – 1.23 (m, 2H), 0.94 (d, *J* = 6.5 Hz, 3H), 0.89 (s, 9H), 0.88 (s, 9H), 0.06 (s, 3H), 0.05 (s, 6H), 0.04 (s, 3H). ^{13}C NMR (151 MHz, $CDCl_3$) δ 171.7, 137.8, 129.2, 128.7, 126.1, 125.6, 125.1, 124.7, 121.2, 100.9, 78.0, 76.6, 75.2, 74.3, 73.0, 70.8, 69.6, 62.9, 47.6, 42.8, 42.0, 36.0, 34.8, 32.1, 31.5, 30.7, 30.5, 28.2, 26.5, 26.1, 26.1, 24.2, 22.9, 19.0, 18.5, 18.2, 12.8, −4.1, −4.5, −5.1 (2C). IR (film): 2956, 2926, 2873, 2854, 1728, 1462, 1440, 1377, 1280, 1261, 1201, 1096, 833 cm^{-1}. MS (ESIpos) *m/z* (%): 843.5 (100 (M+Na)). HRMS (ESI): m/z calcd for $C_{45}H_{80}O_9Si_2Na$ $[M+Na]^+$: 843.5233, found: 843.5242.

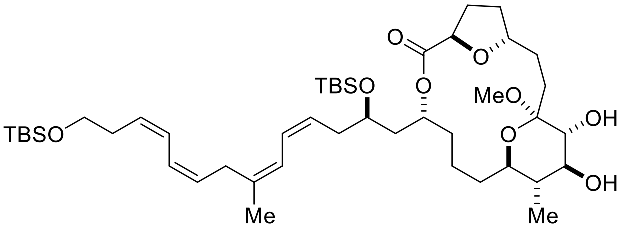

Formosalide A and B (**2**). A solution of TASF (11.7 mg, 43 µmol) and water (0.8 µL, 43 µmol) in DMF (0.1 mL) was added to a solution of compound **S12** (1.4 mg, 2 µmol) in DMF (0.1 mL) at 0 °C. the resulting mixture was stirred for 2 h at that temperature before the ice bath was removed and stirring was continued for a another 72 h at room

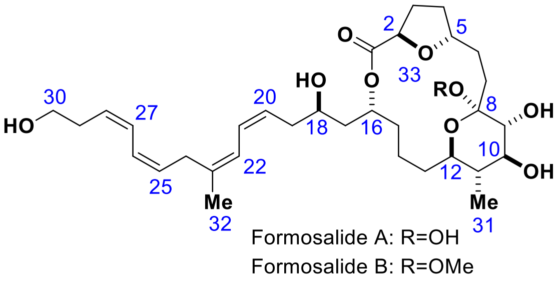

temperature. The mixture was diluted with EtOAc (2 mL) and pH 7 phosphate buffer (2 mL). The aqueous layer was separated and extracted with EtOAc (3 x 2 mL). The combined extracts were washed with brine (2 x 5 mL), dried over Na_2SO_4 and concentrated. Reaction control showed that partial hydrolysis of the glycoside has occured. Therefore the crude material was purified by preparative HPLC (YMC Triart C18, 5 µm, 150 × 20 mm, MeCN/H_2O = 40:60, v = 15 mL/min, λ = 220 nm, 27 °C, 118 bar) to afford Formosalide B (0.5 mg, 49%) and Formosalide A (0.4 mg, 41%) as a colorless oil each.

Analytical and spectroscopic data of Formosalide B (**2**): $[\alpha]_D^{24}$ = +15 (c = 0.02, MeOH) [lit.:[13] +18.8 (c 0,04, MeOH)]. ^{1}H NMR (600 MHz, $CDCl_3$) δ see Table S-11. ^{13}C NMR (151 MHz, $CDCl_3$) δ see Table S-11. IR (film): 3403, 2929, 1717, 1651, 1416, 1204, 1092, 1038 cm^{-1}. MS (ESIpos) *m/z* (%): 615.4 (100 (M+Na)). HRMS (ESI): m/z calcd for $C_{33}H_{52}O_9Na$ $[M+Na]^+$: 615.3504, found: 615.3503

Analytical and spectroscopic data of Formosalide A (**1**): $[\alpha]_D^{24}$ = +13.3 (c = 0.04, MeOH) [lit.:[13] +17.3 (c 0.01, MeOH)]. ^{1}H NMR (600 MHz, $CDCl_3$) δ see Table S-14. ^{13}C NMR (151 MHz, $CDCl_3$) δ see Table S-14. IR (film): 2954, 2921, 2851, 1744, 1607, 1576, 1517, 1463, 1378, 1312, 1246, 1171, 1088, 1019 cm^{-1}. MS (ESIpos) *m/z* (%): 601.3 (100 (M+Na)). HRMS (ESI): m/z calcd for $C_{32}H_{50}O_9Na$ $[M+Na]^+$: 601.3351 , found: 601.3347

Table S-11. Analysis of the NMR data of synthetic **2**; arbitrary numbering scheme as shown in the insert.

The signal assignment corrects some of the assignments made in the literature (6 synth. = 13 of lit.; 7 synth. = 6 of lit.; 13 synth. = 15 of lit.; 15 synth. = 7 of lit.)[13]

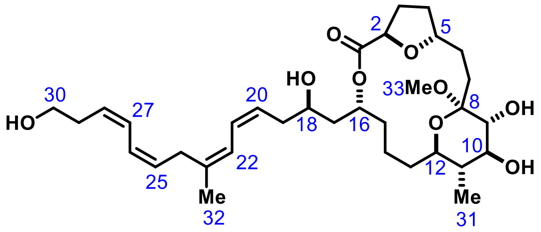

Formosalide B (**2**)

atom n°	^{1}H NMR (600 MHz, $CDCl_3$)[a]					^{13}C NMR (150 MHz, $CDCl_3$)[a]	
	δ [ppm]	m	*J* [Hz]	COSY	NOESY	δ [ppm]	HMBC
1	-					173.4	3b
2	4.57	dd	8.0, 2.3	3b	3a, 3b	77.6	3b
3a	2.34	m	-	3b, 4ab		26.3	-
3b	1.95	m		2, 3a, 4b			
4a	2.09	m		3a, 4b, 5	4b, 5	31.8	2
4b	1.33	m		3a, 4a, 5	4a		
5	4.02	m	-	4ab, 6b	4a, 6a, 9	76.6	2
**6a**	1.84	td	13.5, 3.2	5, 6b, 7a, 7b	5, 9	**30.4**	7b
**6b**	1.55	m		5, 6b, 7a, 7b			
**7a**	2.19	m		6a, 6b, 7b	33	**27.9**	-
**7b**	1.70	m		6a, 6b, 7a			

8	-					100.7	33
9	3.28	t	9.4	9-OH, 10	11	74.2	6
10	3.42	t	9.5	9, 11, 10-OH	12, 31	74.9	12, 31
11	1.35	m		10, 12, 31	9	42.2	31
12	3.13	td	9.6, 2.8	11, 13b	10, 31	72.8	11, 31
**13a**	1.50	m		12, 13b, 14a,b		**31.1**	-
**13b**	1.25	m		12, 13a			
14a	2.01	m		13a, 14b, 15a		19.6	-
14b	1.29	m		13a, 14a, 15b			
**15a**	1.81	m		14a, 15b, 16		**33.9**	-
**15b**	1.55	m		14b, 15a, 16			
16	5.21	t	10.6	16a, 17a		70.1	-
17a	1.69	m		16, 17b		43.1	-
17b	1.56	m		17a, 18			
18	3.50	m	-	17b, 18, 19	18-OH	67.2	19
19	2.33	m	-	18, 20		35.0	-
20	5.38	m	-	19, 21	21	125.6	19
21	6.30	m	-	20, 22	20, 24	126.3	19
22	6.07	d	11.5	21	32	120.8	20, 24, 32
23	-					137.9	21, 24, 32
24	3.02	m	-	25	21, 27, 32	30.6	32
25	5.40	m	-	24, 26	26	129.8	24
26	6.32	m	-	25, 27	25	124.2	24, 27, 28
27	6.47	t	11.2	26, 28	24, 28	126.2	29
28	5.49	m		27, 29	27	127.9	29, 30
29	2.46	dtd	7.4, 5.9, 1.3	28, 30		31.1	-
30	3.68	q	6.0	29, 30-OH		62.2	29
31	0.94	d	6.5	11		12.7	11
32	1.77	s	-	-	22, 24	24.1	22, 24
33	3.20	s	-	-		47.5	9
10-OH	2.27	s					
9-OH	1.90	d	10.4	9			
18-OH	3.02	s			16		
30-OH	1.34	s		30			

Spectral Comparisons

Table S-12. Comparison of the ^{13}C NMR spectra of synthetic **2** and authentic Formosalide B.[13]

Only for the sake of comparison, the signal assignment follows the literature;[13] *note, however, that several assignment need to be corrected (6 synth. = 13 of lit.; 7 synth. = 6 of lit.; 13 synth. = 15 of lit.; 15 synth. = 7 of lit.)*[13]

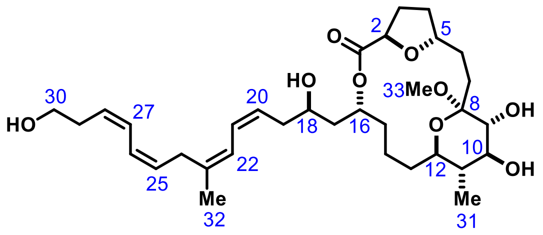

Formosalide B (**2**)

Position	δ (isolated)	δ (synthetic)	Δδ (i-s)
1	173.4	173.4	0.0
2	77.6	77.6	0.0
3	26.3	26.3	0.0
4	31.7	31.8	−0.1
5	76.6	76.6	0.0
6*	27.9	**27.9**	0.0
7*	33.9	**33.9**	0.0
8	100.7	100.7	0.0
9	74.2	74.2	0.0
10	74.9	74.9	0.0
11	42.2	42.2	0.1
12	72.8	72.8	0.0
13*	30.4	**30.4**	0.0
14	19.6	19.6	0.0
15*	31.1	**31.1**	0.0
16	70.1	70.1	−0.0
17	43.0	43.1	−0.1
18	67.2	67.2	0.0
19	35.0	35.0	0.0
20	125.6	125.6	0.0
21	126.3	126.3	0.0
22	120.8	120.8	0.0
23	137.9	137.9	−0.1
24	30.6	30.6	0.0
25	129.7	129.8	−0.1
26	124.2	124.2	0.0
27	126.1	126.2	−0.1
28	127.9	127.9	0.0
29	31.0	31.1	−0.1
30	62.2	62.2	0.0
31	12.7	12.7	0.0
32	24.1	24.1	0.0
33	47.5	47.5	0.0

Graphical comparison of ^{13}C NMR spectra of synthetic **2** (black) and the simulated spectra of the literature shifts (red) of Formosalide B

0.5mg $CDCl_3$ 298 K

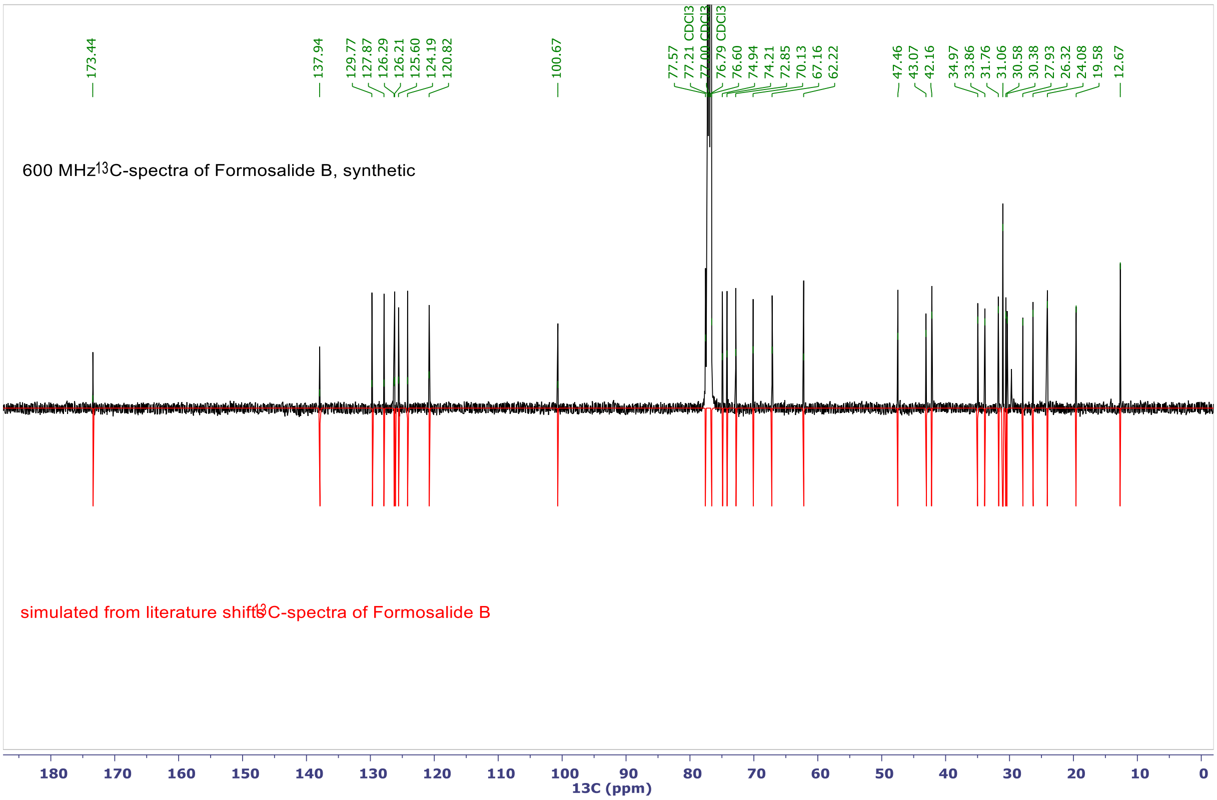

0.5mg $CDCl_3$ 298 K

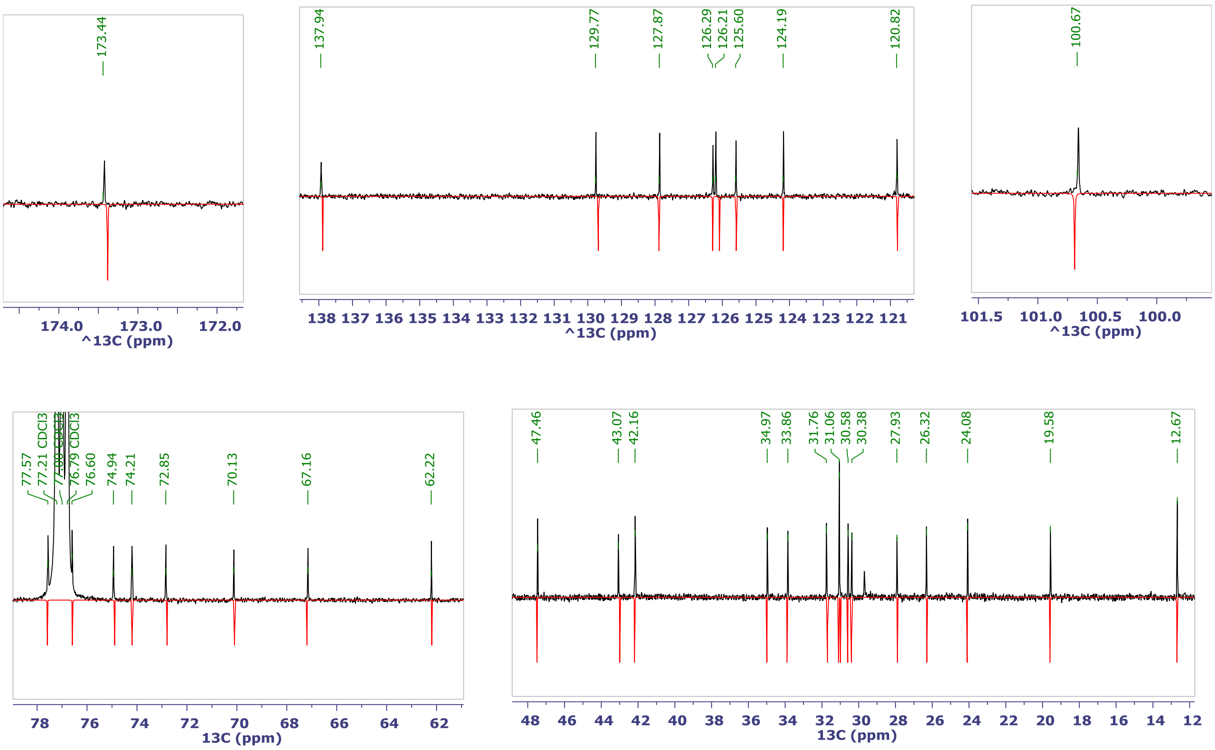

Table S-13. Comparison of the ^{1}H NMR spectra of synthetic **2** and authentic Formosalide B.[13]

Only for the sake of comparison, the signal assignment follows the literature;[13] *note, however, that several assignment need to be corrected (*6 *synth.* = 13 *of lit.;* 7 *synth.* = 6 *of lit.;* 13 *synth.* = 15 *of lit.;* 15 *synth.* = 7 *of lit.)*[13]

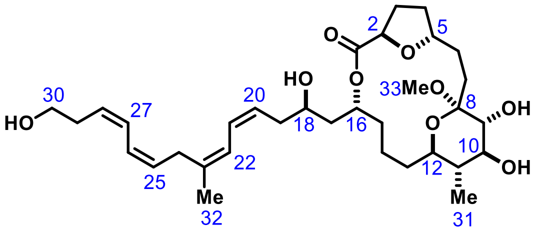

Formosalide B (**2**)

Position	δ (isolated)	δ (synthetic)	Δδ (i-s)
2	4.55	4.57	−0.02
3a	2.33	2.34	−0.01
3b	1.95	1.95	0.00
4a	2.08	2.09	−0.01
4b	1.32	1.33	−0.01
5	4.02	4.02	0.00
6a	2.19	**2.19**	0.00
6b	1.70	**1.72**	−0.02
7a	1.80	**1.81**	−0.01
7b	1.55	**1.56**	−0.01
9	3.28	3.28	0.00
10	3.42	3.42	0.00
11	1.34	1.35	−0.01
12	3.13	3.13	0.00
13a	1.83	**1.84**	−0.01
13b	1.55	**1.55**	0.00
14a	2.01	2.01	0.00
14b	1.28	1.29	−0.01
15a	1.50	**1.51**	−0.01
15b	1.27	**1.26**	0.01
16	5.20	5.21	−0.01
17a	1.67	1.69	−0.02
17b	1.57	1.56	0.01
18	3.50	3.50	0.00
19	2.32	2.33	−0.01
20	5.40	5.38	0.02
21	6.30	6.30	0.00
22	6.06	6.07	−0.01
24	3.01	3.02	−0.01
25	5.39	5.39	0.00

26	6.31	6.32	−0.01
27	6.46	6.47	−0.01
28	5.49	5.49	0.00
29	2.45	2.46	−0.01
30	3.66	3.68	−0.02
31	0.94	0.94	0.00
32	1.76	1.77	−0.01
33	3.20	3.20	0.00

Table S-14. Analysis of the NMR data of synthetic **1**; arbitrary numbering scheme as shown in the insert.

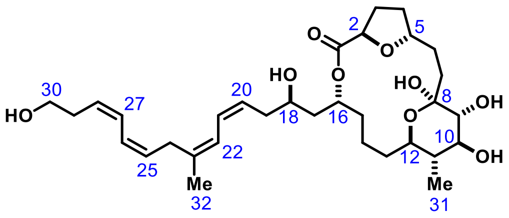

Formosalide A (**1**)

atom n°	^{1}H NMR (600 MHz, $CDCl_3$)[a]					^{13}C NMR (150 MHz, $CDCl_3$)[a]	
	δ [ppm]	m	*J* [Hz]	COSY	NOESY	δ [ppm]	HMBC
1	-					173.63	2a, 3a, 3b
2	4.59	dd	8.3, 5.5	3ab	3b	77.66	3a, 3b
3a	2.32	m		2, 3b, 4b	-	27.37	2, 4b
3b	2.15	m		2, 3a, 4b	2		
4a	2.13	m		4b, 5	5	32.61	2, 3a
4b	1.48			3ab, 4a, 5	-		
5	4.08	m		4ab, 6	4a	79.83	2, 3b, 4b, 7a
6ab	1.79	m		5, 7ab		**29.85**	4b, 7a, 7b
7a	2.28	m		6, 7b	9	**35.22**	
7b	1.67	ddd	14.8, 12.1, 4.3	6, 7a	9		
8	-					97.84	6, 7ab
9	3.05	m		9-OH, 10	7ab, 11	77.51	8-OH, 10
10	3.41	dd	9.0, 10.2	9, 11	12, 31	75.51	11, 12, 31
11	1.38	m	6.5, 10.4	10, 12, 31	9, 31	42.02	10, 31
12	3.53	t	10.4	11, 13b	10, 15a, 31	70.24	11, 13b, 31
13a	1.45	m		13b, 14ab		**30.76**	11, 14a, 15a
13b	1.35	m		12, 13a, 14ab			
14a	1.47	m		13ab, 14b		19.86	12
14b	1.43	m		13ab, 14a			
15a	1.57	m				**34.09**	13a,b
15b	1.52	m					

16	5.04	tt	10.6, 1.9	17ab	15b, 17b	72.89	17a,b
17a	1.75	m		16, 17b	18	42.63	19ab
17b	1.58	m		16, 17a, 18	16		
18	3.54	m	3.7	17b, 18 OH, 19ab	17a, 19a, 20	67.29	17b, 19ab
19a	2.37	m	7.6	18, 19b, 20	18, 22	35.09	21
19b	2.30	m	7.6	18, 19a, 20	20, 22		
20	5.37	dt	7.6, 11.2	19ab, 21	18, 19b, 21	125.36	19a,b, 22
21	6.31	ddm	11.2, 11.4	20, 22	20, 24	126.52	19ab, 22
22	6.07	d	11.4	21, 32	19ab, 32	120.72	20, 21, 24, 32
23	-					138.08	21, 24, 32
24	3.02	d	7.7	25	21, 25, 27, 32	30.59	22, 26, 32
25	5.40	dtt	7.7, 10.9, 1.3	24, 26	24, 26, 32	129.72	24, 27
26	6.31	ddm	11.3, 10.9	25, 27	25, 29	124.13	24, 27, 28
27	6.47	tq	11.3, 11.0, 1.4	26, 28	24, 28	126.19	25, 26, 29
28	5.50	dtt	11.0, 7.7, 1.3	27, 29	27, 29, 30	127.84	26, 29, 30
29	2.46	dtd	7.7, 6.4, 1.5	28, 30	26, 28	31.02	27
30	3.67	m	6.4	29, 30-OH	28	62.18	29
31	0.94	d	6.5	11	10, 11, 12	12.93	
32	1.77	m		22	22, 24, 25	24.09	22, 24
8-OH	4.44	s			10, 12		9
9-OH	1.91	br		9			
10-OH	2.30	m					
18-OH	2.86	d	3.70	18			
30-OH	1.41			30			

Table S-15. Comparison of the ^{13}C NMR Spectra of synthetic **1** and authentic Formosalide A.[13] Because the signals show a slight drift with time (see below), the shift values after 2 h acquisition time are tabulated.

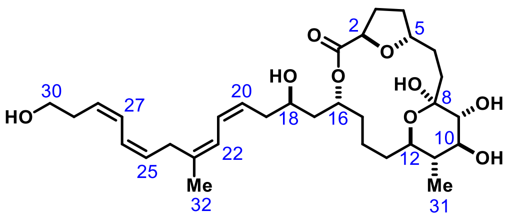

Formosalide A (**1**)

Position	δ (isolated)	δ (synthetic)	Δδ (i–s)
1	173.9	173.6	0.3
2	77.4	77.7	-0.3
3	27.8	27.4	0.4
4	32.2	32.6	-0.4
5	79.7	79.8	-0.1
6	29.5	29.8	-0.3
7	34.8	35.2	-0.4
8	98.1	97.8	0.3
9	77.3	77.5	-0.2
10	75.2	75.5	-0.3
11	42.3	42.0	0.3
12	70.3	70.2	0.1
13	30.9	30.8	0.1
14	20.0	19.9	0.1
15	33.9	34.1	-0.2
16	73.1	72.9	0.2
17	42.5	42.6	-0.1
18	67.3	67.3	0.0
19	35.1	35.1	0.0
20	126.0	125.4	0.6
21	126.1	126.5	-0.4
22	120.7	120.7	0.0
23	138.1	138.1	0.0
24	30.6	30.6	0.0
25	129.6	129.7	-0.1
26	124.2	124.1	0.1
27	125.4	126.2	-0.8
28	127.9	127.8	0.1
29	31.0	31.0	0.0
30	62.1	62.1	0.0
31	13.0	12.9	0.1
32	24.1	24.1	0.0

Slight drift of selected ^{13}C NMR signals with time: after equilibration for 6 d (up), after 2 h (down)

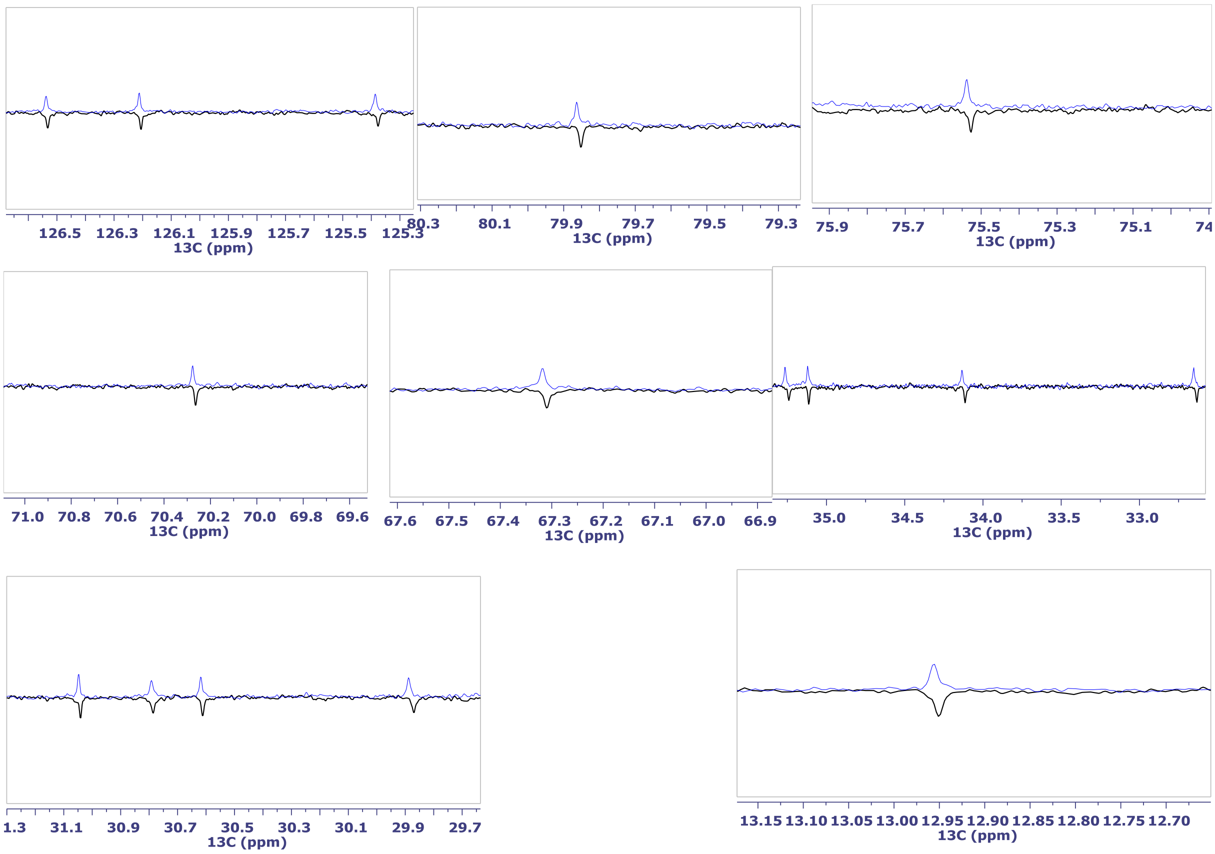

Graphical comparison of ^{13}C NMR spectra of synthetic **1** (black) and the simulated spectra of the literature shifts (red) of Formosalide A

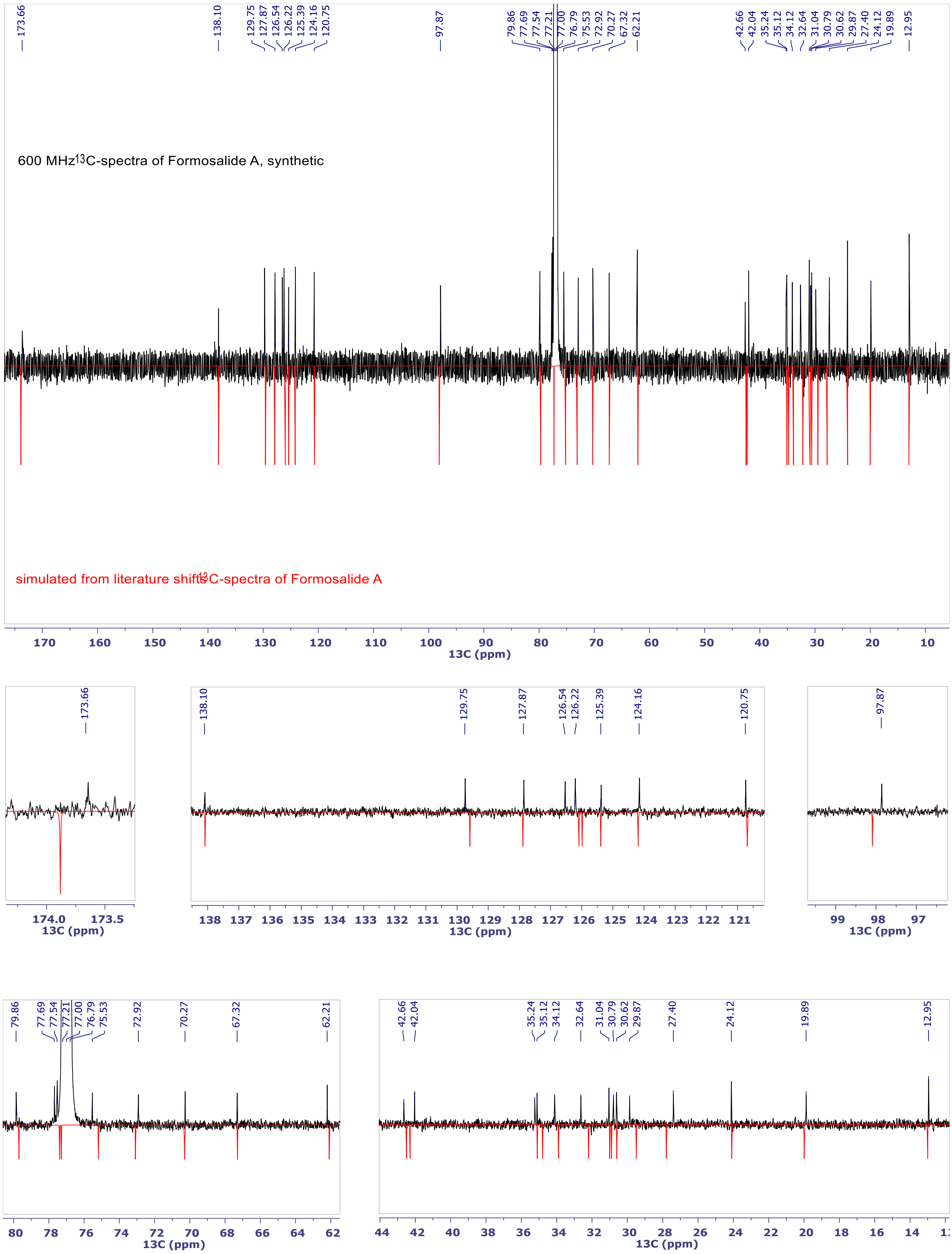

Table S-16. Comparison of the ^{1}H NMR Spectra of synthetic **1** and authentic Formosalide A.[13] The shift values after 2 h acquisition time are tabulated

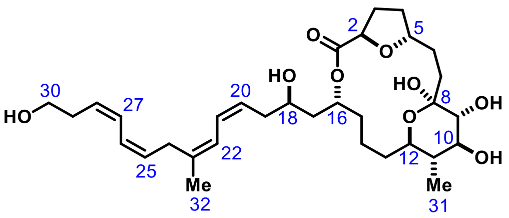

Formosalide A (**1**)

#	δ (isolated)	δ (synthetic)	Δδ (i-s)
2	4.58	4.59	-0.01
3a	2.26	2.32	-0.06
3b	2.16	2.15	0.01
4a	2.10	2.13	-0.03
4b	1.43	1.48	-0.05
5	4.15	4.08	0.07
6a	1.84	1.79	0.05
6b	1.70	1.79	-0.09
7a	2.24	2.28	-0.04
7b	1.61	1.67	-0.06
8 OH		4.44	
9	3.06	3.05	0.01
9 OH		1.91	
10	3.40	3.41	-0.01
10 OH		2.30	
11	1.35	1.38	-0.03
12	3.51	3.53	-0.02
13a	1.45	1.45	0
13b	1.33	1.35	-0.02
14a	1.43	1.47	-0.04
14b	1.43	1.43	0
15a	1.60	1.57	0.03
15b	1.46	1.52	-0.06
16	5.02	5.04	-0.02
17a	1.74	1.75	-0.01
17b	1.56	1.58	-0.02
18	3.54	3.54	0
18 OH		2.86	
19a	2.33	2.37	-0.04
19b	2.33	2.30	0.03
20	5.36	5.37	-0.01
21	6.29	6.31	-0.02
22	6.05	6.07	-0.02

24	3.0	3.02	-0.02
25	5.40	5.40	0
26	6.29	6.31	-0.02
27	6.46	6.46	0
28	5.50	5.50	0
29	2.44	2.46	-0.02
30	3.65	3.67	-0.02
30 OH		1.41	
31	0.92	0.94	-0.02
32	1.76	1.77	-0.01

References

1. Geary, L. M.; Woo, S. K.; Leung, J. C.; Krische, M. J., Diastereo- and Enantioselective Iridium-Catalyzed Carbonyl Propargylation from the Alcohol or Aldehyde Oxidation Level: 1,3-Enynes as Allenylmetal Equivalents. *Angew. Chem. Int. Ed.* **2012,** *51* (12), 2972-2976.
2. Aggarwal, V. K.; Roseblade, S. J.; Barrell, J. K.; Alexander, R., Highly Diastereoselective Nitrone Cycloaddition onto a Chiral Ketene Equivalent: Asymmetric Synthesis of Cispentacin. *Org. Lett.* **2002,** *4* (7), 1227-1229.
3. Schreiber, S. L.; Claus, R. E.; Reagan, J., Ozonolytic cleavage of cycloalkenes to terminally differentiated products. *Tetrahedron Lett.* **1982,** *23* (38), 3867-3870.
4. Bartko, S.; Deng, J.; Danheiser, R. L., Synthesis of 1-Iodopropyne. *Org. Synth.* **2016,** *93*, 245-262.
5. Calter, M. A., Catalytic, Asymmetric Dimerization of Methylketene. *J. Org. Chem.* **1996,** *61* (23), 8006-8007.
6. Valot, G.; Mailhol, D.; Regens, C. S.; O'Malley, D. P.; Godineau, E.; Takikawa, H.; Philipps, P.; Fürstner, A., Concise Total Syntheses of Amphidinolides C and F. *Chem. Eur. J.* **2015,** *21* (6), 2398-2408.
7. Valot, G.; Regens, C. S.; O'Malley, D. P.; Godineau, E.; Takikawa, H.; Fürstner, A., Total Synthesis of Amphidinolide F. *Angew. Chem. Int. Ed.* **2013,** *52* (36), 9534-9538.
8. Szostak, M.; Spain, M.; Procter, D. J., Selective synthesis of 3-hydroxy acids from Meldrum's acids using SmI2-H2O. *Nat. Protoc.* **2012,** *7* (5), 970-977.
9. Cummins, C. C., Reductive cleavage and related reactions leading to molybdenum–element multiple bonds: new pathways offered by three-coordinate molybdenum(III). *Chem. Commun.* **1998,** (17), 1777-1786.
10. Köpfer, A.; Breit, B., Rhodium-Catalyzed Hydroformylation of 1,1-Disubstituted Allenes Employing the Self-Assembling 6-DPPon System. *Angew. Chem. Int. Ed.* **2015,** *54* (23), 6913-6917.
11. Wang, G.; Negishi, E.-i., $AlCl_3$-Promoted Facile *E*-to-*Z* Isomerization Route to (Z)-2-Methyl-1-buten-1,4-ylidene Synthons for Highly Efficient and Selective (Z)-Isoprenoid Synthesis. *Chem. Eur. J.* **2009,** (11), 1679-1682.
12. Middleton, W. J., Tris(Dimethylamino)Sulfonium Difluorotrimethylsilicate. *Org. Synth.* **1986,** *64*.
13. Lu, C.-K.; Chen, Y.-M.; Wang, S.-H.; Wu, Y.-Y.; Cheng, Y.-M., Formosalides A and B, cytotoxic 17-membered ring macrolides from a marine dinoflagellate Prorocentrum sp. *Tetrahedron Lett.* **2009,** *50* (16), 1825-1827.
14. Jensen, T.; Pedersen, H.; Bang-Andersen, B.; Madsen, R.; Jørgensen, M., Palladium-Catalyzed Aryl Amination–Heck Cyclization Cascade: A One-Flask Approach to 3-Substituted Indoles. *Angew. Chem. Int. Ed.* **2008,** *47* (5), 888-890.
